# Role of Host-Guest Interaction in Understanding Polymerisation in Metal-Organic Frameworks

**DOI:** 10.3389/fchem.2021.716294

**Published:** 2021-07-21

**Authors:** A.D. Dinga Wonanke, Poppy Bennett, Lewis Caldwell, Matthew A. Addicoat

**Affiliations:** Department of Chemistry and Forensics, Nottingham Trent University, Nottingham, United Kingdom

**Keywords:** metal-organic frameworks (MOFs), polymerisation, host-guest interaction, DFTB, molecular dynamics

## Abstract

Metal-organic frameworks, MOFs, offer an effective template for polymerisation of polymers with precisely controlled structures within the sub-nanometre scales. However, synthetic difficulties such as monomer infiltration, detailed understanding of polymerisation mechanisms within the MOF nanochannels and the mechanism for removing the MOF template post polymerisation have prevented wide scale implementation of polymerisation in MOFs. This is partly due to the significant lack in understanding of the energetic and atomic-scale intermolecular interactions between the monomers and the MOFs. Consequently in this study, we explore the interaction of varied concentration of styrene, and 3,4-ethylenedioxythiophene (EDOT), at the surface and in the nanochannel of Zn_2_(1,4-ndc)_2_ (dabco), where 1,4-ndc = 1,4-naphthalenedicarboxylate and dabco = 1,4-diazabicyclo[2.2.2]octane. Our results showed that the interactions between monomers are stronger in the nanochannels than at the surfaces of the MOF. Moreover, the MOF-monomer interactions are strongest in the nanochannels and increase with the number of monomers. However, as the number of monomers increases, the monomers turn to bind more strongly at the surface leading to a potential agglomeration of the monomers at the surface.

## 1 Introduction

In recent years, the interest and effort in the synthesis, characterisation, functionalisation, modelling, and designing of novel nanoporous materials have gained a massive insurgence (Materazzi et al., 2008). This owes mostly to the fact that the properties of these materials are not only dependent on how atoms are arranged within their crystals, but also on the size and shape of their pores as well as on their specific surface area. For these reasons, nanoporous materials are heavily investigated for application in gas storage, sieving, filtration, extraction, separation, sensors, drug delivery, and electrochemical energy storage and catalysis (Bastani et al., 2013; Ma et al., 2014; Zhang et al., 2014; Forest et al., 2015; Zhang et al., 2015; Wilkerson and Ramesh, 2016; Fu et al., 2017; Rafiee and Shahebrahimi, 2017).

Hitherto, the most rapidly growing and investigated classes of nanoporous materials that hold potentials for an almost limitless range of applications are known as metal-organic frameworks, MOFs (Li et al., 1999; Yaghi et al., 2000; Yaghi et al., 2003; Mueller et al., 2006). MOFs are organic-inorganic hybrid crystalline porous materials that are formed by covalently binding metal ions or clusters, also known as secondary binding units, SBUs, with organic ligands, also known as linkers, in a variety of 2- and 3-dimensional nets or topologies (Furukawa et al., 2013; Butova et al., 2016). In general, they are materials with typical low mass densities, high internal surface area and large pore volumes. Consequently, they are exploited for several applications including (but not limited to) gas storage, filtration, extraction, separation, sensors, drug delivery, electrochemical energy storage, and catalysis (Furukawa et al., 2013; Ricco et al., 2016; Pettinari et al., 2017).

Moreover, the well-defined porous network and relatively high internal surface area have opened up new avenues for the use of MOFs as a template for various chemical reactions to obtain specific regio- and stereoisomers (Uemura et al., 2005; Liu et al., 2008; Bhakta et al., 2009; Canivet et al., 2011; Distefano et al., 2013; Lee et al., 2015; Chen et al., 2016; Ding et al., 2016; Wang et al., 2017; Mochizuki et al., 2018; Anan et al., 2019; Rivera-Torrente et al., 2019; Schmidt, 2019). Amongst these potential chemical reactions, polymerisation in MOFs has gained significant scientific interest. This is primarily because the highly designable features of MOFs result in nanochannels that can be applied as a tailor-made polymerisation system to obtain highly controlled polymer structures with long-range order. Furthermore, since the MOFs act only as a scaffold for reactions, conventional polymerisation methods can be easily employed, with little or no modification, provided that the reagents and reaction conditions do not destroy the crystal structures of the MOFs (Mochizuki et al., 2018).

So far, polymerisations in MOFs have been used to effectively control polymer molecular weight distribution, stereo-regularity (tacticity), reaction sites, and copolymer sequence (Uemura et al., 2008; Uemura et al., 2009). Consequently, this provides an attractive avenue for not only the precision synthesis of novel polymer materials but also for exploring specific properties of polymer confinement. A comprehensive review describing the state-of-the-art of polymerisation in MOFs was recently published (Schmidt, 2019).

Despite the advantages resulting from polymerisation in MOFs, there is a significant lack in conceptual understanding of how to effectively control these reactions, which consequently restrains their wide-scale application. (Uemura et al., 2009). Firstly, there is still an enormous synthetic challenge on how to effectively infiltrate the monomers in the confinement of the pores before polymerisation. Secondly, there is only very little understanding of the MOF nanochannel polymerisation mechanism, the initiation process and the propagation process. Thirdly, little is known on how the monomer interacts with the MOF framework during polymerisation as well as the mechanism for removing the MOF template (Wang et al., 2017).

The first evidence of polymerisation in MOFs was from the pioneering work of Uemura and co-workers on the radical polymerisation of styrene (Uemura et al., 2005). In this study, the styrene monomer was shown to fully infiltrate the nanochannel by immersing the MOF in the liquid monomer, while excess styrene at the external surface was removed by subjecting the host crystals to reduced pressure. The result from powder X-ray diffraction studies showed that the newly synthesised polymer was fully encapsulated in the nanochannel of the MOF. In a recent study, (Wang et al., 2017), Wang and co-workers performed an oxidative polymerisation of 3,4-ethylenedioxythiophene, EDOT, in a MOF. This time, the authors encountered a significant challenge in fully infiltrating the monomer into the MOF nanochannels and results from this study showed an agglomeration of monomers at the surface of the MOF.

**FIGURE 1:**
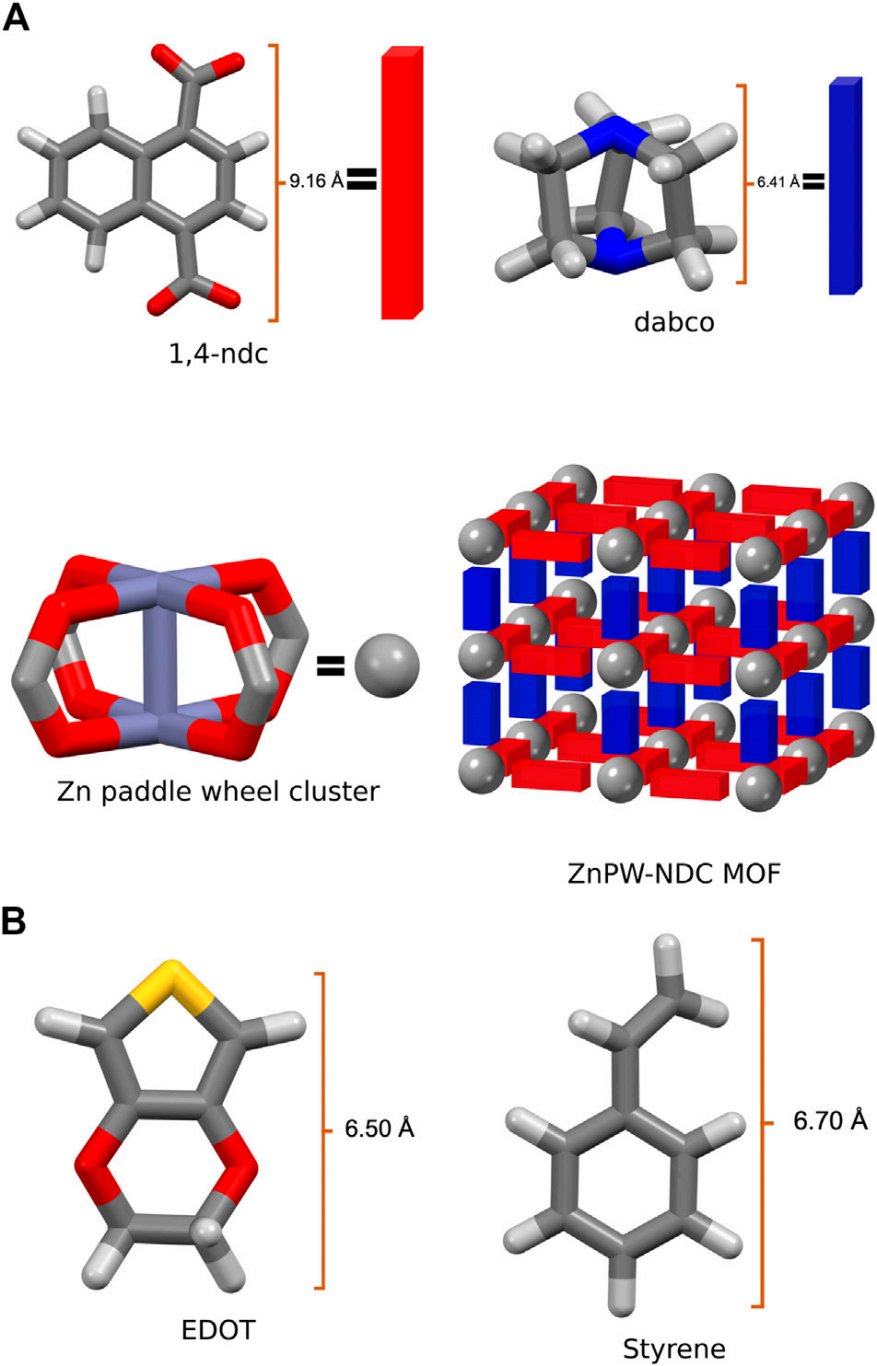
**(A)** the secondary building units of Zn^2^ (1,4-ndc)^2^ (dabco), represented as a net, where the organic linkers constitute the edges and the metal cluster the nodes, **(B)** Illustration of monomers EDOT and Styrene.

An in-depth understanding of the energetic and atomic-scale intermolecular interactions between the monomers both at the surface and in the nanochannels of the pores would be a significant step towards understanding how to fully control these reactions. Consequently in this study, we explore the interaction of styrene and EDOT, at the surface and in the nanochannel of the MOF, Zn_2_(1,4-ndc)_2_ (dabco), where 1,4-ndc = 1,4-naphthalenedicarboxylate and dabco = 1,4-diazabicyclo [2.2.2]octane. Zn_2_(1,4-ndc)_2_ (dabco), hereafter referred to as ZnPW-NDC MOF, is a MOF possessing a zinc paddlewheel building block on which the naphthalenedicarboxylate linkers are joined to form two-dimensional square grids, which are pillared by the dabco ligands as shown in Figure 1 (Klein et al., 2012).

## 2 Methods

To fully explore the intermolecular interactions between the monomers and the ZnPW-NDC MOF, 100 ZnPW-NDC MOF⊃monomers complexes were generated for ZnPW-NDC MOF⊃EDOT and ZnPW-NDC MOF⊃styrene complexes using one, two and three monomers both at the interior of the nanochannel and on the surface of the MOF. The ZnPW-NDC MOF lattice was constructed using the Automatic Topological Generator for Framework Structures package, implemented by one of us (Addicoat et al., 2014) The ZnPW-NDC MOF surface was then constructed by building a 3 × 3 × 1 supercell from and converting to a 2D slab by truncating the *c*-axis and capping with water. MOF⊃monomers complexes were then generated using a random structure generator algorithm, Kick (Addicoat et al., 2013), as described in the Electronic Supporting Information, ESI†. Once generated, all the complexes were optimised using Density Functional Tight Binding (DFTB) with znorg parameter set including UFF dispersion correction as implemented in the Amsterdam modelling suite, AMS, package version ADF 2019.305 (Hourahine et al., 2020; Moreira et al., 2009; Rappe et al., 1992; teVelde et al., 2001). Once optimised, the lowest energy complexes were selected and used to compute the MOF⊃monomers binding energies, BE, using the formula in Eq. 1.

$$BE=E_{MOF⊃monomers}-\left(E_{MOF}+xE_{Monomer}\right)$$ (1)

*E*_*MOF*⊃*monomers*_ is the ground state optimised energy of the MOF⊃monomer complex. *E_MOF_* is the ground state optimised energy of the isolated MOF. *E_Monomer_* is the ground state optimised energy of the monomer and finally, *x* represents the number of monomers (x = 1, 2 or 3) present in each complex.

A molecular dynamics, MD, simulation was then performed on all the optimal MOF⊃monomer composites in order to gauge the intermolecular interaction between the MOF and the monomer over a given time period. The MD simulations were computed using the same znorg parameter set at 343.1 K, 1 atm in the isothermal-isobaric (NPT) ensemble as implemented in DFTB+ program package (Hourahine et al., 2020). The temperature and pressure in this ensemble were maintained using Nosé-Hoover thermostat and the Berendsen barostat respectively (Berendsen et al., 1984; Martyna et al., 1996). The coupling strength used in thermostat corresponded to the maximum frequency for each composite meanwhile a decay constant of 10 femtoseconds was used for the Berendsen barostat. A time step of 1 femtosecond was used and the trajectory was saved for every time step. A total of 100,200 MD time steps were performed for each of the optimal MOF⊃monomer composites constituting the nanochannels. Meanwhile 10,200 MD time steps were performed for the composites constituting the surface.

All computational data corresponding to detailed energetics, intermolecular interactions and MD trajectories can be freely downloaded from http://doi.org/10.5281/zenodo.4382475.

## 3 Results and Discussions

### 3.1 Binding Energy Analysis

The binding energies for the most stable MOF⊃monomer(s) composites are presented in Table 1. The magnitude of these binding energies is a direct measure of the strength of intermolecular interactions between the MOF and the monomers. Hence it can be inferred from these results that all the monomers have an obvious attractive interaction with the ZnPW both in the nanochannel and at the surface.

**TABLE 1:** UFF-Zenorg binding energies for the most stable MOF⊃monomer composites.

BE (kJ mol^−1^)	Nanochannel		Surface	
No of monomers	ZnPW⊃EDOT	ZnPW⊃styrene	MOF⊃EDOT	MOF⊃styrene
1	−121.55	−107.96	−80.28	−114.74
2	−260.49	−228.96	−152.44	−152.99
3	−422.30	−362.63	−198.55	−228.34

In all cases, the strength of this intermolecular interaction increases with the number of monomers. The interactions are generally observed to be stronger in the nanochannels with ZnPW⊃EDOT showing the strongest interactions which are seen to double as the concentration of monomer increases. In the presence of one monomer, styrene is observed to bind more strongly at the surface, indicating a more favourable surface interaction at lower concentrations, possibly impacting the incursion of the monomer into the MOF nanochannels by blocking the surface. This significantly large stability resulting from the increase in monomer concentration could be a bottleneck when it comes to extracting the polymer from the MOF. Within the nanochannels, the increase in stability can partly be attributed to strong monomer-monomer interaction, which was further analysed from MD simulations.

### 3.2 Pairwise Root-Mean-Square Deviation

**FIGURE 2:**
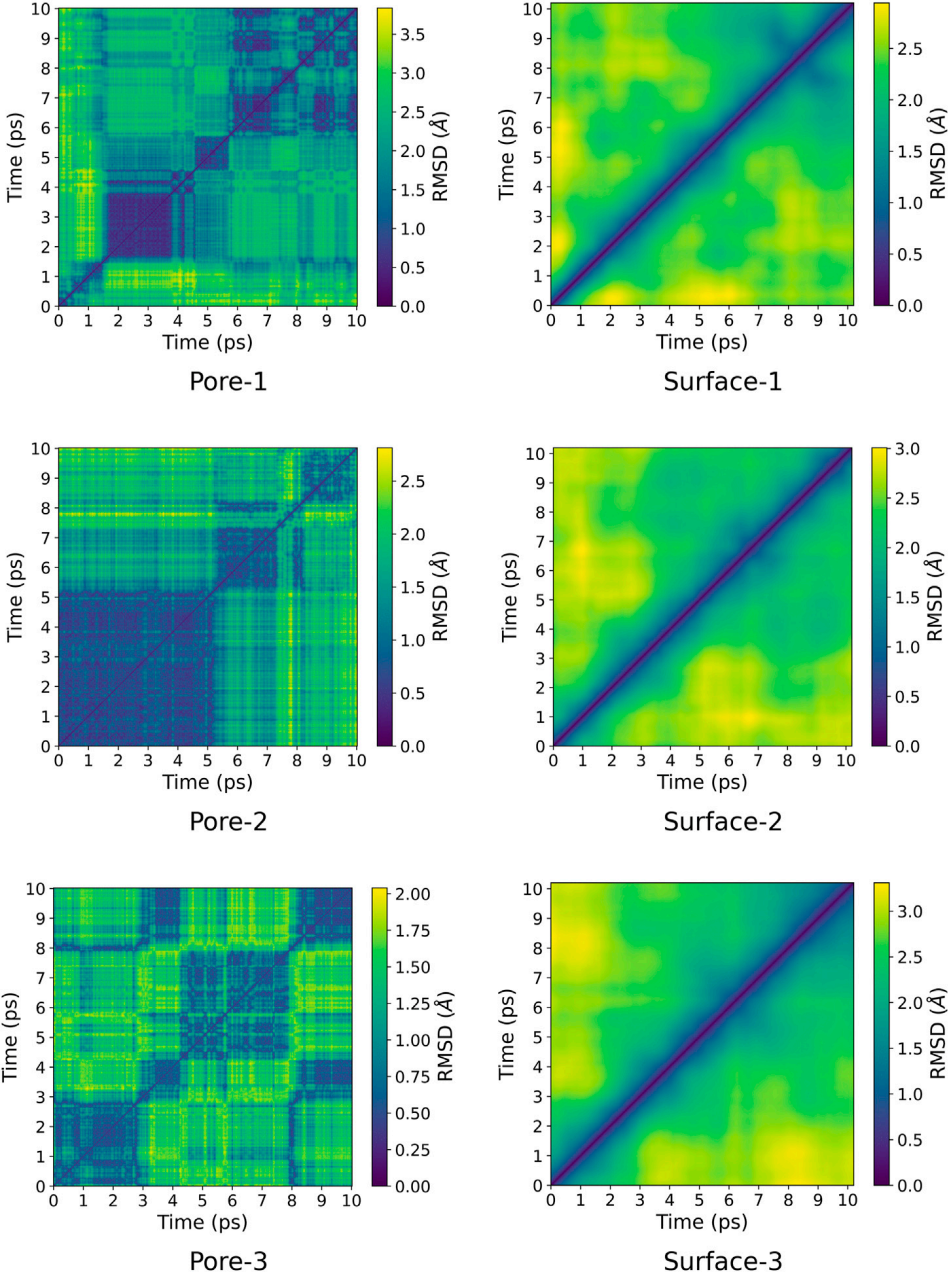
Pairwise RMSD plot for ZnPW⊃EDOT interactions for both nano-channel and surface interaction.The colour gradient corresponds to the RMSD in Å. The labels ”Pore” and ”Surface” correspond to nano-channel and surface interactions respectively and the numbers after the hyphen correspond to the number of monomer(s) present.

**FIGURE 3:**
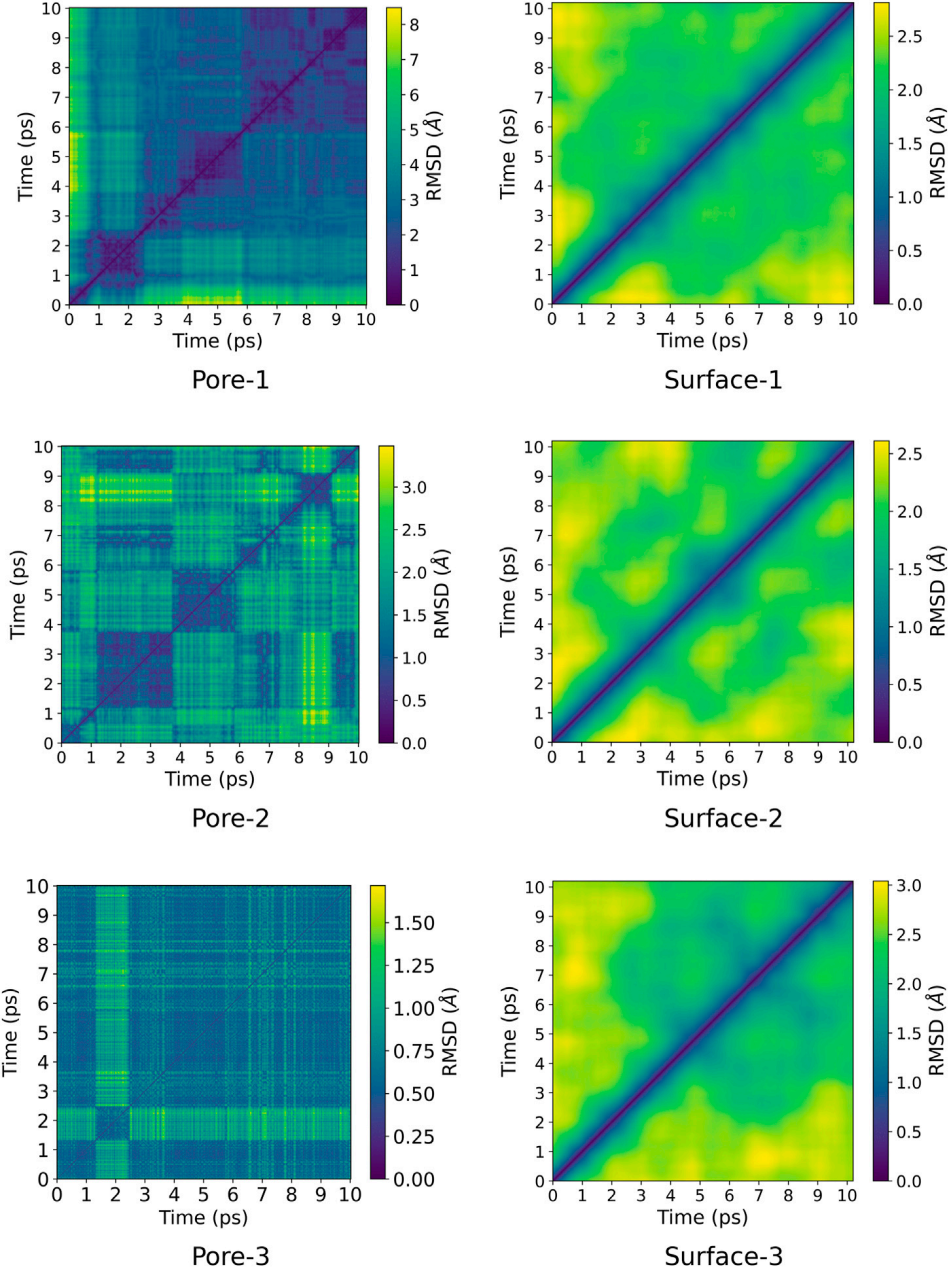
Pairwise RMSD plot for ZnPW⊃styrene interactions for both nano-channel and surface interaction.The colour gradient corresponds to the RMSD in Å. The labels ”Pore” and ”Surface” correspond to nano-channel and surface interactions respectively and the numbers after the hyphen correspond to the number of monomer(s) present.

A pairwise root-mean-square deviation (RMSD) was performed to provide a visual inspection of how each structure changes over time. In the pairwise RMSD, we compute the RMSD of each snapshot in the trajectory with respect to all the other snapshots. The RMSD along the diagonals have values of zero, which correspond to the RMSD of a snapshot with itself. Low RMSD values at the off-diagonal regions correspond to snapshots whose structures are similar to the reference snapshot, while higher values correspond to dissimilar structures. Consequently, occupation of a given state can be observed as blocks of similar RMSDs along the diagonal. The pairwise RMSD for all the MD trajectories are presented in Figures 2, 3, wherein the snapshots are converted into picoseconds, ps and RMSD presented in Ångstrom, Å.

The RMSD for every point in Figures 2, 3 were computed using the formula in Eq. 2 and the python script can freely be downloaded (https://github.com/bafgreat/Pairwise-RMSD).

$$RMSD_{\left(r,s\right)}=\sqrt{\frac{1}{N}\sum_{j=1}{\left|r_{j}-s_{j}\right|}^{2}}$$ (2)

Here, $r_{j}$ and $s_{j}$ are Cartesian coordinates of atom *j* in configuration *r* and *s* respectively, which have been optimally aligned so that the resulting RMSD should be the minimum distance between both configurations. *N* is the total number of atoms in each configuration. The pairwise RMSD was calculated for every 10^th^ snapshots in the MD trajectory for the nano-channel interactions, which reduces the total number of snapshots to 10,200, leading to a significant decrease in computational cost. On the other hand, the RMSD was computed for all the snapshots in the surface interaction for which each trajectory was composed of 10,200 snapshots.

**FIGURE 4:**
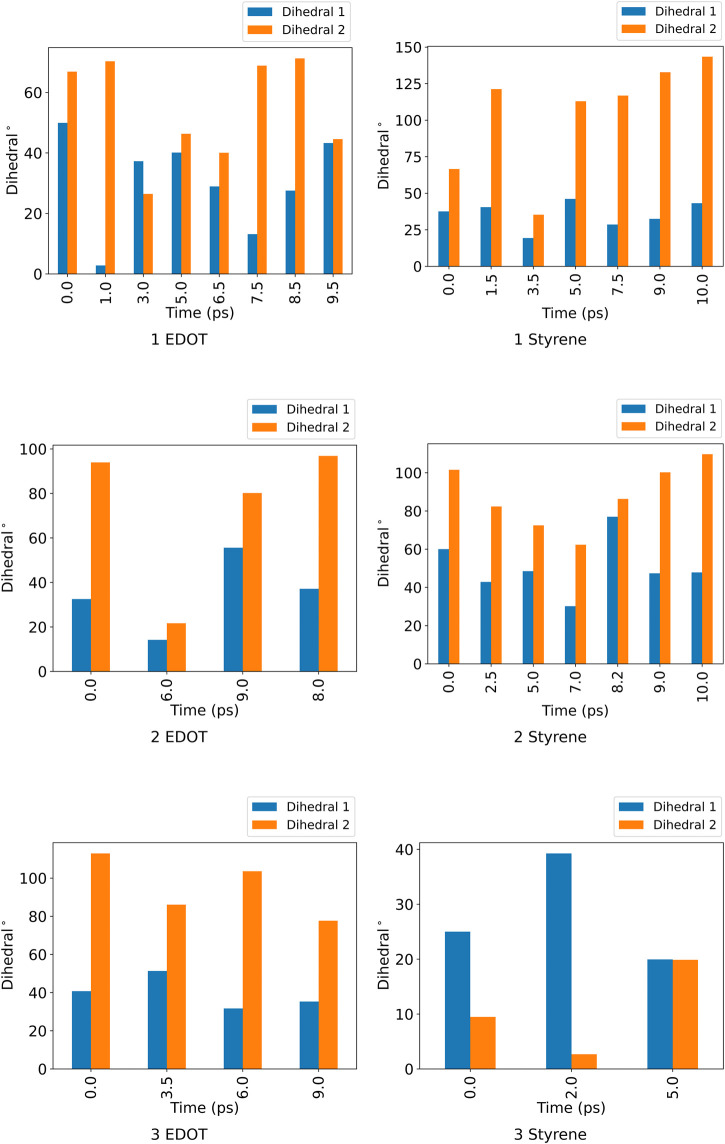
Dihedral angles for the two pairs positionally distinct ndc linker in the unit cell for the distinct conformational states of ZnPW⊃EDOT and ZnPW⊃styrene motifs for nano-channel interaction.

The pairwise RMSD for the nano-channels of both ZnPW⊃EDOT and ZnPW⊃styrene presented in Figures 2, 3 respectively, shows a series of distinct states that are sampled by the system. Here, regions of similar RMSDs are distinctively represented in blocks, corresponding to distinct conformational states. These states differ by the position of the monomer(s) in the nano-channel, as well as in the orientation of the naphthyl of the ndc linker, which determines the dimension of the pore. The orientations of these linkers can be determine by the computing their dihedrals with respect to the plane along the lattice coordinate on which they lie as described in the ESI. The dihedral angles for these distinct conformational states is presented in Figure 4.

For the ZnPW⊃EDOT system containing two monomers, Figure 2, the initial state persists for a longer period before transitioning into the second state. In this state, the dihedral angles formed by one of the ndc linkers are nicely aligned to enable a strong-sandwich π-π intermolecular interaction with one of the EDOT monomers, meanwhile the other monomer interacts favourably with the dabco pillar. Meanwhile, for the ZnPW⊃styerene system containing three monomers, Figure 3, the system visits 3 states and remains in the 3^rd^ state for over 7 ps The dihedral angles for both positionally distinct linkers are observed to be below $20^{\circ }$, which results in an open pore system in which all three styrene monomer sit in the centre of the pore enhancing both monomer-monomer interactions as well as MOF-monomer interactions.

At the surface, the transitions from one conformational state to the other are far less distinct. Here, the snapshots show more dissimilarity across the trajectory, indicative of less favourable interactions for which the systems may want to explore for a longer time scale. In the presence of three monomers, there are few regions of similar RMSD at larger time scale.

### 3.3 Radial Distribution Analysis

**FIGURE 5:**
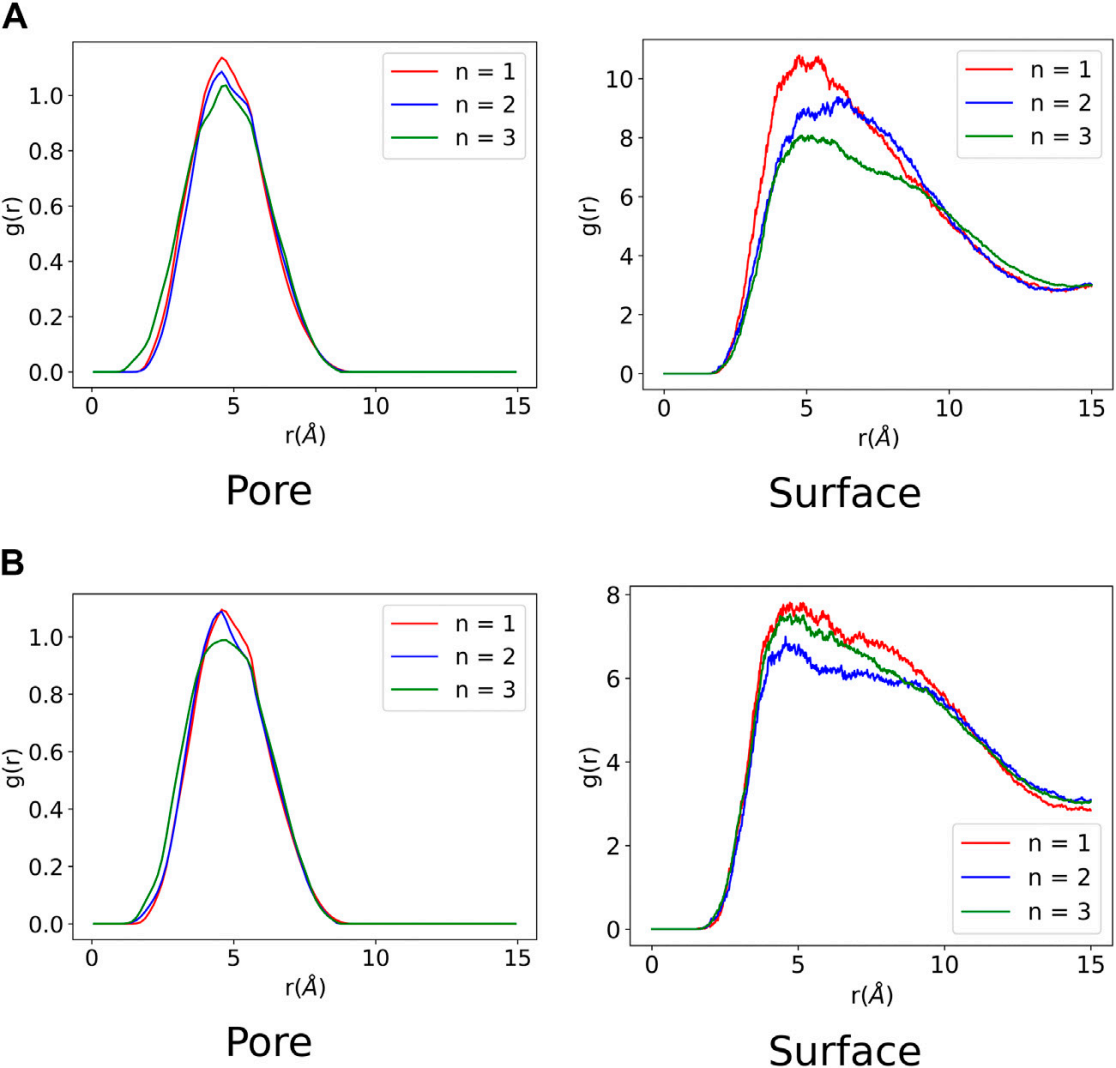
Radial distribution function of ZnPW⊃monomer(s) interactions computed from each molecular dynamic trajectory. *n* corresponds to the number of monomer(s) present in the trajectory. The labels ”Pore” and ”Surface” correspond to nanochannel and surface interactions respectively. **(A)** Nanochannel and surface ZnPW_EDOT radial distribution function. **(B)** Nanochannel and surface ZnPW_styrene radial distribution function.

**FIGURE 6:**
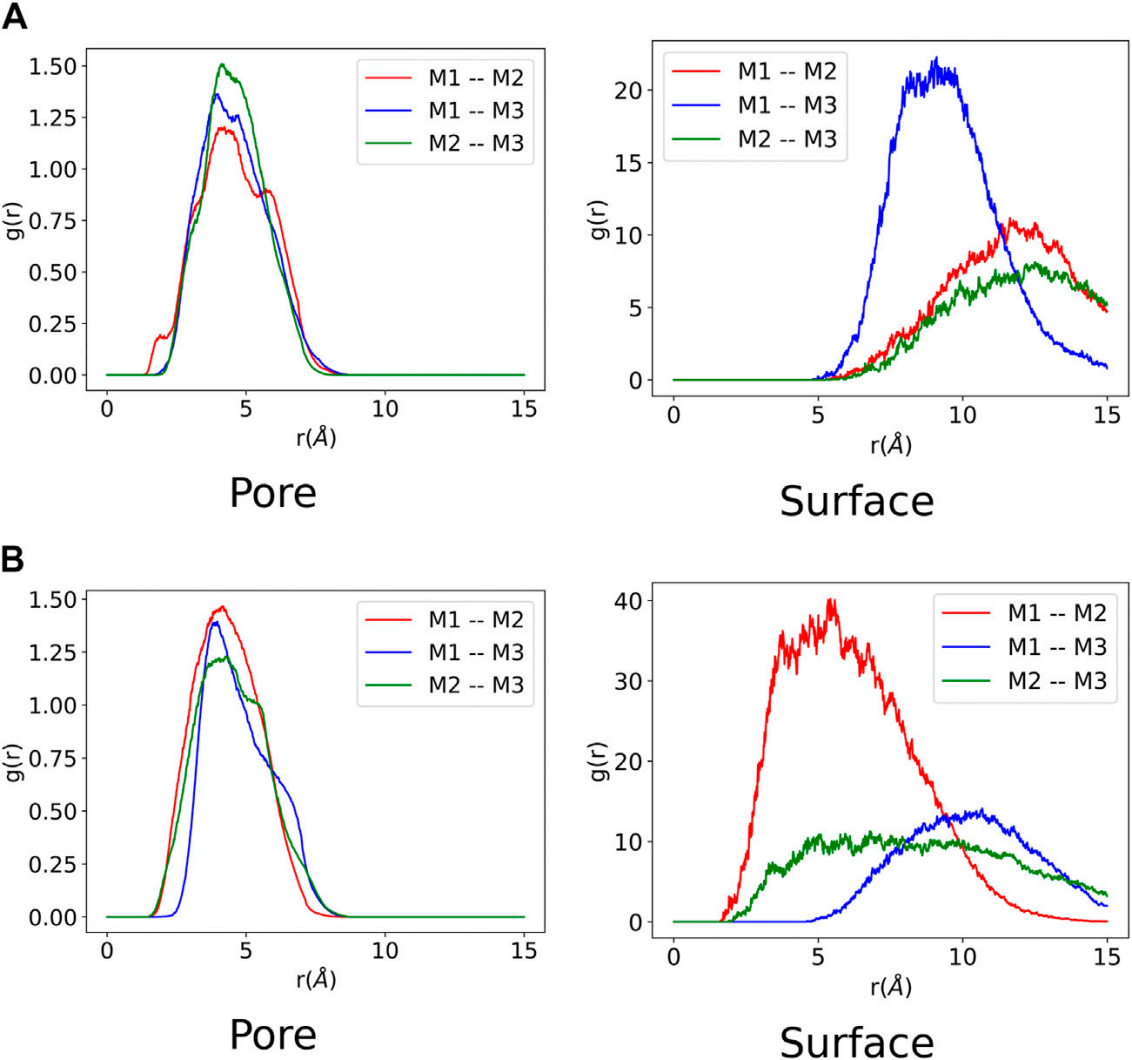
Monomer-monomer radial distribution function from each molecular dynamic trajectory composed of three monomers. Each monomer is labelled $M_{i}$ (i = 1, 2 or 3). The labels ”Pore” and ”Surface” correspond to interactions at the nanochannel and surface respectively. **(A)** EDOT-EDOT radial distribution function at nanochannel and surface of ZnPW. **(B)** Styrene-styrene radial distribution function at nanochannel and surface of ZnPW.

A radial distribution function (RDF) was computed for all the MD trajectories to analyse the ZnPW-NDC MOF⊃monomer(s) and monomer–monomer intermolecular interactions. The RDF was computed using MDAnalysis program package (Michaud-Agrawal et al., 2011; Gowers et al., 2016) and an excellent description of the implementation of RDFs from MD trajectories can be found in the paper by Kohlmeyer and co-workers (Levine et al., 2011) In the RDF analysis, the distance between pairs of atoms of the interacting species in each trajectory snapshot are computed and collected into a histogram, which provides the probability distribution for the interacting species to be found at a given distance in space. The RDF for the ZnPW-NDC MOF⊃monomer(s) and the monomer-monomer intermolecular interactions are presented in Figures 5, 6 and respectively.

It can be observed from Figure 5 that all the monomers have favourable intermolecular interactions with the ZnPW both at the surface and in the nanochannels, which are within the Van der Waals intermolecular range (r < 3.5 Å). However for EDOT, it can be observed from Figure 5A that as the number of monomers increases to three, the number of interactions below 2Å increases both at the surface and in the nanochannel, represented by the 3 green peaks. A similar observation is seen for the surface interaction of ZnPW⊃styrene presented in Figure 5B. This high propensity for the formation of strong intermolecular interactions at the surfaces of ZnPW when the monomer concentrations increases can be used as a proxy for explaining the experimental difficulties encountered in infiltrating the monomers into the nanochannels (Uemura et al., 2005; Wang et al., 2017). Moreover, the strong interaction (represented by the 3 green peaks in Figure 5A) occurring in the nanochannel of ZnPW-NDC MOF⊃EDOT in the presence of three monomers could also explain the relative difficulties encountered during the removal of the ZnPW-NDC MOF from the PEDOT-MOF composite (Wang et al., 2017).

The monomer-monomer interactions for systems containing three monomers are presented in Figure 6. It can be observed for both ZnPW-NDC MOF⊃EDOT and ZnPW-NDC MOF⊃styrene that the monomers have a stronger interaction with the ZnPW-NDC MOF in the nanochannels than at the surface. At the surface of ZnPW-NDC MOF⊃EDOT in Figure 6A, the monomers are significantly dispersed showing very little evidence of an eventual polymerisation at the surface, which implies that the nanochannels provide an efficient platform that brings the monomer to a sufficiently close distance in order to initiate polymerisation. On the other hand, the styrene monomers show some significant interaction at the surface of the ZnPW as observed in Figure 6B.

### 3.4 Contact Analysis

**FIGURE 7:**
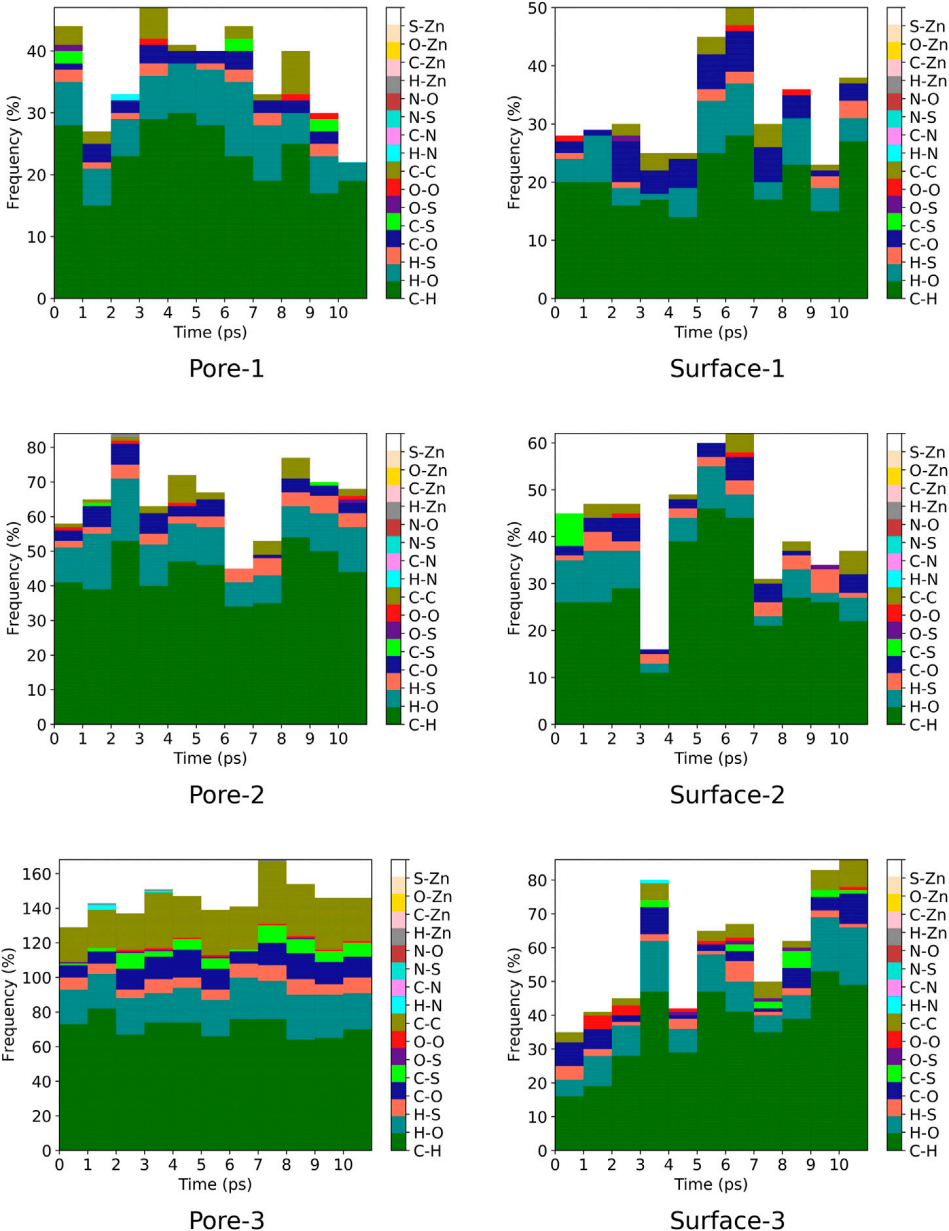
Frequency of ZnPW⊃EDOT intermolecular distances (r < 3.5 Å) for nanochannel and surface interactions. The colour gradient corresponds to specific interatomic interactions and each column correspond to a molecular dynamic trajectory snapshot. Interactions were computed at every 10 ps for systems representing nanochannel interactions and every 1 ps for systems representing surface interactions. The labels ”Pore” and ”Surface” correspond to nanochannel and surface interactions respectively and the numbers after the hyphen correspond to the number of monomer(s) present.

**FIGURE 8:**
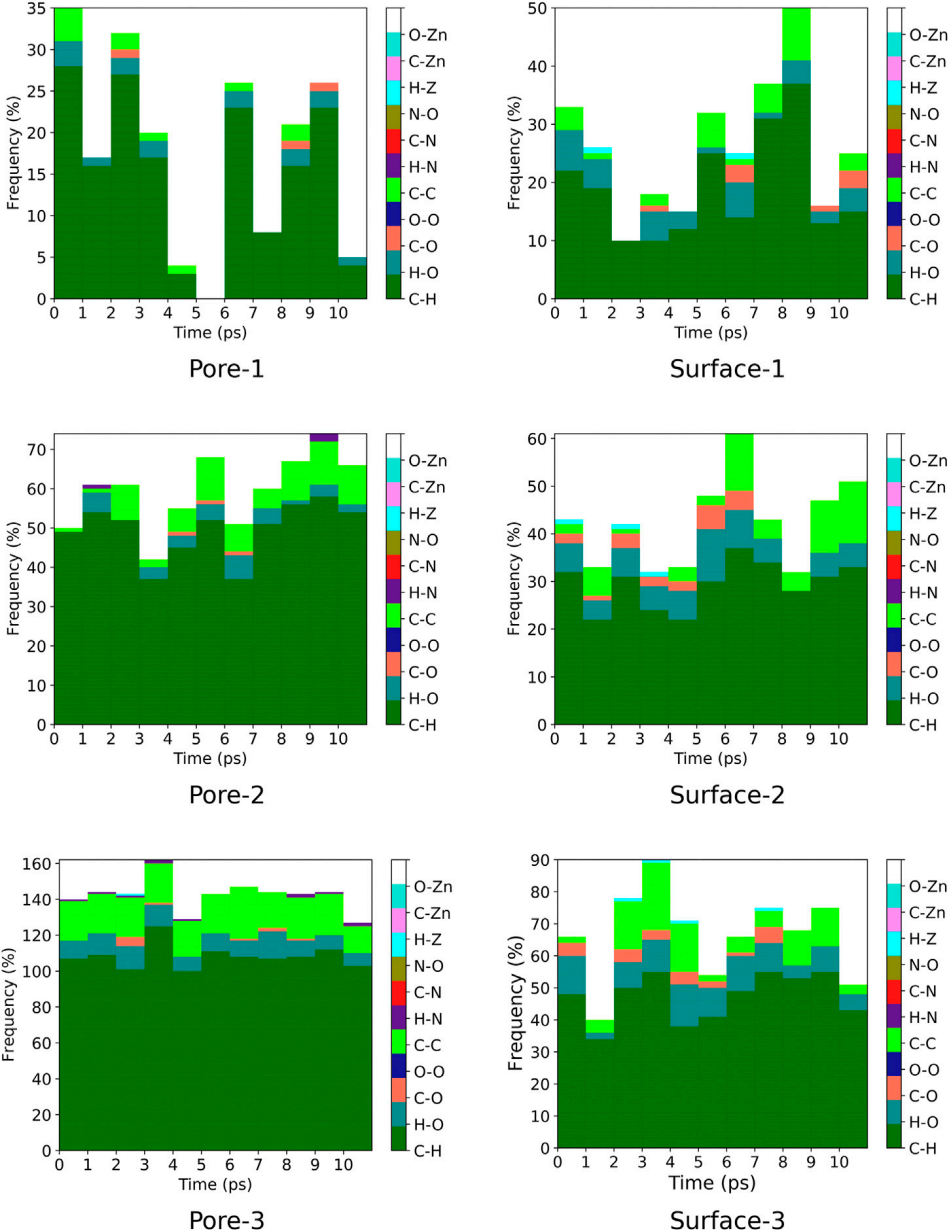
Frequency of ZnPW⊃styrene intermolecular distances (r < 3.5 Å) for nanochannel and surface interactions. The colour gradient corresponds to specific interatomic interactions and each column correspond to a molecular dynamic trajectory snapshot. Interactions were computed at every 10 ps for systems representing nanochannel interactions and every 1 ps for systems representing surface interactions. The labels ”Pore” and ”Surface” correspond to nanochannel and surface interactions respectively and the numbers after the hyphen correspond to the number of monomer(s) present.

A contact analysis was computed for MOF⊃monomer(s) intermolecular distances that are below 3.5 Å in each MD trajectory. The contact analysis was computed for every 1 ps for systems representing surface interactions meanwhile for systems representing nanochannel interactions, we computed intermolecular interactions for every 10 ps. The ZnPW-NDC MOF⊃monomer(s) contact analysis are plotted in Figures 7, 8 and the data for each of these interactions are found in the ESI^†^.

For the nanochannel and surface interaction of both monomers with the ZnPW-NDC MOF, the C-H, H-O and C-C are the most common interactions. These correspond to strong hydrogen-bond and hydrophobic interactions occurring between the monomers and the ZnPW. The number of these interactions are observed to increase with the concentration of monomers. In ZnPW-NDC MOF⊃EDOT systems, Figure 7, there is also a significant number of H-S and C-S interactions, which are known to be highly polarisable and can consequently increase the (ZnPW-NDC MOF)–EDOT electrostatic interactions (Rohwer et al., 2018).

## 4 Conclusion

In this study, we investigated the intermolecular interactions of two monomers, styrene and EDOT, with ZnPW-NDC MOF metal organic framework. It was observed for both monomers that the ZnPW-NDC MOF⊃monomer(s) intermolecular interactions are stronger in systems with higher monomer concentration for both surface and nanochannel interactions. The monomer–monomer interactions are observed to be strongest in the nanochannels, which is indicative of the nanochannel acting as an effective medium that brings monomers into close proximity therefore potentially optimising the polymerisation process. The ZnPW-NDC MOF⊃EDOT showed strong interactions at the surface of the ZnPW, which has been supported by a previous experimental study (Wang et al., 2017). This study reported an agglomeration of monomers at the surface of the ZnPW resulting in potential difficulties with fully infiltrating the monomer into the nanochannels.

## Data Availability

The original contributions presented in the study are included in the article/Supplementary Material. All computational data corresponding to detailed energetics, intermolecular interactions and MD trajectories can be freely downloaded from http://doi.org/10.5281/zenodo.4382475. Further inquiries can be directed to the corresponding author.
