# Supplementary material for: Role of Host-Guest Interaction in Understanding Polymerisation in Metal-Organic Frameworks
Source: Front Chem. 2021 Jul 21;9:716294. doi: 10.3389/fchem.2021.716294 (PMC8333864; doi:10.3389/fchem.2021.716294)
Supplement: Supplementary file 1 [file DataSheet1.PDF]

Role of host-guest interaction in understanding polymerisation in metal organic frameworks†

A.D. Dinga Wonanke,[a] Poppy Bennett,[a] Lewis Caldwell,[a] and Matthew A. Addicoat *[a]

Contents

1 Generation of the MOF⊃monomers complexes 1

2 Root-mean-square deviation (RMSD) time-series 2

3 Computation of dihedrals 5

4 Center-to-center distances 13

5 Monomer to monomer distance of the terminal atom for the first 20 ZnPW⊃monomer(s) energy minima 16

6 Detailed intermolecular distances of r $<$ 3.5 Å 18

1 Generation of the MOF⊃monomers complexes

100 MOF⊃monomers complexes were generated for every monomer with the Zn_2(1,4-ndc)$_2$(dabco), where 1,4-ndc = 1,4-naphthalenedicarboxylate and dabco = 1,4-diazabicyclo[2.2.2]octane, hereafter referred to as ZnPW. This was done by changing the position of the monomers within the nano-channels or at the surface of the ZnPW using a stochastic structure generation algorithm call Kick. [1] In this method, specified molecular fragments in different conformation are randomly translated in a chosen box providing chemically sensible translated entities. For this study, the ZnPW was constrained meanwhile the coordinates

of the monomer(s) were rotated by a random angle around each cartesian axis and the new coordinates translated into the box containing the containing the ZnPW. This was done for n =1, 2, 3 monomer(s) at different position in the nano-channels/surface of the ZnPW.

2 *Root-mean-square deviation (RMSD) time-series*

Time series RMSD plot for MOF$\supset$monomer(s) interactions computed from each molecular dynamic trajectory. The first snapshot for each trajectory was used as reference onto which each all other snapshots were superposed to minimise the resulting RMSD. The labels "Pore" and "Surface" correspond to nano-channel and surface interactions respectively and the numbers after the hyphen correspond to the number of monomer(s) present.

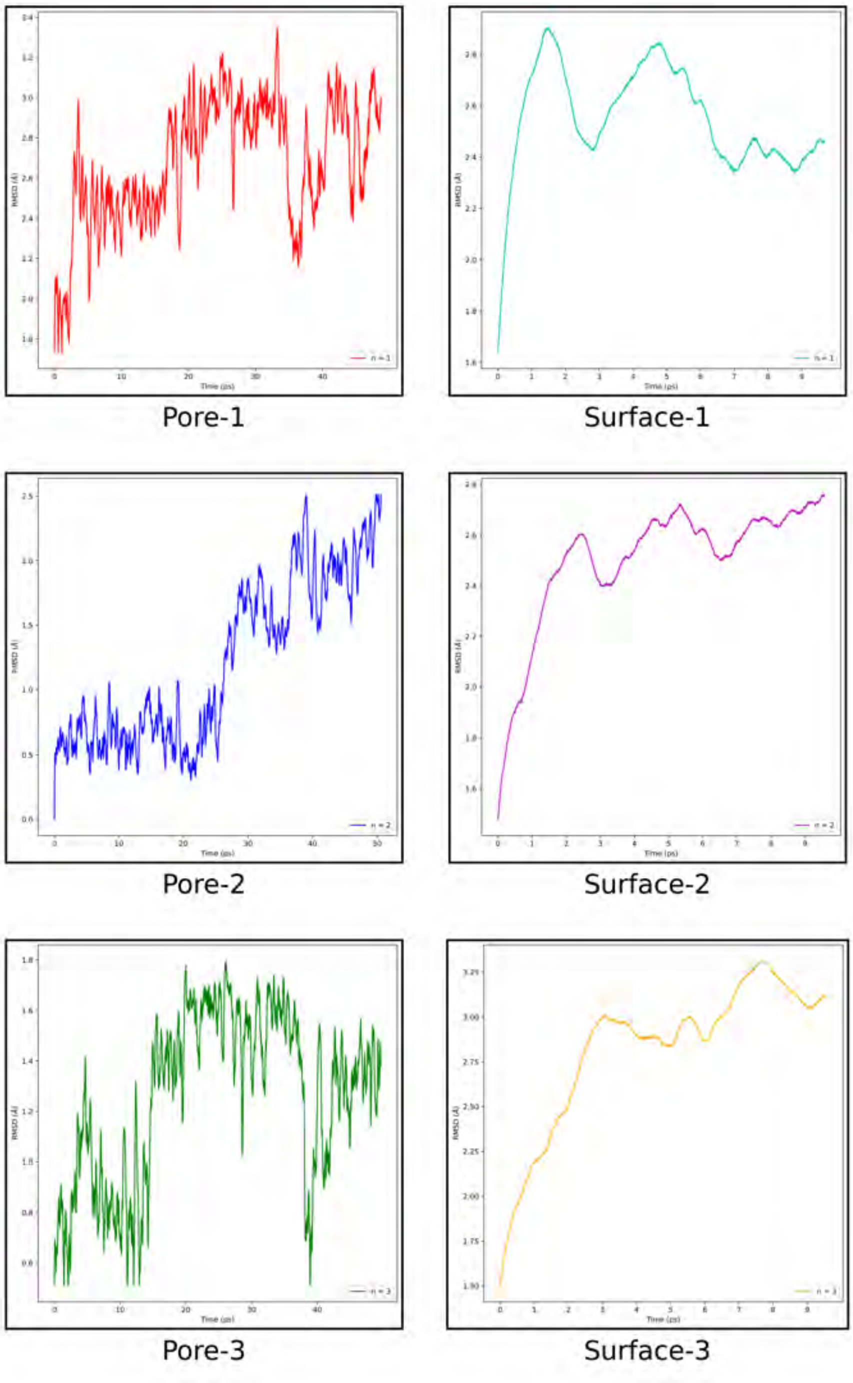

Fig. S1 ZnPW⊃EDOT time series RMSD for both nano-channel and surface interaction.

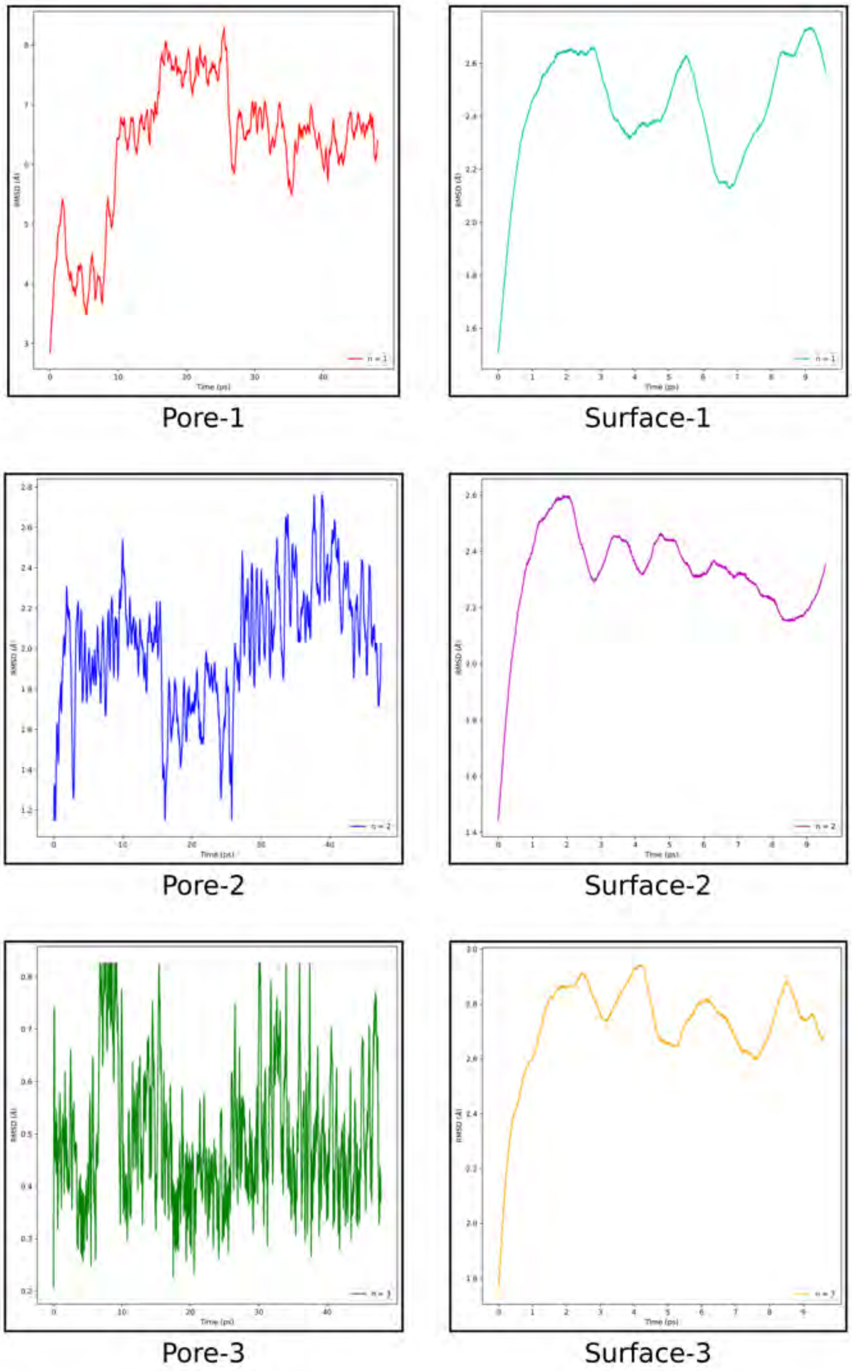

Fig. S2 ZnPW⊃styrene time series RMSD for both nano-channel and surface interaction.

3 Computation of dihedrals

To enable a detailed understanding of how the pore dimension fluctuates with time, we computed the dihedrals for the rotation of the ndc linkers along the plane in which the lie. Our procedure can easily be understood by looking at Fig. S3, we started by computing the vector V_m mid-point between the metal-metal atoms in the paddle wheel sbu. The distance between the atoms 3 at the mid point was computed to obtain the vertical length and the distance between atom 3 and the hydrogen atom bonded to the α-carbon adjacent to atom 1 was computed to obtain the horizontal length. For an orientation in which the metal-metal atom lies along the Z-coordinate as in Fig. S3, a new vector for atom 4 V_{dummy} computed as in equation 1. Overall, the strategy is similar to computing the angle of door, why using 3 corners of the door and one corner of the frame.

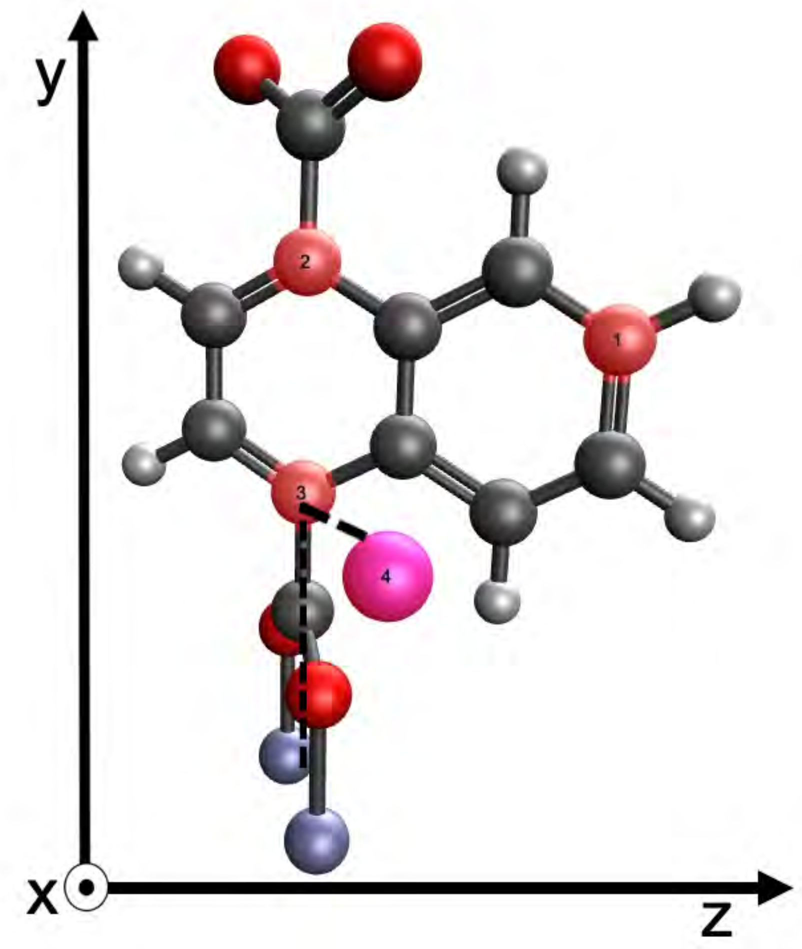

Fig. S3 Scaffold for computing dihedral angle for the rotation of ndc linker

$$\begin{aligned} V_{dummy}(x) &= V_m(x) + horizontallength \\ V_{dummy}(y) &= V_m(y) + verticallength \\ V_{dummy}(z) &= V_m(z) \end{aligned} \quad (1)$$

The dihedral was computed for all the positionally distinct ndc linkers in the cell. For instance, in a single unit cell, there are two positionally distinct ndc linkers coloured orange and blue as illustrated in Fig. S7.

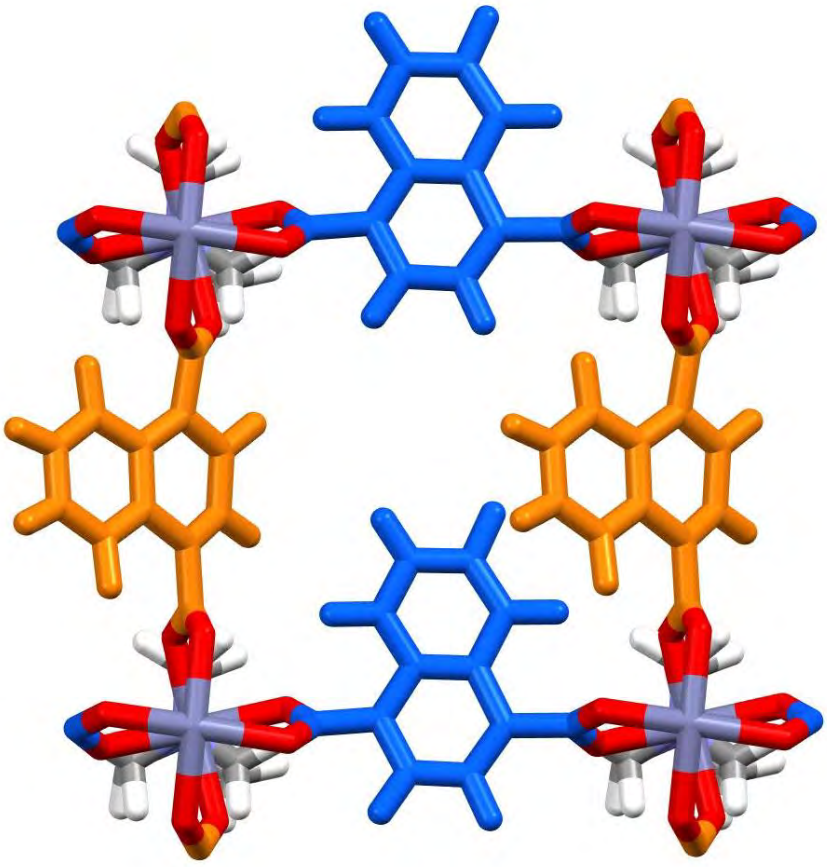

Fig. S4 Illustration of the distinct ndc linkers in a cell

The structures of the first and third conformational states for ZnPW⊃EDOT-2 and ZnPW⊃styrene-3, which were reference in the main text are illustrated in Fig. S5 and Fig. S6 respectively.

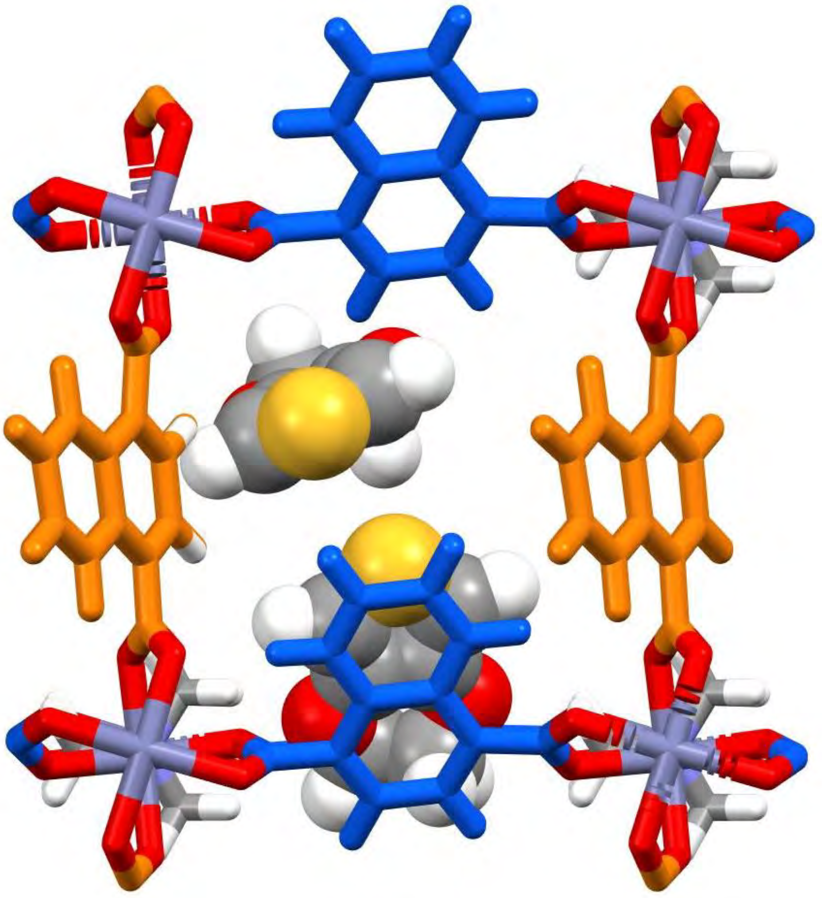

Fig. S5 Structural representation of ZnPW⊃EDOT-2 at 0 ps

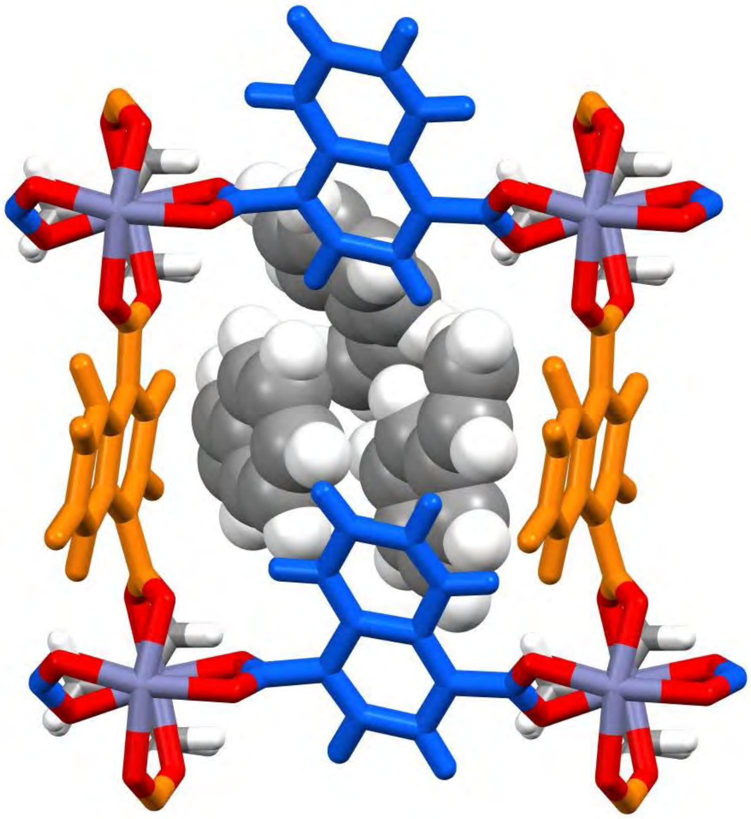

Fig. S6 Structural representation of ZnPW⊃styrene-3 at 5 ps

The dihedrals for all trajectories are illustrated in Fig. S8, **??**, Fig. S9.

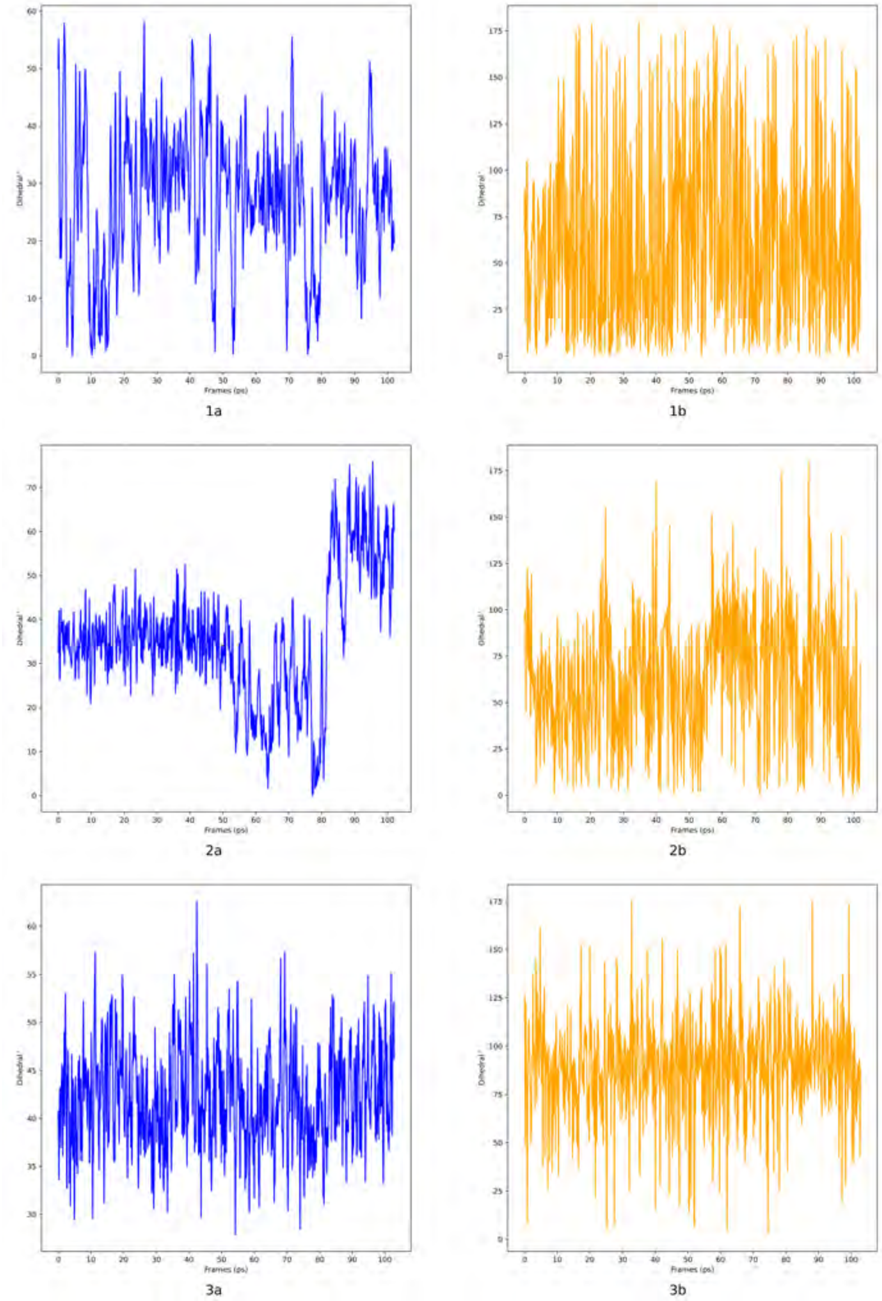

Fig. S7 Dihedral angles of the ndc linkers along for all the MD trajectories of the ZnPW⊃EDOT motif for nano-channel interactions. a and b correspond to the positionally distinct linkers and the numbers 1, 2 and 3 correspond to the number of monomers present.

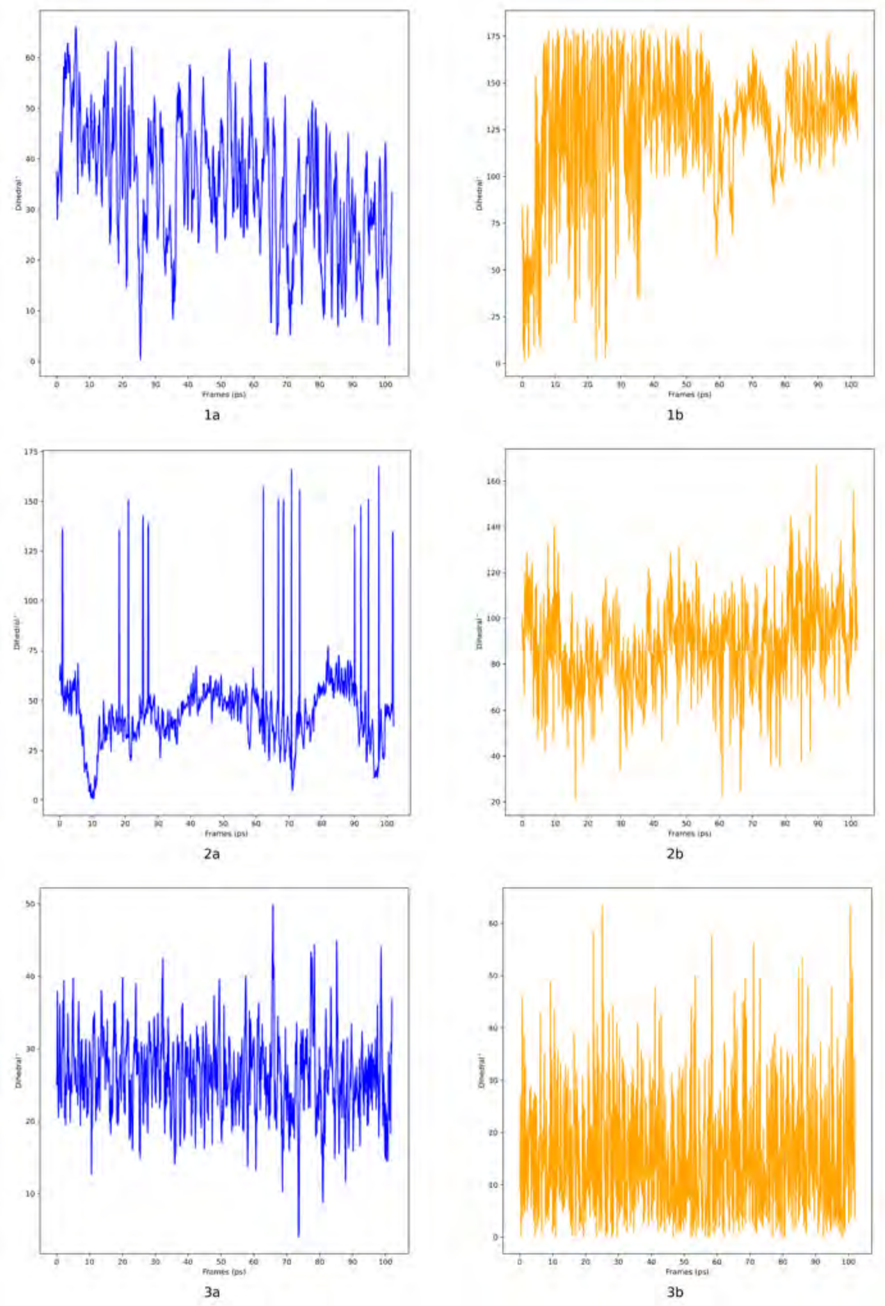

,

Fig. S8 Dihedral angles of the ndc linkers along for all the MD trajectories of the ZnPW⊃Styrene motif for nano-channel interactions. a and b correspond to the positionally distinct linkers and the numbers 1, 2 and 3 correspond to the number of monomers present.

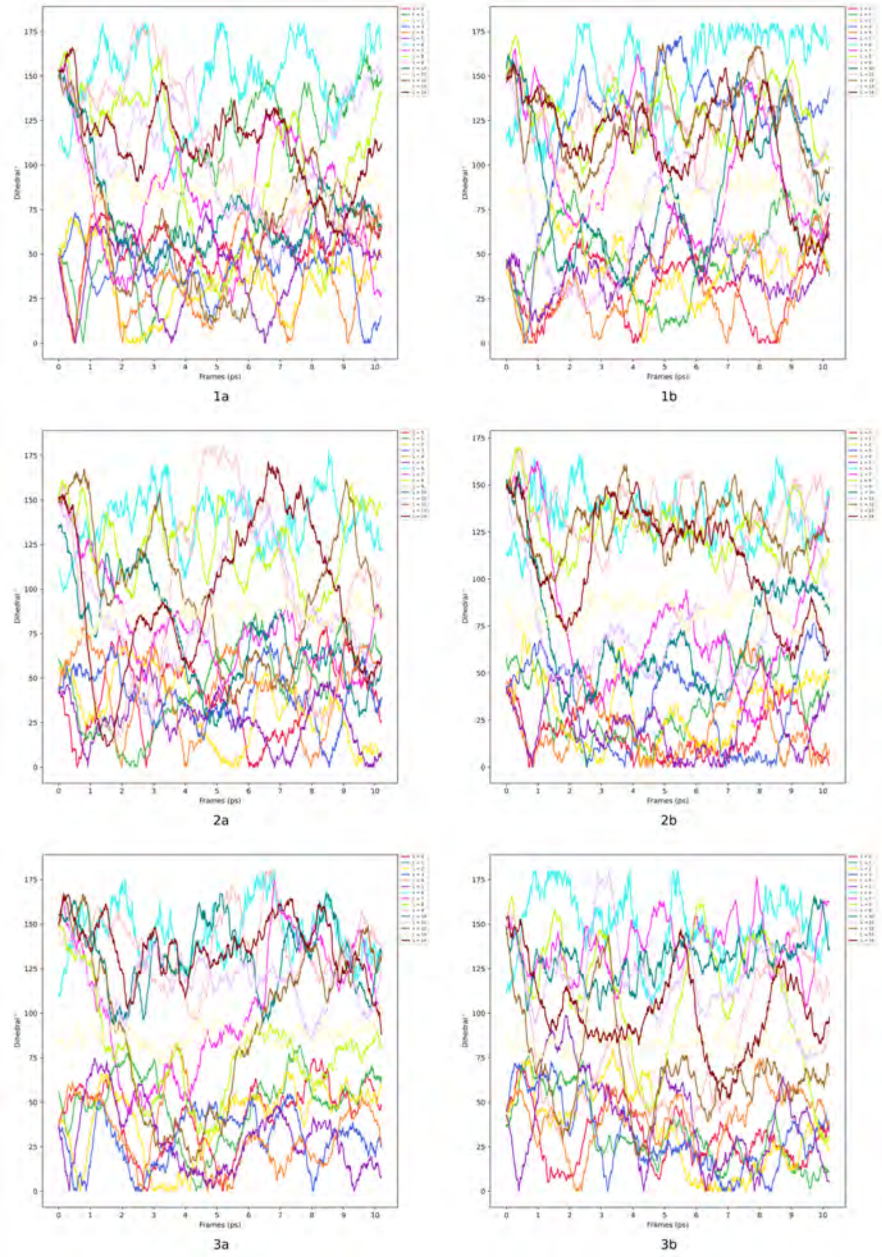

Fig. S9 Dihedral angles of the ndc linkers along for all the MD trajectories of the ZnPW$\supset$monomers motifs for surface interactions. a and b represent EDOT and styrene respectively. The numbers 1, 2 and 3 correspond to the number of monomers present.

4 *Center-to-center distances*

Distances from the center of each monomer to the center of the ZnPW were computed for all systems in order to visualise the correlative movement for each monomer with MOF across the time scale. The center-to-center distances were computed for every snapshot for systems representing surface interactions while this was computed for every 10th snapshots for stems representing nano-channel interactions.

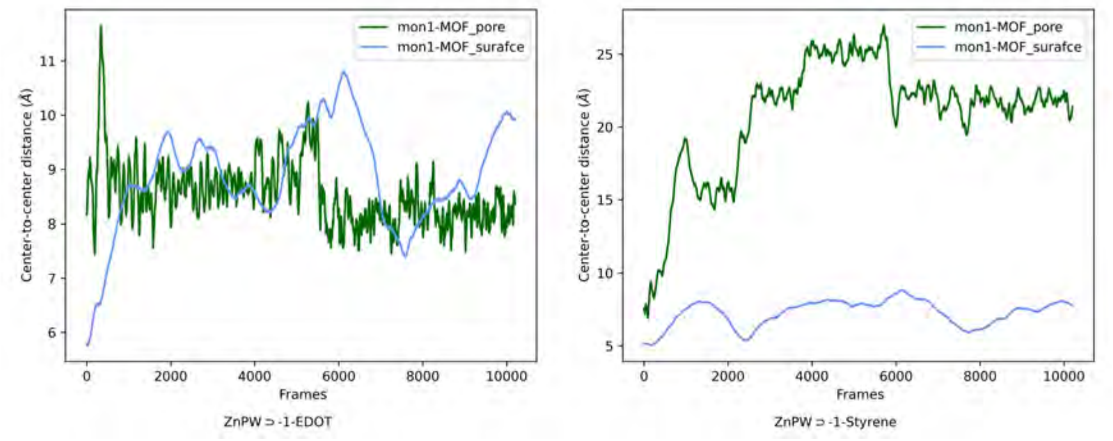

Fig. S10 Plot of the center-to-center distances of EDOT-ZnPW and styrene-ZnPW for systems containing only one monomer.

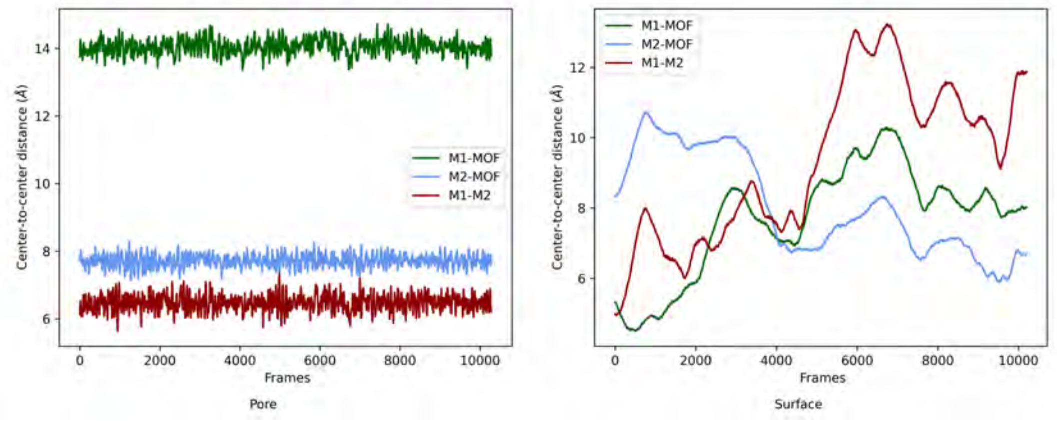

(a) Center-to-center distance of ZnPW⊃EDOT.

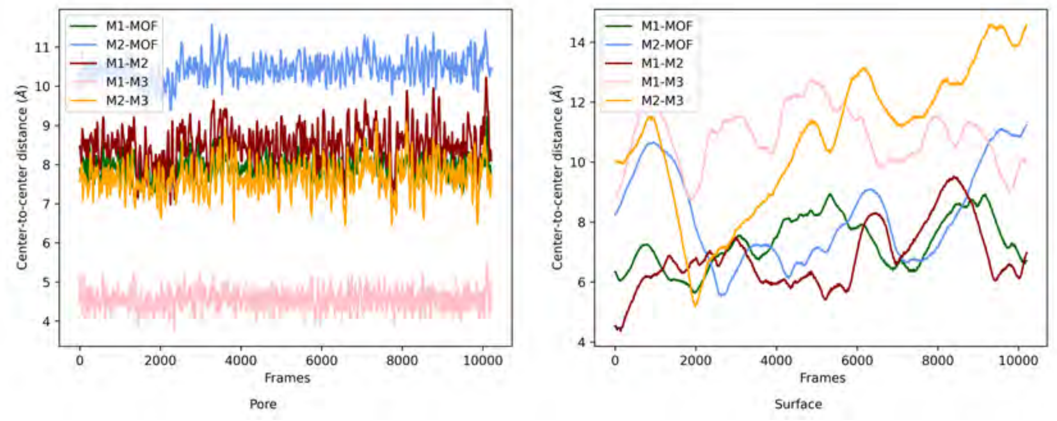

(b) Center-to-center distance of ZnPW⊃styrene .

Fig. S11 Plot of the center-to-center distances of ZnPW⊃monomer(s) for systems containing two monomers. M corresponds to the monomer(s) present in the trajectory and the number correspond to the specific monomer. The labels "Pore" and "Surface" correspond to nano-channel and surface interactions respectively.

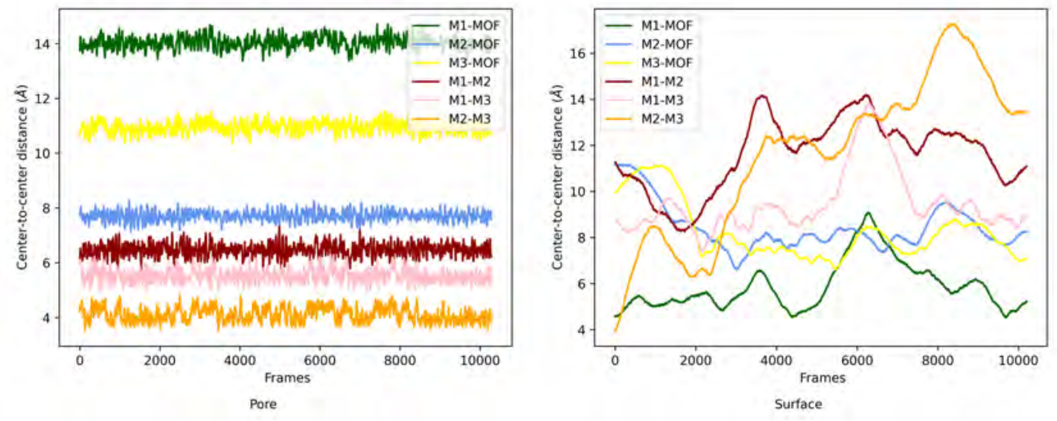

(a) Center-to-center distance of ZnPW⊃EDOT.

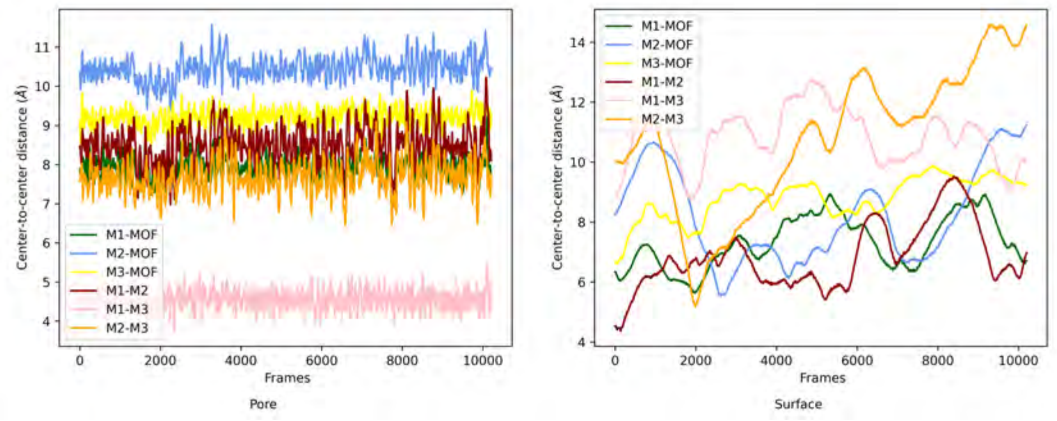

(b) Center-to-center distance of ZnPW⊃styrene.

Fig. S12 Plot of the center-to-center distances of ZnPW⊃monomer(s) for systems containing three monomers. M corresponds to the monomer(s) present in the trajectory and the number correspond to the specific monomer. The labels "Pore" and "Surface" correspond to nano-channel and surface interactions respectively.

5 *Monomer to monomer distance of the terminal atom for the first 20 ZnPW⊃monomer(s) energy minima*

Interatomic distances between the terminus of each monomer were computed for systems with 2 or more monomers involving nano-channel interactions. For EDOT, this correspond to S-S distances and for styrene, this correspond to C-C distances of the α olefins carbons. These distances are represented alongside the interaction energy of the ZnPW⊃monomer(s) composites in table Table S1 and Table S2 for ZnPW⊃EDOT and ZnPW⊃styrene composites respectively.

Table S1 Sulfur to sulfur distances (Å) of monomers, corresponding to susceptibility of monomer-monomer interaction at polymerisation site. The energy (kJmol^{-1}) correspond to the interaction of energy of the ZnPW⊃edot composites.

composites	2 monomers		3 monomers			
	Energy	B1-B2	Energy	B1-B2	B1-B3	B2-B3
1	-260.498	3.525	-422.299	5.806	3.839	7.157
2	-252.164	3.712	-410.377	5.746	4.321	3.725
3	-250.175	3.58	-399.181	5.25	7.321	6.645
4	-246.787	7.761	-391.323	5.747	4.91	6.707
5	-245.127	5.532	-389.968	6.416	4.275	4.475
6	-245.067	6.131	-388.683	4.372	6.593	7.452
7	-241.864	4.414	-386.558	3.457	5.601	5.527
8	-241.538	5.286	-385.282	3.45	5.882	5.582
9	-240.099	4.802	-384.812	5.371	5.129	4.262
10	-238.223	6.277	-383.187	5.203	4.39	7.892
11	-237.646	6.422	-382.866	5.183	6.305	5.468

Continues on next page

Table S1 – *Continued from previous page*

composites	2 monomers		3 monomers			
12	-235.425	3.583	-382.662	6.222	4.064	5.176
13	-235.155	5.79	-380.081	4.663	4.093	4.983
14	-234.584	6.074	-378.61	5.31	3.415	6.309
15	-233.219	6.315	-377.997	4.264	3.965	5.612
16	-232.866	4.309	-375.432	4.333	3.634	4.016
17	-232.69	3.355	-372.877	3.704	5.101	6.766
18	-231.854	4.749	-371.399	6.959	6.787	4.502
19	-231.595	5.111	-370.529	4.529	5.174	6.933
20	-231.239	4.136	-370.077	6.857	6.341	3.818

Table S2 Carbon to carbon distances (Å) of α-carbons of the olefins groups in the styrene monomers. This corresponding to susceptibility of monomer-monomer interaction at polymerisation site. The energy (kJmol^{-1}) correspond to the interaction of energy of the ZnPW⊃styrene composites

composites	2 monomers		3 monomers			
Composites	Energy	B1-B2	Energy	B1-B2	B1-B3	B2-B3
1	-228.964	4.37	-362.637	4.205	6.438	3.822
2	-220.423	4.666	-359.169	4.935	5.912	3.467
3	-218.414	6.182	-357.029	4.359	3.546	6.398
4	-218.261	6.842	-355.408	5.282	5.469	6.866
5	-218.242	4.062	-351.18	4.001	4.32	6.587
6	-217.071	5.17	-346.559	4.681	7.101	3.381

Continues on next page

Table S2 – *Continued from previous page*

composites	2 monomers		3 monomers			
7	-216.817	5.011	-346.428	6.406	3.524	5.33
8	-216.695	6.494	-344.578	5.773	3.699	6.557
9	-215.119	3.658	-344.385	3.771	5.026	3.434
10	-214.605	5.088	-342.569	3.923	4.246	4.008
11	-214.268	3.657	-341.756	3.791	3.543	6.003
12	-212.942	4.168	-339.997	4.526	3.52	4.547
13	-212.777	4.496	-339.523	5.296	6.425	7.198
14	-212.043	6.881	-338.922	5.338	7.825	5.283
15	-211.342	7.072	-338.456	3.759	6.004	5.526
16	-211.281	3.772	-338.343	6.47	6.896	5.301
17	-210.714	5.909	-337.153	7.703	3.956	6.651
18	-210.014	4.512	-336.724	5.187	5.914	4.254
19	-209.991	5.635	-336.516	5.514	7.486	3.569
20	-209.944	5.947	-335.894	5.354	4.453	7.992

6 *Detailed intermolecular distances of $r < 3.5$ Å*

Detailed ZnPW⊃monomer(s) intermolecular distances that are below 3.5 Å in each MD trajectory. This distances were computed for every 1000 frames for systems representing surface interactions and 10000 frames for systems representing nano-channel interactions.

Table S3 Intermolecular distances (r < 3.5 Å) for pore ZnPW⊃-1-EDOT interactions computed for every 10000 molecular dynamic frames.

C-H	H-O	H-S	C-O	C-S	O-S	O-O	C-C	H-N	C-N	N-S
Frame number : 0										
3.427	2.96	2.549	3.5	3.421	2.861	-	3.491	-	-	-
3.087	3.033	2.89	-	3.425	-	-	3.456	-	-	-
3.439	3.402	-	-	-	-	-	3.411	-	-	-
3.325	2.612	-	-	-	-	-	-	-	-	-
3.219	3.07	-	-	-	-	-	-	-	-	-
3.283	3.496	-	-	-	-	-	-	-	-	-
3.411	3.157	-	-	-	-	-	-	-	-	-
3.322	-	-	-	-	-	-	-	-	-	-
3.414	-	-	-	-	-	-	-	-	-	-
3.145	-	-	-	-	-	-	-	-	-	-
3.409	-	-	-	-	-	-	-	-	-	-
3.345	-	-	-	-	-	-	-	-	-	-
3.431	-	-	-	-	-	-	-	-	-	-
3.437	-	-	-	-	-	-	-	-	-	-
3.495	-	-	-	-	-	-	-	-	-	-
3.195	-	-	-	-	-	-	-	-	-	-
2.809	-	-	-	-	-	-	-	-	-	-
3.024	-	-	-	-	-	-	-	-	-	-
2.637	-	-	-	-	-	-	-	-	-	-
3.164	-	-	-	-	-	-	-	-	-	-
2.915	-	-	-	-	-	-	-	-	-	-

Continues on next page

Table S3 – *Continued from previous page*

C-H	H-O	H-S	C-O	C-S	O-S	O-O	C-C	H-N	C-N	N-S
3.279	-	-	-	-	-	-	-	-	-	-
3.047	-	-	-	-	-	-	-	-	-	-
2.87	-	-	-	-	-	-	-	-	-	-
3.05	-	-	-	-	-	-	-	-	-	-
3.328	-	-	-	-	-	-	-	-	-	-
3.287	-	-	-	-	-	-	-	-	-	-
3.14	-	-	-	-	-	-	-	-	-	-
Frame number : 10000										
2.918	2.437	3.456	3.174	-	-	-	3.475	-	-	-
2.72	2.349	-	3.176	-	-	-	3.467	-	-	-
3.498	2.923	-	3.306	-	-	-	-	-	-	-
3.362	3.342	-	-	-	-	-	-	-	-	-
3.448	3.433	-	-	-	-	-	-	-	-	-
2.802	3.063	-	-	-	-	-	-	-	-	-
3.113	-	-	-	-	-	-	-	-	-	-
3.42	-	-	-	-	-	-	-	-	-	-
2.787	-	-	-	-	-	-	-	-	-	-
2.825	-	-	-	-	-	-	-	-	-	-
3.168	-	-	-	-	-	-	-	-	-	-
3.203	-	-	-	-	-	-	-	-	-	-
3.144	-	-	-	-	-	-	-	-	-	-
3.077	-	-	-	-	-	-	-	-	-	-
2.812	-	-	-	-	-	-	-	-	-	-
Frame number : 20000										

Continues on next page

Table S3 – *Continued from previous page*

C-H	H-O	H-S	C-O	C-S	O-S	O-O	C-C	H-N	C-N	N-S
3.259	3.201	3.036	3.338	-	-	-	-	3.205	-	-
3.265	3.263	-	3.252	-	-	-	-	-	-	-
2.859	3.174	-	-	-	-	-	-	-	-	-
3.125	2.407	-	-	-	-	-	-	-	-	-
2.854	3.356	-	-	-	-	-	-	-	-	-
3.128	3.247	-	-	-	-	-	-	-	-	-
3.156	-	-	-	-	-	-	-	-	-	-
3.344	-	-	-	-	-	-	-	-	-	-
3.434	-	-	-	-	-	-	-	-	-	-
3.362	-	-	-	-	-	-	-	-	-	-
2.771	-	-	-	-	-	-	-	-	-	-
3.297	-	-	-	-	-	-	-	-	-	-
2.97	-	-	-	-	-	-	-	-	-	-
3.458	-	-	-	-	-	-	-	-	-	-
3.235	-	-	-	-	-	-	-	-	-	-
3.382	-	-	-	-	-	-	-	-	-	-
3.431	-	-	-	-	-	-	-	-	-	-
3.394	-	-	-	-	-	-	-	-	-	-
3.354	-	-	-	-	-	-	-	-	-	-
3.187	-	-	-	-	-	-	-	-	-	-
3.359	-	-	-	-	-	-	-	-	-	-
3.43	-	-	-	-	-	-	-	-	-	-
3.138	-	-	-	-	-	-	-	-	-	-
Frame number : 30000										

Continues on next page

Table S3 – *Continued from previous page*

C-H	H-O	H-S	C-O	C-S	O-S	O-O	C-C	H-N	C-N	N-S
3.329	2.385	3.053	3.349	-	-	3.37	3.394	-	-	-
3.31	2.99	3.372	3.413	-	-	-	3.263	-	-	-
3.385	2.869	-	3.457	-	-	-	3.495	-	-	-
2.899	2.657	-	-	-	-	-	3.316	-	-	-
3.236	3.262	-	-	-	-	-	3.488	-	-	-
3.254	2.593	-	-	-	-	-	-	-	-	-
2.987	3.289	-	-	-	-	-	-	-	-	-
3.087	-	-	-	-	-	-	-	-	-	-
3.325	-	-	-	-	-	-	-	-	-	-
3.102	-	-	-	-	-	-	-	-	-	-
2.892	-	-	-	-	-	-	-	-	-	-
3.02	-	-	-	-	-	-	-	-	-	-
2.53	-	-	-	-	-	-	-	-	-	-
3.172	-	-	-	-	-	-	-	-	-	-
2.756	-	-	-	-	-	-	-	-	-	-
2.921	-	-	-	-	-	-	-	-	-	-
2.921	-	-	-	-	-	-	-	-	-	-
3.272	-	-	-	-	-	-	-	-	-	-
2.781	-	-	-	-	-	-	-	-	-	-
3.24	-	-	-	-	-	-	-	-	-	-
3.004	-	-	-	-	-	-	-	-	-	-
2.961	-	-	-	-	-	-	-	-	-	-
2.798	-	-	-	-	-	-	-	-	-	-
2.67	-	-	-	-	-	-	-	-	-	-

Continues on next page

Table S3 – *Continued from previous page*

C-H	H-O	H-S	C-O	C-S	O-S	O-O	C-C	H-N	C-N	N-S
2.807	-	-	-	-	-	-	-	-	-	-
3.055	-	-	-	-	-	-	-	-	-	-
3.074	-	-	-	-	-	-	-	-	-	-
3.436	-	-	-	-	-	-	-	-	-	-
3.016	-	-	-	-	-	-	-	-	-	-
Frame number : 40000										
3.049	2.85	-	2.912	-	-	-	3.33	-	-	-
3.151	3.354	-	3.138	-	-	-	-	-	-	-
3.138	2.993	-	-	-	-	-	-	-	-	-
3.038	3.278	-	-	-	-	-	-	-	-	-
3.152	2.53	-	-	-	-	-	-	-	-	-
3.463	2.985	-	-	-	-	-	-	-	-	-
3.248	2.771	-	-	-	-	-	-	-	-	-
3.318	2.806	-	-	-	-	-	-	-	-	-
3.21	-	-	-	-	-	-	-	-	-	-
3.098	-	-	-	-	-	-	-	-	-	-
3.23	-	-	-	-	-	-	-	-	-	-
3.415	-	-	-	-	-	-	-	-	-	-
2.753	-	-	-	-	-	-	-	-	-	-
3.381	-	-	-	-	-	-	-	-	-	-
2.757	-	-	-	-	-	-	-	-	-	-
3.217	-	-	-	-	-	-	-	-	-	-
3.47	-	-	-	-	-	-	-	-	-	-
3.163	-	-	-	-	-	-	-	-	-	-

Continues on next page

Table S3 – *Continued from previous page*

C-H	H-O	H-S	C-O	C-S	O-S	O-O	C-C	H-N	C-N	N-S
3.367	-	-	-	-	-	-	-	-	-	-
3.435	-	-	-	-	-	-	-	-	-	-
3.351	-	-	-	-	-	-	-	-	-	-
3.338	-	-	-	-	-	-	-	-	-	-
3.342	-	-	-	-	-	-	-	-	-	-
2.958	-	-	-	-	-	-	-	-	-	-
3.388	-	-	-	-	-	-	-	-	-	-
2.639	-	-	-	-	-	-	-	-	-	-
2.705	-	-	-	-	-	-	-	-	-	-
3.493	-	-	-	-	-	-	-	-	-	-
3.222	-	-	-	-	-	-	-	-	-	-
3.356	-	-	-	-	-	-	-	-	-	-
Frame number : 50000										
3.374	3.478	2.999	3.261	-	-	-	-	-	-	-
3.145	3.239	-	3.081	-	-	-	-	-	-	-
3.465	2.998	-	-	-	-	-	-	-	-	-
3.417	2.548	-	-	-	-	-	-	-	-	-
3.16	2.813	-	-	-	-	-	-	-	-	-
2.955	2.879	-	-	-	-	-	-	-	-	-
3.275	2.569	-	-	-	-	-	-	-	-	-
3.116	2.82	-	-	-	-	-	-	-	-	-
3.299	3.45	-	-	-	-	-	-	-	-	-
2.921	-	-	-	-	-	-	-	-	-	-
3.052	-	-	-	-	-	-	-	-	-	-

Continues on next page

Table S3 – *Continued from previous page*

C-H	H-O	H-S	C-O	C-S	O-S	O-O	C-C	H-N	C-N	N-S
3.415	-	-	-	-	-	-	-	-	-	-
3.013	-	-	-	-	-	-	-	-	-	-
2.824	-	-	-	-	-	-	-	-	-	-
2.933	-	-	-	-	-	-	-	-	-	-
3.463	-	-	-	-	-	-	-	-	-	-
2.87	-	-	-	-	-	-	-	-	-	-
3.354	-	-	-	-	-	-	-	-	-	-
3.369	-	-	-	-	-	-	-	-	-	-
3.026	-	-	-	-	-	-	-	-	-	-
3.451	-	-	-	-	-	-	-	-	-	-
3.221	-	-	-	-	-	-	-	-	-	-
3.232	-	-	-	-	-	-	-	-	-	-
3.289	-	-	-	-	-	-	-	-	-	-
3.173	-	-	-	-	-	-	-	-	-	-
2.935	-	-	-	-	-	-	-	-	-	-
3.149	-	-	-	-	-	-	-	-	-	-
3.324	-	-	-	-	-	-	-	-	-	-
Frame number : 60000										
3.017	2.853	3.451	3.078	3.499	-	-	3.253	-	-	-
3.467	2.95	3.367	3.083	3.467	-	-	3.067	-	-	-
3.312	2.943	-	3.285	-	-	-	-	-	-	-
3.059	2.815	-	-	-	-	-	-	-	-	-
2.872	2.258	-	-	-	-	-	-	-	-	-
3.312	3.033	-	-	-	-	-	-	-	-	-

Continues on next page

Table S3 – *Continued from previous page*

C-H	H-O	H-S	C-O	C-S	O-S	O-O	C-C	H-N	C-N	N-S
2.963	3.261	-	-	-	-	-	-	-	-	-
3.215	3.379	-	-	-	-	-	-	-	-	-
3.417	2.861	-	-	-	-	-	-	-	-	-
3.195	3.226	-	-	-	-	-	-	-	-	-
2.995	3.393	-	-	-	-	-	-	-	-	-
3.36	3.279	-	-	-	-	-	-	-	-	-
3.085	-	-	-	-	-	-	-	-	-	-
3.066	-	-	-	-	-	-	-	-	-	-
3.457	-	-	-	-	-	-	-	-	-	-
3.354	-	-	-	-	-	-	-	-	-	-
3.409	-	-	-	-	-	-	-	-	-	-
3.351	-	-	-	-	-	-	-	-	-	-
3.408	-	-	-	-	-	-	-	-	-	-
2.977	-	-	-	-	-	-	-	-	-	-
3.308	-	-	-	-	-	-	-	-	-	-
2.852	-	-	-	-	-	-	-	-	-	-
3.304	-	-	-	-	-	-	-	-	-	-
Frame number : 70000										
3.281	2.826	3.441	3.459	-	-	-	3.415	-	-	-
3.03	2.333	3.342	3.197	-	-	-	-	-	-	-
3.225	3.477	-	-	-	-	-	-	-	-	-
3.215	3.158	-	-	-	-	-	-	-	-	-
3.308	3.158	-	-	-	-	-	-	-	-	-
3.216	3.238	-	-	-	-	-	-	-	-	-

Continues on next page

Table S3 – *Continued from previous page*

C-H	H-O	H-S	C-O	C-S	O-S	O-O	C-C	H-N	C-N	N-S
3.392	3.405	-	-	-	-	-	-	-	-	-
3.497	2.793	-	-	-	-	-	-	-	-	-
2.888	3.016	-	-	-	-	-	-	-	-	-
2.811	-	-	-	-	-	-	-	-	-	-
2.978	-	-	-	-	-	-	-	-	-	-
3.211	-	-	-	-	-	-	-	-	-	-
3.273	-	-	-	-	-	-	-	-	-	-
3.052	-	-	-	-	-	-	-	-	-	-
3.447	-	-	-	-	-	-	-	-	-	-
3.148	-	-	-	-	-	-	-	-	-	-
3.244	-	-	-	-	-	-	-	-	-	-
2.74	-	-	-	-	-	-	-	-	-	-
2.686	-	-	-	-	-	-	-	-	-	-
Frame number : 80000										
3.068	2.913	-	3.377	-	-	3.42	3.486	-	-	-
2.876	2.762	-	3.451	-	-	-	3.272	-	-	-
3.159	2.521	-	-	-	-	-	3.371	-	-	-
3.304	2.412	-	-	-	-	-	3.232	-	-	-
3.366	3.411	-	-	-	-	-	3.347	-	-	-
2.71	-	-	-	-	-	-	3.33	-	-	-
3.296	-	-	-	-	-	-	3.409	-	-	-
2.875	-	-	-	-	-	-	-	-	-	-
2.856	-	-	-	-	-	-	-	-	-	-
2.944	-	-	-	-	-	-	-	-	-	-

Continues on next page

Table S3 – *Continued from previous page*

C-H	H-O	H-S	C-O	C-S	O-S	O-O	C-C	H-N	C-N	N-S
3.241	-	-	-	-	-	-	-	-	-	-
3.244	-	-	-	-	-	-	-	-	-	-
2.742	-	-	-	-	-	-	-	-	-	-
3.021	-	-	-	-	-	-	-	-	-	-
2.93	-	-	-	-	-	-	-	-	-	-
2.574	-	-	-	-	-	-	-	-	-	-
2.279	-	-	-	-	-	-	-	-	-	-
2.362	-	-	-	-	-	-	-	-	-	-
3.454	-	-	-	-	-	-	-	-	-	-
3.48	-	-	-	-	-	-	-	-	-	-
3.482	-	-	-	-	-	-	-	-	-	-
3.208	-	-	-	-	-	-	-	-	-	-
3.276	-	-	-	-	-	-	-	-	-	-
3.45	-	-	-	-	-	-	-	-	-	-
3.309	-	-	-	-	-	-	-	-	-	-
Frame number : 90000										
3.43	3.488	3.276	3.208	3.297	-	3.122	-	-	-	-
3.033	3.184	3.42	3.379	3.273	-	-	-	-	-	-
3.107	2.226	-	-	-	-	-	-	-	-	-
3.226	2.765	-	-	-	-	-	-	-	-	-
3.41	2.859	-	-	-	-	-	-	-	-	-
3.351	3.475	-	-	-	-	-	-	-	-	-
3.373	-	-	-	-	-	-	-	-	-	-
2.761	-	-	-	-	-	-	-	-	-	-

Continues on next page

Table S3 – *Continued from previous page*

C-H	H-O	H-S	C-O	C-S	O-S	O-O	C-C	H-N	C-N	N-S
3.474	-	-	-	-	-	-	-	-	-	-
3.003	-	-	-	-	-	-	-	-	-	-
3.426	-	-	-	-	-	-	-	-	-	-
3.353	-	-	-	-	-	-	-	-	-	-
2.964	-	-	-	-	-	-	-	-	-	-
3.433	-	-	-	-	-	-	-	-	-	-
3.48	-	-	-	-	-	-	-	-	-	-
3.271	-	-	-	-	-	-	-	-	-	-
3.443	-	-	-	-	-	-	-	-	-	-
Frame number : 100000										
3.046	2.812	-	-	-	-	-	-	-	-	-
3.226	2.966	-	-	-	-	-	-	-	-	-
3.023	3.081	-	-	-	-	-	-	-	-	-
3.367	-	-	-	-	-	-	-	-	-	-
3.069	-	-	-	-	-	-	-	-	-	-
3.241	-	-	-	-	-	-	-	-	-	-
3.15	-	-	-	-	-	-	-	-	-	-
2.762	-	-	-	-	-	-	-	-	-	-
3.293	-	-	-	-	-	-	-	-	-	-
3.362	-	-	-	-	-	-	-	-	-	-
3.311	-	-	-	-	-	-	-	-	-	-
3.225	-	-	-	-	-	-	-	-	-	-
2.854	-	-	-	-	-	-	-	-	-	-
2.624	-	-	-	-	-	-	-	-	-	-

Continues on next page

Table S3 – *Continued from previous page*

C-H	H-O	H-S	C-O	C-S	O-S	O-O	C-C	H-N	C-N	N-S
2.79	-	-	-	-	-	-	-	-	-	-
3.462	-	-	-	-	-	-	-	-	-	-
3.261	-	-	-	-	-	-	-	-	-	-
3.447	-	-	-	-	-	-	-	-	-	-
3.389	-	-	-	-	-	-	-	-	-	-

Table S4 Intermolecular distances (r < 3.5 Å) for pore ZnPW⊃-2-EDOT interactions computed for every 10000 molecular dynamic frames.

C-H	H-O	H-S	C-O	C-S	O-S	O-O	C-C	H-N	C-N	N-S
Frame number : 0										
3.162	2.908	3.338	3.232	-	-	3.29	3.421	-	-	-
3.455	2.252	3.016	3.415	-	-	-	-	-	-	-
3.056	2.917	-	3.331	-	-	-	-	-	-	-
3.474	3.109	-	-	-	-	-	-	-	-	-
3.487	2.99	-	-	-	-	-	-	-	-	-
3.465	2.692	-	-	-	-	-	-	-	-	-
3.339	3.304	-	-	-	-	-	-	-	-	-
3.367	3.241	-	-	-	-	-	-	-	-	-
3.379	3.089	-	-	-	-	-	-	-	-	-
3.065	2.251	-	-	-	-	-	-	-	-	-
3.413	-	-	-	-	-	-	-	-	-	-
3.494	-	-	-	-	-	-	-	-	-	-

Continues on next page

Table S4 – *Continued from previous page*

C-H	H-O	H-S	C-O	C-S	O-S	O-O	C-C	H-N	C-N	N-S
3.352	-	-	-	-	-	-	-	-	-	-
2.824	-	-	-	-	-	-	-	-	-	-
3.227	-	-	-	-	-	-	-	-	-	-
3.473	-	-	-	-	-	-	-	-	-	-
3.318	-	-	-	-	-	-	-	-	-	-
2.863	-	-	-	-	-	-	-	-	-	-
2.603	-	-	-	-	-	-	-	-	-	-
3.472	-	-	-	-	-	-	-	-	-	-
3.18	-	-	-	-	-	-	-	-	-	-
3.054	-	-	-	-	-	-	-	-	-	-
3.313	-	-	-	-	-	-	-	-	-	-
3.363	-	-	-	-	-	-	-	-	-	-
3.297	-	-	-	-	-	-	-	-	-	-
3.28	-	-	-	-	-	-	-	-	-	-
3.365	-	-	-	-	-	-	-	-	-	-
3.139	-	-	-	-	-	-	-	-	-	-
3.48	-	-	-	-	-	-	-	-	-	-
3.3	-	-	-	-	-	-	-	-	-	-
3.431	-	-	-	-	-	-	-	-	-	-
2.959	-	-	-	-	-	-	-	-	-	-
3.292	-	-	-	-	-	-	-	-	-	-
3.32	-	-	-	-	-	-	-	-	-	-
3.382	-	-	-	-	-	-	-	-	-	-
3.153	-	-	-	-	-	-	-	-	-	-

Continues on next page

Table S4 – *Continued from previous page*

C-H	H-O	H-S	C-O	C-S	O-S	O-O	C-C	H-N	C-N	N-S
2.979	-	-	-	-	-	-	-	-	-	-
3.037	-	-	-	-	-	-	-	-	-	-
2.791	-	-	-	-	-	-	-	-	-	-
3.153	-	-	-	-	-	-	-	-	-	-
3.226	-	-	-	-	-	-	-	-	-	-
Frame number : 10000										
3.37	2.165	2.777	3.192	3.441	-	-	3.45	-	-	-
3.147	3.093	2.638	3.273	-	-	-	-	-	-	-
2.699	3.437	-	3.442	-	-	-	-	-	-	-
2.685	3.256	-	3.276	-	-	-	-	-	-	-
3.38	3.315	-	3.397	-	-	-	-	-	-	-
3.043	3.423	-	3.387	-	-	-	-	-	-	-
2.999	2.455	-	-	-	-	-	-	-	-	-
2.963	3.449	-	-	-	-	-	-	-	-	-
3.079	3.257	-	-	-	-	-	-	-	-	-
2.993	3.313	-	-	-	-	-	-	-	-	-
3.052	3.458	-	-	-	-	-	-	-	-	-
3.261	3.05	-	-	-	-	-	-	-	-	-
3.466	2.66	-	-	-	-	-	-	-	-	-
2.998	2.688	-	-	-	-	-	-	-	-	-
3.375	2.85	-	-	-	-	-	-	-	-	-
3.338	3.237	-	-	-	-	-	-	-	-	-
2.811	-	-	-	-	-	-	-	-	-	-
2.63	-	-	-	-	-	-	-	-	-	-

Continues on next page

Table S4 – *Continued from previous page*

C-H	H-O	H-S	C-O	C-S	O-S	O-O	C-C	H-N	C-N	N-S
3.236	-	-	-	-	-	-	-	-	-	-
3.388	-	-	-	-	-	-	-	-	-	-
3.279	-	-	-	-	-	-	-	-	-	-
3.372	-	-	-	-	-	-	-	-	-	-
3.488	-	-	-	-	-	-	-	-	-	-
2.771	-	-	-	-	-	-	-	-	-	-
3.182	-	-	-	-	-	-	-	-	-	-
3.453	-	-	-	-	-	-	-	-	-	-
3.081	-	-	-	-	-	-	-	-	-	-
3.074	-	-	-	-	-	-	-	-	-	-
3.299	-	-	-	-	-	-	-	-	-	-
3.112	-	-	-	-	-	-	-	-	-	-
2.917	-	-	-	-	-	-	-	-	-	-
3.262	-	-	-	-	-	-	-	-	-	-
3.471	-	-	-	-	-	-	-	-	-	-
3.441	-	-	-	-	-	-	-	-	-	-
3.431	-	-	-	-	-	-	-	-	-	-
3.412	-	-	-	-	-	-	-	-	-	-
2.796	-	-	-	-	-	-	-	-	-	-
2.574	-	-	-	-	-	-	-	-	-	-
3.114	-	-	-	-	-	-	-	-	-	-
Frame number : 20000										
3.488	3.413	3.022	3.339	-	-	3.488	3.34	-	-	-
3.404	2.45	3.282	3.284	-	-	-	-	-	-	-

Continues on next page

Table S4 – *Continued from previous page*

C-H	H-O	H-S	C-O	C-S	O-S	O-O	C-C	H-N	C-N	N-S
3.317	3.26	3.156	3.441	-	-	-	-	-	-	-
3.325	3.399	2.643	3.486	-	-	-	-	-	-	-
3.093	3.258	-	3.15	-	-	-	-	-	-	-
3.448	2.988	-	3.467	-	-	-	-	-	-	-
3.462	2.609	-	-	-	-	-	-	-	-	-
3.277	3.236	-	-	-	-	-	-	-	-	-
3.483	3.301	-	-	-	-	-	-	-	-	-
3.279	3.439	-	-	-	-	-	-	-	-	-
3.45	3.33	-	-	-	-	-	-	-	-	-
3.203	3.353	-	-	-	-	-	-	-	-	-
3.477	3.453	-	-	-	-	-	-	-	-	-
3.307	2.806	-	-	-	-	-	-	-	-	-
3.436	2.367	-	-	-	-	-	-	-	-	-
2.478	2.359	-	-	-	-	-	-	-	-	-
2.298	3.423	-	-	-	-	-	-	-	-	-
2.728	3.114	-	-	-	-	-	-	-	-	-
3.102	-	-	-	-	-	-	-	-	-	-
3.028	-	-	-	-	-	-	-	-	-	-
2.673	-	-	-	-	-	-	-	-	-	-
3.463	-	-	-	-	-	-	-	-	-	-
3.028	-	-	-	-	-	-	-	-	-	-
2.96	-	-	-	-	-	-	-	-	-	-
3.144	-	-	-	-	-	-	-	-	-	-
3.354	-	-	-	-	-	-	-	-	-	-

Continues on next page

Table S4 – *Continued from previous page*

C-H	H-O	H-S	C-O	C-S	O-S	O-O	C-C	H-N	C-N	N-S
2.913	-	-	-	-	-	-	-	-	-	-
3.327	-	-	-	-	-	-	-	-	-	-
3.102	-	-	-	-	-	-	-	-	-	-
2.756	-	-	-	-	-	-	-	-	-	-
3.164	-	-	-	-	-	-	-	-	-	-
2.998	-	-	-	-	-	-	-	-	-	-
3.309	-	-	-	-	-	-	-	-	-	-
3.292	-	-	-	-	-	-	-	-	-	-
3.456	-	-	-	-	-	-	-	-	-	-
3.359	-	-	-	-	-	-	-	-	-	-
3.468	-	-	-	-	-	-	-	-	-	-
3.173	-	-	-	-	-	-	-	-	-	-
3.205	-	-	-	-	-	-	-	-	-	-
3.375	-	-	-	-	-	-	-	-	-	-
3.163	-	-	-	-	-	-	-	-	-	-
3.073	-	-	-	-	-	-	-	-	-	-
3.188	-	-	-	-	-	-	-	-	-	-
2.74	-	-	-	-	-	-	-	-	-	-
3.096	-	-	-	-	-	-	-	-	-	-
3.272	-	-	-	-	-	-	-	-	-	-
3.299	-	-	-	-	-	-	-	-	-	-
3.297	-	-	-	-	-	-	-	-	-	-
3.286	-	-	-	-	-	-	-	-	-	-
2.879	-	-	-	-	-	-	-	-	-	-

Continues on next page

Table S4 – *Continued from previous page*

C-H	H-O	H-S	C-O	C-S	O-S	O-O	C-C	H-N	C-N	N-S
2.597	-	-	-	-	-	-	-	-	-	-
2.961	-	-	-	-	-	-	-	-	-	-
3.498	-	-	-	-	-	-	-	-	-	-
Frame number : 30000										
3.03	2.894	3.433	3.402	-	-	-	3.478	-	-	-
3.079	3.162	3.031	3.214	-	-	-	3.416	-	-	-
3.251	2.863	2.968	3.355	-	-	-	-	-	-	-
3.016	2.809	-	2.933	-	-	-	-	-	-	-
3.449	2.734	-	3.179	-	-	-	-	-	-	-
2.61	3.384	-	3.373	-	-	-	-	-	-	-
3.414	3.486	-	-	-	-	-	-	-	-	-
3.432	3.364	-	-	-	-	-	-	-	-	-
2.96	3.364	-	-	-	-	-	-	-	-	-
2.893	3.402	-	-	-	-	-	-	-	-	-
3.47	3.039	-	-	-	-	-	-	-	-	-
3.491	3.304	-	-	-	-	-	-	-	-	-
3.295	-	-	-	-	-	-	-	-	-	-
2.475	-	-	-	-	-	-	-	-	-	-
3.119	-	-	-	-	-	-	-	-	-	-
2.792	-	-	-	-	-	-	-	-	-	-
3.364	-	-	-	-	-	-	-	-	-	-
3.416	-	-	-	-	-	-	-	-	-	-
3.365	-	-	-	-	-	-	-	-	-	-
3.378	-	-	-	-	-	-	-	-	-	-

Continues on next page

Table S4 – *Continued from previous page*

C-H	H-O	H-S	C-O	C-S	O-S	O-O	C-C	H-N	C-N	N-S
3.138	-	-	-	-	-	-	-	-	-	-
3.295	-	-	-	-	-	-	-	-	-	-
3.374	-	-	-	-	-	-	-	-	-	-
3.069	-	-	-	-	-	-	-	-	-	-
3.267	-	-	-	-	-	-	-	-	-	-
3.426	-	-	-	-	-	-	-	-	-	-
3.021	-	-	-	-	-	-	-	-	-	-
2.63	-	-	-	-	-	-	-	-	-	-
3.382	-	-	-	-	-	-	-	-	-	-
2.801	-	-	-	-	-	-	-	-	-	-
3.449	-	-	-	-	-	-	-	-	-	-
3.178	-	-	-	-	-	-	-	-	-	-
2.921	-	-	-	-	-	-	-	-	-	-
2.485	-	-	-	-	-	-	-	-	-	-
3.406	-	-	-	-	-	-	-	-	-	-
3.237	-	-	-	-	-	-	-	-	-	-
3.079	-	-	-	-	-	-	-	-	-	-
3.345	-	-	-	-	-	-	-	-	-	-
3.175	-	-	-	-	-	-	-	-	-	-
3.108	-	-	-	-	-	-	-	-	-	-
Frame number : 40000										
3.172	3.183	2.944	3.202	-	-	3.143	3.418	-	-	-
3.48	2.096	3.355	3.154	-	-	-	3.433	-	-	-
3.443	3.248	-	3.218	-	-	-	3.373	-	-	-

Continues on next page

Table S4 – *Continued from previous page*

C-H	H-O	H-S	C-O	C-S	O-S	O-O	C-C	H-N	C-N	N-S
3.456	3.072	-	-	-	-	-	3.486	-	-	-
3.469	2.974	-	-	-	-	-	3.291	-	-	-
3.41	2.876	-	-	-	-	-	3.494	-	-	-
3.377	3.047	-	-	-	-	-	3.323	-	-	-
3.318	3.065	-	-	-	-	-	3.439	-	-	-
3.022	3.22	-	-	-	-	-	-	-	-	-
2.977	2.895	-	-	-	-	-	-	-	-	-
2.965	2.905	-	-	-	-	-	-	-	-	-
3.416	-	-	-	-	-	-	-	-	-	-
3.397	-	-	-	-	-	-	-	-	-	-
3.447	-	-	-	-	-	-	-	-	-	-
2.33	-	-	-	-	-	-	-	-	-	-
2.735	-	-	-	-	-	-	-	-	-	-
3.274	-	-	-	-	-	-	-	-	-	-
3.479	-	-	-	-	-	-	-	-	-	-
3.005	-	-	-	-	-	-	-	-	-	-
2.359	-	-	-	-	-	-	-	-	-	-
3.467	-	-	-	-	-	-	-	-	-	-
3.305	-	-	-	-	-	-	-	-	-	-
3.421	-	-	-	-	-	-	-	-	-	-
3.474	-	-	-	-	-	-	-	-	-	-
3.143	-	-	-	-	-	-	-	-	-	-
2.573	-	-	-	-	-	-	-	-	-	-
2.683	-	-	-	-	-	-	-	-	-	-

Continues on next page

Table S4 – *Continued from previous page*

C-H	H-O	H-S	C-O	C-S	O-S	O-O	C-C	H-N	C-N	N-S
3.242	-	-	-	-	-	-	-	-	-	-
3.421	-	-	-	-	-	-	-	-	-	-
3.443	-	-	-	-	-	-	-	-	-	-
3.37	-	-	-	-	-	-	-	-	-	-
3.139	-	-	-	-	-	-	-	-	-	-
3.351	-	-	-	-	-	-	-	-	-	-
3.463	-	-	-	-	-	-	-	-	-	-
3.487	-	-	-	-	-	-	-	-	-	-
3.267	-	-	-	-	-	-	-	-	-	-
3.128	-	-	-	-	-	-	-	-	-	-
3.418	-	-	-	-	-	-	-	-	-	-
2.78	-	-	-	-	-	-	-	-	-	-
2.832	-	-	-	-	-	-	-	-	-	-
3.458	-	-	-	-	-	-	-	-	-	-
3.187	-	-	-	-	-	-	-	-	-	-
3.056	-	-	-	-	-	-	-	-	-	-
2.96	-	-	-	-	-	-	-	-	-	-
3.443	-	-	-	-	-	-	-	-	-	-
3.38	-	-	-	-	-	-	-	-	-	-
3.385	-	-	-	-	-	-	-	-	-	-
Frame number : 50000										
3.02	2.935	3.11	3.439	-	-	-	3.433	-	-	-
3.151	2.851	3.458	3.206	-	-	-	3.44	-	-	-
3.12	3.201	2.98	3.247	-	-	-	-	-	-	-

Continues on next page

Table S4 – *Continued from previous page*

C-H	H-O	H-S	C-O	C-S	O-S	O-O	C-C	H-N	C-N	N-S
2.771	2.56	-	3.379	-	-	-	-	-	-	-
3.059	3.066	-	3.418	-	-	-	-	-	-	-
3.258	3.193	-	-	-	-	-	-	-	-	-
3.203	3.045	-	-	-	-	-	-	-	-	-
3.199	3.388	-	-	-	-	-	-	-	-	-
2.986	3.223	-	-	-	-	-	-	-	-	-
3.321	2.367	-	-	-	-	-	-	-	-	-
3.165	2.802	-	-	-	-	-	-	-	-	-
3.395	-	-	-	-	-	-	-	-	-	-
3.203	-	-	-	-	-	-	-	-	-	-
3.017	-	-	-	-	-	-	-	-	-	-
3.19	-	-	-	-	-	-	-	-	-	-
3.455	-	-	-	-	-	-	-	-	-	-
2.516	-	-	-	-	-	-	-	-	-	-
2.829	-	-	-	-	-	-	-	-	-	-
3.331	-	-	-	-	-	-	-	-	-	-
3.305	-	-	-	-	-	-	-	-	-	-
2.728	-	-	-	-	-	-	-	-	-	-
3.196	-	-	-	-	-	-	-	-	-	-
3.242	-	-	-	-	-	-	-	-	-	-
2.761	-	-	-	-	-	-	-	-	-	-
3.015	-	-	-	-	-	-	-	-	-	-
3.381	-	-	-	-	-	-	-	-	-	-
2.879	-	-	-	-	-	-	-	-	-	-

Continues on next page

Table S4 – *Continued from previous page*

C-H	H-O	H-S	C-O	C-S	O-S	O-O	C-C	H-N	C-N	N-S
3.413	-	-	-	-	-	-	-	-	-	-
3.373	-	-	-	-	-	-	-	-	-	-
3.031	-	-	-	-	-	-	-	-	-	-
3.231	-	-	-	-	-	-	-	-	-	-
3.06	-	-	-	-	-	-	-	-	-	-
3.144	-	-	-	-	-	-	-	-	-	-
3.213	-	-	-	-	-	-	-	-	-	-
3.365	-	-	-	-	-	-	-	-	-	-
3.185	-	-	-	-	-	-	-	-	-	-
3.407	-	-	-	-	-	-	-	-	-	-
3.17	-	-	-	-	-	-	-	-	-	-
3.38	-	-	-	-	-	-	-	-	-	-
2.943	-	-	-	-	-	-	-	-	-	-
3.393	-	-	-	-	-	-	-	-	-	-
3.426	-	-	-	-	-	-	-	-	-	-
3.36	-	-	-	-	-	-	-	-	-	-
3.089	-	-	-	-	-	-	-	-	-	-
3.221	-	-	-	-	-	-	-	-	-	-
3.223	-	-	-	-	-	-	-	-	-	-
Frame number : 60000										
3.438	3.409	3.479	-	-	-	-	-	-	-	-
3.076	2.791	3.244	-	-	-	-	-	-	-	-
3.443	3.397	3.331	-	-	-	-	-	-	-	-
3.301	3.177	3.449	-	-	-	-	-	-	-	-

Continues on next page

Table S4 – *Continued from previous page*

C-H	H-O	H-S	C-O	C-S	O-S	O-O	C-C	H-N	C-N	N-S
3.405	2.586	-	-	-	-	-	-	-	-	-
2.915	2.765	-	-	-	-	-	-	-	-	-
3.428	3.365	-	-	-	-	-	-	-	-	-
3.313	-	-	-	-	-	-	-	-	-	-
3.078	-	-	-	-	-	-	-	-	-	-
3.487	-	-	-	-	-	-	-	-	-	-
2.9	-	-	-	-	-	-	-	-	-	-
3.084	-	-	-	-	-	-	-	-	-	-
2.836	-	-	-	-	-	-	-	-	-	-
3.5	-	-	-	-	-	-	-	-	-	-
3.165	-	-	-	-	-	-	-	-	-	-
3.328	-	-	-	-	-	-	-	-	-	-
3.387	-	-	-	-	-	-	-	-	-	-
2.928	-	-	-	-	-	-	-	-	-	-
3.46	-	-	-	-	-	-	-	-	-	-
3.099	-	-	-	-	-	-	-	-	-	-
2.664	-	-	-	-	-	-	-	-	-	-
2.945	-	-	-	-	-	-	-	-	-	-
3.156	-	-	-	-	-	-	-	-	-	-
3.16	-	-	-	-	-	-	-	-	-	-
3.15	-	-	-	-	-	-	-	-	-	-
2.916	-	-	-	-	-	-	-	-	-	-
3.233	-	-	-	-	-	-	-	-	-	-
2.974	-	-	-	-	-	-	-	-	-	-

Continues on next page

Table S4 – *Continued from previous page*

C-H	H-O	H-S	C-O	C-S	O-S	O-O	C-C	H-N	C-N	N-S
3.204	-	-	-	-	-	-	-	-	-	-
3.459	-	-	-	-	-	-	-	-	-	-
3.171	-	-	-	-	-	-	-	-	-	-
3.057	-	-	-	-	-	-	-	-	-	-
2.702	-	-	-	-	-	-	-	-	-	-
3.374	-	-	-	-	-	-	-	-	-	-
Frame number : 70000										
3.15	3.345	3.281	3.357	-	-	-	3.329	-	-	-
3.415	3.08	2.97	-	-	-	-	3.482	-	-	-
2.795	3.121	3.414	-	-	-	-	3.28	-	-	-
3.256	2.685	3.37	-	-	-	-	3.474	-	-	-
2.834	3.404	3.341	-	-	-	-	-	-	-	-
3.147	2.849	-	-	-	-	-	-	-	-	-
3.295	3.294	-	-	-	-	-	-	-	-	-
3.385	3.302	-	-	-	-	-	-	-	-	-
3.441	-	-	-	-	-	-	-	-	-	-
3.476	-	-	-	-	-	-	-	-	-	-
3.172	-	-	-	-	-	-	-	-	-	-
3.326	-	-	-	-	-	-	-	-	-	-
3.45	-	-	-	-	-	-	-	-	-	-
3.082	-	-	-	-	-	-	-	-	-	-
3.35	-	-	-	-	-	-	-	-	-	-
3.045	-	-	-	-	-	-	-	-	-	-
2.839	-	-	-	-	-	-	-	-	-	-

Continues on next page

Table S4 – *Continued from previous page*

C-H	H-O	H-S	C-O	C-S	O-S	O-O	C-C	H-N	C-N	N-S
2.727	-	-	-	-	-	-	-	-	-	-
3.236	-	-	-	-	-	-	-	-	-	-
2.953	-	-	-	-	-	-	-	-	-	-
3.455	-	-	-	-	-	-	-	-	-	-
3.11	-	-	-	-	-	-	-	-	-	-
3.446	-	-	-	-	-	-	-	-	-	-
3.399	-	-	-	-	-	-	-	-	-	-
3.398	-	-	-	-	-	-	-	-	-	-
2.882	-	-	-	-	-	-	-	-	-	-
3.291	-	-	-	-	-	-	-	-	-	-
3.356	-	-	-	-	-	-	-	-	-	-
3.414	-	-	-	-	-	-	-	-	-	-
3.388	-	-	-	-	-	-	-	-	-	-
3.136	-	-	-	-	-	-	-	-	-	-
3.133	-	-	-	-	-	-	-	-	-	-
3.447	-	-	-	-	-	-	-	-	-	-
3.186	-	-	-	-	-	-	-	-	-	-
3.459	-	-	-	-	-	-	-	-	-	-
Frame number : 80000										
2.761	3.042	2.672	3.349	-	-	-	3.451	-	-	-
3.416	3.418	3.393	3.474	-	-	-	3.332	-	-	-
3.082	3.458	3.374	3.49	-	-	-	3.426	-	-	-
3.071	3.281	3.132	3.459	-	-	-	3.294	-	-	-
3.474	2.837	-	-	-	-	-	3.379	-	-	-

Continues on next page

Table S4 – *Continued from previous page*

C-H	H-O	H-S	C-O	C-S	O-S	O-O	C-C	H-N	C-N	N-S
3.336	3.458	-	-	-	-	-	3.381	-	-	-
3.131	2.78	-	-	-	-	-	-	-	-	-
2.723	3.132	-	-	-	-	-	-	-	-	-
2.764	3.479	-	-	-	-	-	-	-	-	-
3.222	-	-	-	-	-	-	-	-	-	-
3.428	-	-	-	-	-	-	-	-	-	-
3.382	-	-	-	-	-	-	-	-	-	-
2.681	-	-	-	-	-	-	-	-	-	-
2.449	-	-	-	-	-	-	-	-	-	-
3.35	-	-	-	-	-	-	-	-	-	-
3.0	-	-	-	-	-	-	-	-	-	-
2.785	-	-	-	-	-	-	-	-	-	-
3.039	-	-	-	-	-	-	-	-	-	-
3.237	-	-	-	-	-	-	-	-	-	-
3.216	-	-	-	-	-	-	-	-	-	-
3.292	-	-	-	-	-	-	-	-	-	-
3.439	-	-	-	-	-	-	-	-	-	-
3.274	-	-	-	-	-	-	-	-	-	-
3.35	-	-	-	-	-	-	-	-	-	-
2.841	-	-	-	-	-	-	-	-	-	-
2.665	-	-	-	-	-	-	-	-	-	-
3.108	-	-	-	-	-	-	-	-	-	-
2.991	-	-	-	-	-	-	-	-	-	-
2.697	-	-	-	-	-	-	-	-	-	-

Continues on next page

Table S4 – *Continued from previous page*

C-H	H-O	H-S	C-O	C-S	O-S	O-O	C-C	H-N	C-N	N-S
3.21	-	-	-	-	-	-	-	-	-	-
3.461	-	-	-	-	-	-	-	-	-	-
2.863	-	-	-	-	-	-	-	-	-	-
2.95	-	-	-	-	-	-	-	-	-	-
3.032	-	-	-	-	-	-	-	-	-	-
3.357	-	-	-	-	-	-	-	-	-	-
3.21	-	-	-	-	-	-	-	-	-	-
3.247	-	-	-	-	-	-	-	-	-	-
3.433	-	-	-	-	-	-	-	-	-	-
3.496	-	-	-	-	-	-	-	-	-	-
3.461	-	-	-	-	-	-	-	-	-	-
2.921	-	-	-	-	-	-	-	-	-	-
3.238	-	-	-	-	-	-	-	-	-	-
2.801	-	-	-	-	-	-	-	-	-	-
3.08	-	-	-	-	-	-	-	-	-	-
3.386	-	-	-	-	-	-	-	-	-	-
2.807	-	-	-	-	-	-	-	-	-	-
3.324	-	-	-	-	-	-	-	-	-	-
3.396	-	-	-	-	-	-	-	-	-	-
3.393	-	-	-	-	-	-	-	-	-	-
3.055	-	-	-	-	-	-	-	-	-	-
2.591	-	-	-	-	-	-	-	-	-	-
3.021	-	-	-	-	-	-	-	-	-	-
3.307	-	-	-	-	-	-	-	-	-	-

Continues on next page

Table S4 – *Continued from previous page*

C-H	H-O	H-S	C-O	C-S	O-S	O-O	C-C	H-N	C-N	N-S
3.356	-	-	-	-	-	-	-	-	-	-
Frame number : 90000										
3.27	3.294	3.425	3.142	3.357	-	-	-	-	-	-
3.037	3.367	2.608	2.991	-	-	-	-	-	-	-
3.443	3.129	3.389	3.344	-	-	-	-	-	-	-
3.439	2.897	3.301	-	-	-	-	-	-	-	-
3.142	3.428	3.478	-	-	-	-	-	-	-	-
3.117	2.113	-	-	-	-	-	-	-	-	-
3.429	3.478	-	-	-	-	-	-	-	-	-
3.195	2.866	-	-	-	-	-	-	-	-	-
3.48	3.188	-	-	-	-	-	-	-	-	-
3.384	2.633	-	-	-	-	-	-	-	-	-
3.223	3.154	-	-	-	-	-	-	-	-	-
3.435	-	-	-	-	-	-	-	-	-	-
3.326	-	-	-	-	-	-	-	-	-	-
3.431	-	-	-	-	-	-	-	-	-	-
2.747	-	-	-	-	-	-	-	-	-	-
3.183	-	-	-	-	-	-	-	-	-	-
3.443	-	-	-	-	-	-	-	-	-	-
3.063	-	-	-	-	-	-	-	-	-	-
3.192	-	-	-	-	-	-	-	-	-	-
3.289	-	-	-	-	-	-	-	-	-	-
2.725	-	-	-	-	-	-	-	-	-	-
3.427	-	-	-	-	-	-	-	-	-	-

Continues on next page

Table S4 – *Continued from previous page*

C-H	H-O	H-S	C-O	C-S	O-S	O-O	C-C	H-N	C-N	N-S
2.876	-	-	-	-	-	-	-	-	-	-
3.379	-	-	-	-	-	-	-	-	-	-
3.376	-	-	-	-	-	-	-	-	-	-
3.306	-	-	-	-	-	-	-	-	-	-
3.481	-	-	-	-	-	-	-	-	-	-
3.403	-	-	-	-	-	-	-	-	-	-
3.124	-	-	-	-	-	-	-	-	-	-
2.891	-	-	-	-	-	-	-	-	-	-
3.215	-	-	-	-	-	-	-	-	-	-
3.287	-	-	-	-	-	-	-	-	-	-
3.015	-	-	-	-	-	-	-	-	-	-
3.148	-	-	-	-	-	-	-	-	-	-
3.439	-	-	-	-	-	-	-	-	-	-
3.061	-	-	-	-	-	-	-	-	-	-
2.969	-	-	-	-	-	-	-	-	-	-
3.296	-	-	-	-	-	-	-	-	-	-
3.17	-	-	-	-	-	-	-	-	-	-
3.474	-	-	-	-	-	-	-	-	-	-
3.223	-	-	-	-	-	-	-	-	-	-
3.374	-	-	-	-	-	-	-	-	-	-
3.389	-	-	-	-	-	-	-	-	-	-
3.142	-	-	-	-	-	-	-	-	-	-
3.254	-	-	-	-	-	-	-	-	-	-
3.113	-	-	-	-	-	-	-	-	-	-

Continues on next page

Table S4 – *Continued from previous page*

C-H	H-O	H-S	C-O	C-S	O-S	O-O	C-C	H-N	C-N	N-S
2.734	-	-	-	-	-	-	-	-	-	-
2.871	-	-	-	-	-	-	-	-	-	-
2.756	-	-	-	-	-	-	-	-	-	-
3.033	-	-	-	-	-	-	-	-	-	-
Frame number : 100000										
3.324	3.438	2.752	3.414	-	3.325	3.236	3.485	-	-	-
3.208	3.495	3.478	3.407	-	-	-	3.448	-	-	-
3.479	2.756	2.93	3.32	-	-	-	-	-	-	-
3.189	3.422	3.299	-	-	-	-	-	-	-	-
3.221	2.791	-	-	-	-	-	-	-	-	-
2.89	2.685	-	-	-	-	-	-	-	-	-
3.373	2.925	-	-	-	-	-	-	-	-	-
2.757	2.294	-	-	-	-	-	-	-	-	-
3.341	2.589	-	-	-	-	-	-	-	-	-
3.37	3.143	-	-	-	-	-	-	-	-	-
2.85	3.194	-	-	-	-	-	-	-	-	-
3.125	3.461	-	-	-	-	-	-	-	-	-
3.305	3.066	-	-	-	-	-	-	-	-	-
3.206	-	-	-	-	-	-	-	-	-	-
2.82	-	-	-	-	-	-	-	-	-	-
3.124	-	-	-	-	-	-	-	-	-	-
3.092	-	-	-	-	-	-	-	-	-	-
3.222	-	-	-	-	-	-	-	-	-	-
3.429	-	-	-	-	-	-	-	-	-	-

Continues on next page

Table S4 – *Continued from previous page*

C-H	H-O	H-S	C-O	C-S	O-S	O-O	C-C	H-N	C-N	N-S
2.73	-	-	-	-	-	-	-	-	-	-
2.783	-	-	-	-	-	-	-	-	-	-
3.062	-	-	-	-	-	-	-	-	-	-
3.402	-	-	-	-	-	-	-	-	-	-
3.161	-	-	-	-	-	-	-	-	-	-
3.033	-	-	-	-	-	-	-	-	-	-
3.328	-	-	-	-	-	-	-	-	-	-
3.0	-	-	-	-	-	-	-	-	-	-
3.009	-	-	-	-	-	-	-	-	-	-
3.07	-	-	-	-	-	-	-	-	-	-
2.969	-	-	-	-	-	-	-	-	-	-
3.479	-	-	-	-	-	-	-	-	-	-
3.126	-	-	-	-	-	-	-	-	-	-
3.253	-	-	-	-	-	-	-	-	-	-
3.319	-	-	-	-	-	-	-	-	-	-
3.468	-	-	-	-	-	-	-	-	-	-
3.376	-	-	-	-	-	-	-	-	-	-
3.321	-	-	-	-	-	-	-	-	-	-
3.484	-	-	-	-	-	-	-	-	-	-
3.409	-	-	-	-	-	-	-	-	-	-
3.345	-	-	-	-	-	-	-	-	-	-
3.333	-	-	-	-	-	-	-	-	-	-
3.436	-	-	-	-	-	-	-	-	-	-
3.007	-	-	-	-	-	-	-	-	-	-

Continues on next page

Table S4 – *Continued from previous page*

C-H	H-O	H-S	C-O	C-S	O-S	O-O	C-C	H-N	C-N	N-S
2.888	-	-	-	-	-	-	-	-	-	-

Table S5 Intermolecular distances (r < 3.5 Å) for pore ZnPW⊃-2-EDOT interactions computed for every 10000 molecular dynamic frames.

C-H	H-O	H-S	C-O	C-S	O-S	O-O	C-C	H-N	C-N	N-S
Frame number : 0										
3.162	2.908	3.338	3.232	-	-	3.29	3.421	-	-	-
3.455	2.252	3.016	3.415	-	-	-	-	-	-	-
3.056	2.917	-	3.331	-	-	-	-	-	-	-
3.474	3.109	-	-	-	-	-	-	-	-	-
3.487	2.99	-	-	-	-	-	-	-	-	-
3.465	2.692	-	-	-	-	-	-	-	-	-
3.339	3.304	-	-	-	-	-	-	-	-	-
3.367	3.241	-	-	-	-	-	-	-	-	-
3.379	3.089	-	-	-	-	-	-	-	-	-
3.065	2.251	-	-	-	-	-	-	-	-	-
3.413	-	-	-	-	-	-	-	-	-	-
3.494	-	-	-	-	-	-	-	-	-	-
3.352	-	-	-	-	-	-	-	-	-	-
2.824	-	-	-	-	-	-	-	-	-	-
3.227	-	-	-	-	-	-	-	-	-	-
3.473	-	-	-	-	-	-	-	-	-	-

Continues on next page

Table S5 – *Continued from previous page*

C-H	H-O	H-S	C-O	C-S	O-S	O-O	C-C	H-N	C-N	N-S
3.318	-	-	-	-	-	-	-	-	-	-
2.863	-	-	-	-	-	-	-	-	-	-
2.603	-	-	-	-	-	-	-	-	-	-
3.472	-	-	-	-	-	-	-	-	-	-
3.18	-	-	-	-	-	-	-	-	-	-
3.054	-	-	-	-	-	-	-	-	-	-
3.313	-	-	-	-	-	-	-	-	-	-
3.363	-	-	-	-	-	-	-	-	-	-
3.297	-	-	-	-	-	-	-	-	-	-
3.28	-	-	-	-	-	-	-	-	-	-
3.365	-	-	-	-	-	-	-	-	-	-
3.139	-	-	-	-	-	-	-	-	-	-
3.48	-	-	-	-	-	-	-	-	-	-
3.3	-	-	-	-	-	-	-	-	-	-
3.431	-	-	-	-	-	-	-	-	-	-
2.959	-	-	-	-	-	-	-	-	-	-
3.292	-	-	-	-	-	-	-	-	-	-
3.32	-	-	-	-	-	-	-	-	-	-
3.382	-	-	-	-	-	-	-	-	-	-
3.153	-	-	-	-	-	-	-	-	-	-
2.979	-	-	-	-	-	-	-	-	-	-
3.037	-	-	-	-	-	-	-	-	-	-
2.791	-	-	-	-	-	-	-	-	-	-
3.153	-	-	-	-	-	-	-	-	-	-

Continues on next page

Table S5 – *Continued from previous page*

C-H	H-O	H-S	C-O	C-S	O-S	O-O	C-C	H-N	C-N	N-S
3.226	-	-	-	-	-	-	-	-	-	-
Frame number : 10000										
3.37	2.165	2.777	3.192	3.441	-	-	3.45	-	-	-
3.147	3.093	2.638	3.273	-	-	-	-	-	-	-
2.699	3.437	-	3.442	-	-	-	-	-	-	-
2.685	3.256	-	3.276	-	-	-	-	-	-	-
3.38	3.315	-	3.397	-	-	-	-	-	-	-
3.043	3.423	-	3.387	-	-	-	-	-	-	-
2.999	2.455	-	-	-	-	-	-	-	-	-
2.963	3.449	-	-	-	-	-	-	-	-	-
3.079	3.257	-	-	-	-	-	-	-	-	-
2.993	3.313	-	-	-	-	-	-	-	-	-
3.052	3.458	-	-	-	-	-	-	-	-	-
3.261	3.05	-	-	-	-	-	-	-	-	-
3.466	2.66	-	-	-	-	-	-	-	-	-
2.998	2.688	-	-	-	-	-	-	-	-	-
3.375	2.85	-	-	-	-	-	-	-	-	-
3.338	3.237	-	-	-	-	-	-	-	-	-
2.811	-	-	-	-	-	-	-	-	-	-
2.63	-	-	-	-	-	-	-	-	-	-
3.236	-	-	-	-	-	-	-	-	-	-
3.388	-	-	-	-	-	-	-	-	-	-
3.279	-	-	-	-	-	-	-	-	-	-
3.372	-	-	-	-	-	-	-	-	-	-

Continues on next page

Table S5 – *Continued from previous page*

C-H	H-O	H-S	C-O	C-S	O-S	O-O	C-C	H-N	C-N	N-S
3.488	-	-	-	-	-	-	-	-	-	-
2.771	-	-	-	-	-	-	-	-	-	-
3.182	-	-	-	-	-	-	-	-	-	-
3.453	-	-	-	-	-	-	-	-	-	-
3.081	-	-	-	-	-	-	-	-	-	-
3.074	-	-	-	-	-	-	-	-	-	-
3.299	-	-	-	-	-	-	-	-	-	-
3.112	-	-	-	-	-	-	-	-	-	-
2.917	-	-	-	-	-	-	-	-	-	-
3.262	-	-	-	-	-	-	-	-	-	-
3.471	-	-	-	-	-	-	-	-	-	-
3.441	-	-	-	-	-	-	-	-	-	-
3.431	-	-	-	-	-	-	-	-	-	-
3.412	-	-	-	-	-	-	-	-	-	-
2.796	-	-	-	-	-	-	-	-	-	-
2.574	-	-	-	-	-	-	-	-	-	-
3.114	-	-	-	-	-	-	-	-	-	-
Frame number : 20000										
3.488	3.413	3.022	3.339	-	-	3.488	3.34	-	-	-
3.404	2.45	3.282	3.284	-	-	-	-	-	-	-
3.317	3.26	3.156	3.441	-	-	-	-	-	-	-
3.325	3.399	2.643	3.486	-	-	-	-	-	-	-
3.093	3.258	-	3.15	-	-	-	-	-	-	-
3.448	2.988	-	3.467	-	-	-	-	-	-	-

Continues on next page

Table S5 – *Continued from previous page*

C-H	H-O	H-S	C-O	C-S	O-S	O-O	C-C	H-N	C-N	N-S
3.462	2.609	-	-	-	-	-	-	-	-	-
3.277	3.236	-	-	-	-	-	-	-	-	-
3.483	3.301	-	-	-	-	-	-	-	-	-
3.279	3.439	-	-	-	-	-	-	-	-	-
3.45	3.33	-	-	-	-	-	-	-	-	-
3.203	3.353	-	-	-	-	-	-	-	-	-
3.477	3.453	-	-	-	-	-	-	-	-	-
3.307	2.806	-	-	-	-	-	-	-	-	-
3.436	2.367	-	-	-	-	-	-	-	-	-
2.478	2.359	-	-	-	-	-	-	-	-	-
2.298	3.423	-	-	-	-	-	-	-	-	-
2.728	3.114	-	-	-	-	-	-	-	-	-
3.102	-	-	-	-	-	-	-	-	-	-
3.028	-	-	-	-	-	-	-	-	-	-
2.673	-	-	-	-	-	-	-	-	-	-
3.463	-	-	-	-	-	-	-	-	-	-
3.028	-	-	-	-	-	-	-	-	-	-
2.96	-	-	-	-	-	-	-	-	-	-
3.144	-	-	-	-	-	-	-	-	-	-
3.354	-	-	-	-	-	-	-	-	-	-
2.913	-	-	-	-	-	-	-	-	-	-
3.327	-	-	-	-	-	-	-	-	-	-
3.102	-	-	-	-	-	-	-	-	-	-
2.756	-	-	-	-	-	-	-	-	-	-

Continues on next page

Table S5 – *Continued from previous page*

C-H	H-O	H-S	C-O	C-S	O-S	O-O	C-C	H-N	C-N	N-S
3.164	-	-	-	-	-	-	-	-	-	-
2.998	-	-	-	-	-	-	-	-	-	-
3.309	-	-	-	-	-	-	-	-	-	-
3.292	-	-	-	-	-	-	-	-	-	-
3.456	-	-	-	-	-	-	-	-	-	-
3.359	-	-	-	-	-	-	-	-	-	-
3.468	-	-	-	-	-	-	-	-	-	-
3.173	-	-	-	-	-	-	-	-	-	-
3.205	-	-	-	-	-	-	-	-	-	-
3.375	-	-	-	-	-	-	-	-	-	-
3.163	-	-	-	-	-	-	-	-	-	-
3.073	-	-	-	-	-	-	-	-	-	-
3.188	-	-	-	-	-	-	-	-	-	-
2.74	-	-	-	-	-	-	-	-	-	-
3.096	-	-	-	-	-	-	-	-	-	-
3.272	-	-	-	-	-	-	-	-	-	-
3.299	-	-	-	-	-	-	-	-	-	-
3.297	-	-	-	-	-	-	-	-	-	-
3.286	-	-	-	-	-	-	-	-	-	-
2.879	-	-	-	-	-	-	-	-	-	-
2.597	-	-	-	-	-	-	-	-	-	-
2.961	-	-	-	-	-	-	-	-	-	-
3.498	-	-	-	-	-	-	-	-	-	-
Frame number : 30000										

Continues on next page

Table S5 – *Continued from previous page*

C-H	H-O	H-S	C-O	C-S	O-S	O-O	C-C	H-N	C-N	N-S
3.03	2.894	3.433	3.402	-	-	-	3.478	-	-	-
3.079	3.162	3.031	3.214	-	-	-	3.416	-	-	-
3.251	2.863	2.968	3.355	-	-	-	-	-	-	-
3.016	2.809	-	2.933	-	-	-	-	-	-	-
3.449	2.734	-	3.179	-	-	-	-	-	-	-
2.61	3.384	-	3.373	-	-	-	-	-	-	-
3.414	3.486	-	-	-	-	-	-	-	-	-
3.432	3.364	-	-	-	-	-	-	-	-	-
2.96	3.364	-	-	-	-	-	-	-	-	-
2.893	3.402	-	-	-	-	-	-	-	-	-
3.47	3.039	-	-	-	-	-	-	-	-	-
3.491	3.304	-	-	-	-	-	-	-	-	-
3.295	-	-	-	-	-	-	-	-	-	-
2.475	-	-	-	-	-	-	-	-	-	-
3.119	-	-	-	-	-	-	-	-	-	-
2.792	-	-	-	-	-	-	-	-	-	-
3.364	-	-	-	-	-	-	-	-	-	-
3.416	-	-	-	-	-	-	-	-	-	-
3.365	-	-	-	-	-	-	-	-	-	-
3.378	-	-	-	-	-	-	-	-	-	-
3.138	-	-	-	-	-	-	-	-	-	-
3.295	-	-	-	-	-	-	-	-	-	-
3.374	-	-	-	-	-	-	-	-	-	-
3.069	-	-	-	-	-	-	-	-	-	-

Continues on next page

Table S5 – *Continued from previous page*

C-H	H-O	H-S	C-O	C-S	O-S	O-O	C-C	H-N	C-N	N-S
3.267	-	-	-	-	-	-	-	-	-	-
3.426	-	-	-	-	-	-	-	-	-	-
3.021	-	-	-	-	-	-	-	-	-	-
2.63	-	-	-	-	-	-	-	-	-	-
3.382	-	-	-	-	-	-	-	-	-	-
2.801	-	-	-	-	-	-	-	-	-	-
3.449	-	-	-	-	-	-	-	-	-	-
3.178	-	-	-	-	-	-	-	-	-	-
2.921	-	-	-	-	-	-	-	-	-	-
2.485	-	-	-	-	-	-	-	-	-	-
3.406	-	-	-	-	-	-	-	-	-	-
3.237	-	-	-	-	-	-	-	-	-	-
3.079	-	-	-	-	-	-	-	-	-	-
3.345	-	-	-	-	-	-	-	-	-	-
3.175	-	-	-	-	-	-	-	-	-	-
3.108	-	-	-	-	-	-	-	-	-	-
Frame number : 40000										
3.172	3.183	2.944	3.202	-	-	3.143	3.418	-	-	-
3.48	2.096	3.355	3.154	-	-	-	3.433	-	-	-
3.443	3.248	-	3.218	-	-	-	3.373	-	-	-
3.456	3.072	-	-	-	-	-	3.486	-	-	-
3.469	2.974	-	-	-	-	-	3.291	-	-	-
3.41	2.876	-	-	-	-	-	3.494	-	-	-
3.377	3.047	-	-	-	-	-	3.323	-	-	-

Continues on next page

Table S5 – *Continued from previous page*

C-H	H-O	H-S	C-O	C-S	O-S	O-O	C-C	H-N	C-N	N-S
3.318	3.065	-	-	-	-	-	3.439	-	-	-
3.022	3.22	-	-	-	-	-	-	-	-	-
2.977	2.895	-	-	-	-	-	-	-	-	-
2.965	2.905	-	-	-	-	-	-	-	-	-
3.416	-	-	-	-	-	-	-	-	-	-
3.397	-	-	-	-	-	-	-	-	-	-
3.447	-	-	-	-	-	-	-	-	-	-
2.33	-	-	-	-	-	-	-	-	-	-
2.735	-	-	-	-	-	-	-	-	-	-
3.274	-	-	-	-	-	-	-	-	-	-
3.479	-	-	-	-	-	-	-	-	-	-
3.005	-	-	-	-	-	-	-	-	-	-
2.359	-	-	-	-	-	-	-	-	-	-
3.467	-	-	-	-	-	-	-	-	-	-
3.305	-	-	-	-	-	-	-	-	-	-
3.421	-	-	-	-	-	-	-	-	-	-
3.474	-	-	-	-	-	-	-	-	-	-
3.143	-	-	-	-	-	-	-	-	-	-
2.573	-	-	-	-	-	-	-	-	-	-
2.683	-	-	-	-	-	-	-	-	-	-
3.242	-	-	-	-	-	-	-	-	-	-
3.421	-	-	-	-	-	-	-	-	-	-
3.443	-	-	-	-	-	-	-	-	-	-
3.37	-	-	-	-	-	-	-	-	-	-

Continues on next page

Table S5 – *Continued from previous page*

C-H	H-O	H-S	C-O	C-S	O-S	O-O	C-C	H-N	C-N	N-S
3.139	-	-	-	-	-	-	-	-	-	-
3.351	-	-	-	-	-	-	-	-	-	-
3.463	-	-	-	-	-	-	-	-	-	-
3.487	-	-	-	-	-	-	-	-	-	-
3.267	-	-	-	-	-	-	-	-	-	-
3.128	-	-	-	-	-	-	-	-	-	-
3.418	-	-	-	-	-	-	-	-	-	-
2.78	-	-	-	-	-	-	-	-	-	-
2.832	-	-	-	-	-	-	-	-	-	-
3.458	-	-	-	-	-	-	-	-	-	-
3.187	-	-	-	-	-	-	-	-	-	-
3.056	-	-	-	-	-	-	-	-	-	-
2.96	-	-	-	-	-	-	-	-	-	-
3.443	-	-	-	-	-	-	-	-	-	-
3.38	-	-	-	-	-	-	-	-	-	-
3.385	-	-	-	-	-	-	-	-	-	-
Frame number : 50000										
3.02	2.935	3.11	3.439	-	-	-	3.433	-	-	-
3.151	2.851	3.458	3.206	-	-	-	3.44	-	-	-
3.12	3.201	2.98	3.247	-	-	-	-	-	-	-
2.771	2.56	-	3.379	-	-	-	-	-	-	-
3.059	3.066	-	3.418	-	-	-	-	-	-	-
3.258	3.193	-	-	-	-	-	-	-	-	-
3.203	3.045	-	-	-	-	-	-	-	-	-

Continues on next page

Table S5 – *Continued from previous page*

C-H	H-O	H-S	C-O	C-S	O-S	O-O	C-C	H-N	C-N	N-S
3.199	3.388	-	-	-	-	-	-	-	-	-
2.986	3.223	-	-	-	-	-	-	-	-	-
3.321	2.367	-	-	-	-	-	-	-	-	-
3.165	2.802	-	-	-	-	-	-	-	-	-
3.395	-	-	-	-	-	-	-	-	-	-
3.203	-	-	-	-	-	-	-	-	-	-
3.017	-	-	-	-	-	-	-	-	-	-
3.19	-	-	-	-	-	-	-	-	-	-
3.455	-	-	-	-	-	-	-	-	-	-
2.516	-	-	-	-	-	-	-	-	-	-
2.829	-	-	-	-	-	-	-	-	-	-
3.331	-	-	-	-	-	-	-	-	-	-
3.305	-	-	-	-	-	-	-	-	-	-
2.728	-	-	-	-	-	-	-	-	-	-
3.196	-	-	-	-	-	-	-	-	-	-
3.242	-	-	-	-	-	-	-	-	-	-
2.761	-	-	-	-	-	-	-	-	-	-
3.015	-	-	-	-	-	-	-	-	-	-
3.381	-	-	-	-	-	-	-	-	-	-
2.879	-	-	-	-	-	-	-	-	-	-
3.413	-	-	-	-	-	-	-	-	-	-
3.373	-	-	-	-	-	-	-	-	-	-
3.031	-	-	-	-	-	-	-	-	-	-
3.231	-	-	-	-	-	-	-	-	-	-

Continues on next page

Table S5 – *Continued from previous page*

C-H	H-O	H-S	C-O	C-S	O-S	O-O	C-C	H-N	C-N	N-S
3.06	-	-	-	-	-	-	-	-	-	-
3.144	-	-	-	-	-	-	-	-	-	-
3.213	-	-	-	-	-	-	-	-	-	-
3.365	-	-	-	-	-	-	-	-	-	-
3.185	-	-	-	-	-	-	-	-	-	-
3.407	-	-	-	-	-	-	-	-	-	-
3.17	-	-	-	-	-	-	-	-	-	-
3.38	-	-	-	-	-	-	-	-	-	-
2.943	-	-	-	-	-	-	-	-	-	-
3.393	-	-	-	-	-	-	-	-	-	-
3.426	-	-	-	-	-	-	-	-	-	-
3.36	-	-	-	-	-	-	-	-	-	-
3.089	-	-	-	-	-	-	-	-	-	-
3.221	-	-	-	-	-	-	-	-	-	-
3.223	-	-	-	-	-	-	-	-	-	-
Frame number : 60000										
3.438	3.409	3.479	-	-	-	-	-	-	-	-
3.076	2.791	3.244	-	-	-	-	-	-	-	-
3.443	3.397	3.331	-	-	-	-	-	-	-	-
3.301	3.177	3.449	-	-	-	-	-	-	-	-
3.405	2.586	-	-	-	-	-	-	-	-	-
2.915	2.765	-	-	-	-	-	-	-	-	-
3.428	3.365	-	-	-	-	-	-	-	-	-
3.313	-	-	-	-	-	-	-	-	-	-

Continues on next page

Table S5 – *Continued from previous page*

C-H	H-O	H-S	C-O	C-S	O-S	O-O	C-C	H-N	C-N	N-S
3.078	-	-	-	-	-	-	-	-	-	-
3.487	-	-	-	-	-	-	-	-	-	-
2.9	-	-	-	-	-	-	-	-	-	-
3.084	-	-	-	-	-	-	-	-	-	-
2.836	-	-	-	-	-	-	-	-	-	-
3.5	-	-	-	-	-	-	-	-	-	-
3.165	-	-	-	-	-	-	-	-	-	-
3.328	-	-	-	-	-	-	-	-	-	-
3.387	-	-	-	-	-	-	-	-	-	-
2.928	-	-	-	-	-	-	-	-	-	-
3.46	-	-	-	-	-	-	-	-	-	-
3.099	-	-	-	-	-	-	-	-	-	-
2.664	-	-	-	-	-	-	-	-	-	-
2.945	-	-	-	-	-	-	-	-	-	-
3.156	-	-	-	-	-	-	-	-	-	-
3.16	-	-	-	-	-	-	-	-	-	-
3.15	-	-	-	-	-	-	-	-	-	-
2.916	-	-	-	-	-	-	-	-	-	-
3.233	-	-	-	-	-	-	-	-	-	-
2.974	-	-	-	-	-	-	-	-	-	-
3.204	-	-	-	-	-	-	-	-	-	-
3.459	-	-	-	-	-	-	-	-	-	-
3.171	-	-	-	-	-	-	-	-	-	-
3.057	-	-	-	-	-	-	-	-	-	-

Continues on next page

Table S5 – *Continued from previous page*

C-H	H-O	H-S	C-O	C-S	O-S	O-O	C-C	H-N	C-N	N-S
2.702	-	-	-	-	-	-	-	-	-	-
3.374	-	-	-	-	-	-	-	-	-	-
Frame number : 70000										
3.15	3.345	3.281	3.357	-	-	-	3.329	-	-	-
3.415	3.08	2.97	-	-	-	-	3.482	-	-	-
2.795	3.121	3.414	-	-	-	-	3.28	-	-	-
3.256	2.685	3.37	-	-	-	-	3.474	-	-	-
2.834	3.404	3.341	-	-	-	-	-	-	-	-
3.147	2.849	-	-	-	-	-	-	-	-	-
3.295	3.294	-	-	-	-	-	-	-	-	-
3.385	3.302	-	-	-	-	-	-	-	-	-
3.441	-	-	-	-	-	-	-	-	-	-
3.476	-	-	-	-	-	-	-	-	-	-
3.172	-	-	-	-	-	-	-	-	-	-
3.326	-	-	-	-	-	-	-	-	-	-
3.45	-	-	-	-	-	-	-	-	-	-
3.082	-	-	-	-	-	-	-	-	-	-
3.35	-	-	-	-	-	-	-	-	-	-
3.045	-	-	-	-	-	-	-	-	-	-
2.839	-	-	-	-	-	-	-	-	-	-
2.727	-	-	-	-	-	-	-	-	-	-
3.236	-	-	-	-	-	-	-	-	-	-
2.953	-	-	-	-	-	-	-	-	-	-
3.455	-	-	-	-	-	-	-	-	-	-

Continues on next page

Table S5 – *Continued from previous page*

C-H	H-O	H-S	C-O	C-S	O-S	O-O	C-C	H-N	C-N	N-S
3.11	-	-	-	-	-	-	-	-	-	-
3.446	-	-	-	-	-	-	-	-	-	-
3.399	-	-	-	-	-	-	-	-	-	-
3.398	-	-	-	-	-	-	-	-	-	-
2.882	-	-	-	-	-	-	-	-	-	-
3.291	-	-	-	-	-	-	-	-	-	-
3.356	-	-	-	-	-	-	-	-	-	-
3.414	-	-	-	-	-	-	-	-	-	-
3.388	-	-	-	-	-	-	-	-	-	-
3.136	-	-	-	-	-	-	-	-	-	-
3.133	-	-	-	-	-	-	-	-	-	-
3.447	-	-	-	-	-	-	-	-	-	-
3.186	-	-	-	-	-	-	-	-	-	-
3.459	-	-	-	-	-	-	-	-	-	-
Frame number : 80000										
2.761	3.042	2.672	3.349	-	-	-	3.451	-	-	-
3.416	3.418	3.393	3.474	-	-	-	3.332	-	-	-
3.082	3.458	3.374	3.49	-	-	-	3.426	-	-	-
3.071	3.281	3.132	3.459	-	-	-	3.294	-	-	-
3.474	2.837	-	-	-	-	-	3.379	-	-	-
3.336	3.458	-	-	-	-	-	3.381	-	-	-
3.131	2.78	-	-	-	-	-	-	-	-	-
2.723	3.132	-	-	-	-	-	-	-	-	-
2.764	3.479	-	-	-	-	-	-	-	-	-

Continues on next page

Table S5 – *Continued from previous page*

C-H	H-O	H-S	C-O	C-S	O-S	O-O	C-C	H-N	C-N	N-S
3.222	-	-	-	-	-	-	-	-	-	-
3.428	-	-	-	-	-	-	-	-	-	-
3.382	-	-	-	-	-	-	-	-	-	-
2.681	-	-	-	-	-	-	-	-	-	-
2.449	-	-	-	-	-	-	-	-	-	-
3.35	-	-	-	-	-	-	-	-	-	-
3.0	-	-	-	-	-	-	-	-	-	-
2.785	-	-	-	-	-	-	-	-	-	-
3.039	-	-	-	-	-	-	-	-	-	-
3.237	-	-	-	-	-	-	-	-	-	-
3.216	-	-	-	-	-	-	-	-	-	-
3.292	-	-	-	-	-	-	-	-	-	-
3.439	-	-	-	-	-	-	-	-	-	-
3.274	-	-	-	-	-	-	-	-	-	-
3.35	-	-	-	-	-	-	-	-	-	-
2.841	-	-	-	-	-	-	-	-	-	-
2.665	-	-	-	-	-	-	-	-	-	-
3.108	-	-	-	-	-	-	-	-	-	-
2.991	-	-	-	-	-	-	-	-	-	-
2.697	-	-	-	-	-	-	-	-	-	-
3.21	-	-	-	-	-	-	-	-	-	-
3.461	-	-	-	-	-	-	-	-	-	-
2.863	-	-	-	-	-	-	-	-	-	-
2.95	-	-	-	-	-	-	-	-	-	-

Continues on next page

Table S5 – *Continued from previous page*

C-H	H-O	H-S	C-O	C-S	O-S	O-O	C-C	H-N	C-N	N-S
3.032	-	-	-	-	-	-	-	-	-	-
3.357	-	-	-	-	-	-	-	-	-	-
3.21	-	-	-	-	-	-	-	-	-	-
3.247	-	-	-	-	-	-	-	-	-	-
3.433	-	-	-	-	-	-	-	-	-	-
3.496	-	-	-	-	-	-	-	-	-	-
3.461	-	-	-	-	-	-	-	-	-	-
2.921	-	-	-	-	-	-	-	-	-	-
3.238	-	-	-	-	-	-	-	-	-	-
2.801	-	-	-	-	-	-	-	-	-	-
3.08	-	-	-	-	-	-	-	-	-	-
3.386	-	-	-	-	-	-	-	-	-	-
2.807	-	-	-	-	-	-	-	-	-	-
3.324	-	-	-	-	-	-	-	-	-	-
3.396	-	-	-	-	-	-	-	-	-	-
3.393	-	-	-	-	-	-	-	-	-	-
3.055	-	-	-	-	-	-	-	-	-	-
2.591	-	-	-	-	-	-	-	-	-	-
3.021	-	-	-	-	-	-	-	-	-	-
3.307	-	-	-	-	-	-	-	-	-	-
3.356	-	-	-	-	-	-	-	-	-	-
Frame number : 90000										
3.27	3.294	3.425	3.142	3.357	-	-	-	-	-	-
3.037	3.367	2.608	2.991	-	-	-	-	-	-	-

Continues on next page

Table S5 – *Continued from previous page*

C-H	H-O	H-S	C-O	C-S	O-S	O-O	C-C	H-N	C-N	N-S
3.443	3.129	3.389	3.344	-	-	-	-	-	-	-
3.439	2.897	3.301	-	-	-	-	-	-	-	-
3.142	3.428	3.478	-	-	-	-	-	-	-	-
3.117	2.113	-	-	-	-	-	-	-	-	-
3.429	3.478	-	-	-	-	-	-	-	-	-
3.195	2.866	-	-	-	-	-	-	-	-	-
3.48	3.188	-	-	-	-	-	-	-	-	-
3.384	2.633	-	-	-	-	-	-	-	-	-
3.223	3.154	-	-	-	-	-	-	-	-	-
3.435	-	-	-	-	-	-	-	-	-	-
3.326	-	-	-	-	-	-	-	-	-	-
3.431	-	-	-	-	-	-	-	-	-	-
2.747	-	-	-	-	-	-	-	-	-	-
3.183	-	-	-	-	-	-	-	-	-	-
3.443	-	-	-	-	-	-	-	-	-	-
3.063	-	-	-	-	-	-	-	-	-	-
3.192	-	-	-	-	-	-	-	-	-	-
3.289	-	-	-	-	-	-	-	-	-	-
2.725	-	-	-	-	-	-	-	-	-	-
3.427	-	-	-	-	-	-	-	-	-	-
2.876	-	-	-	-	-	-	-	-	-	-
3.379	-	-	-	-	-	-	-	-	-	-
3.376	-	-	-	-	-	-	-	-	-	-
3.306	-	-	-	-	-	-	-	-	-	-

Continues on next page

Table S5 – *Continued from previous page*

C-H	H-O	H-S	C-O	C-S	O-S	O-O	C-C	H-N	C-N	N-S
3.481	-	-	-	-	-	-	-	-	-	-
3.403	-	-	-	-	-	-	-	-	-	-
3.124	-	-	-	-	-	-	-	-	-	-
2.891	-	-	-	-	-	-	-	-	-	-
3.215	-	-	-	-	-	-	-	-	-	-
3.287	-	-	-	-	-	-	-	-	-	-
3.015	-	-	-	-	-	-	-	-	-	-
3.148	-	-	-	-	-	-	-	-	-	-
3.439	-	-	-	-	-	-	-	-	-	-
3.061	-	-	-	-	-	-	-	-	-	-
2.969	-	-	-	-	-	-	-	-	-	-
3.296	-	-	-	-	-	-	-	-	-	-
3.17	-	-	-	-	-	-	-	-	-	-
3.474	-	-	-	-	-	-	-	-	-	-
3.223	-	-	-	-	-	-	-	-	-	-
3.374	-	-	-	-	-	-	-	-	-	-
3.389	-	-	-	-	-	-	-	-	-	-
3.142	-	-	-	-	-	-	-	-	-	-
3.254	-	-	-	-	-	-	-	-	-	-
3.113	-	-	-	-	-	-	-	-	-	-
2.734	-	-	-	-	-	-	-	-	-	-
2.871	-	-	-	-	-	-	-	-	-	-
2.756	-	-	-	-	-	-	-	-	-	-
3.033	-	-	-	-	-	-	-	-	-	-

Continues on next page

Table S5 – *Continued from previous page*

C-H	H-O	H-S	C-O	C-S	O-S	O-O	C-C	H-N	C-N	N-S
Frame number : 100000										
3.324	3.438	2.752	3.414	-	3.325	3.236	3.485	-	-	-
3.208	3.495	3.478	3.407	-	-	-	3.448	-	-	-
3.479	2.756	2.93	3.32	-	-	-	-	-	-	-
3.189	3.422	3.299	-	-	-	-	-	-	-	-
3.221	2.791	-	-	-	-	-	-	-	-	-
2.89	2.685	-	-	-	-	-	-	-	-	-
3.373	2.925	-	-	-	-	-	-	-	-	-
2.757	2.294	-	-	-	-	-	-	-	-	-
3.341	2.589	-	-	-	-	-	-	-	-	-
3.37	3.143	-	-	-	-	-	-	-	-	-
2.85	3.194	-	-	-	-	-	-	-	-	-
3.125	3.461	-	-	-	-	-	-	-	-	-
3.305	3.066	-	-	-	-	-	-	-	-	-
3.206	-	-	-	-	-	-	-	-	-	-
2.82	-	-	-	-	-	-	-	-	-	-
3.124	-	-	-	-	-	-	-	-	-	-
3.092	-	-	-	-	-	-	-	-	-	-
3.222	-	-	-	-	-	-	-	-	-	-
3.429	-	-	-	-	-	-	-	-	-	-
2.73	-	-	-	-	-	-	-	-	-	-
2.783	-	-	-	-	-	-	-	-	-	-
3.062	-	-	-	-	-	-	-	-	-	-
3.402	-	-	-	-	-	-	-	-	-	-

Continues on next page

Table S5 – *Continued from previous page*

C-H	H-O	H-S	C-O	C-S	O-S	O-O	C-C	H-N	C-N	N-S
3.161	-	-	-	-	-	-	-	-	-	-
3.033	-	-	-	-	-	-	-	-	-	-
3.328	-	-	-	-	-	-	-	-	-	-
3.0	-	-	-	-	-	-	-	-	-	-
3.009	-	-	-	-	-	-	-	-	-	-
3.07	-	-	-	-	-	-	-	-	-	-
2.969	-	-	-	-	-	-	-	-	-	-
3.479	-	-	-	-	-	-	-	-	-	-
3.126	-	-	-	-	-	-	-	-	-	-
3.253	-	-	-	-	-	-	-	-	-	-
3.319	-	-	-	-	-	-	-	-	-	-
3.468	-	-	-	-	-	-	-	-	-	-
3.376	-	-	-	-	-	-	-	-	-	-
3.321	-	-	-	-	-	-	-	-	-	-
3.484	-	-	-	-	-	-	-	-	-	-
3.409	-	-	-	-	-	-	-	-	-	-
3.345	-	-	-	-	-	-	-	-	-	-
3.333	-	-	-	-	-	-	-	-	-	-
3.436	-	-	-	-	-	-	-	-	-	-
3.007	-	-	-	-	-	-	-	-	-	-
2.888	-	-	-	-	-	-	-	-	-	-

Table S6 Intermolecular distances (r < 3.5 Å) for surface ZnPW⊃-1-EDOT interactions computed for every 1000 molecular dynamic frames.

C-H	H-O	H-S	C-O	C-S	O-S	O-O	C-C	H-N	C-N	N-S
Frame number : 0										
3.498	3.18	3.078	3.369	-	-	3.273	-	-	-	-
3.388	2.754	-	3.486	-	-	-	-	-	-	-
2.815	2.684	-	-	-	-	-	-	-	-	-
3.354	3.214	-	-	-	-	-	-	-	-	-
3.431	-	-	-	-	-	-	-	-	-	-
3.249	-	-	-	-	-	-	-	-	-	-
2.996	-	-	-	-	-	-	-	-	-	-
3.488	-	-	-	-	-	-	-	-	-	-
3.076	-	-	-	-	-	-	-	-	-	-
3.37	-	-	-	-	-	-	-	-	-	-
3.088	-	-	-	-	-	-	-	-	-	-
3.029	-	-	-	-	-	-	-	-	-	-
3.117	-	-	-	-	-	-	-	-	-	-
3.176	-	-	-	-	-	-	-	-	-	-
3.237	-	-	-	-	-	-	-	-	-	-
3.434	-	-	-	-	-	-	-	-	-	-
3.348	-	-	-	-	-	-	-	-	-	-
3.107	-	-	-	-	-	-	-	-	-	-
3.151	-	-	-	-	-	-	-	-	-	-
3.172	-	-	-	-	-	-	-	-	-	-
Frame number : 1000										

Continues on next page

Table S6 – *Continued from previous page*

C-H	H-O	H-S	C-O	C-S	O-S	O-O	C-C	H-N	C-N	N-S
3.453	2.855	-	3.09	-	-	-	-	-	-	-
3.34	2.668	-	-	-	-	-	-	-	-	-
3.297	3.351	-	-	-	-	-	-	-	-	-
3.216	3.332	-	-	-	-	-	-	-	-	-
2.809	2.069	-	-	-	-	-	-	-	-	-
2.828	3.331	-	-	-	-	-	-	-	-	-
3.31	3.408	-	-	-	-	-	-	-	-	-
3.192	3.454	-	-	-	-	-	-	-	-	-
3.364	-	-	-	-	-	-	-	-	-	-
3.334	-	-	-	-	-	-	-	-	-	-
3.323	-	-	-	-	-	-	-	-	-	-
3.325	-	-	-	-	-	-	-	-	-	-
3.251	-	-	-	-	-	-	-	-	-	-
2.435	-	-	-	-	-	-	-	-	-	-
2.59	-	-	-	-	-	-	-	-	-	-
3.31	-	-	-	-	-	-	-	-	-	-
3.01	-	-	-	-	-	-	-	-	-	-
3.347	-	-	-	-	-	-	-	-	-	-
2.832	-	-	-	-	-	-	-	-	-	-
2.776	-	-	-	-	-	-	-	-	-	-
Frame number : 2000										
3.422	3.189	2.583	3.221	-	3.423	-	3.341	-	-	-
2.755	2.753	-	3.391	-	-	-	3.459	-	-	-
3.48	3.086	-	3.324	-	-	-	-	-	-	-

Continues on next page

Table S6 – *Continued from previous page*

C-H	H-O	H-S	C-O	C-S	O-S	O-O	C-C	H-N	C-N	N-S
2.498	-	-	3.031	-	-	-	-	-	-	-
3.444	-	-	3.06	-	-	-	-	-	-	-
2.516	-	-	2.939	-	-	-	-	-	-	-
2.677	-	-	3.49	-	-	-	-	-	-	-
3.396	-	-	-	-	-	-	-	-	-	-
3.399	-	-	-	-	-	-	-	-	-	-
3.284	-	-	-	-	-	-	-	-	-	-
2.705	-	-	-	-	-	-	-	-	-	-
3.207	-	-	-	-	-	-	-	-	-	-
3.061	-	-	-	-	-	-	-	-	-	-
3.278	-	-	-	-	-	-	-	-	-	-
3.178	-	-	-	-	-	-	-	-	-	-
3.432	-	-	-	-	-	-	-	-	-	-
Frame number : 3000										
3.451	2.98	-	3.429	-	-	-	3.443	-	-	-
3.39	-	-	3.304	-	-	-	3.5	-	-	-
3.046	-	-	2.916	-	-	-	3.222	-	-	-
3.288	-	-	3.239	-	-	-	-	-	-	-
3.121	-	-	-	-	-	-	-	-	-	-
3.488	-	-	-	-	-	-	-	-	-	-
3.428	-	-	-	-	-	-	-	-	-	-
2.686	-	-	-	-	-	-	-	-	-	-
3.233	-	-	-	-	-	-	-	-	-	-
3.047	-	-	-	-	-	-	-	-	-	-

Continues on next page

Table S6 – *Continued from previous page*

C-H	H-O	H-S	C-O	C-S	O-S	O-O	C-C	H-N	C-N	N-S
2.645	-	-	-	-	-	-	-	-	-	-
2.879	-	-	-	-	-	-	-	-	-	-
3.322	-	-	-	-	-	-	-	-	-	-
3.411	-	-	-	-	-	-	-	-	-	-
2.932	-	-	-	-	-	-	-	-	-	-
2.898	-	-	-	-	-	-	-	-	-	-
2.539	-	-	-	-	-	-	-	-	-	-
Frame number : 4000										
3.269	3.412	-	3.112	-	-	-	3.342	-	-	-
3.454	3.449	-	3.229	-	-	-	-	-	-	-
3.158	3.485	-	3.484	-	-	-	-	-	-	-
3.362	3.01	-	3.462	-	-	-	-	-	-	-
2.849	3.275	-	3.493	-	-	-	-	-	-	-
2.807	-	-	-	-	-	-	-	-	-	-
2.818	-	-	-	-	-	-	-	-	-	-
3.062	-	-	-	-	-	-	-	-	-	-
3.024	-	-	-	-	-	-	-	-	-	-
3.078	-	-	-	-	-	-	-	-	-	-
2.694	-	-	-	-	-	-	-	-	-	-
3.443	-	-	-	-	-	-	-	-	-	-
3.314	-	-	-	-	-	-	-	-	-	-
2.984	-	-	-	-	-	-	-	-	-	-
Frame number : 5000										
3.007	3.459	3.388	3.228	-	-	-	3.469	-	-	-

Continues on next page

Table S6 – *Continued from previous page*

C-H	H-O	H-S	C-O	C-S	O-S	O-O	C-C	H-N	C-N	N-S
3.002	3.426	2.945	3.406	-	-	-	3.492	-	-	-
3.231	2.472	-	3.311	-	-	-	3.287	-	-	-
2.971	3.006	-	3.011	-	-	-	-	-	-	-
3.186	2.668	-	3.329	-	-	-	-	-	-	-
3.414	3.238	-	3.316	-	-	-	-	-	-	-
3.081	2.904	-	-	-	-	-	-	-	-	-
2.886	3.431	-	-	-	-	-	-	-	-	-
2.871	2.522	-	-	-	-	-	-	-	-	-
3.113	-	-	-	-	-	-	-	-	-	-
3.198	-	-	-	-	-	-	-	-	-	-
3.166	-	-	-	-	-	-	-	-	-	-
3.43	-	-	-	-	-	-	-	-	-	-
3.152	-	-	-	-	-	-	-	-	-	-
3.078	-	-	-	-	-	-	-	-	-	-
3.368	-	-	-	-	-	-	-	-	-	-
2.588	-	-	-	-	-	-	-	-	-	-
2.417	-	-	-	-	-	-	-	-	-	-
2.872	-	-	-	-	-	-	-	-	-	-
3.008	-	-	-	-	-	-	-	-	-	-
2.646	-	-	-	-	-	-	-	-	-	-
3.428	-	-	-	-	-	-	-	-	-	-
2.859	-	-	-	-	-	-	-	-	-	-
3.228	-	-	-	-	-	-	-	-	-	-
3.353	-	-	-	-	-	-	-	-	-	-

Continues on next page

Table S6 – *Continued from previous page*

C-H	H-O	H-S	C-O	C-S	O-S	O-O	C-C	H-N	C-N	N-S
Frame number : 6000										
3.431	2.846	2.808	3.428	-	-	2.816	3.429	-	-	-
3.336	2.911	3.187	3.228	-	-	-	3.469	-	-	-
3.169	3.4	-	3.142	-	-	-	3.487	-	-	-
3.433	2.669	-	2.95	-	-	-	-	-	-	-
3.337	3.398	-	3.427	-	-	-	-	-	-	-
3.401	3.463	-	2.99	-	-	-	-	-	-	-
2.627	3.249	-	3.44	-	-	-	-	-	-	-
3.069	3.179	-	-	-	-	-	-	-	-	-
3.178	3.29	-	-	-	-	-	-	-	-	-
3.374	-	-	-	-	-	-	-	-	-	-
2.776	-	-	-	-	-	-	-	-	-	-
3.308	-	-	-	-	-	-	-	-	-	-
3.352	-	-	-	-	-	-	-	-	-	-
2.875	-	-	-	-	-	-	-	-	-	-
3.339	-	-	-	-	-	-	-	-	-	-
3.391	-	-	-	-	-	-	-	-	-	-
3.275	-	-	-	-	-	-	-	-	-	-
2.704	-	-	-	-	-	-	-	-	-	-
3.098	-	-	-	-	-	-	-	-	-	-
2.536	-	-	-	-	-	-	-	-	-	-
2.842	-	-	-	-	-	-	-	-	-	-
2.892	-	-	-	-	-	-	-	-	-	-
3.203	-	-	-	-	-	-	-	-	-	-

Continues on next page

Table S6 – *Continued from previous page*

C-H	H-O	H-S	C-O	C-S	O-S	O-O	C-C	H-N	C-N	N-S
3.493	-	-	-	-	-	-	-	-	-	-
3.021	-	-	-	-	-	-	-	-	-	-
3.396	-	-	-	-	-	-	-	-	-	-
3.178	-	-	-	-	-	-	-	-	-	-
3.157	-	-	-	-	-	-	-	-	-	-
Frame number : 7000										
3.24	3.262	-	3.057	-	-	-	3.451	-	-	-
3.446	2.902	-	3.26	-	-	-	3.479	-	-	-
3.127	3.092	-	3.336	-	-	-	3.49	-	-	-
3.27	-	-	3.102	-	-	-	3.343	-	-	-
3.429	-	-	3.161	-	-	-	-	-	-	-
3.498	-	-	3.449	-	-	-	-	-	-	-
3.077	-	-	-	-	-	-	-	-	-	-
3.261	-	-	-	-	-	-	-	-	-	-
2.589	-	-	-	-	-	-	-	-	-	-
2.622	-	-	-	-	-	-	-	-	-	-
3.337	-	-	-	-	-	-	-	-	-	-
3.086	-	-	-	-	-	-	-	-	-	-
2.944	-	-	-	-	-	-	-	-	-	-
3.091	-	-	-	-	-	-	-	-	-	-
2.746	-	-	-	-	-	-	-	-	-	-
3.136	-	-	-	-	-	-	-	-	-	-
3.008	-	-	-	-	-	-	-	-	-	-
Frame number : 8000										

Continues on next page

Table S6 – *Continued from previous page*

C-H	H-O	H-S	C-O	C-S	O-S	O-O	C-C	H-N	C-N	N-S
3.271	2.578	-	3.183	-	-	3.493	-	-	-	-
3.188	2.907	-	3.448	-	-	-	-	-	-	-
3.333	2.979	-	3.354	-	-	-	-	-	-	-
3.014	2.638	-	3.379	-	-	-	-	-	-	-
3.146	3.412	-	-	-	-	-	-	-	-	-
3.241	3.016	-	-	-	-	-	-	-	-	-
2.769	2.977	-	-	-	-	-	-	-	-	-
3.138	3.395	-	-	-	-	-	-	-	-	-
3.259	-	-	-	-	-	-	-	-	-	-
3.13	-	-	-	-	-	-	-	-	-	-
3.482	-	-	-	-	-	-	-	-	-	-
3.13	-	-	-	-	-	-	-	-	-	-
3.411	-	-	-	-	-	-	-	-	-	-
2.714	-	-	-	-	-	-	-	-	-	-
3.13	-	-	-	-	-	-	-	-	-	-
3.475	-	-	-	-	-	-	-	-	-	-
3.301	-	-	-	-	-	-	-	-	-	-
3.149	-	-	-	-	-	-	-	-	-	-
3.493	-	-	-	-	-	-	-	-	-	-
3.261	-	-	-	-	-	-	-	-	-	-
2.949	-	-	-	-	-	-	-	-	-	-
3.351	-	-	-	-	-	-	-	-	-	-
2.882	-	-	-	-	-	-	-	-	-	-
Frame number : 9000										

Continues on next page

Table S6 – *Continued from previous page*

C-H	H-O	H-S	C-O	C-S	O-S	O-O	C-C	H-N	C-N	N-S
3.49	3.447	3.454	3.13	-	-	-	3.407	-	-	-
3.266	2.433	3.075	-	-	-	-	-	-	-	-
2.643	2.938	-	-	-	-	-	-	-	-	-
2.772	3.385	-	-	-	-	-	-	-	-	-
3.1	-	-	-	-	-	-	-	-	-	-
3.18	-	-	-	-	-	-	-	-	-	-
2.874	-	-	-	-	-	-	-	-	-	-
2.91	-	-	-	-	-	-	-	-	-	-
3.345	-	-	-	-	-	-	-	-	-	-
3.336	-	-	-	-	-	-	-	-	-	-
3.489	-	-	-	-	-	-	-	-	-	-
3.313	-	-	-	-	-	-	-	-	-	-
3.177	-	-	-	-	-	-	-	-	-	-
3.307	-	-	-	-	-	-	-	-	-	-
3.429	-	-	-	-	-	-	-	-	-	-
Frame number : 10000										
3.474	3.102	3.041	3.311	-	-	-	3.405	-	-	-
2.823	2.704	3.342	2.958	-	-	-	-	-	-	-
3.314	3.395	3.484	3.187	-	-	-	-	-	-	-
3.219	2.234	-	-	-	-	-	-	-	-	-
3.131	-	-	-	-	-	-	-	-	-	-
2.418	-	-	-	-	-	-	-	-	-	-
3.047	-	-	-	-	-	-	-	-	-	-
3.27	-	-	-	-	-	-	-	-	-	-

Continues on next page

Table S6 – *Continued from previous page*

C-H	H-O	H-S	C-O	C-S	O-S	O-O	C-C	H-N	C-N	N-S
3.078	-	-	-	-	-	-	-	-	-	-
2.895	-	-	-	-	-	-	-	-	-	-
3.455	-	-	-	-	-	-	-	-	-	-
3.152	-	-	-	-	-	-	-	-	-	-
2.668	-	-	-	-	-	-	-	-	-	-
3.253	-	-	-	-	-	-	-	-	-	-
2.509	-	-	-	-	-	-	-	-	-	-
2.814	-	-	-	-	-	-	-	-	-	-
3.156	-	-	-	-	-	-	-	-	-	-
3.179	-	-	-	-	-	-	-	-	-	-
3.285	-	-	-	-	-	-	-	-	-	-
2.943	-	-	-	-	-	-	-	-	-	-
3.157	-	-	-	-	-	-	-	-	-	-
2.702	-	-	-	-	-	-	-	-	-	-
2.995	-	-	-	-	-	-	-	-	-	-
2.54	-	-	-	-	-	-	-	-	-	-
2.723	-	-	-	-	-	-	-	-	-	-
3.487	-	-	-	-	-	-	-	-	-	-
3.207	-	-	-	-	-	-	-	-	-	-

Table S7 Intermolecular distances (r < 3.5 Å) for surface ZnPW⊃-2-EDOT interactions computed for every 1000 molecular dynamic frames.

C-H	H-O	H-S	C-O	C-S	O-S	O-O	C-C	H-N	C-N	N-S
Frame number : 0										
3.347	2.94	3.193	3.367	3.475	-	-	-	-	-	-
3.498	2.738	-	3.152	3.312	-	-	-	-	-	-
3.297	3.095	-	-	3.442	-	-	-	-	-	-
3.444	3.253	-	-	3.465	-	-	-	-	-	-
3.286	3.178	-	-	3.436	-	-	-	-	-	-
3.05	2.843	-	-	3.429	-	-	-	-	-	-
3.109	2.846	-	-	3.445	-	-	-	-	-	-
3.096	3.096	-	-	-	-	-	-	-	-	-
3.162	3.409	-	-	-	-	-	-	-	-	-
3.108	-	-	-	-	-	-	-	-	-	-
2.834	-	-	-	-	-	-	-	-	-	-
3.166	-	-	-	-	-	-	-	-	-	-
2.961	-	-	-	-	-	-	-	-	-	-
3.389	-	-	-	-	-	-	-	-	-	-
3.486	-	-	-	-	-	-	-	-	-	-
3.23	-	-	-	-	-	-	-	-	-	-
3.301	-	-	-	-	-	-	-	-	-	-
3.004	-	-	-	-	-	-	-	-	-	-
3.337	-	-	-	-	-	-	-	-	-	-
3.215	-	-	-	-	-	-	-	-	-	-
3.136	-	-	-	-	-	-	-	-	-	-

Continues on next page

Table S7 – *Continued from previous page*

C-H	H-O	H-S	C-O	C-S	O-S	O-O	C-C	H-N	C-N	N-S
3.253	-	-	-	-	-	-	-	-	-	-
3.206	-	-	-	-	-	-	-	-	-	-
3.155	-	-	-	-	-	-	-	-	-	-
3.226	-	-	-	-	-	-	-	-	-	-
3.04	-	-	-	-	-	-	-	-	-	-
Frame number : 1000										
2.912	3.11	3.374	3.477	-	-	-	3.451	-	-	-
3.085	3.489	3.025	3.389	-	-	-	3.11	-	-	-
2.655	2.576	2.751	2.945	-	-	-	3.335	-	-	-
2.999	2.993	3.096	-	-	-	-	-	-	-	-
2.618	3.452	-	-	-	-	-	-	-	-	-
3.014	3.469	-	-	-	-	-	-	-	-	-
3.014	3.256	-	-	-	-	-	-	-	-	-
3.489	2.473	-	-	-	-	-	-	-	-	-
3.264	2.636	-	-	-	-	-	-	-	-	-
2.976	3.488	-	-	-	-	-	-	-	-	-
3.108	3.263	-	-	-	-	-	-	-	-	-
3.328	-	-	-	-	-	-	-	-	-	-
3.317	-	-	-	-	-	-	-	-	-	-
3.498	-	-	-	-	-	-	-	-	-	-
3.289	-	-	-	-	-	-	-	-	-	-
2.926	-	-	-	-	-	-	-	-	-	-
3.222	-	-	-	-	-	-	-	-	-	-
3.481	-	-	-	-	-	-	-	-	-	-

Continues on next page

Table S7 – *Continued from previous page*

C-H	H-O	H-S	C-O	C-S	O-S	O-O	C-C	H-N	C-N	N-S
3.167	-	-	-	-	-	-	-	-	-	-
3.241	-	-	-	-	-	-	-	-	-	-
3.482	-	-	-	-	-	-	-	-	-	-
3.487	-	-	-	-	-	-	-	-	-	-
3.241	-	-	-	-	-	-	-	-	-	-
2.879	-	-	-	-	-	-	-	-	-	-
2.664	-	-	-	-	-	-	-	-	-	-
3.369	-	-	-	-	-	-	-	-	-	-
Frame number : 2000										
3.321	3.217	3.121	3.312	-	-	3.206	3.399	-	-	-
2.859	2.604	3.143	3.125	-	-	-	3.455	-	-	-
3.089	3.127	-	2.973	-	-	-	-	-	-	-
3.2	3.324	-	3.351	-	-	-	-	-	-	-
3.473	3.072	-	3.337	-	-	-	-	-	-	-
3.323	3.241	-	-	-	-	-	-	-	-	-
3.387	3.213	-	-	-	-	-	-	-	-	-
3.025	2.624	-	-	-	-	-	-	-	-	-
3.129	-	-	-	-	-	-	-	-	-	-
2.568	-	-	-	-	-	-	-	-	-	-
3.457	-	-	-	-	-	-	-	-	-	-
3.413	-	-	-	-	-	-	-	-	-	-
3.242	-	-	-	-	-	-	-	-	-	-
3.034	-	-	-	-	-	-	-	-	-	-
3.234	-	-	-	-	-	-	-	-	-	-

Continues on next page

Table S7 – *Continued from previous page*

C-H	H-O	H-S	C-O	C-S	O-S	O-O	C-C	H-N	C-N	N-S
3.363	-	-	-	-	-	-	-	-	-	-
3.322	-	-	-	-	-	-	-	-	-	-
3.324	-	-	-	-	-	-	-	-	-	-
3.478	-	-	-	-	-	-	-	-	-	-
3.261	-	-	-	-	-	-	-	-	-	-
3.454	-	-	-	-	-	-	-	-	-	-
3.143	-	-	-	-	-	-	-	-	-	-
2.943	-	-	-	-	-	-	-	-	-	-
2.818	-	-	-	-	-	-	-	-	-	-
3.405	-	-	-	-	-	-	-	-	-	-
3.297	-	-	-	-	-	-	-	-	-	-
3.135	-	-	-	-	-	-	-	-	-	-
3.106	-	-	-	-	-	-	-	-	-	-
3.477	-	-	-	-	-	-	-	-	-	-
Frame number : 3000										
3.076	2.375	3.365	3.446	-	-	-	-	-	-	-
3.431	3.398	3.283	-	-	-	-	-	-	-	-
3.361	-	-	-	-	-	-	-	-	-	-
3.171	-	-	-	-	-	-	-	-	-	-
3.491	-	-	-	-	-	-	-	-	-	-
3.397	-	-	-	-	-	-	-	-	-	-
3.325	-	-	-	-	-	-	-	-	-	-
3.137	-	-	-	-	-	-	-	-	-	-
3.362	-	-	-	-	-	-	-	-	-	-

Continues on next page

Table S7 – *Continued from previous page*

C-H	H-O	H-S	C-O	C-S	O-S	O-O	C-C	H-N	C-N	N-S
3.329	-	-	-	-	-	-	-	-	-	-
3.387	-	-	-	-	-	-	-	-	-	-
Frame number : 4000										
3.314	3.286	3.472	3.457	-	-	-	3.363	-	-	-
3.376	3.302	3.178	3.41	-	-	-	-	-	-	-
3.293	3.13	-	-	-	-	-	-	-	-	-
3.073	2.393	-	-	-	-	-	-	-	-	-
3.372	3.102	-	-	-	-	-	-	-	-	-
3.246	-	-	-	-	-	-	-	-	-	-
3.38	-	-	-	-	-	-	-	-	-	-
2.989	-	-	-	-	-	-	-	-	-	-
2.734	-	-	-	-	-	-	-	-	-	-
2.641	-	-	-	-	-	-	-	-	-	-
2.999	-	-	-	-	-	-	-	-	-	-
3.135	-	-	-	-	-	-	-	-	-	-
3.139	-	-	-	-	-	-	-	-	-	-
3.143	-	-	-	-	-	-	-	-	-	-
3.009	-	-	-	-	-	-	-	-	-	-
2.81	-	-	-	-	-	-	-	-	-	-
3.14	-	-	-	-	-	-	-	-	-	-
2.865	-	-	-	-	-	-	-	-	-	-
3.036	-	-	-	-	-	-	-	-	-	-
3.274	-	-	-	-	-	-	-	-	-	-
3.458	-	-	-	-	-	-	-	-	-	-

Continues on next page

Table S7 – *Continued from previous page*

C-H	H-O	H-S	C-O	C-S	O-S	O-O	C-C	H-N	C-N	N-S
3.248	-	-	-	-	-	-	-	-	-	-
2.915	-	-	-	-	-	-	-	-	-	-
3.451	-	-	-	-	-	-	-	-	-	-
3.189	-	-	-	-	-	-	-	-	-	-
3.259	-	-	-	-	-	-	-	-	-	-
3.057	-	-	-	-	-	-	-	-	-	-
3.103	-	-	-	-	-	-	-	-	-	-
3.098	-	-	-	-	-	-	-	-	-	-
3.128	-	-	-	-	-	-	-	-	-	-
3.38	-	-	-	-	-	-	-	-	-	-
3.02	-	-	-	-	-	-	-	-	-	-
2.894	-	-	-	-	-	-	-	-	-	-
3.277	-	-	-	-	-	-	-	-	-	-
3.362	-	-	-	-	-	-	-	-	-	-
2.911	-	-	-	-	-	-	-	-	-	-
3.058	-	-	-	-	-	-	-	-	-	-
2.86	-	-	-	-	-	-	-	-	-	-
3.418	-	-	-	-	-	-	-	-	-	-
Frame number : 5000										
3.092	2.374	3.433	3.4	-	-	-	-	-	-	-
3.358	3.207	3.444	3.475	-	-	-	-	-	-	-
2.944	2.518	-	3.002	-	-	-	-	-	-	-
3.329	2.575	-	-	-	-	-	-	-	-	-
3.33	3.373	-	-	-	-	-	-	-	-	-

Continues on next page

Table S7 – *Continued from previous page*

C-H	H-O	H-S	C-O	C-S	O-S	O-O	C-C	H-N	C-N	N-S
3.148	2.914	-	-	-	-	-	-	-	-	-
3.152	3.47	-	-	-	-	-	-	-	-	-
3.425	2.139	-	-	-	-	-	-	-	-	-
2.966	3.049	-	-	-	-	-	-	-	-	-
2.97	-	-	-	-	-	-	-	-	-	-
3.445	-	-	-	-	-	-	-	-	-	-
3.158	-	-	-	-	-	-	-	-	-	-
2.747	-	-	-	-	-	-	-	-	-	-
2.438	-	-	-	-	-	-	-	-	-	-
3.151	-	-	-	-	-	-	-	-	-	-
3.18	-	-	-	-	-	-	-	-	-	-
3.06	-	-	-	-	-	-	-	-	-	-
3.279	-	-	-	-	-	-	-	-	-	-
3.14	-	-	-	-	-	-	-	-	-	-
3.068	-	-	-	-	-	-	-	-	-	-
3.031	-	-	-	-	-	-	-	-	-	-
3.409	-	-	-	-	-	-	-	-	-	-
2.99	-	-	-	-	-	-	-	-	-	-
3.153	-	-	-	-	-	-	-	-	-	-
3.086	-	-	-	-	-	-	-	-	-	-
3.093	-	-	-	-	-	-	-	-	-	-
3.167	-	-	-	-	-	-	-	-	-	-
3.364	-	-	-	-	-	-	-	-	-	-
3.266	-	-	-	-	-	-	-	-	-	-

Continues on next page

Table S7 – *Continued from previous page*

C-H	H-O	H-S	C-O	C-S	O-S	O-O	C-C	H-N	C-N	N-S
3.198	-	-	-	-	-	-	-	-	-	-
3.019	-	-	-	-	-	-	-	-	-	-
2.99	-	-	-	-	-	-	-	-	-	-
2.945	-	-	-	-	-	-	-	-	-	-
3.169	-	-	-	-	-	-	-	-	-	-
2.893	-	-	-	-	-	-	-	-	-	-
3.385	-	-	-	-	-	-	-	-	-	-
3.06	-	-	-	-	-	-	-	-	-	-
3.482	-	-	-	-	-	-	-	-	-	-
3.478	-	-	-	-	-	-	-	-	-	-
3.149	-	-	-	-	-	-	-	-	-	-
3.396	-	-	-	-	-	-	-	-	-	-
3.058	-	-	-	-	-	-	-	-	-	-
3.265	-	-	-	-	-	-	-	-	-	-
3.474	-	-	-	-	-	-	-	-	-	-
3.08	-	-	-	-	-	-	-	-	-	-
3.331	-	-	-	-	-	-	-	-	-	-
Frame number : 6000										
3.398	2.247	2.794	3.114	-	-	3.281	3.239	-	-	-
3.399	2.831	3.372	3.183	-	-	-	3.34	-	-	-
2.811	3.236	2.963	3.42	-	-	-	3.497	-	-	-
2.73	3.457	-	3.29	-	-	-	3.313	-	-	-
3.191	2.758	-	3.461	-	-	-	-	-	-	-
3.196	-	-	-	-	-	-	-	-	-	-

Continues on next page

Table S7 – *Continued from previous page*

C-H	H-O	H-S	C-O	C-S	O-S	O-O	C-C	H-N	C-N	N-S
3.347	-	-	-	-	-	-	-	-	-	-
2.982	-	-	-	-	-	-	-	-	-	-
3.29	-	-	-	-	-	-	-	-	-	-
3.174	-	-	-	-	-	-	-	-	-	-
3.449	-	-	-	-	-	-	-	-	-	-
3.349	-	-	-	-	-	-	-	-	-	-
3.413	-	-	-	-	-	-	-	-	-	-
3.201	-	-	-	-	-	-	-	-	-	-
3.313	-	-	-	-	-	-	-	-	-	-
3.304	-	-	-	-	-	-	-	-	-	-
3.251	-	-	-	-	-	-	-	-	-	-
3.248	-	-	-	-	-	-	-	-	-	-
3.187	-	-	-	-	-	-	-	-	-	-
2.407	-	-	-	-	-	-	-	-	-	-
2.88	-	-	-	-	-	-	-	-	-	-
3.375	-	-	-	-	-	-	-	-	-	-
3.405	-	-	-	-	-	-	-	-	-	-
3.444	-	-	-	-	-	-	-	-	-	-
2.878	-	-	-	-	-	-	-	-	-	-
3.226	-	-	-	-	-	-	-	-	-	-
3.387	-	-	-	-	-	-	-	-	-	-
3.417	-	-	-	-	-	-	-	-	-	-
3.307	-	-	-	-	-	-	-	-	-	-
2.973	-	-	-	-	-	-	-	-	-	-

Continues on next page

Table S7 – *Continued from previous page*

C-H	H-O	H-S	C-O	C-S	O-S	O-O	C-C	H-N	C-N	N-S
3.428	-	-	-	-	-	-	-	-	-	-
3.043	-	-	-	-	-	-	-	-	-	-
2.879	-	-	-	-	-	-	-	-	-	-
3.175	-	-	-	-	-	-	-	-	-	-
2.711	-	-	-	-	-	-	-	-	-	-
2.701	-	-	-	-	-	-	-	-	-	-
3.389	-	-	-	-	-	-	-	-	-	-
3.042	-	-	-	-	-	-	-	-	-	-
3.358	-	-	-	-	-	-	-	-	-	-
3.477	-	-	-	-	-	-	-	-	-	-
3.268	-	-	-	-	-	-	-	-	-	-
2.883	-	-	-	-	-	-	-	-	-	-
3.204	-	-	-	-	-	-	-	-	-	-
3.13	-	-	-	-	-	-	-	-	-	-
Frame number : 7000										
3.081	2.11	3.006	3.378	-	-	-	3.489	-	-	-
2.767	2.64	2.93	3.369	-	-	-	-	-	-	-
3.271	-	3.49	3.387	-	-	-	-	-	-	-
3.053	-	-	3.208	-	-	-	-	-	-	-
2.668	-	-	-	-	-	-	-	-	-	-
2.794	-	-	-	-	-	-	-	-	-	-
2.961	-	-	-	-	-	-	-	-	-	-
2.726	-	-	-	-	-	-	-	-	-	-
2.656	-	-	-	-	-	-	-	-	-	-

Continues on next page

Table S7 – *Continued from previous page*

C-H	H-O	H-S	C-O	C-S	O-S	O-O	C-C	H-N	C-N	N-S
3.022	-	-	-	-	-	-	-	-	-	-
2.897	-	-	-	-	-	-	-	-	-	-
3.303	-	-	-	-	-	-	-	-	-	-
3.074	-	-	-	-	-	-	-	-	-	-
3.448	-	-	-	-	-	-	-	-	-	-
3.236	-	-	-	-	-	-	-	-	-	-
3.152	-	-	-	-	-	-	-	-	-	-
3.398	-	-	-	-	-	-	-	-	-	-
2.392	-	-	-	-	-	-	-	-	-	-
2.68	-	-	-	-	-	-	-	-	-	-
3.27	-	-	-	-	-	-	-	-	-	-
3.379	-	-	-	-	-	-	-	-	-	-
Frame number : 8000										
2.914	3.299	3.115	3.468	-	-	-	3.443	-	-	-
3.003	3.354	3.198	-	-	-	-	3.5	-	-	-
3.147	2.811	3.299	-	-	-	-	-	-	-	-
2.974	3.409	-	-	-	-	-	-	-	-	-
3.327	3.154	-	-	-	-	-	-	-	-	-
3.367	3.414	-	-	-	-	-	-	-	-	-
3.194	-	-	-	-	-	-	-	-	-	-
3.211	-	-	-	-	-	-	-	-	-	-
3.272	-	-	-	-	-	-	-	-	-	-
2.853	-	-	-	-	-	-	-	-	-	-
2.95	-	-	-	-	-	-	-	-	-	-

Continues on next page

Table S7 – *Continued from previous page*

C-H	H-O	H-S	C-O	C-S	O-S	O-O	C-C	H-N	C-N	N-S
2.947	-	-	-	-	-	-	-	-	-	-
3.481	-	-	-	-	-	-	-	-	-	-
3.31	-	-	-	-	-	-	-	-	-	-
3.085	-	-	-	-	-	-	-	-	-	-
3.48	-	-	-	-	-	-	-	-	-	-
3.322	-	-	-	-	-	-	-	-	-	-
3.358	-	-	-	-	-	-	-	-	-	-
3.48	-	-	-	-	-	-	-	-	-	-
3.342	-	-	-	-	-	-	-	-	-	-
3.485	-	-	-	-	-	-	-	-	-	-
3.399	-	-	-	-	-	-	-	-	-	-
3.216	-	-	-	-	-	-	-	-	-	-
3.301	-	-	-	-	-	-	-	-	-	-
3.441	-	-	-	-	-	-	-	-	-	-
3.259	-	-	-	-	-	-	-	-	-	-
3.241	-	-	-	-	-	-	-	-	-	-
Frame number : 9000										
3.459	3.254	2.948	-	-	3.413	-	-	-	-	-
3.367	3.034	3.15	-	-	-	-	-	-	-	-
3.129	-	2.852	-	-	-	-	-	-	-	-
3.453	-	3.332	-	-	-	-	-	-	-	-
3.094	-	2.88	-	-	-	-	-	-	-	-
2.828	-	-	-	-	-	-	-	-	-	-
3.085	-	-	-	-	-	-	-	-	-	-

Continues on next page

Table S7 – *Continued from previous page*

C-H	H-O	H-S	C-O	C-S	O-S	O-O	C-C	H-N	C-N	N-S
3.485	-	-	-	-	-	-	-	-	-	-
3.49	-	-	-	-	-	-	-	-	-	-
3.037	-	-	-	-	-	-	-	-	-	-
2.517	-	-	-	-	-	-	-	-	-	-
3.169	-	-	-	-	-	-	-	-	-	-
2.809	-	-	-	-	-	-	-	-	-	-
2.593	-	-	-	-	-	-	-	-	-	-
3.312	-	-	-	-	-	-	-	-	-	-
3.225	-	-	-	-	-	-	-	-	-	-
3.282	-	-	-	-	-	-	-	-	-	-
3.465	-	-	-	-	-	-	-	-	-	-
3.383	-	-	-	-	-	-	-	-	-	-
2.823	-	-	-	-	-	-	-	-	-	-
3.349	-	-	-	-	-	-	-	-	-	-
2.845	-	-	-	-	-	-	-	-	-	-
3.332	-	-	-	-	-	-	-	-	-	-
3.208	-	-	-	-	-	-	-	-	-	-
3.48	-	-	-	-	-	-	-	-	-	-
3.051	-	-	-	-	-	-	-	-	-	-
Frame number : 10000										
3.392	3.2	2.983	3.128	-	-	-	3.301	-	-	-
3.308	3.43	-	2.974	-	-	-	3.482	-	-	-
2.853	2.954	-	3.357	-	-	-	3.466	-	-	-
3.446	3.249	-	3.439	-	-	-	3.416	-	-	-

Continues on next page

Table S7 – *Continued from previous page*

C-H	H-O	H-S	C-O	C-S	O-S	O-O	C-C	H-N	C-N	N-S
3.263	3.038	-	-	-	-	-	3.318	-	-	-
2.89	-	-	-	-	-	-	-	-	-	-
2.965	-	-	-	-	-	-	-	-	-	-
3.473	-	-	-	-	-	-	-	-	-	-
3.498	-	-	-	-	-	-	-	-	-	-
3.463	-	-	-	-	-	-	-	-	-	-
2.805	-	-	-	-	-	-	-	-	-	-
2.89	-	-	-	-	-	-	-	-	-	-
2.813	-	-	-	-	-	-	-	-	-	-
2.705	-	-	-	-	-	-	-	-	-	-
2.727	-	-	-	-	-	-	-	-	-	-
3.303	-	-	-	-	-	-	-	-	-	-
3.307	-	-	-	-	-	-	-	-	-	-
3.148	-	-	-	-	-	-	-	-	-	-
2.895	-	-	-	-	-	-	-	-	-	-
3.446	-	-	-	-	-	-	-	-	-	-
2.908	-	-	-	-	-	-	-	-	-	-
3.018	-	-	-	-	-	-	-	-	-	-

Table S8 Intermolecular distances (r < 3.5 Å) for surface ZnPW⊃-3-EDOT interactions computed for every 1000 molecular dynamic frames.

C-H	H-O	H-S	C-O	C-S	O-S	O-O	C-C	H-N	C-N	N-S
Frame number : 0										
3.33	2.755	3.136	2.984	-	-	-	3.493	-	-	-
3.291	3.348	3.099	3.397	-	-	-	3.41	-	-	-
3.282	2.931	3.113	3.433	-	-	-	3.345	-	-	-
3.355	3.16	2.653	3.465	-	-	-	-	-	-	-
3.155	3.158	-	3.317	-	-	-	-	-	-	-
3.414	-	-	3.426	-	-	-	-	-	-	-
3.181	-	-	3.478	-	-	-	-	-	-	-
3.012	-	-	-	-	-	-	-	-	-	-
3.421	-	-	-	-	-	-	-	-	-	-
3.227	-	-	-	-	-	-	-	-	-	-
3.413	-	-	-	-	-	-	-	-	-	-
3.303	-	-	-	-	-	-	-	-	-	-
3.258	-	-	-	-	-	-	-	-	-	-
2.963	-	-	-	-	-	-	-	-	-	-
3.354	-	-	-	-	-	-	-	-	-	-
3.052	-	-	-	-	-	-	-	-	-	-
Frame number : 1000										
3.38	2.727	3.115	3.233	-	-	3.183	3.381	-	-	-
3.434	3.34	2.65	3.226	-	-	3.331	-	-	-	-
2.952	3.469	-	3.033	-	-	3.458	-	-	-	-
3.364	2.341	-	3.341	-	-	2.939	-	-	-	-

Continues on next page

Table S8 – *Continued from previous page*

C-H	H-O	H-S	C-O	C-S	O-S	O-O	C-C	H-N	C-N	N-S
3.426	3.271	-	3.376	-	-	-	-	-	-	-
3.431	2.449	-	3.265	-	-	-	-	-	-	-
2.898	2.746	-	-	-	-	-	-	-	-	-
3.456	3.375	-	-	-	-	-	-	-	-	-
3.105	3.013	-	-	-	-	-	-	-	-	-
3.289	-	-	-	-	-	-	-	-	-	-
3.204	-	-	-	-	-	-	-	-	-	-
3.206	-	-	-	-	-	-	-	-	-	-
2.746	-	-	-	-	-	-	-	-	-	-
2.435	-	-	-	-	-	-	-	-	-	-
3.342	-	-	-	-	-	-	-	-	-	-
3.267	-	-	-	-	-	-	-	-	-	-
3.226	-	-	-	-	-	-	-	-	-	-
3.169	-	-	-	-	-	-	-	-	-	-
3.217	-	-	-	-	-	-	-	-	-	-
Frame number : 2000										
2.872	3.237	3.46	3.492	-	-	2.709	3.455	-	-	-
3.331	3.392	-	3.355	-	-	3.393	3.461	-	-	-
3.401	3.056	-	-	-	-	3.363	-	-	-	-
3.312	2.36	-	-	-	-	-	-	-	-	-
2.917	2.785	-	-	-	-	-	-	-	-	-
3.11	3.001	-	-	-	-	-	-	-	-	-
2.926	2.508	-	-	-	-	-	-	-	-	-
2.367	2.509	-	-	-	-	-	-	-	-	-

Continues on next page

Table S8 – *Continued from previous page*

C-H	H-O	H-S	C-O	C-S	O-S	O-O	C-C	H-N	C-N	N-S
3.026	3.381	-	-	-	-	-	-	-	-	-
2.777	-	-	-	-	-	-	-	-	-	-
3.229	-	-	-	-	-	-	-	-	-	-
2.421	-	-	-	-	-	-	-	-	-	-
3.255	-	-	-	-	-	-	-	-	-	-
3.39	-	-	-	-	-	-	-	-	-	-
3.294	-	-	-	-	-	-	-	-	-	-
2.993	-	-	-	-	-	-	-	-	-	-
3.356	-	-	-	-	-	-	-	-	-	-
3.133	-	-	-	-	-	-	-	-	-	-
2.756	-	-	-	-	-	-	-	-	-	-
2.713	-	-	-	-	-	-	-	-	-	-
3.429	-	-	-	-	-	-	-	-	-	-
3.212	-	-	-	-	-	-	-	-	-	-
3.233	-	-	-	-	-	-	-	-	-	-
3.387	-	-	-	-	-	-	-	-	-	-
3.46	-	-	-	-	-	-	-	-	-	-
3.426	-	-	-	-	-	-	-	-	-	-
3.481	-	-	-	-	-	-	-	-	-	-
3.318	-	-	-	-	-	-	-	-	-	-
Frame number : 3000										
3.364	2.709	2.91	3.467	3.471	-	-	3.259	3.439	-	-
3.429	2.96	3.32	3.326	3.357	-	-	3.448	-	-	-
3.396	3.092	-	3.27	-	-	-	3.344	-	-	-

Continues on next page

Table S8 – *Continued from previous page*

C-H	H-O	H-S	C-O	C-S	O-S	O-O	C-C	H-N	C-N	N-S
3.272	3.258	-	3.471	-	-	-	3.345	-	-	-
2.821	3.355	-	3.307	-	-	-	3.22	-	-	-
3.258	3.188	-	3.468	-	-	-	-	-	-	-
3.297	2.449	-	3.384	-	-	-	-	-	-	-
3.273	3.085	-	3.363	-	-	-	-	-	-	-
2.632	3.179	-	-	-	-	-	-	-	-	-
3.496	2.751	-	-	-	-	-	-	-	-	-
3.049	3.28	-	-	-	-	-	-	-	-	-
3.276	2.993	-	-	-	-	-	-	-	-	-
2.959	2.229	-	-	-	-	-	-	-	-	-
3.052	2.949	-	-	-	-	-	-	-	-	-
3.44	3.011	-	-	-	-	-	-	-	-	-
3.325	-	-	-	-	-	-	-	-	-	-
3.5	-	-	-	-	-	-	-	-	-	-
3.307	-	-	-	-	-	-	-	-	-	-
2.702	-	-	-	-	-	-	-	-	-	-
3.123	-	-	-	-	-	-	-	-	-	-
2.547	-	-	-	-	-	-	-	-	-	-
2.671	-	-	-	-	-	-	-	-	-	-
2.518	-	-	-	-	-	-	-	-	-	-
3.01	-	-	-	-	-	-	-	-	-	-
2.956	-	-	-	-	-	-	-	-	-	-
3.457	-	-	-	-	-	-	-	-	-	-
3.305	-	-	-	-	-	-	-	-	-	-

Continues on next page

Table S8 – *Continued from previous page*

C-H	H-O	H-S	C-O	C-S	O-S	O-O	C-C	H-N	C-N	N-S
3.168	-	-	-	-	-	-	-	-	-	-
2.832	-	-	-	-	-	-	-	-	-	-
2.905	-	-	-	-	-	-	-	-	-	-
3.348	-	-	-	-	-	-	-	-	-	-
3.32	-	-	-	-	-	-	-	-	-	-
3.499	-	-	-	-	-	-	-	-	-	-
3.179	-	-	-	-	-	-	-	-	-	-
3.0	-	-	-	-	-	-	-	-	-	-
3.419	-	-	-	-	-	-	-	-	-	-
2.832	-	-	-	-	-	-	-	-	-	-
3.463	-	-	-	-	-	-	-	-	-	-
3.183	-	-	-	-	-	-	-	-	-	-
3.468	-	-	-	-	-	-	-	-	-	-
3.499	-	-	-	-	-	-	-	-	-	-
3.189	-	-	-	-	-	-	-	-	-	-
2.839	-	-	-	-	-	-	-	-	-	-
3.086	-	-	-	-	-	-	-	-	-	-
3.442	-	-	-	-	-	-	-	-	-	-
3.224	-	-	-	-	-	-	-	-	-	-
2.966	-	-	-	-	-	-	-	-	-	-
Frame number : 4000										
3.079	2.845	3.11	3.489	-	3.309	3.485	-	-	-	-
2.966	3.484	3.114	-	-	-	-	-	-	-	-
3.289	3.359	2.999	-	-	-	-	-	-	-	-

Continues on next page

Table S8 – *Continued from previous page*

C-H	H-O	H-S	C-O	C-S	O-S	O-O	C-C	H-N	C-N	N-S
3.219	2.724	-	-	-	-	-	-	-	-	-
3.446	2.956	-	-	-	-	-	-	-	-	-
3.137	3.497	-	-	-	-	-	-	-	-	-
3.342	2.611	-	-	-	-	-	-	-	-	-
3.044	-	-	-	-	-	-	-	-	-	-
3.216	-	-	-	-	-	-	-	-	-	-
3.426	-	-	-	-	-	-	-	-	-	-
3.479	-	-	-	-	-	-	-	-	-	-
3.086	-	-	-	-	-	-	-	-	-	-
3.402	-	-	-	-	-	-	-	-	-	-
3.145	-	-	-	-	-	-	-	-	-	-
3.07	-	-	-	-	-	-	-	-	-	-
3.34	-	-	-	-	-	-	-	-	-	-
3.19	-	-	-	-	-	-	-	-	-	-
3.269	-	-	-	-	-	-	-	-	-	-
2.946	-	-	-	-	-	-	-	-	-	-
2.916	-	-	-	-	-	-	-	-	-	-
3.003	-	-	-	-	-	-	-	-	-	-
2.61	-	-	-	-	-	-	-	-	-	-
3.459	-	-	-	-	-	-	-	-	-	-
3.134	-	-	-	-	-	-	-	-	-	-
3.161	-	-	-	-	-	-	-	-	-	-
3.401	-	-	-	-	-	-	-	-	-	-
2.917	-	-	-	-	-	-	-	-	-	-

Continues on next page

Table S8 – *Continued from previous page*

C-H	H-O	H-S	C-O	C-S	O-S	O-O	C-C	H-N	C-N	N-S
2.976	-	-	-	-	-	-	-	-	-	-
3.26	-	-	-	-	-	-	-	-	-	-
Frame number : 5000										
2.945	2.534	3.46	3.457	-	-	3.299	3.459	-	-	-
3.071	2.583	-	3.111	-	-	-	3.391	-	-	-
3.356	2.54	-	-	-	-	-	3.463	-	-	-
3.406	3.008	-	-	-	-	-	-	-	-	-
3.313	2.808	-	-	-	-	-	-	-	-	-
3.195	3.474	-	-	-	-	-	-	-	-	-
3.418	3.01	-	-	-	-	-	-	-	-	-
3.127	2.357	-	-	-	-	-	-	-	-	-
3.064	3.156	-	-	-	-	-	-	-	-	-
3.211	3.345	-	-	-	-	-	-	-	-	-
3.339	3.111	-	-	-	-	-	-	-	-	-
3.466	-	-	-	-	-	-	-	-	-	-
3.493	-	-	-	-	-	-	-	-	-	-
3.136	-	-	-	-	-	-	-	-	-	-
3.074	-	-	-	-	-	-	-	-	-	-
2.996	-	-	-	-	-	-	-	-	-	-
3.051	-	-	-	-	-	-	-	-	-	-
2.499	-	-	-	-	-	-	-	-	-	-
2.563	-	-	-	-	-	-	-	-	-	-
3.339	-	-	-	-	-	-	-	-	-	-
2.83	-	-	-	-	-	-	-	-	-	-

Continues on next page

Table S8 – *Continued from previous page*

C-H	H-O	H-S	C-O	C-S	O-S	O-O	C-C	H-N	C-N	N-S
3.323	-	-	-	-	-	-	-	-	-	-
3.056	-	-	-	-	-	-	-	-	-	-
3.235	-	-	-	-	-	-	-	-	-	-
3.034	-	-	-	-	-	-	-	-	-	-
2.889	-	-	-	-	-	-	-	-	-	-
3.139	-	-	-	-	-	-	-	-	-	-
2.952	-	-	-	-	-	-	-	-	-	-
3.335	-	-	-	-	-	-	-	-	-	-
3.231	-	-	-	-	-	-	-	-	-	-
3.48	-	-	-	-	-	-	-	-	-	-
3.256	-	-	-	-	-	-	-	-	-	-
3.147	-	-	-	-	-	-	-	-	-	-
3.397	-	-	-	-	-	-	-	-	-	-
2.572	-	-	-	-	-	-	-	-	-	-
2.912	-	-	-	-	-	-	-	-	-	-
2.662	-	-	-	-	-	-	-	-	-	-
2.561	-	-	-	-	-	-	-	-	-	-
3.337	-	-	-	-	-	-	-	-	-	-
3.178	-	-	-	-	-	-	-	-	-	-
3.369	-	-	-	-	-	-	-	-	-	-
3.477	-	-	-	-	-	-	-	-	-	-
3.45	-	-	-	-	-	-	-	-	-	-
3.096	-	-	-	-	-	-	-	-	-	-
3.329	-	-	-	-	-	-	-	-	-	-

Continues on next page

Table S8 – *Continued from previous page*

C-H	H-O	H-S	C-O	C-S	O-S	O-O	C-C	H-N	C-N	N-S
3.049	-	-	-	-	-	-	-	-	-	-
3.425	-	-	-	-	-	-	-	-	-	-
Frame number : 6000										
3.354	3.101	2.363	3.492	3.409	3.464	2.798	3.272	-	-	-
2.816	3.41	3.424	3.486	3.26	-	-	3.18	-	-	-
3.185	3.413	3.447	3.378	-	-	-	3.111	-	-	-
2.614	2.989	3.406	-	-	-	-	3.438	-	-	-
2.927	3.12	2.847	-	-	-	-	-	-	-	-
3.377	1.907	3.312	-	-	-	-	-	-	-	-
3.188	3.033	-	-	-	-	-	-	-	-	-
3.388	3.277	-	-	-	-	-	-	-	-	-
3.201	3.309	-	-	-	-	-	-	-	-	-
3.051	-	-	-	-	-	-	-	-	-	-
3.219	-	-	-	-	-	-	-	-	-	-
3.177	-	-	-	-	-	-	-	-	-	-
3.011	-	-	-	-	-	-	-	-	-	-
3.041	-	-	-	-	-	-	-	-	-	-
3.156	-	-	-	-	-	-	-	-	-	-
3.459	-	-	-	-	-	-	-	-	-	-
3.407	-	-	-	-	-	-	-	-	-	-
3.001	-	-	-	-	-	-	-	-	-	-
2.701	-	-	-	-	-	-	-	-	-	-
2.883	-	-	-	-	-	-	-	-	-	-
3.466	-	-	-	-	-	-	-	-	-	-

Continues on next page

Table S8 – *Continued from previous page*

C-H	H-O	H-S	C-O	C-S	O-S	O-O	C-C	H-N	C-N	N-S
2.474	-	-	-	-	-	-	-	-	-	-
3.449	-	-	-	-	-	-	-	-	-	-
3.144	-	-	-	-	-	-	-	-	-	-
3.279	-	-	-	-	-	-	-	-	-	-
3.365	-	-	-	-	-	-	-	-	-	-
3.246	-	-	-	-	-	-	-	-	-	-
3.238	-	-	-	-	-	-	-	-	-	-
3.416	-	-	-	-	-	-	-	-	-	-
3.26	-	-	-	-	-	-	-	-	-	-
2.839	-	-	-	-	-	-	-	-	-	-
3.363	-	-	-	-	-	-	-	-	-	-
3.451	-	-	-	-	-	-	-	-	-	-
3.152	-	-	-	-	-	-	-	-	-	-
3.301	-	-	-	-	-	-	-	-	-	-
2.962	-	-	-	-	-	-	-	-	-	-
2.635	-	-	-	-	-	-	-	-	-	-
3.161	-	-	-	-	-	-	-	-	-	-
3.41	-	-	-	-	-	-	-	-	-	-
3.328	-	-	-	-	-	-	-	-	-	-
3.455	-	-	-	-	-	-	-	-	-	-
Frame number : 7000										
3.278	3.412	3.239	3.446	3.388	3.451	-	3.473	-	-	-
3.436	3.012	-	-	3.494	-	-	3.454	-	-	-
3.183	2.703	-	-	-	-	-	3.471	-	-	-

Continues on next page

Table S8 – *Continued from previous page*

C-H	H-O	H-S	C-O	C-S	O-S	O-O	C-C	H-N	C-N	N-S
3.055	3.228	-	-	-	-	-	3.456	-	-	-
3.257	3.337	-	-	-	-	-	3.456	-	-	-
3.228	-	-	-	-	-	-	-	-	-	-
3.048	-	-	-	-	-	-	-	-	-	-
3.44	-	-	-	-	-	-	-	-	-	-
2.91	-	-	-	-	-	-	-	-	-	-
3.392	-	-	-	-	-	-	-	-	-	-
2.89	-	-	-	-	-	-	-	-	-	-
3.329	-	-	-	-	-	-	-	-	-	-
3.314	-	-	-	-	-	-	-	-	-	-
3.479	-	-	-	-	-	-	-	-	-	-
3.283	-	-	-	-	-	-	-	-	-	-
2.844	-	-	-	-	-	-	-	-	-	-
3.394	-	-	-	-	-	-	-	-	-	-
3.414	-	-	-	-	-	-	-	-	-	-
2.424	-	-	-	-	-	-	-	-	-	-
3.304	-	-	-	-	-	-	-	-	-	-
3.061	-	-	-	-	-	-	-	-	-	-
2.598	-	-	-	-	-	-	-	-	-	-
3.14	-	-	-	-	-	-	-	-	-	-
3.122	-	-	-	-	-	-	-	-	-	-
2.554	-	-	-	-	-	-	-	-	-	-
2.934	-	-	-	-	-	-	-	-	-	-
3.028	-	-	-	-	-	-	-	-	-	-

Continues on next page

Table S8 – *Continued from previous page*

C-H	H-O	H-S	C-O	C-S	O-S	O-O	C-C	H-N	C-N	N-S
2.79	-	-	-	-	-	-	-	-	-	-
3.467	-	-	-	-	-	-	-	-	-	-
3.342	-	-	-	-	-	-	-	-	-	-
3.383	-	-	-	-	-	-	-	-	-	-
2.952	-	-	-	-	-	-	-	-	-	-
2.804	-	-	-	-	-	-	-	-	-	-
3.159	-	-	-	-	-	-	-	-	-	-
3.456	-	-	-	-	-	-	-	-	-	-
Frame number : 8000										
3.144	2.643	2.761	3.021	3.358	3.41	-	3.375	-	-	-
2.912	2.89	3.014	3.493	3.394	-	-	3.38	-	-	-
3.123	3.182	-	3.5	3.458	-	-	-	-	-	-
2.606	2.703	-	3.494	3.405	-	-	-	-	-	-
3.045	3.445	-	3.425	3.463	-	-	-	-	-	-
3.497	2.726	-	3.498	-	-	-	-	-	-	-
3.258	2.941	-	-	-	-	-	-	-	-	-
3.092	-	-	-	-	-	-	-	-	-	-
2.996	-	-	-	-	-	-	-	-	-	-
3.333	-	-	-	-	-	-	-	-	-	-
3.384	-	-	-	-	-	-	-	-	-	-
3.455	-	-	-	-	-	-	-	-	-	-
3.21	-	-	-	-	-	-	-	-	-	-
3.281	-	-	-	-	-	-	-	-	-	-
3.47	-	-	-	-	-	-	-	-	-	-

Continues on next page

Table S8 – *Continued from previous page*

C-H	H-O	H-S	C-O	C-S	O-S	O-O	C-C	H-N	C-N	N-S
3.46	-	-	-	-	-	-	-	-	-	-
3.203	-	-	-	-	-	-	-	-	-	-
3.091	-	-	-	-	-	-	-	-	-	-
3.184	-	-	-	-	-	-	-	-	-	-
3.35	-	-	-	-	-	-	-	-	-	-
3.288	-	-	-	-	-	-	-	-	-	-
3.315	-	-	-	-	-	-	-	-	-	-
3.467	-	-	-	-	-	-	-	-	-	-
3.303	-	-	-	-	-	-	-	-	-	-
3.287	-	-	-	-	-	-	-	-	-	-
3.061	-	-	-	-	-	-	-	-	-	-
3.134	-	-	-	-	-	-	-	-	-	-
2.967	-	-	-	-	-	-	-	-	-	-
3.107	-	-	-	-	-	-	-	-	-	-
3.253	-	-	-	-	-	-	-	-	-	-
2.925	-	-	-	-	-	-	-	-	-	-
2.883	-	-	-	-	-	-	-	-	-	-
3.183	-	-	-	-	-	-	-	-	-	-
2.919	-	-	-	-	-	-	-	-	-	-
3.394	-	-	-	-	-	-	-	-	-	-
3.119	-	-	-	-	-	-	-	-	-	-
3.404	-	-	-	-	-	-	-	-	-	-
3.197	-	-	-	-	-	-	-	-	-	-
3.495	-	-	-	-	-	-	-	-	-	-

Continues on next page

Table S8 – *Continued from previous page*

C-H	H-O	H-S	C-O	C-S	O-S	O-O	C-C	H-N	C-N	N-S
Frame number : 9000										
3.333	3.415	3.173	3.057	3.316	-	-	3.1	-	-	-
3.249	2.425	2.725	3.318	3.409	-	-	3.295	-	-	-
3.485	3.028	-	2.766	-	-	-	3.462	-	-	-
2.947	3.498	-	3.473	-	-	-	3.47	-	-	-
3.31	2.27	-	-	-	-	-	3.251	-	-	-
3.323	2.528	-	-	-	-	-	3.469	-	-	-
3.222	2.837	-	-	-	-	-	-	-	-	-
3.191	3.319	-	-	-	-	-	-	-	-	-
3.321	3.067	-	-	-	-	-	-	-	-	-
2.921	3.362	-	-	-	-	-	-	-	-	-
3.429	2.974	-	-	-	-	-	-	-	-	-
3.392	3.324	-	-	-	-	-	-	-	-	-
2.977	3.149	-	-	-	-	-	-	-	-	-
3.23	3.293	-	-	-	-	-	-	-	-	-
2.97	2.611	-	-	-	-	-	-	-	-	-
3.172	3.459	-	-	-	-	-	-	-	-	-
2.977	-	-	-	-	-	-	-	-	-	-
3.45	-	-	-	-	-	-	-	-	-	-
3.283	-	-	-	-	-	-	-	-	-	-
2.958	-	-	-	-	-	-	-	-	-	-
3.245	-	-	-	-	-	-	-	-	-	-
3.063	-	-	-	-	-	-	-	-	-	-
3.226	-	-	-	-	-	-	-	-	-	-

Continues on next page

Table S8 – *Continued from previous page*

C-H	H-O	H-S	C-O	C-S	O-S	O-O	C-C	H-N	C-N	N-S
3.248	-	-	-	-	-	-	-	-	-	-
3.163	-	-	-	-	-	-	-	-	-	-
3.469	-	-	-	-	-	-	-	-	-	-
3.325	-	-	-	-	-	-	-	-	-	-
3.429	-	-	-	-	-	-	-	-	-	-
3.398	-	-	-	-	-	-	-	-	-	-
3.488	-	-	-	-	-	-	-	-	-	-
3.342	-	-	-	-	-	-	-	-	-	-
3.354	-	-	-	-	-	-	-	-	-	-
2.896	-	-	-	-	-	-	-	-	-	-
2.914	-	-	-	-	-	-	-	-	-	-
3.471	-	-	-	-	-	-	-	-	-	-
3.455	-	-	-	-	-	-	-	-	-	-
3.216	-	-	-	-	-	-	-	-	-	-
3.354	-	-	-	-	-	-	-	-	-	-
2.848	-	-	-	-	-	-	-	-	-	-
3.265	-	-	-	-	-	-	-	-	-	-
2.891	-	-	-	-	-	-	-	-	-	-
3.078	-	-	-	-	-	-	-	-	-	-
3.302	-	-	-	-	-	-	-	-	-	-
3.315	-	-	-	-	-	-	-	-	-	-
3.212	-	-	-	-	-	-	-	-	-	-
3.387	-	-	-	-	-	-	-	-	-	-
3.213	-	-	-	-	-	-	-	-	-	-

Continues on next page

Table S8 – *Continued from previous page*

C-H	H-O	H-S	C-O	C-S	O-S	O-O	C-C	H-N	C-N	N-S
3.197	-	-	-	-	-	-	-	-	-	-
3.383	-	-	-	-	-	-	-	-	-	-
3.369	-	-	-	-	-	-	-	-	-	-
3.483	-	-	-	-	-	-	-	-	-	-
3.114	-	-	-	-	-	-	-	-	-	-
3.11	-	-	-	-	-	-	-	-	-	-
Frame number : 10000										
3.472	3.289	3.413	3.088	3.259	-	3.48	3.44	-	-	-
3.045	3.042	-	3.379	-	-	-	3.385	-	-	-
3.026	3.03	-	3.065	-	-	-	3.197	-	-	-
2.919	3.287	-	3.275	-	-	-	3.45	-	-	-
2.96	3.316	-	3.395	-	-	-	3.404	-	-	-
3.157	3.243	-	3.337	-	-	-	3.16	-	-	-
3.239	3.388	-	3.318	-	-	-	3.417	-	-	-
2.886	3.182	-	3.468	-	-	-	3.454	-	-	-
3.302	3.453	-	3.489	-	-	-	-	-	-	-
2.883	3.317	-	-	-	-	-	-	-	-	-
2.979	3.165	-	-	-	-	-	-	-	-	-
2.932	3.35	-	-	-	-	-	-	-	-	-
3.027	3.473	-	-	-	-	-	-	-	-	-
2.918	2.942	-	-	-	-	-	-	-	-	-
3.162	3.022	-	-	-	-	-	-	-	-	-
3.189	2.907	-	-	-	-	-	-	-	-	-
3.407	2.911	-	-	-	-	-	-	-	-	-

Continues on next page

Table S8 – *Continued from previous page*

C-H	H-O	H-S	C-O	C-S	O-S	O-O	C-C	H-N	C-N	N-S
3.309	-	-	-	-	-	-	-	-	-	-
3.068	-	-	-	-	-	-	-	-	-	-
3.339	-	-	-	-	-	-	-	-	-	-
2.994	-	-	-	-	-	-	-	-	-	-
2.949	-	-	-	-	-	-	-	-	-	-
3.084	-	-	-	-	-	-	-	-	-	-
3.221	-	-	-	-	-	-	-	-	-	-
2.941	-	-	-	-	-	-	-	-	-	-
2.868	-	-	-	-	-	-	-	-	-	-
3.338	-	-	-	-	-	-	-	-	-	-
3.016	-	-	-	-	-	-	-	-	-	-
3.222	-	-	-	-	-	-	-	-	-	-
3.354	-	-	-	-	-	-	-	-	-	-
3.383	-	-	-	-	-	-	-	-	-	-
3.493	-	-	-	-	-	-	-	-	-	-
3.416	-	-	-	-	-	-	-	-	-	-
3.47	-	-	-	-	-	-	-	-	-	-
2.941	-	-	-	-	-	-	-	-	-	-
2.369	-	-	-	-	-	-	-	-	-	-
3.154	-	-	-	-	-	-	-	-	-	-
3.368	-	-	-	-	-	-	-	-	-	-
3.265	-	-	-	-	-	-	-	-	-	-
2.819	-	-	-	-	-	-	-	-	-	-
3.306	-	-	-	-	-	-	-	-	-	-

Continues on next page

Table S8 – *Continued from previous page*

C-H	H-O	H-S	C-O	C-S	O-S	O-O	C-C	H-N	C-N	N-S
2.951	-	-	-	-	-	-	-	-	-	-
2.879	-	-	-	-	-	-	-	-	-	-
2.255	-	-	-	-	-	-	-	-	-	-
2.878	-	-	-	-	-	-	-	-	-	-
2.494	-	-	-	-	-	-	-	-	-	-
3.17	-	-	-	-	-	-	-	-	-	-
3.498	-	-	-	-	-	-	-	-	-	-
3.476	-	-	-	-	-	-	-	-	-	-

Table S9 Intermolecular distances (r < 3.5 Å) for pore ZnPW$\supset$-1-Styrene interactions computed for every 10000 molecular dynamic frames.

C-H	H-O	C-O	O-O	C-C	H-N	C-N	N-O	H-Z	C-Zn	O-Zn
Frame number : 0										
3.043	2.817	-	-	3.27	-	-	-	-	-	-
3.49	3.354	-	-	3.455	-	-	-	-	-	-
3.119	2.9	-	-	3.44	-	-	-	-	-	-
3.185	-	-	-	3.412	-	-	-	-	-	-
3.016	-	-	-	-	-	-	-	-	-	-
3.367	-	-	-	-	-	-	-	-	-	-
3.074	-	-	-	-	-	-	-	-	-	-
3.421	-	-	-	-	-	-	-	-	-	-
2.973	-	-	-	-	-	-	-	-	-	-

Continues on next page

Table S9 – *Continued from previous page*

C-H	H-O	C-O	O-O	C-C	H-N	C-N	N-O	H-Z	C-Zn	O-Zn
3.23	-	-	-	-	-	-	-	-	-	-
3.221	-	-	-	-	-	-	-	-	-	-
3.051	-	-	-	-	-	-	-	-	-	-
3.453	-	-	-	-	-	-	-	-	-	-
3.352	-	-	-	-	-	-	-	-	-	-
2.951	-	-	-	-	-	-	-	-	-	-
3.364	-	-	-	-	-	-	-	-	-	-
3.475	-	-	-	-	-	-	-	-	-	-
3.243	-	-	-	-	-	-	-	-	-	-
3.465	-	-	-	-	-	-	-	-	-	-
3.461	-	-	-	-	-	-	-	-	-	-
3.419	-	-	-	-	-	-	-	-	-	-
3.09	-	-	-	-	-	-	-	-	-	-
3.242	-	-	-	-	-	-	-	-	-	-
3.321	-	-	-	-	-	-	-	-	-	-
3.436	-	-	-	-	-	-	-	-	-	-
3.458	-	-	-	-	-	-	-	-	-	-
3.312	-	-	-	-	-	-	-	-	-	-
3.216	-	-	-	-	-	-	-	-	-	-
Frame number : 10000										
2.957	3.044	-	-	-	-	-	-	-	-	-
3.254	-	-	-	-	-	-	-	-	-	-
3.261	-	-	-	-	-	-	-	-	-	-
3.041	-	-	-	-	-	-	-	-	-	-

Continues on next page

Table S9 – *Continued from previous page*

C-H	H-O	C-O	O-O	C-C	H-N	C-N	N-O	H-Z	C-Zn	O-Zn
3.466	-	-	-	-	-	-	-	-	-	-
3.183	-	-	-	-	-	-	-	-	-	-
3.36	-	-	-	-	-	-	-	-	-	-
2.897	-	-	-	-	-	-	-	-	-	-
2.819	-	-	-	-	-	-	-	-	-	-
2.677	-	-	-	-	-	-	-	-	-	-
3.211	-	-	-	-	-	-	-	-	-	-
3.485	-	-	-	-	-	-	-	-	-	-
3.163	-	-	-	-	-	-	-	-	-	-
2.575	-	-	-	-	-	-	-	-	-	-
3.108	-	-	-	-	-	-	-	-	-	-
3.386	-	-	-	-	-	-	-	-	-	-
Frame number : 20000										
3.386	2.145	3.293	-	3.419	-	-	-	-	-	-
3.281	3.35	-	-	3.441	-	-	-	-	-	-
3.259	-	-	-	-	-	-	-	-	-	-
2.731	-	-	-	-	-	-	-	-	-	-
3.2	-	-	-	-	-	-	-	-	-	-
3.151	-	-	-	-	-	-	-	-	-	-
3.156	-	-	-	-	-	-	-	-	-	-
3.194	-	-	-	-	-	-	-	-	-	-
3.423	-	-	-	-	-	-	-	-	-	-
3.026	-	-	-	-	-	-	-	-	-	-
2.857	-	-	-	-	-	-	-	-	-	-

Continues on next page

Table S9 – *Continued from previous page*

C-H	H-O	C-O	O-O	C-C	H-N	C-N	N-O	H-Z	C-Zn	O-Zn
3.495	-	-	-	-	-	-	-	-	-	-
2.96	-	-	-	-	-	-	-	-	-	-
2.71	-	-	-	-	-	-	-	-	-	-
3.04	-	-	-	-	-	-	-	-	-	-
3.181	-	-	-	-	-	-	-	-	-	-
2.767	-	-	-	-	-	-	-	-	-	-
2.644	-	-	-	-	-	-	-	-	-	-
2.764	-	-	-	-	-	-	-	-	-	-
3.412	-	-	-	-	-	-	-	-	-	-
3.464	-	-	-	-	-	-	-	-	-	-
3.424	-	-	-	-	-	-	-	-	-	-
3.369	-	-	-	-	-	-	-	-	-	-
2.671	-	-	-	-	-	-	-	-	-	-
3.005	-	-	-	-	-	-	-	-	-	-
3.334	-	-	-	-	-	-	-	-	-	-
2.969	-	-	-	-	-	-	-	-	-	-
Frame number : 30000										
3.408	3.041	-	-	3.285	-	-	-	-	-	-
3.281	3.37	-	-	-	-	-	-	-	-	-
3.002	-	-	-	-	-	-	-	-	-	-
3.337	-	-	-	-	-	-	-	-	-	-
3.31	-	-	-	-	-	-	-	-	-	-
3.097	-	-	-	-	-	-	-	-	-	-
3.456	-	-	-	-	-	-	-	-	-	-

Continues on next page

Table S9 – *Continued from previous page*

C-H	H-O	C-O	O-O	C-C	H-N	C-N	N-O	H-Z	C-Zn	O-Zn
3.469	-	-	-	-	-	-	-	-	-	-
2.961	-	-	-	-	-	-	-	-	-	-
2.98	-	-	-	-	-	-	-	-	-	-
3.215	-	-	-	-	-	-	-	-	-	-
3.344	-	-	-	-	-	-	-	-	-	-
2.553	-	-	-	-	-	-	-	-	-	-
3.052	-	-	-	-	-	-	-	-	-	-
3.487	-	-	-	-	-	-	-	-	-	-
3.158	-	-	-	-	-	-	-	-	-	-
3.264	-	-	-	-	-	-	-	-	-	-
Frame number : 40000										
3.393	-	-	-	3.26	-	-	-	-	-	-
3.016	-	-	-	-	-	-	-	-	-	-
3.29	-	-	-	-	-	-	-	-	-	-
Frame number : 50000										
Frame number : 60000										
3.378	2.766	-	-	3.462	-	-	-	-	-	-
3.14	3.175	-	-	-	-	-	-	-	-	-
2.943	-	-	-	-	-	-	-	-	-	-
3.083	-	-	-	-	-	-	-	-	-	-
3.5	-	-	-	-	-	-	-	-	-	-
3.188	-	-	-	-	-	-	-	-	-	-
3.496	-	-	-	-	-	-	-	-	-	-
3.238	-	-	-	-	-	-	-	-	-	-

Continues on next page

Table S9 – *Continued from previous page*

C-H	H-O	C-O	O-O	C-C	H-N	C-N	N-O	H-Z	C-Zn	O-Zn
2.946	-	-	-	-	-	-	-	-	-	-
3.037	-	-	-	-	-	-	-	-	-	-
2.989	-	-	-	-	-	-	-	-	-	-
2.933	-	-	-	-	-	-	-	-	-	-
3.031	-	-	-	-	-	-	-	-	-	-
3.371	-	-	-	-	-	-	-	-	-	-
3.098	-	-	-	-	-	-	-	-	-	-
3.185	-	-	-	-	-	-	-	-	-	-
3.079	-	-	-	-	-	-	-	-	-	-
3.049	-	-	-	-	-	-	-	-	-	-
3.41	-	-	-	-	-	-	-	-	-	-
3.455	-	-	-	-	-	-	-	-	-	-
2.873	-	-	-	-	-	-	-	-	-	-
3.496	-	-	-	-	-	-	-	-	-	-
3.069	-	-	-	-	-	-	-	-	-	-
Frame number : 70000										
3.163	-	-	-	-	-	-	-	-	-	-
3.426	-	-	-	-	-	-	-	-	-	-
3.336	-	-	-	-	-	-	-	-	-	-
3.402	-	-	-	-	-	-	-	-	-	-
3.472	-	-	-	-	-	-	-	-	-	-
3.317	-	-	-	-	-	-	-	-	-	-
2.858	-	-	-	-	-	-	-	-	-	-
2.977	-	-	-	-	-	-	-	-	-	-

Continues on next page

Table S9 – *Continued from previous page*

C-H	H-O	C-O	O-O	C-C	H-N	C-N	N-O	H-Z	C-Zn	O-Zn
Frame number : 80000										
3.311	3.197	3.408	-	3.394	-	-	-	-	-	-
3.261	2.342	-	-	3.435	-	-	-	-	-	-
3.216	-	-	-	-	-	-	-	-	-	-
3.145	-	-	-	-	-	-	-	-	-	-
2.755	-	-	-	-	-	-	-	-	-	-
3.442	-	-	-	-	-	-	-	-	-	-
3.437	-	-	-	-	-	-	-	-	-	-
2.993	-	-	-	-	-	-	-	-	-	-
3.131	-	-	-	-	-	-	-	-	-	-
2.794	-	-	-	-	-	-	-	-	-	-
3.294	-	-	-	-	-	-	-	-	-	-
3.433	-	-	-	-	-	-	-	-	-	-
3.481	-	-	-	-	-	-	-	-	-	-
3.468	-	-	-	-	-	-	-	-	-	-
3.384	-	-	-	-	-	-	-	-	-	-
3.194	-	-	-	-	-	-	-	-	-	-
Frame number : 90000										
3.484	3.419	3.487	-	-	-	-	-	-	-	-
2.508	3.022	-	-	-	-	-	-	-	-	-
3.116	-	-	-	-	-	-	-	-	-	-
3.383	-	-	-	-	-	-	-	-	-	-
2.712	-	-	-	-	-	-	-	-	-	-
3.019	-	-	-	-	-	-	-	-	-	-

Continues on next page

Table S9 – *Continued from previous page*

C-H	H-O	C-O	O-O	C-C	H-N	C-N	N-O	H-Z	C-Zn	O-Zn
3.348	-	-	-	-	-	-	-	-	-	-
3.399	-	-	-	-	-	-	-	-	-	-
3.452	-	-	-	-	-	-	-	-	-	-
3.37	-	-	-	-	-	-	-	-	-	-
2.8	-	-	-	-	-	-	-	-	-	-
3.482	-	-	-	-	-	-	-	-	-	-
2.559	-	-	-	-	-	-	-	-	-	-
2.943	-	-	-	-	-	-	-	-	-	-
2.92	-	-	-	-	-	-	-	-	-	-
3.369	-	-	-	-	-	-	-	-	-	-
3.477	-	-	-	-	-	-	-	-	-	-
3.309	-	-	-	-	-	-	-	-	-	-
3.18	-	-	-	-	-	-	-	-	-	-
3.361	-	-	-	-	-	-	-	-	-	-
3.494	-	-	-	-	-	-	-	-	-	-
3.477	-	-	-	-	-	-	-	-	-	-
3.27	-	-	-	-	-	-	-	-	-	-
Frame number : 100000										
3.463	3.244	-	-	-	-	-	-	-	-	-
3.386	-	-	-	-	-	-	-	-	-	-
3.442	-	-	-	-	-	-	-	-	-	-
3.384	-	-	-	-	-	-	-	-	-	-

Table S10 Intermolecular distances (r < 3.5 Å) for pore ZnPW⊃-2-Styrene interactions computed for every 10000 molecular dynamic frames.

C-H	H-O	C-O	O-O	C-C	H-N	C-N	N-O	H-Z	C-Zn	O-Zn
Frame number : 0										
3.123	-	-	-	3.456	-	-	-	-	-	-
2.842	-	-	-	-	-	-	-	-	-	-
3.48	-	-	-	-	-	-	-	-	-	-
3.378	-	-	-	-	-	-	-	-	-	-
3.393	-	-	-	-	-	-	-	-	-	-
2.94	-	-	-	-	-	-	-	-	-	-
2.756	-	-	-	-	-	-	-	-	-	-
2.739	-	-	-	-	-	-	-	-	-	-
3.2	-	-	-	-	-	-	-	-	-	-
2.932	-	-	-	-	-	-	-	-	-	-
3.426	-	-	-	-	-	-	-	-	-	-
3.283	-	-	-	-	-	-	-	-	-	-
2.945	-	-	-	-	-	-	-	-	-	-
2.798	-	-	-	-	-	-	-	-	-	-
3.226	-	-	-	-	-	-	-	-	-	-
3.334	-	-	-	-	-	-	-	-	-	-
3.191	-	-	-	-	-	-	-	-	-	-
3.412	-	-	-	-	-	-	-	-	-	-
3.351	-	-	-	-	-	-	-	-	-	-
3.159	-	-	-	-	-	-	-	-	-	-
3.497	-	-	-	-	-	-	-	-	-	-

Continues on next page

Table S10 – *Continued from previous page*

C-H	H-O	C-O	O-O	C-C	H-N	C-N	N-O	H-Z	C-Zn	O-Zn
3.353	-	-	-	-	-	-	-	-	-	-
3.177	-	-	-	-	-	-	-	-	-	-
2.801	-	-	-	-	-	-	-	-	-	-
2.699	-	-	-	-	-	-	-	-	-	-
2.687	-	-	-	-	-	-	-	-	-	-
2.875	-	-	-	-	-	-	-	-	-	-
3.421	-	-	-	-	-	-	-	-	-	-
2.824	-	-	-	-	-	-	-	-	-	-
2.604	-	-	-	-	-	-	-	-	-	-
3.141	-	-	-	-	-	-	-	-	-	-
3.43	-	-	-	-	-	-	-	-	-	-
3.155	-	-	-	-	-	-	-	-	-	-
3.402	-	-	-	-	-	-	-	-	-	-
3.401	-	-	-	-	-	-	-	-	-	-
3.398	-	-	-	-	-	-	-	-	-	-
3.403	-	-	-	-	-	-	-	-	-	-
3.244	-	-	-	-	-	-	-	-	-	-
3.251	-	-	-	-	-	-	-	-	-	-
3.364	-	-	-	-	-	-	-	-	-	-
3.365	-	-	-	-	-	-	-	-	-	-
3.277	-	-	-	-	-	-	-	-	-	-
3.061	-	-	-	-	-	-	-	-	-	-
3.442	-	-	-	-	-	-	-	-	-	-
3.358	-	-	-	-	-	-	-	-	-	-

Continues on next page

Table S10 – *Continued from previous page*

C-H	H-O	C-O	O-O	C-C	H-N	C-N	N-O	H-Z	C-Zn	O-Zn
3.203	-	-	-	-	-	-	-	-	-	-
3.216	-	-	-	-	-	-	-	-	-	-
3.361	-	-	-	-	-	-	-	-	-	-
3.223	-	-	-	-	-	-	-	-	-	-
Frame number : 10000										
3.484	2.769	-	-	3.163	3.421	-	-	-	-	-
3.465	3.195	-	-	-	-	-	-	-	-	-
3.024	2.909	-	-	-	-	-	-	-	-	-
3.279	3.407	-	-	-	-	-	-	-	-	-
2.84	3.099	-	-	-	-	-	-	-	-	-
3.317	-	-	-	-	-	-	-	-	-	-
3.444	-	-	-	-	-	-	-	-	-	-
3.185	-	-	-	-	-	-	-	-	-	-
2.699	-	-	-	-	-	-	-	-	-	-
2.684	-	-	-	-	-	-	-	-	-	-
3.128	-	-	-	-	-	-	-	-	-	-
3.453	-	-	-	-	-	-	-	-	-	-
3.229	-	-	-	-	-	-	-	-	-	-
3.225	-	-	-	-	-	-	-	-	-	-
3.286	-	-	-	-	-	-	-	-	-	-
3.354	-	-	-	-	-	-	-	-	-	-
2.621	-	-	-	-	-	-	-	-	-	-
2.957	-	-	-	-	-	-	-	-	-	-
3.397	-	-	-	-	-	-	-	-	-	-

Continues on next page

Table S10 – *Continued from previous page*

C-H	H-O	C-O	O-O	C-C	H-N	C-N	N-O	H-Z	C-Zn	O-Zn
2.768	-	-	-	-	-	-	-	-	-	-
2.287	-	-	-	-	-	-	-	-	-	-
3.29	-	-	-	-	-	-	-	-	-	-
3.181	-	-	-	-	-	-	-	-	-	-
2.933	-	-	-	-	-	-	-	-	-	-
2.936	-	-	-	-	-	-	-	-	-	-
3.086	-	-	-	-	-	-	-	-	-	-
3.091	-	-	-	-	-	-	-	-	-	-
3.373	-	-	-	-	-	-	-	-	-	-
3.021	-	-	-	-	-	-	-	-	-	-
3.331	-	-	-	-	-	-	-	-	-	-
3.294	-	-	-	-	-	-	-	-	-	-
3.397	-	-	-	-	-	-	-	-	-	-
3.048	-	-	-	-	-	-	-	-	-	-
3.372	-	-	-	-	-	-	-	-	-	-
3.016	-	-	-	-	-	-	-	-	-	-
3.185	-	-	-	-	-	-	-	-	-	-
3.47	-	-	-	-	-	-	-	-	-	-
3.21	-	-	-	-	-	-	-	-	-	-
3.237	-	-	-	-	-	-	-	-	-	-
3.172	-	-	-	-	-	-	-	-	-	-
3.354	-	-	-	-	-	-	-	-	-	-
3.291	-	-	-	-	-	-	-	-	-	-
3.208	-	-	-	-	-	-	-	-	-	-

Continues on next page

Table S10 – *Continued from previous page*

C-H	H-O	C-O	O-O	C-C	H-N	C-N	N-O	H-Z	C-Zn	O-Zn
3.492	-	-	-	-	-	-	-	-	-	-
2.992	-	-	-	-	-	-	-	-	-	-
3.095	-	-	-	-	-	-	-	-	-	-
3.25	-	-	-	-	-	-	-	-	-	-
3.498	-	-	-	-	-	-	-	-	-	-
3.293	-	-	-	-	-	-	-	-	-	-
3.433	-	-	-	-	-	-	-	-	-	-
3.396	-	-	-	-	-	-	-	-	-	-
3.426	-	-	-	-	-	-	-	-	-	-
3.041	-	-	-	-	-	-	-	-	-	-
3.47	-	-	-	-	-	-	-	-	-	-
Frame number : 20000										
3.167	-	-	-	3.473	-	-	-	-	-	-
3.307	-	-	-	3.458	-	-	-	-	-	-
2.934	-	-	-	3.48	-	-	-	-	-	-
2.858	-	-	-	3.29	-	-	-	-	-	-
2.503	-	-	-	3.037	-	-	-	-	-	-
3.179	-	-	-	3.424	-	-	-	-	-	-
2.457	-	-	-	3.107	-	-	-	-	-	-
3.497	-	-	-	2.99	-	-	-	-	-	-
3.329	-	-	-	3.283	-	-	-	-	-	-
3.498	-	-	-	-	-	-	-	-	-	-
3.267	-	-	-	-	-	-	-	-	-	-
2.971	-	-	-	-	-	-	-	-	-	-

Continues on next page

Table S10 – *Continued from previous page*

C-H	H-O	C-O	O-O	C-C	H-N	C-N	N-O	H-Z	C-Zn	O-Zn
3.434	-	-	-	-	-	-	-	-	-	-
3.243	-	-	-	-	-	-	-	-	-	-
3.042	-	-	-	-	-	-	-	-	-	-
3.407	-	-	-	-	-	-	-	-	-	-
3.071	-	-	-	-	-	-	-	-	-	-
3.398	-	-	-	-	-	-	-	-	-	-
3.401	-	-	-	-	-	-	-	-	-	-
3.292	-	-	-	-	-	-	-	-	-	-
3.002	-	-	-	-	-	-	-	-	-	-
3.026	-	-	-	-	-	-	-	-	-	-
2.394	-	-	-	-	-	-	-	-	-	-
2.539	-	-	-	-	-	-	-	-	-	-
3.304	-	-	-	-	-	-	-	-	-	-
2.342	-	-	-	-	-	-	-	-	-	-
2.636	-	-	-	-	-	-	-	-	-	-
3.424	-	-	-	-	-	-	-	-	-	-
3.328	-	-	-	-	-	-	-	-	-	-
3.401	-	-	-	-	-	-	-	-	-	-
2.401	-	-	-	-	-	-	-	-	-	-
2.963	-	-	-	-	-	-	-	-	-	-
3.298	-	-	-	-	-	-	-	-	-	-
2.975	-	-	-	-	-	-	-	-	-	-
3.433	-	-	-	-	-	-	-	-	-	-
2.691	-	-	-	-	-	-	-	-	-	-

Continues on next page

Table S10 – *Continued from previous page*

C-H	H-O	C-O	O-O	C-C	H-N	C-N	N-O	H-Z	C-Zn	O-Zn
2.971	-	-	-	-	-	-	-	-	-	-
3.406	-	-	-	-	-	-	-	-	-	-
3.291	-	-	-	-	-	-	-	-	-	-
3.127	-	-	-	-	-	-	-	-	-	-
3.425	-	-	-	-	-	-	-	-	-	-
2.956	-	-	-	-	-	-	-	-	-	-
3.336	-	-	-	-	-	-	-	-	-	-
2.687	-	-	-	-	-	-	-	-	-	-
3.133	-	-	-	-	-	-	-	-	-	-
3.153	-	-	-	-	-	-	-	-	-	-
3.159	-	-	-	-	-	-	-	-	-	-
3.28	-	-	-	-	-	-	-	-	-	-
3.274	-	-	-	-	-	-	-	-	-	-
3.198	-	-	-	-	-	-	-	-	-	-
3.228	-	-	-	-	-	-	-	-	-	-
3.127	-	-	-	-	-	-	-	-	-	-
Frame number : 30000										
3.072	3.294	-	-	3.461	-	-	-	-	-	-
3.148	3.382	-	-	3.472	-	-	-	-	-	-
2.601	3.039	-	-	-	-	-	-	-	-	-
3.038	-	-	-	-	-	-	-	-	-	-
3.305	-	-	-	-	-	-	-	-	-	-
3.27	-	-	-	-	-	-	-	-	-	-
2.9	-	-	-	-	-	-	-	-	-	-

Continues on next page

Table S10 – *Continued from previous page*

C-H	H-O	C-O	O-O	C-C	H-N	C-N	N-O	H-Z	C-Zn	O-Zn
3.235	-	-	-	-	-	-	-	-	-	-
3.318	-	-	-	-	-	-	-	-	-	-
3.457	-	-	-	-	-	-	-	-	-	-
2.636	-	-	-	-	-	-	-	-	-	-
2.443	-	-	-	-	-	-	-	-	-	-
2.899	-	-	-	-	-	-	-	-	-	-
3.195	-	-	-	-	-	-	-	-	-	-
3.374	-	-	-	-	-	-	-	-	-	-
3.489	-	-	-	-	-	-	-	-	-	-
3.281	-	-	-	-	-	-	-	-	-	-
2.999	-	-	-	-	-	-	-	-	-	-
2.976	-	-	-	-	-	-	-	-	-	-
2.966	-	-	-	-	-	-	-	-	-	-
3.01	-	-	-	-	-	-	-	-	-	-
3.432	-	-	-	-	-	-	-	-	-	-
3.117	-	-	-	-	-	-	-	-	-	-
3.323	-	-	-	-	-	-	-	-	-	-
3.469	-	-	-	-	-	-	-	-	-	-
3.473	-	-	-	-	-	-	-	-	-	-
3.452	-	-	-	-	-	-	-	-	-	-
2.955	-	-	-	-	-	-	-	-	-	-
3.412	-	-	-	-	-	-	-	-	-	-
3.228	-	-	-	-	-	-	-	-	-	-
3.123	-	-	-	-	-	-	-	-	-	-

Continues on next page

Table S10 – *Continued from previous page*

C-H	H-O	C-O	O-O	C-C	H-N	C-N	N-O	H-Z	C-Zn	O-Zn
3.33	-	-	-	-	-	-	-	-	-	-
3.019	-	-	-	-	-	-	-	-	-	-
2.809	-	-	-	-	-	-	-	-	-	-
3.196	-	-	-	-	-	-	-	-	-	-
2.846	-	-	-	-	-	-	-	-	-	-
3.205	-	-	-	-	-	-	-	-	-	-
Frame number : 40000										
3.241	3.234	3.391	-	3.347	-	-	-	-	-	-
3.333	2.459	-	-	3.309	-	-	-	-	-	-
3.174	3.381	-	-	3.473	-	-	-	-	-	-
3.456	-	-	-	3.467	-	-	-	-	-	-
3.0	-	-	-	3.312	-	-	-	-	-	-
3.042	-	-	-	3.466	-	-	-	-	-	-
3.436	-	-	-	-	-	-	-	-	-	-
2.989	-	-	-	-	-	-	-	-	-	-
3.36	-	-	-	-	-	-	-	-	-	-
3.307	-	-	-	-	-	-	-	-	-	-
3.334	-	-	-	-	-	-	-	-	-	-
3.483	-	-	-	-	-	-	-	-	-	-
3.488	-	-	-	-	-	-	-	-	-	-
2.614	-	-	-	-	-	-	-	-	-	-
3.48	-	-	-	-	-	-	-	-	-	-
2.876	-	-	-	-	-	-	-	-	-	-
3.146	-	-	-	-	-	-	-	-	-	-

Continues on next page

Table S10 – *Continued from previous page*

C-H	H-O	C-O	O-O	C-C	H-N	C-N	N-O	H-Z	C-Zn	O-Zn
3.188	-	-	-	-	-	-	-	-	-	-
3.056	-	-	-	-	-	-	-	-	-	-
3.362	-	-	-	-	-	-	-	-	-	-
3.474	-	-	-	-	-	-	-	-	-	-
3.386	-	-	-	-	-	-	-	-	-	-
3.414	-	-	-	-	-	-	-	-	-	-
2.848	-	-	-	-	-	-	-	-	-	-
2.316	-	-	-	-	-	-	-	-	-	-
2.4	-	-	-	-	-	-	-	-	-	-
3.168	-	-	-	-	-	-	-	-	-	-
3.456	-	-	-	-	-	-	-	-	-	-
2.971	-	-	-	-	-	-	-	-	-	-
3.375	-	-	-	-	-	-	-	-	-	-
3.365	-	-	-	-	-	-	-	-	-	-
3.163	-	-	-	-	-	-	-	-	-	-
2.546	-	-	-	-	-	-	-	-	-	-
3.069	-	-	-	-	-	-	-	-	-	-
3.43	-	-	-	-	-	-	-	-	-	-
2.985	-	-	-	-	-	-	-	-	-	-
3.3	-	-	-	-	-	-	-	-	-	-
2.927	-	-	-	-	-	-	-	-	-	-
3.425	-	-	-	-	-	-	-	-	-	-
3.424	-	-	-	-	-	-	-	-	-	-
3.101	-	-	-	-	-	-	-	-	-	-

Continues on next page

Table S10 – *Continued from previous page*

C-H	H-O	C-O	O-O	C-C	H-N	C-N	N-O	H-Z	C-Zn	O-Zn
3.076	-	-	-	-	-	-	-	-	-	-
3.378	-	-	-	-	-	-	-	-	-	-
3.121	-	-	-	-	-	-	-	-	-	-
3.057	-	-	-	-	-	-	-	-	-	-
Frame number : 50000										
3.087	3.116	3.251	-	3.482	-	-	-	-	-	-
3.471	2.57	-	-	3.402	-	-	-	-	-	-
3.373	3.385	-	-	3.074	-	-	-	-	-	-
3.139	3.384	-	-	2.866	-	-	-	-	-	-
3.359	-	-	-	3.31	-	-	-	-	-	-
2.859	-	-	-	3.247	-	-	-	-	-	-
2.793	-	-	-	3.49	-	-	-	-	-	-
2.682	-	-	-	3.121	-	-	-	-	-	-
3.327	-	-	-	3.187	-	-	-	-	-	-
2.836	-	-	-	3.459	-	-	-	-	-	-
2.913	-	-	-	3.104	-	-	-	-	-	-
3.253	-	-	-	-	-	-	-	-	-	-
3.418	-	-	-	-	-	-	-	-	-	-
3.129	-	-	-	-	-	-	-	-	-	-
3.166	-	-	-	-	-	-	-	-	-	-
2.418	-	-	-	-	-	-	-	-	-	-
3.377	-	-	-	-	-	-	-	-	-	-
3.038	-	-	-	-	-	-	-	-	-	-
3.047	-	-	-	-	-	-	-	-	-	-

Continues on next page

Table S10 – *Continued from previous page*

C-H	H-O	C-O	O-O	C-C	H-N	C-N	N-O	H-Z	C-Zn	O-Zn
3.253	-	-	-	-	-	-	-	-	-	-
3.297	-	-	-	-	-	-	-	-	-	-
3.402	-	-	-	-	-	-	-	-	-	-
3.353	-	-	-	-	-	-	-	-	-	-
3.143	-	-	-	-	-	-	-	-	-	-
3.359	-	-	-	-	-	-	-	-	-	-
2.488	-	-	-	-	-	-	-	-	-	-
3.382	-	-	-	-	-	-	-	-	-	-
3.343	-	-	-	-	-	-	-	-	-	-
3.407	-	-	-	-	-	-	-	-	-	-
3.016	-	-	-	-	-	-	-	-	-	-
3.266	-	-	-	-	-	-	-	-	-	-
3.315	-	-	-	-	-	-	-	-	-	-
3.423	-	-	-	-	-	-	-	-	-	-
3.103	-	-	-	-	-	-	-	-	-	-
3.27	-	-	-	-	-	-	-	-	-	-
3.424	-	-	-	-	-	-	-	-	-	-
2.916	-	-	-	-	-	-	-	-	-	-
3.27	-	-	-	-	-	-	-	-	-	-
3.053	-	-	-	-	-	-	-	-	-	-
3.472	-	-	-	-	-	-	-	-	-	-
3.482	-	-	-	-	-	-	-	-	-	-
3.206	-	-	-	-	-	-	-	-	-	-
3.359	-	-	-	-	-	-	-	-	-	-

Continues on next page

Table S10 – *Continued from previous page*

C-H	H-O	C-O	O-O	C-C	H-N	C-N	N-O	H-Z	C-Zn	O-Zn
3.388	-	-	-	-	-	-	-	-	-	-
3.415	-	-	-	-	-	-	-	-	-	-
2.686	-	-	-	-	-	-	-	-	-	-
3.458	-	-	-	-	-	-	-	-	-	-
3.46	-	-	-	-	-	-	-	-	-	-
3.241	-	-	-	-	-	-	-	-	-	-
3.315	-	-	-	-	-	-	-	-	-	-
3.421	-	-	-	-	-	-	-	-	-	-
3.261	-	-	-	-	-	-	-	-	-	-
Frame number : 60000										
2.732	3.097	3.429	-	3.455	-	-	-	-	-	-
3.164	3.365	-	-	3.348	-	-	-	-	-	-
3.38	3.188	-	-	2.983	-	-	-	-	-	-
2.714	3.412	-	-	3.452	-	-	-	-	-	-
3.074	3.046	-	-	3.28	-	-	-	-	-	-
3.384	2.469	-	-	3.195	-	-	-	-	-	-
3.497	-	-	-	3.335	-	-	-	-	-	-
3.062	-	-	-	-	-	-	-	-	-	-
3.139	-	-	-	-	-	-	-	-	-	-
2.799	-	-	-	-	-	-	-	-	-	-
3.359	-	-	-	-	-	-	-	-	-	-
3.243	-	-	-	-	-	-	-	-	-	-
2.696	-	-	-	-	-	-	-	-	-	-
3.395	-	-	-	-	-	-	-	-	-	-

Continues on next page

Table S10 – *Continued from previous page*

C-H	H-O	C-O	O-O	C-C	H-N	C-N	N-O	H-Z	C-Zn	O-Zn
2.8	-	-	-	-	-	-	-	-	-	-
3.332	-	-	-	-	-	-	-	-	-	-
3.42	-	-	-	-	-	-	-	-	-	-
2.719	-	-	-	-	-	-	-	-	-	-
3.491	-	-	-	-	-	-	-	-	-	-
2.659	-	-	-	-	-	-	-	-	-	-
2.68	-	-	-	-	-	-	-	-	-	-
3.348	-	-	-	-	-	-	-	-	-	-
3.094	-	-	-	-	-	-	-	-	-	-
3.074	-	-	-	-	-	-	-	-	-	-
3.196	-	-	-	-	-	-	-	-	-	-
3.29	-	-	-	-	-	-	-	-	-	-
3.147	-	-	-	-	-	-	-	-	-	-
2.995	-	-	-	-	-	-	-	-	-	-
3.141	-	-	-	-	-	-	-	-	-	-
2.812	-	-	-	-	-	-	-	-	-	-
2.554	-	-	-	-	-	-	-	-	-	-
3.048	-	-	-	-	-	-	-	-	-	-
3.332	-	-	-	-	-	-	-	-	-	-
3.061	-	-	-	-	-	-	-	-	-	-
3.384	-	-	-	-	-	-	-	-	-	-
3.242	-	-	-	-	-	-	-	-	-	-
3.017	-	-	-	-	-	-	-	-	-	-
Frame number : 70000										

Continues on next page

Table S10 – *Continued from previous page*

C-H	H-O	C-O	O-O	C-C	H-N	C-N	N-O	H-Z	C-Zn	O-Zn
2.879	3.463	-	-	3.395	-	-	-	-	-	-
3.079	2.784	-	-	3.459	-	-	-	-	-	-
3.269	3.344	-	-	3.03	-	-	-	-	-	-
3.416	3.319	-	-	3.23	-	-	-	-	-	-
3.074	-	-	-	3.155	-	-	-	-	-	-
3.453	-	-	-	-	-	-	-	-	-	-
3.382	-	-	-	-	-	-	-	-	-	-
3.382	-	-	-	-	-	-	-	-	-	-
2.53	-	-	-	-	-	-	-	-	-	-
2.643	-	-	-	-	-	-	-	-	-	-
2.087	-	-	-	-	-	-	-	-	-	-
2.534	-	-	-	-	-	-	-	-	-	-
3.115	-	-	-	-	-	-	-	-	-	-
2.51	-	-	-	-	-	-	-	-	-	-
3.49	-	-	-	-	-	-	-	-	-	-
2.832	-	-	-	-	-	-	-	-	-	-
3.455	-	-	-	-	-	-	-	-	-	-
3.269	-	-	-	-	-	-	-	-	-	-
3.293	-	-	-	-	-	-	-	-	-	-
3.104	-	-	-	-	-	-	-	-	-	-
3.408	-	-	-	-	-	-	-	-	-	-
3.258	-	-	-	-	-	-	-	-	-	-
2.9	-	-	-	-	-	-	-	-	-	-
3.46	-	-	-	-	-	-	-	-	-	-

Continues on next page

Table S10 – *Continued from previous page*

C-H	H-O	C-O	O-O	C-C	H-N	C-N	N-O	H-Z	C-Zn	O-Zn
3.228	-	-	-	-	-	-	-	-	-	-
3.402	-	-	-	-	-	-	-	-	-	-
2.993	-	-	-	-	-	-	-	-	-	-
3.01	-	-	-	-	-	-	-	-	-	-
3.301	-	-	-	-	-	-	-	-	-	-
3.428	-	-	-	-	-	-	-	-	-	-
3.001	-	-	-	-	-	-	-	-	-	-
2.759	-	-	-	-	-	-	-	-	-	-
2.755	-	-	-	-	-	-	-	-	-	-
2.663	-	-	-	-	-	-	-	-	-	-
2.779	-	-	-	-	-	-	-	-	-	-
3.073	-	-	-	-	-	-	-	-	-	-
3.469	-	-	-	-	-	-	-	-	-	-
3.384	-	-	-	-	-	-	-	-	-	-
3.078	-	-	-	-	-	-	-	-	-	-
3.097	-	-	-	-	-	-	-	-	-	-
2.932	-	-	-	-	-	-	-	-	-	-
3.083	-	-	-	-	-	-	-	-	-	-
3.394	-	-	-	-	-	-	-	-	-	-
2.926	-	-	-	-	-	-	-	-	-	-
3.331	-	-	-	-	-	-	-	-	-	-
3.193	-	-	-	-	-	-	-	-	-	-
3.345	-	-	-	-	-	-	-	-	-	-
3.066	-	-	-	-	-	-	-	-	-	-

Continues on next page

Table S10 – *Continued from previous page*

C-H	H-O	C-O	O-O	C-C	H-N	C-N	N-O	H-Z	C-Zn	O-Zn
2.814	-	-	-	-	-	-	-	-	-	-
2.666	-	-	-	-	-	-	-	-	-	-
3.159	-	-	-	-	-	-	-	-	-	-
Frame number : 80000										
3.433	2.908	-	-	3.312	-	-	-	-	-	-
3.492	-	-	-	3.442	-	-	-	-	-	-
2.807	-	-	-	3.342	-	-	-	-	-	-
3.383	-	-	-	3.284	-	-	-	-	-	-
2.883	-	-	-	3.318	-	-	-	-	-	-
2.684	-	-	-	3.462	-	-	-	-	-	-
2.734	-	-	-	3.332	-	-	-	-	-	-
3.312	-	-	-	2.903	-	-	-	-	-	-
2.679	-	-	-	3.371	-	-	-	-	-	-
3.35	-	-	-	3.059	-	-	-	-	-	-
3.095	-	-	-	-	-	-	-	-	-	-
3.315	-	-	-	-	-	-	-	-	-	-
2.965	-	-	-	-	-	-	-	-	-	-
3.463	-	-	-	-	-	-	-	-	-	-
3.084	-	-	-	-	-	-	-	-	-	-
2.628	-	-	-	-	-	-	-	-	-	-
3.492	-	-	-	-	-	-	-	-	-	-
3.221	-	-	-	-	-	-	-	-	-	-
3.406	-	-	-	-	-	-	-	-	-	-
2.953	-	-	-	-	-	-	-	-	-	-

Continues on next page

Table S10 – *Continued from previous page*

C-H	H-O	C-O	O-O	C-C	H-N	C-N	N-O	H-Z	C-Zn	O-Zn
3.326	-	-	-	-	-	-	-	-	-	-
3.417	-	-	-	-	-	-	-	-	-	-
2.703	-	-	-	-	-	-	-	-	-	-
2.785	-	-	-	-	-	-	-	-	-	-
3.125	-	-	-	-	-	-	-	-	-	-
2.468	-	-	-	-	-	-	-	-	-	-
2.589	-	-	-	-	-	-	-	-	-	-
3.475	-	-	-	-	-	-	-	-	-	-
3.086	-	-	-	-	-	-	-	-	-	-
3.361	-	-	-	-	-	-	-	-	-	-
3.39	-	-	-	-	-	-	-	-	-	-
2.987	-	-	-	-	-	-	-	-	-	-
2.975	-	-	-	-	-	-	-	-	-	-
3.354	-	-	-	-	-	-	-	-	-	-
3.352	-	-	-	-	-	-	-	-	-	-
3.199	-	-	-	-	-	-	-	-	-	-
3.112	-	-	-	-	-	-	-	-	-	-
2.713	-	-	-	-	-	-	-	-	-	-
3.354	-	-	-	-	-	-	-	-	-	-
3.46	-	-	-	-	-	-	-	-	-	-
3.186	-	-	-	-	-	-	-	-	-	-
2.926	-	-	-	-	-	-	-	-	-	-
3.281	-	-	-	-	-	-	-	-	-	-
2.559	-	-	-	-	-	-	-	-	-	-

Continues on next page

Table S10 – *Continued from previous page*

C-H	H-O	C-O	O-O	C-C	H-N	C-N	N-O	H-Z	C-Zn	O-Zn
3.104	-	-	-	-	-	-	-	-	-	-
2.552	-	-	-	-	-	-	-	-	-	-
3.144	-	-	-	-	-	-	-	-	-	-
3.445	-	-	-	-	-	-	-	-	-	-
3.419	-	-	-	-	-	-	-	-	-	-
2.504	-	-	-	-	-	-	-	-	-	-
1.993	-	-	-	-	-	-	-	-	-	-
2.806	-	-	-	-	-	-	-	-	-	-
3.303	-	-	-	-	-	-	-	-	-	-
3.062	-	-	-	-	-	-	-	-	-	-
3.058	-	-	-	-	-	-	-	-	-	-
3.371	-	-	-	-	-	-	-	-	-	-
Frame number : 90000										
3.362	2.414	-	-	3.463	3.191	-	-	-	-	-
3.161	3.115	-	-	3.035	2.942	-	-	-	-	-
3.364	3.396	-	-	3.18	-	-	-	-	-	-
2.804	-	-	-	3.452	-	-	-	-	-	-
3.44	-	-	-	3.325	-	-	-	-	-	-
3.254	-	-	-	3.5	-	-	-	-	-	-
3.477	-	-	-	3.173	-	-	-	-	-	-
2.514	-	-	-	2.983	-	-	-	-	-	-
2.719	-	-	-	3.422	-	-	-	-	-	-
3.479	-	-	-	3.142	-	-	-	-	-	-
2.568	-	-	-	3.074	-	-	-	-	-	-

Continues on next page

Table S10 – *Continued from previous page*

C-H	H-O	C-O	O-O	C-C	H-N	C-N	N-O	H-Z	C-Zn	O-Zn
2.825	-	-	-	-	-	-	-	-	-	-
3.359	-	-	-	-	-	-	-	-	-	-
3.499	-	-	-	-	-	-	-	-	-	-
2.98	-	-	-	-	-	-	-	-	-	-
3.179	-	-	-	-	-	-	-	-	-	-
3.36	-	-	-	-	-	-	-	-	-	-
2.745	-	-	-	-	-	-	-	-	-	-
2.847	-	-	-	-	-	-	-	-	-	-
2.395	-	-	-	-	-	-	-	-	-	-
3.436	-	-	-	-	-	-	-	-	-	-
2.907	-	-	-	-	-	-	-	-	-	-
3.342	-	-	-	-	-	-	-	-	-	-
3.348	-	-	-	-	-	-	-	-	-	-
2.443	-	-	-	-	-	-	-	-	-	-
2.155	-	-	-	-	-	-	-	-	-	-
3.336	-	-	-	-	-	-	-	-	-	-
2.939	-	-	-	-	-	-	-	-	-	-
3.178	-	-	-	-	-	-	-	-	-	-
2.787	-	-	-	-	-	-	-	-	-	-
3.148	-	-	-	-	-	-	-	-	-	-
3.086	-	-	-	-	-	-	-	-	-	-
3.128	-	-	-	-	-	-	-	-	-	-
2.535	-	-	-	-	-	-	-	-	-	-
2.216	-	-	-	-	-	-	-	-	-	-

Continues on next page

Table S10 – *Continued from previous page*

C-H	H-O	C-O	O-O	C-C	H-N	C-N	N-O	H-Z	C-Zn	O-Zn
2.806	-	-	-	-	-	-	-	-	-	-
2.835	-	-	-	-	-	-	-	-	-	-
2.761	-	-	-	-	-	-	-	-	-	-
3.421	-	-	-	-	-	-	-	-	-	-
2.768	-	-	-	-	-	-	-	-	-	-
3.405	-	-	-	-	-	-	-	-	-	-
3.392	-	-	-	-	-	-	-	-	-	-
3.439	-	-	-	-	-	-	-	-	-	-
3.088	-	-	-	-	-	-	-	-	-	-
3.2	-	-	-	-	-	-	-	-	-	-
3.45	-	-	-	-	-	-	-	-	-	-
3.457	-	-	-	-	-	-	-	-	-	-
3.485	-	-	-	-	-	-	-	-	-	-
3.099	-	-	-	-	-	-	-	-	-	-
3.394	-	-	-	-	-	-	-	-	-	-
3.331	-	-	-	-	-	-	-	-	-	-
3.164	-	-	-	-	-	-	-	-	-	-
3.287	-	-	-	-	-	-	-	-	-	-
3.386	-	-	-	-	-	-	-	-	-	-
3.472	-	-	-	-	-	-	-	-	-	-
3.489	-	-	-	-	-	-	-	-	-	-
3.191	-	-	-	-	-	-	-	-	-	-
3.242	-	-	-	-	-	-	-	-	-	-
Frame number : 100000										

Continues on next page

Table S10 – *Continued from previous page*

C-H	H-O	C-O	O-O	C-C	H-N	C-N	N-O	H-Z	C-Zn	O-Zn
2.992	3.406	-	-	3.475	-	-	-	-	-	-
3.072	3.244	-	-	3.404	-	-	-	-	-	-
3.305	-	-	-	3.335	-	-	-	-	-	-
3.449	-	-	-	3.307	-	-	-	-	-	-
2.902	-	-	-	3.05	-	-	-	-	-	-
3.278	-	-	-	3.036	-	-	-	-	-	-
3.21	-	-	-	3.26	-	-	-	-	-	-
2.895	-	-	-	3.476	-	-	-	-	-	-
2.922	-	-	-	3.413	-	-	-	-	-	-
3.399	-	-	-	3.193	-	-	-	-	-	-
2.406	-	-	-	-	-	-	-	-	-	-
3.322	-	-	-	-	-	-	-	-	-	-
2.798	-	-	-	-	-	-	-	-	-	-
3.289	-	-	-	-	-	-	-	-	-	-
3.359	-	-	-	-	-	-	-	-	-	-
3.194	-	-	-	-	-	-	-	-	-	-
3.231	-	-	-	-	-	-	-	-	-	-
3.45	-	-	-	-	-	-	-	-	-	-
3.032	-	-	-	-	-	-	-	-	-	-
3.345	-	-	-	-	-	-	-	-	-	-
3.031	-	-	-	-	-	-	-	-	-	-
3.433	-	-	-	-	-	-	-	-	-	-
3.057	-	-	-	-	-	-	-	-	-	-
2.999	-	-	-	-	-	-	-	-	-	-

Continues on next page

Table S10 – *Continued from previous page*

C-H	H-O	C-O	O-O	C-C	H-N	C-N	N-O	H-Z	C-Zn	O-Zn
3.135	-	-	-	-	-	-	-	-	-	-
3.288	-	-	-	-	-	-	-	-	-	-
2.955	-	-	-	-	-	-	-	-	-	-
2.517	-	-	-	-	-	-	-	-	-	-
3.279	-	-	-	-	-	-	-	-	-	-
3.163	-	-	-	-	-	-	-	-	-	-
3.406	-	-	-	-	-	-	-	-	-	-
2.542	-	-	-	-	-	-	-	-	-	-
3.204	-	-	-	-	-	-	-	-	-	-
3.271	-	-	-	-	-	-	-	-	-	-
3.481	-	-	-	-	-	-	-	-	-	-
2.662	-	-	-	-	-	-	-	-	-	-
3.409	-	-	-	-	-	-	-	-	-	-
3.089	-	-	-	-	-	-	-	-	-	-
2.449	-	-	-	-	-	-	-	-	-	-
3.183	-	-	-	-	-	-	-	-	-	-
3.405	-	-	-	-	-	-	-	-	-	-
3.23	-	-	-	-	-	-	-	-	-	-
2.805	-	-	-	-	-	-	-	-	-	-
3.063	-	-	-	-	-	-	-	-	-	-
3.089	-	-	-	-	-	-	-	-	-	-
3.166	-	-	-	-	-	-	-	-	-	-
3.223	-	-	-	-	-	-	-	-	-	-
2.391	-	-	-	-	-	-	-	-	-	-

Continues on next page

Table S10 – *Continued from previous page*

C-H	H-O	C-O	O-O	C-C	H-N	C-N	N-O	H-Z	C-Zn	O-Zn
2.558	-	-	-	-	-	-	-	-	-	-
3.476	-	-	-	-	-	-	-	-	-	-
3.15	-	-	-	-	-	-	-	-	-	-
2.718	-	-	-	-	-	-	-	-	-	-
2.848	-	-	-	-	-	-	-	-	-	-
2.915	-	-	-	-	-	-	-	-	-	-

Table S11 Intermolecular distances (r < 3.5 Å) for pore ZnPW⊃-3-Styrene interactions computed for every 10000 molecular dynamic frames.

C-H	H-O	C-O	O-O	C-C	H-N	C-N	N-O	H-Z	C-Zn	O-Zn
Frame number : 0										
3.082	2.856	-	-	3.272	3.394	-	-	-	-	-
3.29	3.371	-	-	3.14	-	-	-	-	-	-
3.446	2.807	-	-	3.418	-	-	-	-	-	-
2.655	3.415	-	-	3.433	-	-	-	-	-	-
3.266	3.057	-	-	3.399	-	-	-	-	-	-
2.953	3.248	-	-	3.477	-	-	-	-	-	-
3.327	3.477	-	-	3.454	-	-	-	-	-	-
3.309	3.012	-	-	3.181	-	-	-	-	-	-
3.239	3.446	-	-	3.215	-	-	-	-	-	-
2.883	3.456	-	-	3.172	-	-	-	-	-	-
3.207	-	-	-	3.201	-	-	-	-	-	-

Continues on next page

Table S11 – *Continued from previous page*

C-H	H-O	C-O	O-O	C-C	H-N	C-N	N-O	H-Z	C-Zn	O-Zn
2.859	-	-	-	3.322	-	-	-	-	-	-
2.853	-	-	-	3.345	-	-	-	-	-	-
3.073	-	-	-	3.442	-	-	-	-	-	-
3.359	-	-	-	3.091	-	-	-	-	-	-
3.466	-	-	-	3.399	-	-	-	-	-	-
2.833	-	-	-	3.294	-	-	-	-	-	-
3.499	-	-	-	3.395	-	-	-	-	-	-
2.976	-	-	-	2.948	-	-	-	-	-	-
3.461	-	-	-	3.061	-	-	-	-	-	-
3.335	-	-	-	3.402	-	-	-	-	-	-
3.347	-	-	-	3.471	-	-	-	-	-	-
2.605	-	-	-	-	-	-	-	-	-	-
3.021	-	-	-	-	-	-	-	-	-	-
3.465	-	-	-	-	-	-	-	-	-	-
3.369	-	-	-	-	-	-	-	-	-	-
2.957	-	-	-	-	-	-	-	-	-	-
3.0	-	-	-	-	-	-	-	-	-	-
3.264	-	-	-	-	-	-	-	-	-	-
3.192	-	-	-	-	-	-	-	-	-	-
2.781	-	-	-	-	-	-	-	-	-	-
2.377	-	-	-	-	-	-	-	-	-	-
2.69	-	-	-	-	-	-	-	-	-	-
3.28	-	-	-	-	-	-	-	-	-	-
3.178	-	-	-	-	-	-	-	-	-	-

Continues on next page

Table S11 – *Continued from previous page*

C-H	H-O	C-O	O-O	C-C	H-N	C-N	N-O	H-Z	C-Zn	O-Zn
3.498	-	-	-	-	-	-	-	-	-	-
3.137	-	-	-	-	-	-	-	-	-	-
3.189	-	-	-	-	-	-	-	-	-	-
3.411	-	-	-	-	-	-	-	-	-	-
3.122	-	-	-	-	-	-	-	-	-	-
2.906	-	-	-	-	-	-	-	-	-	-
2.894	-	-	-	-	-	-	-	-	-	-
3.299	-	-	-	-	-	-	-	-	-	-
3.133	-	-	-	-	-	-	-	-	-	-
2.798	-	-	-	-	-	-	-	-	-	-
2.798	-	-	-	-	-	-	-	-	-	-
3.275	-	-	-	-	-	-	-	-	-	-
3.368	-	-	-	-	-	-	-	-	-	-
3.202	-	-	-	-	-	-	-	-	-	-
3.394	-	-	-	-	-	-	-	-	-	-
2.679	-	-	-	-	-	-	-	-	-	-
3.397	-	-	-	-	-	-	-	-	-	-
3.485	-	-	-	-	-	-	-	-	-	-
3.403	-	-	-	-	-	-	-	-	-	-
3.24	-	-	-	-	-	-	-	-	-	-
3.144	-	-	-	-	-	-	-	-	-	-
2.991	-	-	-	-	-	-	-	-	-	-
2.817	-	-	-	-	-	-	-	-	-	-
2.894	-	-	-	-	-	-	-	-	-	-

Continues on next page

Table S11 – *Continued from previous page*

C-H	H-O	C-O	O-O	C-C	H-N	C-N	N-O	H-Z	C-Zn	O-Zn
2.996	-	-	-	-	-	-	-	-	-	-
2.878	-	-	-	-	-	-	-	-	-	-
2.854	-	-	-	-	-	-	-	-	-	-
3.261	-	-	-	-	-	-	-	-	-	-
3.237	-	-	-	-	-	-	-	-	-	-
2.309	-	-	-	-	-	-	-	-	-	-
3.229	-	-	-	-	-	-	-	-	-	-
3.388	-	-	-	-	-	-	-	-	-	-
3.269	-	-	-	-	-	-	-	-	-	-
3.031	-	-	-	-	-	-	-	-	-	-
3.113	-	-	-	-	-	-	-	-	-	-
3.137	-	-	-	-	-	-	-	-	-	-
2.85	-	-	-	-	-	-	-	-	-	-
3.151	-	-	-	-	-	-	-	-	-	-
3.097	-	-	-	-	-	-	-	-	-	-
3.436	-	-	-	-	-	-	-	-	-	-
3.3	-	-	-	-	-	-	-	-	-	-
3.176	-	-	-	-	-	-	-	-	-	-
2.959	-	-	-	-	-	-	-	-	-	-
3.388	-	-	-	-	-	-	-	-	-	-
3.264	-	-	-	-	-	-	-	-	-	-
3.299	-	-	-	-	-	-	-	-	-	-
2.687	-	-	-	-	-	-	-	-	-	-
2.776	-	-	-	-	-	-	-	-	-	-

Continues on next page

Table S11 – *Continued from previous page*

C-H	H-O	C-O	O-O	C-C	H-N	C-N	N-O	H-Z	C-Zn	O-Zn
3.012	-	-	-	-	-	-	-	-	-	-
3.103	-	-	-	-	-	-	-	-	-	-
3.309	-	-	-	-	-	-	-	-	-	-
2.952	-	-	-	-	-	-	-	-	-	-
2.922	-	-	-	-	-	-	-	-	-	-
2.191	-	-	-	-	-	-	-	-	-	-
2.559	-	-	-	-	-	-	-	-	-	-
3.317	-	-	-	-	-	-	-	-	-	-
3.183	-	-	-	-	-	-	-	-	-	-
2.756	-	-	-	-	-	-	-	-	-	-
3.163	-	-	-	-	-	-	-	-	-	-
3.413	-	-	-	-	-	-	-	-	-	-
2.929	-	-	-	-	-	-	-	-	-	-
3.474	-	-	-	-	-	-	-	-	-	-
3.47	-	-	-	-	-	-	-	-	-	-
3.162	-	-	-	-	-	-	-	-	-	-
2.649	-	-	-	-	-	-	-	-	-	-
3.018	-	-	-	-	-	-	-	-	-	-
3.359	-	-	-	-	-	-	-	-	-	-
3.114	-	-	-	-	-	-	-	-	-	-
3.168	-	-	-	-	-	-	-	-	-	-
2.859	-	-	-	-	-	-	-	-	-	-
3.446	-	-	-	-	-	-	-	-	-	-
3.441	-	-	-	-	-	-	-	-	-	-

Continues on next page

Table S11 – *Continued from previous page*

C-H	H-O	C-O	O-O	C-C	H-N	C-N	N-O	H-Z	C-Zn	O-Zn
Frame number : 10000										
2.821	2.913	-	-	3.21	3.397	-	-	-	-	-
2.717	3.298	-	-	3.417	-	-	-	-	-	-
3.143	2.791	-	-	2.707	-	-	-	-	-	-
2.296	3.451	-	-	3.44	-	-	-	-	-	-
3.173	2.809	-	-	3.437	-	-	-	-	-	-
2.88	3.007	-	-	2.755	-	-	-	-	-	-
3.396	3.129	-	-	3.332	-	-	-	-	-	-
3.4	2.914	-	-	3.484	-	-	-	-	-	-
3.167	3.297	-	-	3.434	-	-	-	-	-	-
3.132	3.363	-	-	3.458	-	-	-	-	-	-
2.689	3.056	-	-	3.427	-	-	-	-	-	-
3.292	3.094	-	-	3.228	-	-	-	-	-	-
2.725	-	-	-	3.366	-	-	-	-	-	-
3.15	-	-	-	3.428	-	-	-	-	-	-
2.726	-	-	-	3.451	-	-	-	-	-	-
3.328	-	-	-	3.357	-	-	-	-	-	-
3.082	-	-	-	3.247	-	-	-	-	-	-
2.844	-	-	-	3.419	-	-	-	-	-	-
2.915	-	-	-	3.303	-	-	-	-	-	-
3.393	-	-	-	3.143	-	-	-	-	-	-
2.844	-	-	-	3.431	-	-	-	-	-	-
3.219	-	-	-	3.5	-	-	-	-	-	-
3.242	-	-	-	-	-	-	-	-	-	-

Continues on next page

Table S11 – *Continued from previous page*

C-H	H-O	C-O	O-O	C-C	H-N	C-N	N-O	H-Z	C-Zn	O-Zn
3.31	-	-	-	-	-	-	-	-	-	-
3.258	-	-	-	-	-	-	-	-	-	-
2.704	-	-	-	-	-	-	-	-	-	-
3.166	-	-	-	-	-	-	-	-	-	-
3.35	-	-	-	-	-	-	-	-	-	-
3.24	-	-	-	-	-	-	-	-	-	-
3.127	-	-	-	-	-	-	-	-	-	-
3.477	-	-	-	-	-	-	-	-	-	-
3.164	-	-	-	-	-	-	-	-	-	-
3.327	-	-	-	-	-	-	-	-	-	-
3.128	-	-	-	-	-	-	-	-	-	-
2.663	-	-	-	-	-	-	-	-	-	-
2.508	-	-	-	-	-	-	-	-	-	-
3.084	-	-	-	-	-	-	-	-	-	-
3.391	-	-	-	-	-	-	-	-	-	-
3.488	-	-	-	-	-	-	-	-	-	-
3.253	-	-	-	-	-	-	-	-	-	-
2.772	-	-	-	-	-	-	-	-	-	-
3.37	-	-	-	-	-	-	-	-	-	-
3.25	-	-	-	-	-	-	-	-	-	-
2.821	-	-	-	-	-	-	-	-	-	-
3.257	-	-	-	-	-	-	-	-	-	-
3.32	-	-	-	-	-	-	-	-	-	-
2.732	-	-	-	-	-	-	-	-	-	-

Continues on next page

Table S11 – *Continued from previous page*

C-H	H-O	C-O	O-O	C-C	H-N	C-N	N-O	H-Z	C-Zn	O-Zn
2.403	-	-	-	-	-	-	-	-	-	-
2.979	-	-	-	-	-	-	-	-	-	-
2.801	-	-	-	-	-	-	-	-	-	-
3.307	-	-	-	-	-	-	-	-	-	-
3.217	-	-	-	-	-	-	-	-	-	-
3.345	-	-	-	-	-	-	-	-	-	-
3.088	-	-	-	-	-	-	-	-	-	-
2.819	-	-	-	-	-	-	-	-	-	-
3.326	-	-	-	-	-	-	-	-	-	-
3.412	-	-	-	-	-	-	-	-	-	-
3.051	-	-	-	-	-	-	-	-	-	-
2.751	-	-	-	-	-	-	-	-	-	-
2.85	-	-	-	-	-	-	-	-	-	-
3.323	-	-	-	-	-	-	-	-	-	-
3.204	-	-	-	-	-	-	-	-	-	-
3.441	-	-	-	-	-	-	-	-	-	-
3.332	-	-	-	-	-	-	-	-	-	-
3.154	-	-	-	-	-	-	-	-	-	-
2.486	-	-	-	-	-	-	-	-	-	-
3.438	-	-	-	-	-	-	-	-	-	-
3.435	-	-	-	-	-	-	-	-	-	-
3.355	-	-	-	-	-	-	-	-	-	-
3.422	-	-	-	-	-	-	-	-	-	-
3.132	-	-	-	-	-	-	-	-	-	-

Continues on next page

Table S11 – *Continued from previous page*

C-H	H-O	C-O	O-O	C-C	H-N	C-N	N-O	H-Z	C-Zn	O-Zn
3.494	-	-	-	-	-	-	-	-	-	-
2.89	-	-	-	-	-	-	-	-	-	-
3.122	-	-	-	-	-	-	-	-	-	-
3.103	-	-	-	-	-	-	-	-	-	-
3.194	-	-	-	-	-	-	-	-	-	-
3.399	-	-	-	-	-	-	-	-	-	-
3.485	-	-	-	-	-	-	-	-	-	-
3.356	-	-	-	-	-	-	-	-	-	-
2.992	-	-	-	-	-	-	-	-	-	-
3.294	-	-	-	-	-	-	-	-	-	-
3.033	-	-	-	-	-	-	-	-	-	-
2.5	-	-	-	-	-	-	-	-	-	-
3.017	-	-	-	-	-	-	-	-	-	-
3.308	-	-	-	-	-	-	-	-	-	-
2.759	-	-	-	-	-	-	-	-	-	-
3.138	-	-	-	-	-	-	-	-	-	-
3.034	-	-	-	-	-	-	-	-	-	-
2.429	-	-	-	-	-	-	-	-	-	-
3.037	-	-	-	-	-	-	-	-	-	-
3.431	-	-	-	-	-	-	-	-	-	-
3.006	-	-	-	-	-	-	-	-	-	-
3.443	-	-	-	-	-	-	-	-	-	-
2.448	-	-	-	-	-	-	-	-	-	-
2.833	-	-	-	-	-	-	-	-	-	-

Continues on next page

Table S11 – *Continued from previous page*

C-H	H-O	C-O	O-O	C-C	H-N	C-N	N-O	H-Z	C-Zn	O-Zn
3.238	-	-	-	-	-	-	-	-	-	-
3.291	-	-	-	-	-	-	-	-	-	-
2.868	-	-	-	-	-	-	-	-	-	-
3.407	-	-	-	-	-	-	-	-	-	-
3.371	-	-	-	-	-	-	-	-	-	-
3.044	-	-	-	-	-	-	-	-	-	-
3.356	-	-	-	-	-	-	-	-	-	-
3.007	-	-	-	-	-	-	-	-	-	-
2.904	-	-	-	-	-	-	-	-	-	-
3.101	-	-	-	-	-	-	-	-	-	-
2.633	-	-	-	-	-	-	-	-	-	-
2.716	-	-	-	-	-	-	-	-	-	-
3.369	-	-	-	-	-	-	-	-	-	-
3.318	-	-	-	-	-	-	-	-	-	-
Frame number : 20000										
3.165	3.241	3.327	-	3.24	3.315	-	-	3.336	-	-
3.047	2.939	3.284	-	3.161	-	-	-	-	-	-
3.088	3.467	3.263	-	3.437	-	-	-	-	-	-
2.602	2.318	3.268	-	3.367	-	-	-	-	-	-
3.451	2.806	3.245	-	3.301	-	-	-	-	-	-
2.805	3.186	-	-	3.381	-	-	-	-	-	-
3.443	3.229	-	-	3.352	-	-	-	-	-	-
2.914	2.599	-	-	3.104	-	-	-	-	-	-
3.496	2.693	-	-	3.484	-	-	-	-	-	-

Continues on next page

Table S11 – *Continued from previous page*

C-H	H-O	C-O	O-O	C-C	H-N	C-N	N-O	H-Z	C-Zn	O-Zn
2.839	3.141	-	-	2.986	-	-	-	-	-	-
2.755	3.008	-	-	3.306	-	-	-	-	-	-
3.137	2.35	-	-	3.279	-	-	-	-	-	-
3.489	3.421	-	-	3.294	-	-	-	-	-	-
3.36	-	-	-	2.782	-	-	-	-	-	-
3.314	-	-	-	3.446	-	-	-	-	-	-
3.405	-	-	-	3.471	-	-	-	-	-	-
3.134	-	-	-	3.385	-	-	-	-	-	-
3.266	-	-	-	3.446	-	-	-	-	-	-
3.309	-	-	-	3.402	-	-	-	-	-	-
3.074	-	-	-	3.406	-	-	-	-	-	-
2.592	-	-	-	3.329	-	-	-	-	-	-
3.191	-	-	-	3.303	-	-	-	-	-	-
3.432	-	-	-	-	-	-	-	-	-	-
2.786	-	-	-	-	-	-	-	-	-	-
3.031	-	-	-	-	-	-	-	-	-	-
3.233	-	-	-	-	-	-	-	-	-	-
3.405	-	-	-	-	-	-	-	-	-	-
3.354	-	-	-	-	-	-	-	-	-	-
3.349	-	-	-	-	-	-	-	-	-	-
2.932	-	-	-	-	-	-	-	-	-	-
2.684	-	-	-	-	-	-	-	-	-	-
2.861	-	-	-	-	-	-	-	-	-	-
3.453	-	-	-	-	-	-	-	-	-	-

Continues on next page

Table S11 – *Continued from previous page*

C-H	H-O	C-O	O-O	C-C	H-N	C-N	N-O	H-Z	C-Zn	O-Zn
3.156	-	-	-	-	-	-	-	-	-	-
2.706	-	-	-	-	-	-	-	-	-	-
3.314	-	-	-	-	-	-	-	-	-	-
3.264	-	-	-	-	-	-	-	-	-	-
3.144	-	-	-	-	-	-	-	-	-	-
2.472	-	-	-	-	-	-	-	-	-	-
3.152	-	-	-	-	-	-	-	-	-	-
3.379	-	-	-	-	-	-	-	-	-	-
3.258	-	-	-	-	-	-	-	-	-	-
3.232	-	-	-	-	-	-	-	-	-	-
3.183	-	-	-	-	-	-	-	-	-	-
3.191	-	-	-	-	-	-	-	-	-	-
3.097	-	-	-	-	-	-	-	-	-	-
2.791	-	-	-	-	-	-	-	-	-	-
3.249	-	-	-	-	-	-	-	-	-	-
3.262	-	-	-	-	-	-	-	-	-	-
3.316	-	-	-	-	-	-	-	-	-	-
3.33	-	-	-	-	-	-	-	-	-	-
3.415	-	-	-	-	-	-	-	-	-	-
3.389	-	-	-	-	-	-	-	-	-	-
2.773	-	-	-	-	-	-	-	-	-	-
3.374	-	-	-	-	-	-	-	-	-	-
2.957	-	-	-	-	-	-	-	-	-	-
3.281	-	-	-	-	-	-	-	-	-	-

Continues on next page

Table S11 – *Continued from previous page*

C-H	H-O	C-O	O-O	C-C	H-N	C-N	N-O	H-Z	C-Zn	O-Zn
2.528	-	-	-	-	-	-	-	-	-	-
2.513	-	-	-	-	-	-	-	-	-	-
2.868	-	-	-	-	-	-	-	-	-	-
3.154	-	-	-	-	-	-	-	-	-	-
2.757	-	-	-	-	-	-	-	-	-	-
2.562	-	-	-	-	-	-	-	-	-	-
3.417	-	-	-	-	-	-	-	-	-	-
3.375	-	-	-	-	-	-	-	-	-	-
3.498	-	-	-	-	-	-	-	-	-	-
3.092	-	-	-	-	-	-	-	-	-	-
3.39	-	-	-	-	-	-	-	-	-	-
3.066	-	-	-	-	-	-	-	-	-	-
2.212	-	-	-	-	-	-	-	-	-	-
2.851	-	-	-	-	-	-	-	-	-	-
3.401	-	-	-	-	-	-	-	-	-	-
3.18	-	-	-	-	-	-	-	-	-	-
3.295	-	-	-	-	-	-	-	-	-	-
2.568	-	-	-	-	-	-	-	-	-	-
1.803	-	-	-	-	-	-	-	-	-	-
2.67	-	-	-	-	-	-	-	-	-	-
3.469	-	-	-	-	-	-	-	-	-	-
2.808	-	-	-	-	-	-	-	-	-	-
2.792	-	-	-	-	-	-	-	-	-	-
3.287	-	-	-	-	-	-	-	-	-	-

Continues on next page

Table S11 – *Continued from previous page*

C-H	H-O	C-O	O-O	C-C	H-N	C-N	N-O	H-Z	C-Zn	O-Zn
3.155	-	-	-	-	-	-	-	-	-	-
3.026	-	-	-	-	-	-	-	-	-	-
3.39	-	-	-	-	-	-	-	-	-	-
3.25	-	-	-	-	-	-	-	-	-	-
2.248	-	-	-	-	-	-	-	-	-	-
2.673	-	-	-	-	-	-	-	-	-	-
2.919	-	-	-	-	-	-	-	-	-	-
3.434	-	-	-	-	-	-	-	-	-	-
3.168	-	-	-	-	-	-	-	-	-	-
2.797	-	-	-	-	-	-	-	-	-	-
3.376	-	-	-	-	-	-	-	-	-	-
3.452	-	-	-	-	-	-	-	-	-	-
2.706	-	-	-	-	-	-	-	-	-	-
3.192	-	-	-	-	-	-	-	-	-	-
3.105	-	-	-	-	-	-	-	-	-	-
3.121	-	-	-	-	-	-	-	-	-	-
2.886	-	-	-	-	-	-	-	-	-	-
2.375	-	-	-	-	-	-	-	-	-	-
2.739	-	-	-	-	-	-	-	-	-	-
3.108	-	-	-	-	-	-	-	-	-	-
Frame number : 30000										
2.682	3.433	3.372	-	3.305	3.282	-	-	-	-	-
3.453	3.326	-	-	2.868	3.412	-	-	-	-	-
3.47	3.46	-	-	3.493	-	-	-	-	-	-

Continues on next page

Table S11 – *Continued from previous page*

C-H	H-O	C-O	O-O	C-C	H-N	C-N	N-O	H-Z	C-Zn	O-Zn
2.984	3.295	-	-	2.989	-	-	-	-	-	-
3.297	3.141	-	-	3.048	-	-	-	-	-	-
2.311	3.441	-	-	2.829	-	-	-	-	-	-
3.072	3.059	-	-	3.045	-	-	-	-	-	-
2.92	2.477	-	-	3.274	-	-	-	-	-	-
3.461	3.044	-	-	3.34	-	-	-	-	-	-
3.406	3.393	-	-	3.221	-	-	-	-	-	-
3.232	3.011	-	-	3.299	-	-	-	-	-	-
3.418	3.273	-	-	3.217	-	-	-	-	-	-
3.461	-	-	-	3.287	-	-	-	-	-	-
3.182	-	-	-	3.253	-	-	-	-	-	-
2.776	-	-	-	3.364	-	-	-	-	-	-
3.27	-	-	-	2.944	-	-	-	-	-	-
3.234	-	-	-	3.473	-	-	-	-	-	-
3.443	-	-	-	3.21	-	-	-	-	-	-
3.356	-	-	-	3.43	-	-	-	-	-	-
2.849	-	-	-	3.418	-	-	-	-	-	-
3.095	-	-	-	3.352	-	-	-	-	-	-
3.442	-	-	-	3.404	-	-	-	-	-	-
3.475	-	-	-	-	-	-	-	-	-	-
2.789	-	-	-	-	-	-	-	-	-	-
3.385	-	-	-	-	-	-	-	-	-	-
3.001	-	-	-	-	-	-	-	-	-	-
3.284	-	-	-	-	-	-	-	-	-	-

Continues on next page

Table S11 – *Continued from previous page*

C-H	H-O	C-O	O-O	C-C	H-N	C-N	N-O	H-Z	C-Zn	O-Zn
3.104	-	-	-	-	-	-	-	-	-	-
3.329	-	-	-	-	-	-	-	-	-	-
3.4	-	-	-	-	-	-	-	-	-	-
3.197	-	-	-	-	-	-	-	-	-	-
3.295	-	-	-	-	-	-	-	-	-	-
2.779	-	-	-	-	-	-	-	-	-	-
2.477	-	-	-	-	-	-	-	-	-	-
2.468	-	-	-	-	-	-	-	-	-	-
2.896	-	-	-	-	-	-	-	-	-	-
3.244	-	-	-	-	-	-	-	-	-	-
2.877	-	-	-	-	-	-	-	-	-	-
3.16	-	-	-	-	-	-	-	-	-	-
3.483	-	-	-	-	-	-	-	-	-	-
2.568	-	-	-	-	-	-	-	-	-	-
2.895	-	-	-	-	-	-	-	-	-	-
3.371	-	-	-	-	-	-	-	-	-	-
3.439	-	-	-	-	-	-	-	-	-	-
3.013	-	-	-	-	-	-	-	-	-	-
2.572	-	-	-	-	-	-	-	-	-	-
2.292	-	-	-	-	-	-	-	-	-	-
3.277	-	-	-	-	-	-	-	-	-	-
3.172	-	-	-	-	-	-	-	-	-	-
3.455	-	-	-	-	-	-	-	-	-	-
2.44	-	-	-	-	-	-	-	-	-	-

Continues on next page

Table S11 – *Continued from previous page*

C-H	H-O	C-O	O-O	C-C	H-N	C-N	N-O	H-Z	C-Zn	O-Zn
2.588	-	-	-	-	-	-	-	-	-	-
2.979	-	-	-	-	-	-	-	-	-	-
3.418	-	-	-	-	-	-	-	-	-	-
3.274	-	-	-	-	-	-	-	-	-	-
3.321	-	-	-	-	-	-	-	-	-	-
2.696	-	-	-	-	-	-	-	-	-	-
3.454	-	-	-	-	-	-	-	-	-	-
3.485	-	-	-	-	-	-	-	-	-	-
3.432	-	-	-	-	-	-	-	-	-	-
2.945	-	-	-	-	-	-	-	-	-	-
3.321	-	-	-	-	-	-	-	-	-	-
3.072	-	-	-	-	-	-	-	-	-	-
3.381	-	-	-	-	-	-	-	-	-	-
2.898	-	-	-	-	-	-	-	-	-	-
2.884	-	-	-	-	-	-	-	-	-	-
2.7	-	-	-	-	-	-	-	-	-	-
2.799	-	-	-	-	-	-	-	-	-	-
2.972	-	-	-	-	-	-	-	-	-	-
3.001	-	-	-	-	-	-	-	-	-	-
3.219	-	-	-	-	-	-	-	-	-	-
3.314	-	-	-	-	-	-	-	-	-	-
3.461	-	-	-	-	-	-	-	-	-	-
2.88	-	-	-	-	-	-	-	-	-	-
2.228	-	-	-	-	-	-	-	-	-	-

Continues on next page

Table S11 – *Continued from previous page*

C-H	H-O	C-O	O-O	C-C	H-N	C-N	N-O	H-Z	C-Zn	O-Zn
3.177	-	-	-	-	-	-	-	-	-	-
3.477	-	-	-	-	-	-	-	-	-	-
3.398	-	-	-	-	-	-	-	-	-	-
3.312	-	-	-	-	-	-	-	-	-	-
3.292	-	-	-	-	-	-	-	-	-	-
3.366	-	-	-	-	-	-	-	-	-	-
3.354	-	-	-	-	-	-	-	-	-	-
3.352	-	-	-	-	-	-	-	-	-	-
3.466	-	-	-	-	-	-	-	-	-	-
3.491	-	-	-	-	-	-	-	-	-	-
3.4	-	-	-	-	-	-	-	-	-	-
3.186	-	-	-	-	-	-	-	-	-	-
3.385	-	-	-	-	-	-	-	-	-	-
3.312	-	-	-	-	-	-	-	-	-	-
3.393	-	-	-	-	-	-	-	-	-	-
2.991	-	-	-	-	-	-	-	-	-	-
3.421	-	-	-	-	-	-	-	-	-	-
3.131	-	-	-	-	-	-	-	-	-	-
3.324	-	-	-	-	-	-	-	-	-	-
3.166	-	-	-	-	-	-	-	-	-	-
3.464	-	-	-	-	-	-	-	-	-	-
3.04	-	-	-	-	-	-	-	-	-	-
3.469	-	-	-	-	-	-	-	-	-	-
3.244	-	-	-	-	-	-	-	-	-	-

Continues on next page

Table S11 – *Continued from previous page*

C-H	H-O	C-O	O-O	C-C	H-N	C-N	N-O	H-Z	C-Zn	O-Zn
3.427	-	-	-	-	-	-	-	-	-	-
3.026	-	-	-	-	-	-	-	-	-	-
2.897	-	-	-	-	-	-	-	-	-	-
2.44	-	-	-	-	-	-	-	-	-	-
3.127	-	-	-	-	-	-	-	-	-	-
3.05	-	-	-	-	-	-	-	-	-	-
2.747	-	-	-	-	-	-	-	-	-	-
3.325	-	-	-	-	-	-	-	-	-	-
3.476	-	-	-	-	-	-	-	-	-	-
3.424	-	-	-	-	-	-	-	-	-	-
2.287	-	-	-	-	-	-	-	-	-	-
2.879	-	-	-	-	-	-	-	-	-	-
3.372	-	-	-	-	-	-	-	-	-	-
3.219	-	-	-	-	-	-	-	-	-	-
2.827	-	-	-	-	-	-	-	-	-	-
3.208	-	-	-	-	-	-	-	-	-	-
3.055	-	-	-	-	-	-	-	-	-	-
3.052	-	-	-	-	-	-	-	-	-	-
3.429	-	-	-	-	-	-	-	-	-	-
3.279	-	-	-	-	-	-	-	-	-	-
3.224	-	-	-	-	-	-	-	-	-	-
3.311	-	-	-	-	-	-	-	-	-	-
2.908	-	-	-	-	-	-	-	-	-	-
2.839	-	-	-	-	-	-	-	-	-	-

Continues on next page

Table S11 – *Continued from previous page*

C-H	H-O	C-O	O-O	C-C	H-N	C-N	N-O	H-Z	C-Zn	O-Zn
3.485	-	-	-	-	-	-	-	-	-	-
3.127	-	-	-	-	-	-	-	-	-	-
Frame number : 40000										
2.643	3.377	-	-	3.276	3.086	-	-	-	-	-
3.12	3.427	-	-	3.318	-	-	-	-	-	-
3.169	3.378	-	-	3.431	-	-	-	-	-	-
3.288	3.207	-	-	3.402	-	-	-	-	-	-
2.542	2.763	-	-	3.26	-	-	-	-	-	-
2.786	3.273	-	-	3.468	-	-	-	-	-	-
3.107	3.033	-	-	3.412	-	-	-	-	-	-
2.867	3.04	-	-	3.431	-	-	-	-	-	-
2.746	-	-	-	3.221	-	-	-	-	-	-
3.22	-	-	-	3.118	-	-	-	-	-	-
2.434	-	-	-	3.152	-	-	-	-	-	-
3.361	-	-	-	3.453	-	-	-	-	-	-
3.384	-	-	-	3.32	-	-	-	-	-	-
2.758	-	-	-	3.389	-	-	-	-	-	-
3.115	-	-	-	3.181	-	-	-	-	-	-
3.408	-	-	-	3.383	-	-	-	-	-	-
3.499	-	-	-	2.94	-	-	-	-	-	-
3.357	-	-	-	3.0	-	-	-	-	-	-
2.911	-	-	-	3.451	-	-	-	-	-	-
2.597	-	-	-	3.418	-	-	-	-	-	-
3.264	-	-	-	-	-	-	-	-	-	-

Continues on next page

Table S11 – *Continued from previous page*

C-H	H-O	C-O	O-O	C-C	H-N	C-N	N-O	H-Z	C-Zn	O-Zn
3.132	-	-	-	-	-	-	-	-	-	-
3.053	-	-	-	-	-	-	-	-	-	-
3.332	-	-	-	-	-	-	-	-	-	-
3.31	-	-	-	-	-	-	-	-	-	-
3.1	-	-	-	-	-	-	-	-	-	-
3.189	-	-	-	-	-	-	-	-	-	-
3.018	-	-	-	-	-	-	-	-	-	-
2.971	-	-	-	-	-	-	-	-	-	-
2.551	-	-	-	-	-	-	-	-	-	-
2.357	-	-	-	-	-	-	-	-	-	-
2.675	-	-	-	-	-	-	-	-	-	-
3.314	-	-	-	-	-	-	-	-	-	-
3.407	-	-	-	-	-	-	-	-	-	-
3.441	-	-	-	-	-	-	-	-	-	-
2.853	-	-	-	-	-	-	-	-	-	-
3.277	-	-	-	-	-	-	-	-	-	-
3.417	-	-	-	-	-	-	-	-	-	-
3.222	-	-	-	-	-	-	-	-	-	-
3.037	-	-	-	-	-	-	-	-	-	-
2.951	-	-	-	-	-	-	-	-	-	-
2.977	-	-	-	-	-	-	-	-	-	-
2.605	-	-	-	-	-	-	-	-	-	-
3.335	-	-	-	-	-	-	-	-	-	-
2.805	-	-	-	-	-	-	-	-	-	-

Continues on next page

Table S11 – *Continued from previous page*

C-H	H-O	C-O	O-O	C-C	H-N	C-N	N-O	H-Z	C-Zn	O-Zn
3.415	-	-	-	-	-	-	-	-	-	-
2.897	-	-	-	-	-	-	-	-	-	-
3.367	-	-	-	-	-	-	-	-	-	-
3.371	-	-	-	-	-	-	-	-	-	-
3.37	-	-	-	-	-	-	-	-	-	-
3.439	-	-	-	-	-	-	-	-	-	-
3.006	-	-	-	-	-	-	-	-	-	-
2.68	-	-	-	-	-	-	-	-	-	-
2.741	-	-	-	-	-	-	-	-	-	-
3.35	-	-	-	-	-	-	-	-	-	-
3.292	-	-	-	-	-	-	-	-	-	-
3.128	-	-	-	-	-	-	-	-	-	-
2.452	-	-	-	-	-	-	-	-	-	-
3.252	-	-	-	-	-	-	-	-	-	-
3.463	-	-	-	-	-	-	-	-	-	-
3.309	-	-	-	-	-	-	-	-	-	-
3.326	-	-	-	-	-	-	-	-	-	-
3.42	-	-	-	-	-	-	-	-	-	-
2.703	-	-	-	-	-	-	-	-	-	-
2.67	-	-	-	-	-	-	-	-	-	-
3.121	-	-	-	-	-	-	-	-	-	-
2.637	-	-	-	-	-	-	-	-	-	-
3.212	-	-	-	-	-	-	-	-	-	-
2.399	-	-	-	-	-	-	-	-	-	-

Continues on next page

Table S11 – *Continued from previous page*

C-H	H-O	C-O	O-O	C-C	H-N	C-N	N-O	H-Z	C-Zn	O-Zn
2.508	-	-	-	-	-	-	-	-	-	-
3.477	-	-	-	-	-	-	-	-	-	-
3.233	-	-	-	-	-	-	-	-	-	-
3.243	-	-	-	-	-	-	-	-	-	-
3.028	-	-	-	-	-	-	-	-	-	-
3.49	-	-	-	-	-	-	-	-	-	-
3.244	-	-	-	-	-	-	-	-	-	-
3.379	-	-	-	-	-	-	-	-	-	-
2.634	-	-	-	-	-	-	-	-	-	-
2.719	-	-	-	-	-	-	-	-	-	-
3.418	-	-	-	-	-	-	-	-	-	-
2.853	-	-	-	-	-	-	-	-	-	-
3.161	-	-	-	-	-	-	-	-	-	-
3.415	-	-	-	-	-	-	-	-	-	-
2.983	-	-	-	-	-	-	-	-	-	-
2.82	-	-	-	-	-	-	-	-	-	-
2.117	-	-	-	-	-	-	-	-	-	-
2.463	-	-	-	-	-	-	-	-	-	-
3.131	-	-	-	-	-	-	-	-	-	-
2.719	-	-	-	-	-	-	-	-	-	-
3.377	-	-	-	-	-	-	-	-	-	-
3.358	-	-	-	-	-	-	-	-	-	-
2.968	-	-	-	-	-	-	-	-	-	-
3.172	-	-	-	-	-	-	-	-	-	-

Continues on next page

Table S11 – *Continued from previous page*

C-H	H-O	C-O	O-O	C-C	H-N	C-N	N-O	H-Z	C-Zn	O-Zn
3.034	-	-	-	-	-	-	-	-	-	-
2.509	-	-	-	-	-	-	-	-	-	-
3.085	-	-	-	-	-	-	-	-	-	-
3.267	-	-	-	-	-	-	-	-	-	-
3.163	-	-	-	-	-	-	-	-	-	-
3.012	-	-	-	-	-	-	-	-	-	-
3.5	-	-	-	-	-	-	-	-	-	-
Frame number : 50000										
3.243	3.443	-	-	3.359	-	-	-	-	-	-
3.481	3.362	-	-	2.896	-	-	-	-	-	-
3.484	3.458	-	-	2.958	-	-	-	-	-	-
3.353	2.934	-	-	3.442	-	-	-	-	-	-
3.43	3.198	-	-	3.384	-	-	-	-	-	-
2.73	3.121	-	-	3.184	-	-	-	-	-	-
3.236	3.442	-	-	3.215	-	-	-	-	-	-
2.844	3.203	-	-	3.18	-	-	-	-	-	-
3.376	3.301	-	-	3.106	-	-	-	-	-	-
3.377	3.135	-	-	3.282	-	-	-	-	-	-
3.137	-	-	-	3.22	-	-	-	-	-	-
3.223	-	-	-	3.36	-	-	-	-	-	-
3.077	-	-	-	2.773	-	-	-	-	-	-
3.122	-	-	-	3.315	-	-	-	-	-	-
3.306	-	-	-	3.486	-	-	-	-	-	-
2.82	-	-	-	3.33	-	-	-	-	-	-

Continues on next page

C-H	H-O	C-O	O-O	C-C	H-N	C-N	N-O	H-Z	C-Zn	O-Zn
3.243	-	-	-	3.318	-	-	-	-	-	-
2.778	-	-	-	2.858	-	-	-	-	-	-
3.308	-	-	-	3.092	-	-	-	-	-	-
3.099	-	-	-	3.155	-	-	-	-	-	-
3.023	-	-	-	3.487	-	-	-	-	-	-
2.645	-	-	-	3.344	-	-	-	-	-	-
3.028	-	-	-	-	-	-	-	-	-	-
3.084	-	-	-	-	-	-	-	-	-	-
3.239	-	-	-	-	-	-	-	-	-	-
2.812	-	-	-	-	-	-	-	-	-	-
3.489	-	-	-	-	-	-	-	-	-	-
3.12	-	-	-	-	-	-	-	-	-	-
3.5	-	-	-	-	-	-	-	-	-	-
3.241	-	-	-	-	-	-	-	-	-	-
2.798	-	-	-	-	-	-	-	-	-	-
3.296	-	-	-	-	-	-	-	-	-	-
2.944	-	-	-	-	-	-	-	-	-	-
2.425	-	-	-	-	-	-	-	-	-	-
2.419	-	-	-	-	-	-	-	-	-	-
3.062	-	-	-	-	-	-	-	-	-	-
3.397	-	-	-	-	-	-	-	-	-	-
2.902	-	-	-	-	-	-	-	-	-	-
2.533	-	-	-	-	-	-	-	-	-	-
3.093	-	-	-	-	-	-	-	-	-	-

Continues on next page

Table S11 – *Continued from previous page*

C-H	H-O	C-O	O-O	C-C	H-N	C-N	N-O	H-Z	C-Zn	O-Zn
3.126	-	-	-	-	-	-	-	-	-	-
3.24	-	-	-	-	-	-	-	-	-	-
3.386	-	-	-	-	-	-	-	-	-	-
3.306	-	-	-	-	-	-	-	-	-	-
3.318	-	-	-	-	-	-	-	-	-	-
3.34	-	-	-	-	-	-	-	-	-	-
3.283	-	-	-	-	-	-	-	-	-	-
2.891	-	-	-	-	-	-	-	-	-	-
3.351	-	-	-	-	-	-	-	-	-	-
3.289	-	-	-	-	-	-	-	-	-	-
3.318	-	-	-	-	-	-	-	-	-	-
2.705	-	-	-	-	-	-	-	-	-	-
3.226	-	-	-	-	-	-	-	-	-	-
3.332	-	-	-	-	-	-	-	-	-	-
3.426	-	-	-	-	-	-	-	-	-	-
3.389	-	-	-	-	-	-	-	-	-	-
2.961	-	-	-	-	-	-	-	-	-	-
2.982	-	-	-	-	-	-	-	-	-	-
3.39	-	-	-	-	-	-	-	-	-	-
3.104	-	-	-	-	-	-	-	-	-	-
3.02	-	-	-	-	-	-	-	-	-	-
3.464	-	-	-	-	-	-	-	-	-	-
3.436	-	-	-	-	-	-	-	-	-	-
2.889	-	-	-	-	-	-	-	-	-	-

Continues on next page

Table S11 – *Continued from previous page*

C-H	H-O	C-O	O-O	C-C	H-N	C-N	N-O	H-Z	C-Zn	O-Zn
2.849	-	-	-	-	-	-	-	-	-	-
2.888	-	-	-	-	-	-	-	-	-	-
3.466	-	-	-	-	-	-	-	-	-	-
3.488	-	-	-	-	-	-	-	-	-	-
2.806	-	-	-	-	-	-	-	-	-	-
1.817	-	-	-	-	-	-	-	-	-	-
2.632	-	-	-	-	-	-	-	-	-	-
3.477	-	-	-	-	-	-	-	-	-	-
3.212	-	-	-	-	-	-	-	-	-	-
3.162	-	-	-	-	-	-	-	-	-	-
3.271	-	-	-	-	-	-	-	-	-	-
3.009	-	-	-	-	-	-	-	-	-	-
3.333	-	-	-	-	-	-	-	-	-	-
3.468	-	-	-	-	-	-	-	-	-	-
3.394	-	-	-	-	-	-	-	-	-	-
3.04	-	-	-	-	-	-	-	-	-	-
3.398	-	-	-	-	-	-	-	-	-	-
3.405	-	-	-	-	-	-	-	-	-	-
3.136	-	-	-	-	-	-	-	-	-	-
3.462	-	-	-	-	-	-	-	-	-	-
3.393	-	-	-	-	-	-	-	-	-	-
3.37	-	-	-	-	-	-	-	-	-	-
3.227	-	-	-	-	-	-	-	-	-	-
3.075	-	-	-	-	-	-	-	-	-	-

Continues on next page

Table S11 – *Continued from previous page*

C-H	H-O	C-O	O-O	C-C	H-N	C-N	N-O	H-Z	C-Zn	O-Zn
3.403	-	-	-	-	-	-	-	-	-	-
3.207	-	-	-	-	-	-	-	-	-	-
3.15	-	-	-	-	-	-	-	-	-	-
2.96	-	-	-	-	-	-	-	-	-	-
2.834	-	-	-	-	-	-	-	-	-	-
2.046	-	-	-	-	-	-	-	-	-	-
2.408	-	-	-	-	-	-	-	-	-	-
3.426	-	-	-	-	-	-	-	-	-	-
2.856	-	-	-	-	-	-	-	-	-	-
2.635	-	-	-	-	-	-	-	-	-	-
3.122	-	-	-	-	-	-	-	-	-	-
3.287	-	-	-	-	-	-	-	-	-	-
2.964	-	-	-	-	-	-	-	-	-	-
3.345	-	-	-	-	-	-	-	-	-	-
2.567	-	-	-	-	-	-	-	-	-	-
2.847	-	-	-	-	-	-	-	-	-	-
3.397	-	-	-	-	-	-	-	-	-	-
3.045	-	-	-	-	-	-	-	-	-	-
2.89	-	-	-	-	-	-	-	-	-	-
3.063	-	-	-	-	-	-	-	-	-	-
2.748	-	-	-	-	-	-	-	-	-	-
3.436	-	-	-	-	-	-	-	-	-	-
3.358	-	-	-	-	-	-	-	-	-	-
Frame number : 60000										

Continues on next page

Table S11 – *Continued from previous page*

C-H	H-O	C-O	O-O	C-C	H-N	C-N	N-O	H-Z	C-Zn	O-Zn
2.938	2.793	3.273	-	3.35	-	-	-	-	-	-
2.926	2.897	-	-	3.364	-	-	-	-	-	-
3.29	2.856	-	-	3.313	-	-	-	-	-	-
2.962	2.827	-	-	3.387	-	-	-	-	-	-
3.251	3.001	-	-	3.354	-	-	-	-	-	-
3.334	2.491	-	-	3.463	-	-	-	-	-	-
3.299	3.293	-	-	3.441	-	-	-	-	-	-
3.193	3.079	-	-	3.282	-	-	-	-	-	-
3.223	3.016	-	-	2.965	-	-	-	-	-	-
3.306	-	-	-	3.01	-	-	-	-	-	-
2.771	-	-	-	3.35	-	-	-	-	-	-
2.857	-	-	-	3.456	-	-	-	-	-	-
3.046	-	-	-	3.408	-	-	-	-	-	-
2.571	-	-	-	3.097	-	-	-	-	-	-
3.191	-	-	-	3.456	-	-	-	-	-	-
2.854	-	-	-	3.057	-	-	-	-	-	-
3.465	-	-	-	3.119	-	-	-	-	-	-
2.969	-	-	-	3.186	-	-	-	-	-	-
3.383	-	-	-	3.021	-	-	-	-	-	-
3.297	-	-	-	3.186	-	-	-	-	-	-
2.449	-	-	-	3.468	-	-	-	-	-	-
3.199	-	-	-	3.299	-	-	-	-	-	-
3.413	-	-	-	3.445	-	-	-	-	-	-
3.379	-	-	-	3.312	-	-	-	-	-	-

Continues on next page

Table S11 – *Continued from previous page*

C-H	H-O	C-O	O-O	C-C	H-N	C-N	N-O	H-Z	C-Zn	O-Zn
3.497	-	-	-	3.242	-	-	-	-	-	-
3.45	-	-	-	3.47	-	-	-	-	-	-
3.359	-	-	-	3.077	-	-	-	-	-	-
3.457	-	-	-	3.252	-	-	-	-	-	-
3.129	-	-	-	3.413	-	-	-	-	-	-
2.669	-	-	-	-	-	-	-	-	-	-
2.331	-	-	-	-	-	-	-	-	-	-
2.376	-	-	-	-	-	-	-	-	-	-
2.652	-	-	-	-	-	-	-	-	-	-
2.972	-	-	-	-	-	-	-	-	-	-
3.327	-	-	-	-	-	-	-	-	-	-
2.697	-	-	-	-	-	-	-	-	-	-
2.802	-	-	-	-	-	-	-	-	-	-
3.336	-	-	-	-	-	-	-	-	-	-
3.034	-	-	-	-	-	-	-	-	-	-
3.389	-	-	-	-	-	-	-	-	-	-
3.3	-	-	-	-	-	-	-	-	-	-
3.281	-	-	-	-	-	-	-	-	-	-
3.011	-	-	-	-	-	-	-	-	-	-
2.759	-	-	-	-	-	-	-	-	-	-
3.186	-	-	-	-	-	-	-	-	-	-
3.355	-	-	-	-	-	-	-	-	-	-
2.27	-	-	-	-	-	-	-	-	-	-
3.493	-	-	-	-	-	-	-	-	-	-

Continues on next page

Table S11 – *Continued from previous page*

C-H	H-O	C-O	O-O	C-C	H-N	C-N	N-O	H-Z	C-Zn	O-Zn
3.145	-	-	-	-	-	-	-	-	-	-
3.352	-	-	-	-	-	-	-	-	-	-
3.167	-	-	-	-	-	-	-	-	-	-
2.872	-	-	-	-	-	-	-	-	-	-
3.257	-	-	-	-	-	-	-	-	-	-
3.177	-	-	-	-	-	-	-	-	-	-
3.38	-	-	-	-	-	-	-	-	-	-
3.296	-	-	-	-	-	-	-	-	-	-
3.263	-	-	-	-	-	-	-	-	-	-
2.415	-	-	-	-	-	-	-	-	-	-
2.747	-	-	-	-	-	-	-	-	-	-
3.291	-	-	-	-	-	-	-	-	-	-
2.477	-	-	-	-	-	-	-	-	-	-
3.009	-	-	-	-	-	-	-	-	-	-
2.755	-	-	-	-	-	-	-	-	-	-
3.043	-	-	-	-	-	-	-	-	-	-
3.056	-	-	-	-	-	-	-	-	-	-
2.998	-	-	-	-	-	-	-	-	-	-
3.377	-	-	-	-	-	-	-	-	-	-
3.268	-	-	-	-	-	-	-	-	-	-
3.459	-	-	-	-	-	-	-	-	-	-
3.423	-	-	-	-	-	-	-	-	-	-
3.491	-	-	-	-	-	-	-	-	-	-
2.849	-	-	-	-	-	-	-	-	-	-

Continues on next page

Table S11 – *Continued from previous page*

C-H	H-O	C-O	O-O	C-C	H-N	C-N	N-O	H-Z	C-Zn	O-Zn
3.323	-	-	-	-	-	-	-	-	-	-
3.161	-	-	-	-	-	-	-	-	-	-
2.957	-	-	-	-	-	-	-	-	-	-
3.346	-	-	-	-	-	-	-	-	-	-
3.365	-	-	-	-	-	-	-	-	-	-
2.646	-	-	-	-	-	-	-	-	-	-
3.453	-	-	-	-	-	-	-	-	-	-
2.201	-	-	-	-	-	-	-	-	-	-
2.581	-	-	-	-	-	-	-	-	-	-
3.464	-	-	-	-	-	-	-	-	-	-
2.729	-	-	-	-	-	-	-	-	-	-
2.955	-	-	-	-	-	-	-	-	-	-
2.912	-	-	-	-	-	-	-	-	-	-
3.122	-	-	-	-	-	-	-	-	-	-
3.267	-	-	-	-	-	-	-	-	-	-
2.481	-	-	-	-	-	-	-	-	-	-
2.893	-	-	-	-	-	-	-	-	-	-
3.07	-	-	-	-	-	-	-	-	-	-
3.418	-	-	-	-	-	-	-	-	-	-
3.014	-	-	-	-	-	-	-	-	-	-
3.47	-	-	-	-	-	-	-	-	-	-
2.712	-	-	-	-	-	-	-	-	-	-
2.633	-	-	-	-	-	-	-	-	-	-
3.218	-	-	-	-	-	-	-	-	-	-

Continues on next page

Table S11 – *Continued from previous page*

C-H	H-O	C-O	O-O	C-C	H-N	C-N	N-O	H-Z	C-Zn	O-Zn
3.365	-	-	-	-	-	-	-	-	-	-
3.031	-	-	-	-	-	-	-	-	-	-
3.117	-	-	-	-	-	-	-	-	-	-
3.436	-	-	-	-	-	-	-	-	-	-
3.484	-	-	-	-	-	-	-	-	-	-
3.191	-	-	-	-	-	-	-	-	-	-
3.498	-	-	-	-	-	-	-	-	-	-
3.418	-	-	-	-	-	-	-	-	-	-
3.206	-	-	-	-	-	-	-	-	-	-
2.932	-	-	-	-	-	-	-	-	-	-
2.595	-	-	-	-	-	-	-	-	-	-
3.059	-	-	-	-	-	-	-	-	-	-
Frame number : 70000										
3.086	3.108	3.368	-	3.259	-	-	-	-	-	-
2.994	3.152	3.304	-	3.431	-	-	-	-	-	-
3.482	2.858	-	-	3.259	-	-	-	-	-	-
3.006	3.459	-	-	3.413	-	-	-	-	-	-
3.353	3.303	-	-	3.319	-	-	-	-	-	-
3.351	2.668	-	-	2.611	-	-	-	-	-	-
3.487	3.375	-	-	2.59	-	-	-	-	-	-
3.025	3.354	-	-	3.218	-	-	-	-	-	-
2.742	2.345	-	-	3.482	-	-	-	-	-	-
3.116	3.468	-	-	3.183	-	-	-	-	-	-
2.792	3.154	-	-	3.288	-	-	-	-	-	-

Continues on next page

Table S11 – *Continued from previous page*

C-H	H-O	C-O	O-O	C-C	H-N	C-N	N-O	H-Z	C-Zn	O-Zn
2.877	2.702	-	-	2.926	-	-	-	-	-	-
3.285	3.432	-	-	3.292	-	-	-	-	-	-
3.424	3.493	-	-	3.392	-	-	-	-	-	-
3.414	3.222	-	-	3.084	-	-	-	-	-	-
3.447	-	-	-	3.331	-	-	-	-	-	-
2.536	-	-	-	3.078	-	-	-	-	-	-
2.89	-	-	-	3.328	-	-	-	-	-	-
3.323	-	-	-	2.808	-	-	-	-	-	-
3.137	-	-	-	3.172	-	-	-	-	-	-
2.787	-	-	-	-	-	-	-	-	-	-
2.214	-	-	-	-	-	-	-	-	-	-
2.698	-	-	-	-	-	-	-	-	-	-
3.066	-	-	-	-	-	-	-	-	-	-
3.228	-	-	-	-	-	-	-	-	-	-
3.283	-	-	-	-	-	-	-	-	-	-
2.98	-	-	-	-	-	-	-	-	-	-
3.102	-	-	-	-	-	-	-	-	-	-
3.489	-	-	-	-	-	-	-	-	-	-
3.361	-	-	-	-	-	-	-	-	-	-
2.869	-	-	-	-	-	-	-	-	-	-
2.839	-	-	-	-	-	-	-	-	-	-
2.98	-	-	-	-	-	-	-	-	-	-
3.369	-	-	-	-	-	-	-	-	-	-
2.634	-	-	-	-	-	-	-	-	-	-

Continues on next page

Table S11 – *Continued from previous page*

C-H	H-O	C-O	O-O	C-C	H-N	C-N	N-O	H-Z	C-Zn	O-Zn
2.086	-	-	-	-	-	-	-	-	-	-
2.711	-	-	-	-	-	-	-	-	-	-
3.086	-	-	-	-	-	-	-	-	-	-
2.829	-	-	-	-	-	-	-	-	-	-
3.234	-	-	-	-	-	-	-	-	-	-
2.687	-	-	-	-	-	-	-	-	-	-
2.799	-	-	-	-	-	-	-	-	-	-
3.08	-	-	-	-	-	-	-	-	-	-
3.235	-	-	-	-	-	-	-	-	-	-
3.284	-	-	-	-	-	-	-	-	-	-
3.372	-	-	-	-	-	-	-	-	-	-
2.963	-	-	-	-	-	-	-	-	-	-
3.238	-	-	-	-	-	-	-	-	-	-
3.147	-	-	-	-	-	-	-	-	-	-
3.336	-	-	-	-	-	-	-	-	-	-
3.041	-	-	-	-	-	-	-	-	-	-
3.296	-	-	-	-	-	-	-	-	-	-
3.413	-	-	-	-	-	-	-	-	-	-
2.381	-	-	-	-	-	-	-	-	-	-
2.957	-	-	-	-	-	-	-	-	-	-
3.138	-	-	-	-	-	-	-	-	-	-
2.91	-	-	-	-	-	-	-	-	-	-
2.654	-	-	-	-	-	-	-	-	-	-
3.105	-	-	-	-	-	-	-	-	-	-

Continues on next page

Table S11 – *Continued from previous page*

C-H	H-O	C-O	O-O	C-C	H-N	C-N	N-O	H-Z	C-Zn	O-Zn
2.804	-	-	-	-	-	-	-	-	-	-
2.879	-	-	-	-	-	-	-	-	-	-
3.118	-	-	-	-	-	-	-	-	-	-
3.223	-	-	-	-	-	-	-	-	-	-
3.466	-	-	-	-	-	-	-	-	-	-
2.685	-	-	-	-	-	-	-	-	-	-
2.558	-	-	-	-	-	-	-	-	-	-
3.105	-	-	-	-	-	-	-	-	-	-
3.177	-	-	-	-	-	-	-	-	-	-
2.857	-	-	-	-	-	-	-	-	-	-
2.093	-	-	-	-	-	-	-	-	-	-
3.099	-	-	-	-	-	-	-	-	-	-
2.798	-	-	-	-	-	-	-	-	-	-
2.87	-	-	-	-	-	-	-	-	-	-
3.414	-	-	-	-	-	-	-	-	-	-
3.46	-	-	-	-	-	-	-	-	-	-
3.351	-	-	-	-	-	-	-	-	-	-
3.413	-	-	-	-	-	-	-	-	-	-
2.885	-	-	-	-	-	-	-	-	-	-
3.045	-	-	-	-	-	-	-	-	-	-
3.338	-	-	-	-	-	-	-	-	-	-
3.404	-	-	-	-	-	-	-	-	-	-
2.661	-	-	-	-	-	-	-	-	-	-
2.677	-	-	-	-	-	-	-	-	-	-

Continues on next page

Table S11 – *Continued from previous page*

C-H	H-O	C-O	O-O	C-C	H-N	C-N	N-O	H-Z	C-Zn	O-Zn
2.923	-	-	-	-	-	-	-	-	-	-
2.364	-	-	-	-	-	-	-	-	-	-
3.114	-	-	-	-	-	-	-	-	-	-
2.252	-	-	-	-	-	-	-	-	-	-
2.736	-	-	-	-	-	-	-	-	-	-
3.271	-	-	-	-	-	-	-	-	-	-
2.72	-	-	-	-	-	-	-	-	-	-
3.118	-	-	-	-	-	-	-	-	-	-
2.959	-	-	-	-	-	-	-	-	-	-
3.344	-	-	-	-	-	-	-	-	-	-
2.985	-	-	-	-	-	-	-	-	-	-
2.288	-	-	-	-	-	-	-	-	-	-
2.951	-	-	-	-	-	-	-	-	-	-
3.113	-	-	-	-	-	-	-	-	-	-
3.274	-	-	-	-	-	-	-	-	-	-
2.89	-	-	-	-	-	-	-	-	-	-
3.012	-	-	-	-	-	-	-	-	-	-
3.346	-	-	-	-	-	-	-	-	-	-
3.019	-	-	-	-	-	-	-	-	-	-
2.937	-	-	-	-	-	-	-	-	-	-
3.271	-	-	-	-	-	-	-	-	-	-
3.249	-	-	-	-	-	-	-	-	-	-
3.347	-	-	-	-	-	-	-	-	-	-
3.095	-	-	-	-	-	-	-	-	-	-

Continues on next page

Table S11 – *Continued from previous page*

C-H	H-O	C-O	O-O	C-C	H-N	C-N	N-O	H-Z	C-Zn	O-Zn
Frame number : 80000										
2.759	2.977	3.49	-	3.283	3.47	-	-	-	-	-
2.889	2.981	-	-	3.031	3.493	-	-	-	-	-
2.725	3.006	-	-	3.392	-	-	-	-	-	-
3.219	3.368	-	-	3.46	-	-	-	-	-	-
3.22	2.778	-	-	3.491	-	-	-	-	-	-
3.342	2.634	-	-	3.274	-	-	-	-	-	-
3.386	3.379	-	-	3.379	-	-	-	-	-	-
3.023	2.867	-	-	3.143	-	-	-	-	-	-
2.238	3.347	-	-	3.03	-	-	-	-	-	-
2.724	-	-	-	3.352	-	-	-	-	-	-
2.7	-	-	-	3.395	-	-	-	-	-	-
3.429	-	-	-	3.176	-	-	-	-	-	-
3.473	-	-	-	3.25	-	-	-	-	-	-
3.455	-	-	-	2.707	-	-	-	-	-	-
2.687	-	-	-	3.296	-	-	-	-	-	-
3.172	-	-	-	3.201	-	-	-	-	-	-
3.402	-	-	-	3.242	-	-	-	-	-	-
2.887	-	-	-	3.066	-	-	-	-	-	-
3.229	-	-	-	3.301	-	-	-	-	-	-
3.386	-	-	-	2.8	-	-	-	-	-	-
2.89	-	-	-	3.266	-	-	-	-	-	-
2.409	-	-	-	3.398	-	-	-	-	-	-
2.708	-	-	-	3.432	-	-	-	-	-	-

Continues on next page

Table S11 – *Continued from previous page*

C-H	H-O	C-O	O-O	C-C	H-N	C-N	N-O	H-Z	C-Zn	O-Zn
3.259	-	-	-	-	-	-	-	-	-	-
2.73	-	-	-	-	-	-	-	-	-	-
2.756	-	-	-	-	-	-	-	-	-	-
3.453	-	-	-	-	-	-	-	-	-	-
3.251	-	-	-	-	-	-	-	-	-	-
3.229	-	-	-	-	-	-	-	-	-	-
2.764	-	-	-	-	-	-	-	-	-	-
3.437	-	-	-	-	-	-	-	-	-	-
3.011	-	-	-	-	-	-	-	-	-	-
3.482	-	-	-	-	-	-	-	-	-	-
3.458	-	-	-	-	-	-	-	-	-	-
2.927	-	-	-	-	-	-	-	-	-	-
3.375	-	-	-	-	-	-	-	-	-	-
3.012	-	-	-	-	-	-	-	-	-	-
2.5	-	-	-	-	-	-	-	-	-	-
2.883	-	-	-	-	-	-	-	-	-	-
2.935	-	-	-	-	-	-	-	-	-	-
2.836	-	-	-	-	-	-	-	-	-	-
3.398	-	-	-	-	-	-	-	-	-	-
3.366	-	-	-	-	-	-	-	-	-	-
3.273	-	-	-	-	-	-	-	-	-	-
3.193	-	-	-	-	-	-	-	-	-	-
3.37	-	-	-	-	-	-	-	-	-	-
2.876	-	-	-	-	-	-	-	-	-	-

Continues on next page

Table S11 – *Continued from previous page*

C-H	H-O	C-O	O-O	C-C	H-N	C-N	N-O	H-Z	C-Zn	O-Zn
3.459	-	-	-	-	-	-	-	-	-	-
2.846	-	-	-	-	-	-	-	-	-	-
2.748	-	-	-	-	-	-	-	-	-	-
2.522	-	-	-	-	-	-	-	-	-	-
2.953	-	-	-	-	-	-	-	-	-	-
3.412	-	-	-	-	-	-	-	-	-	-
3.072	-	-	-	-	-	-	-	-	-	-
3.45	-	-	-	-	-	-	-	-	-	-
2.559	-	-	-	-	-	-	-	-	-	-
3.159	-	-	-	-	-	-	-	-	-	-
3.304	-	-	-	-	-	-	-	-	-	-
3.494	-	-	-	-	-	-	-	-	-	-
3.438	-	-	-	-	-	-	-	-	-	-
3.341	-	-	-	-	-	-	-	-	-	-
3.198	-	-	-	-	-	-	-	-	-	-
2.87	-	-	-	-	-	-	-	-	-	-
3.014	-	-	-	-	-	-	-	-	-	-
3.02	-	-	-	-	-	-	-	-	-	-
3.424	-	-	-	-	-	-	-	-	-	-
2.949	-	-	-	-	-	-	-	-	-	-
2.892	-	-	-	-	-	-	-	-	-	-
3.141	-	-	-	-	-	-	-	-	-	-
3.233	-	-	-	-	-	-	-	-	-	-
3.496	-	-	-	-	-	-	-	-	-	-

Continues on next page

Table S11 – *Continued from previous page*

C-H	H-O	C-O	O-O	C-C	H-N	C-N	N-O	H-Z	C-Zn	O-Zn
2.644	-	-	-	-	-	-	-	-	-	-
1.895	-	-	-	-	-	-	-	-	-	-
2.914	-	-	-	-	-	-	-	-	-	-
3.331	-	-	-	-	-	-	-	-	-	-
3.064	-	-	-	-	-	-	-	-	-	-
3.318	-	-	-	-	-	-	-	-	-	-
2.825	-	-	-	-	-	-	-	-	-	-
2.99	-	-	-	-	-	-	-	-	-	-
3.213	-	-	-	-	-	-	-	-	-	-
3.237	-	-	-	-	-	-	-	-	-	-
3.479	-	-	-	-	-	-	-	-	-	-
2.919	-	-	-	-	-	-	-	-	-	-
3.457	-	-	-	-	-	-	-	-	-	-
2.893	-	-	-	-	-	-	-	-	-	-
2.629	-	-	-	-	-	-	-	-	-	-
3.25	-	-	-	-	-	-	-	-	-	-
2.672	-	-	-	-	-	-	-	-	-	-
3.325	-	-	-	-	-	-	-	-	-	-
2.407	-	-	-	-	-	-	-	-	-	-
2.925	-	-	-	-	-	-	-	-	-	-
3.13	-	-	-	-	-	-	-	-	-	-
2.722	-	-	-	-	-	-	-	-	-	-
3.495	-	-	-	-	-	-	-	-	-	-
3.134	-	-	-	-	-	-	-	-	-	-

Continues on next page

Table S11 – *Continued from previous page*

C-H	H-O	C-O	O-O	C-C	H-N	C-N	N-O	H-Z	C-Zn	O-Zn
2.854	-	-	-	-	-	-	-	-	-	-
2.256	-	-	-	-	-	-	-	-	-	-
3.089	-	-	-	-	-	-	-	-	-	-
3.044	-	-	-	-	-	-	-	-	-	-
3.284	-	-	-	-	-	-	-	-	-	-
3.093	-	-	-	-	-	-	-	-	-	-
2.32	-	-	-	-	-	-	-	-	-	-
2.863	-	-	-	-	-	-	-	-	-	-
2.777	-	-	-	-	-	-	-	-	-	-
3.256	-	-	-	-	-	-	-	-	-	-
3.287	-	-	-	-	-	-	-	-	-	-
3.109	-	-	-	-	-	-	-	-	-	-
3.141	-	-	-	-	-	-	-	-	-	-
Frame number : 90000										
3.013	2.928	-	-	3.498	3.399	-	-	-	-	-
3.332	3.037	-	-	3.416	-	-	-	-	-	-
3.44	3.421	-	-	3.477	-	-	-	-	-	-
3.399	3.341	-	-	3.426	-	-	-	-	-	-
3.458	3.398	-	-	3.18	-	-	-	-	-	-
2.747	3.373	-	-	3.398	-	-	-	-	-	-
3.309	2.766	-	-	2.997	-	-	-	-	-	-
3.032	3.352	-	-	2.983	-	-	-	-	-	-
3.488	-	-	-	2.831	-	-	-	-	-	-
3.288	-	-	-	2.92	-	-	-	-	-	-

Continues on next page

Table S11 – *Continued from previous page*

C-H	H-O	C-O	O-O	C-C	H-N	C-N	N-O	H-Z	C-Zn	O-Zn
3.381	-	-	-	3.217	-	-	-	-	-	-
3.18	-	-	-	3.106	-	-	-	-	-	-
2.627	-	-	-	3.306	-	-	-	-	-	-
3.477	-	-	-	2.945	-	-	-	-	-	-
2.963	-	-	-	3.278	-	-	-	-	-	-
3.478	-	-	-	3.198	-	-	-	-	-	-
2.723	-	-	-	3.336	-	-	-	-	-	-
3.231	-	-	-	3.074	-	-	-	-	-	-
3.337	-	-	-	3.392	-	-	-	-	-	-
3.119	-	-	-	3.355	-	-	-	-	-	-
2.941	-	-	-	2.956	-	-	-	-	-	-
3.221	-	-	-	2.82	-	-	-	-	-	-
3.343	-	-	-	3.465	-	-	-	-	-	-
3.101	-	-	-	-	-	-	-	-	-	-
3.451	-	-	-	-	-	-	-	-	-	-
2.855	-	-	-	-	-	-	-	-	-	-
3.213	-	-	-	-	-	-	-	-	-	-
3.428	-	-	-	-	-	-	-	-	-	-
3.358	-	-	-	-	-	-	-	-	-	-
3.446	-	-	-	-	-	-	-	-	-	-
2.913	-	-	-	-	-	-	-	-	-	-
3.351	-	-	-	-	-	-	-	-	-	-
2.807	-	-	-	-	-	-	-	-	-	-
2.295	-	-	-	-	-	-	-	-	-	-

Continues on next page

Table S11 – *Continued from previous page*

C-H	H-O	C-O	O-O	C-C	H-N	C-N	N-O	H-Z	C-Zn	O-Zn
2.812	-	-	-	-	-	-	-	-	-	-
3.126	-	-	-	-	-	-	-	-	-	-
3.394	-	-	-	-	-	-	-	-	-	-
2.929	-	-	-	-	-	-	-	-	-	-
3.026	-	-	-	-	-	-	-	-	-	-
3.355	-	-	-	-	-	-	-	-	-	-
3.141	-	-	-	-	-	-	-	-	-	-
2.682	-	-	-	-	-	-	-	-	-	-
2.598	-	-	-	-	-	-	-	-	-	-
3.069	-	-	-	-	-	-	-	-	-	-
3.381	-	-	-	-	-	-	-	-	-	-
3.162	-	-	-	-	-	-	-	-	-	-
2.778	-	-	-	-	-	-	-	-	-	-
2.739	-	-	-	-	-	-	-	-	-	-
3.25	-	-	-	-	-	-	-	-	-	-
2.958	-	-	-	-	-	-	-	-	-	-
3.05	-	-	-	-	-	-	-	-	-	-
3.259	-	-	-	-	-	-	-	-	-	-
2.377	-	-	-	-	-	-	-	-	-	-
3.426	-	-	-	-	-	-	-	-	-	-
3.469	-	-	-	-	-	-	-	-	-	-
3.389	-	-	-	-	-	-	-	-	-	-
3.371	-	-	-	-	-	-	-	-	-	-
3.127	-	-	-	-	-	-	-	-	-	-

Continues on next page

Table S11 – *Continued from previous page*

C-H	H-O	C-O	O-O	C-C	H-N	C-N	N-O	H-Z	C-Zn	O-Zn
3.144	-	-	-	-	-	-	-	-	-	-
3.226	-	-	-	-	-	-	-	-	-	-
2.885	-	-	-	-	-	-	-	-	-	-
2.887	-	-	-	-	-	-	-	-	-	-
2.971	-	-	-	-	-	-	-	-	-	-
2.654	-	-	-	-	-	-	-	-	-	-
2.68	-	-	-	-	-	-	-	-	-	-
3.296	-	-	-	-	-	-	-	-	-	-
3.352	-	-	-	-	-	-	-	-	-	-
3.056	-	-	-	-	-	-	-	-	-	-
2.304	-	-	-	-	-	-	-	-	-	-
3.338	-	-	-	-	-	-	-	-	-	-
3.119	-	-	-	-	-	-	-	-	-	-
3.205	-	-	-	-	-	-	-	-	-	-
3.205	-	-	-	-	-	-	-	-	-	-
3.219	-	-	-	-	-	-	-	-	-	-
3.113	-	-	-	-	-	-	-	-	-	-
2.838	-	-	-	-	-	-	-	-	-	-
3.446	-	-	-	-	-	-	-	-	-	-
3.247	-	-	-	-	-	-	-	-	-	-
2.758	-	-	-	-	-	-	-	-	-	-
2.951	-	-	-	-	-	-	-	-	-	-
3.172	-	-	-	-	-	-	-	-	-	-
2.828	-	-	-	-	-	-	-	-	-	-

Continues on next page

Table S11 – *Continued from previous page*

C-H	H-O	C-O	O-O	C-C	H-N	C-N	N-O	H-Z	C-Zn	O-Zn
3.468	-	-	-	-	-	-	-	-	-	-
3.223	-	-	-	-	-	-	-	-	-	-
2.546	-	-	-	-	-	-	-	-	-	-
3.421	-	-	-	-	-	-	-	-	-	-
3.37	-	-	-	-	-	-	-	-	-	-
3.415	-	-	-	-	-	-	-	-	-	-
3.344	-	-	-	-	-	-	-	-	-	-
2.467	-	-	-	-	-	-	-	-	-	-
2.393	-	-	-	-	-	-	-	-	-	-
3.15	-	-	-	-	-	-	-	-	-	-
3.465	-	-	-	-	-	-	-	-	-	-
2.834	-	-	-	-	-	-	-	-	-	-
3.448	-	-	-	-	-	-	-	-	-	-
3.373	-	-	-	-	-	-	-	-	-	-
2.847	-	-	-	-	-	-	-	-	-	-
3.368	-	-	-	-	-	-	-	-	-	-
2.493	-	-	-	-	-	-	-	-	-	-
1.918	-	-	-	-	-	-	-	-	-	-
2.118	-	-	-	-	-	-	-	-	-	-
3.137	-	-	-	-	-	-	-	-	-	-
3.411	-	-	-	-	-	-	-	-	-	-
2.985	-	-	-	-	-	-	-	-	-	-
3.482	-	-	-	-	-	-	-	-	-	-
3.174	-	-	-	-	-	-	-	-	-	-

Continues on next page

Table S11 – *Continued from previous page*

C-H	H-O	C-O	O-O	C-C	H-N	C-N	N-O	H-Z	C-Zn	O-Zn
3.497	-	-	-	-	-	-	-	-	-	-
2.607	-	-	-	-	-	-	-	-	-	-
3.02	-	-	-	-	-	-	-	-	-	-
3.388	-	-	-	-	-	-	-	-	-	-
3.309	-	-	-	-	-	-	-	-	-	-
3.401	-	-	-	-	-	-	-	-	-	-
Frame number : 100000										
3.313	3.233	-	-	3.235	3.355	-	-	-	-	-
3.406	2.658	-	-	2.924	3.414	-	-	-	-	-
3.492	2.883	-	-	3.085	-	-	-	-	-	-
3.13	2.764	-	-	3.214	-	-	-	-	-	-
2.913	3.297	-	-	3.476	-	-	-	-	-	-
2.886	3.037	-	-	3.289	-	-	-	-	-	-
3.077	3.242	-	-	2.898	-	-	-	-	-	-
2.6	-	-	-	3.342	-	-	-	-	-	-
3.127	-	-	-	3.089	-	-	-	-	-	-
2.825	-	-	-	3.326	-	-	-	-	-	-
3.132	-	-	-	3.216	-	-	-	-	-	-
3.317	-	-	-	2.957	-	-	-	-	-	-
3.223	-	-	-	2.863	-	-	-	-	-	-
2.99	-	-	-	2.987	-	-	-	-	-	-
3.159	-	-	-	3.078	-	-	-	-	-	-
3.405	-	-	-	-	-	-	-	-	-	-
2.785	-	-	-	-	-	-	-	-	-	-

Continues on next page

Table S11 – *Continued from previous page*

C-H	H-O	C-O	O-O	C-C	H-N	C-N	N-O	H-Z	C-Zn	O-Zn
3.04	-	-	-	-	-	-	-	-	-	-
3.292	-	-	-	-	-	-	-	-	-	-
3.3	-	-	-	-	-	-	-	-	-	-
3.257	-	-	-	-	-	-	-	-	-	-
3.354	-	-	-	-	-	-	-	-	-	-
2.835	-	-	-	-	-	-	-	-	-	-
2.638	-	-	-	-	-	-	-	-	-	-
3.353	-	-	-	-	-	-	-	-	-	-
2.937	-	-	-	-	-	-	-	-	-	-
3.408	-	-	-	-	-	-	-	-	-	-
3.416	-	-	-	-	-	-	-	-	-	-
2.907	-	-	-	-	-	-	-	-	-	-
2.901	-	-	-	-	-	-	-	-	-	-
2.213	-	-	-	-	-	-	-	-	-	-
3.169	-	-	-	-	-	-	-	-	-	-
3.335	-	-	-	-	-	-	-	-	-	-
3.378	-	-	-	-	-	-	-	-	-	-
2.775	-	-	-	-	-	-	-	-	-	-
3.424	-	-	-	-	-	-	-	-	-	-
3.313	-	-	-	-	-	-	-	-	-	-
3.068	-	-	-	-	-	-	-	-	-	-
2.865	-	-	-	-	-	-	-	-	-	-
3.165	-	-	-	-	-	-	-	-	-	-
3.051	-	-	-	-	-	-	-	-	-	-

Continues on next page

Table S11 – *Continued from previous page*

C-H	H-O	C-O	O-O	C-C	H-N	C-N	N-O	H-Z	C-Zn	O-Zn
3.128	-	-	-	-	-	-	-	-	-	-
3.377	-	-	-	-	-	-	-	-	-	-
3.469	-	-	-	-	-	-	-	-	-	-
2.566	-	-	-	-	-	-	-	-	-	-
2.41	-	-	-	-	-	-	-	-	-	-
3.075	-	-	-	-	-	-	-	-	-	-
2.93	-	-	-	-	-	-	-	-	-	-
3.024	-	-	-	-	-	-	-	-	-	-
2.918	-	-	-	-	-	-	-	-	-	-
3.26	-	-	-	-	-	-	-	-	-	-
3.287	-	-	-	-	-	-	-	-	-	-
3.281	-	-	-	-	-	-	-	-	-	-
3.156	-	-	-	-	-	-	-	-	-	-
3.431	-	-	-	-	-	-	-	-	-	-
2.951	-	-	-	-	-	-	-	-	-	-
3.214	-	-	-	-	-	-	-	-	-	-
2.567	-	-	-	-	-	-	-	-	-	-
2.565	-	-	-	-	-	-	-	-	-	-
3.067	-	-	-	-	-	-	-	-	-	-
3.257	-	-	-	-	-	-	-	-	-	-
3.266	-	-	-	-	-	-	-	-	-	-
3.229	-	-	-	-	-	-	-	-	-	-
3.039	-	-	-	-	-	-	-	-	-	-
3.413	-	-	-	-	-	-	-	-	-	-

Continues on next page

Table S11 – *Continued from previous page*

C-H	H-O	C-O	O-O	C-C	H-N	C-N	N-O	H-Z	C-Zn	O-Zn
3.142	-	-	-	-	-	-	-	-	-	-
2.721	-	-	-	-	-	-	-	-	-	-
2.181	-	-	-	-	-	-	-	-	-	-
3.011	-	-	-	-	-	-	-	-	-	-
3.351	-	-	-	-	-	-	-	-	-	-
3.195	-	-	-	-	-	-	-	-	-	-
2.978	-	-	-	-	-	-	-	-	-	-
3.255	-	-	-	-	-	-	-	-	-	-
2.872	-	-	-	-	-	-	-	-	-	-
2.768	-	-	-	-	-	-	-	-	-	-
3.066	-	-	-	-	-	-	-	-	-	-
3.132	-	-	-	-	-	-	-	-	-	-
3.259	-	-	-	-	-	-	-	-	-	-
3.224	-	-	-	-	-	-	-	-	-	-
3.166	-	-	-	-	-	-	-	-	-	-
3.461	-	-	-	-	-	-	-	-	-	-
3.019	-	-	-	-	-	-	-	-	-	-
3.468	-	-	-	-	-	-	-	-	-	-
2.841	-	-	-	-	-	-	-	-	-	-
3.301	-	-	-	-	-	-	-	-	-	-
3.169	-	-	-	-	-	-	-	-	-	-
3.458	-	-	-	-	-	-	-	-	-	-
2.466	-	-	-	-	-	-	-	-	-	-
2.026	-	-	-	-	-	-	-	-	-	-

Continues on next page

Table S11 – *Continued from previous page*

C-H	H-O	C-O	O-O	C-C	H-N	C-N	N-O	H-Z	C-Zn	O-Zn
2.4	-	-	-	-	-	-	-	-	-	-
2.902	-	-	-	-	-	-	-	-	-	-
2.57	-	-	-	-	-	-	-	-	-	-
3.032	-	-	-	-	-	-	-	-	-	-
3.274	-	-	-	-	-	-	-	-	-	-
3.015	-	-	-	-	-	-	-	-	-	-
3.299	-	-	-	-	-	-	-	-	-	-
3.375	-	-	-	-	-	-	-	-	-	-
2.277	-	-	-	-	-	-	-	-	-	-
2.953	-	-	-	-	-	-	-	-	-	-
3.467	-	-	-	-	-	-	-	-	-	-
3.395	-	-	-	-	-	-	-	-	-	-
3.255	-	-	-	-	-	-	-	-	-	-
3.207	-	-	-	-	-	-	-	-	-	-

Table S12 Intermolecular distances (r < 3.5 Å) for surface ZnPW⊃-1-Styrene interactions computed for every 1000 molecular dynamic frames.

C-H	H-O	C-O	O-O	C-C	H-N	C-N	N-O	H-Z	C-Zn	O-Zn
Frame number : 0										
3.333	3.253	-	-	3.397	-	-	-	-	-	-
3.262	3.351	-	-	3.37	-	-	-	-	-	-
3.031	2.676	-	-	3.465	-	-	-	-	-	-

Continues on next page

Table S12 – *Continued from previous page*

C-H	H-O	C-O	O-O	C-C	H-N	C-N	N-O	H-Z	C-Zn	O-Zn
2.94	3.338	-	-	3.471	-	-	-	-	-	-
3.421	3.266	-	-	-	-	-	-	-	-	-
3.477	3.298	-	-	-	-	-	-	-	-	-
3.146	2.938	-	-	-	-	-	-	-	-	-
3.465	-	-	-	-	-	-	-	-	-	-
3.486	-	-	-	-	-	-	-	-	-	-
2.984	-	-	-	-	-	-	-	-	-	-
3.309	-	-	-	-	-	-	-	-	-	-
2.841	-	-	-	-	-	-	-	-	-	-
3.197	-	-	-	-	-	-	-	-	-	-
3.186	-	-	-	-	-	-	-	-	-	-
3.231	-	-	-	-	-	-	-	-	-	-
2.983	-	-	-	-	-	-	-	-	-	-
3.066	-	-	-	-	-	-	-	-	-	-
2.964	-	-	-	-	-	-	-	-	-	-
3.058	-	-	-	-	-	-	-	-	-	-
3.101	-	-	-	-	-	-	-	-	-	-
3.427	-	-	-	-	-	-	-	-	-	-
3.467	-	-	-	-	-	-	-	-	-	-
Frame number : 1000										
3.339	3.403	-	-	3.401	-	-	-	3.369	-	-
3.357	3.304	-	-	-	-	-	-	-	-	-
2.826	2.76	-	-	-	-	-	-	-	-	-
2.696	2.586	-	-	-	-	-	-	-	-	-

Continues on next page

Table S12 – *Continued from previous page*

C-H	H-O	C-O	O-O	C-C	H-N	C-N	N-O	H-Z	C-Zn	O-Zn
3.066	3.25	-	-	-	-	-	-	-	-	-
3.473	-	-	-	-	-	-	-	-	-	-
3.367	-	-	-	-	-	-	-	-	-	-
3.463	-	-	-	-	-	-	-	-	-	-
3.328	-	-	-	-	-	-	-	-	-	-
3.267	-	-	-	-	-	-	-	-	-	-
3.365	-	-	-	-	-	-	-	-	-	-
3.262	-	-	-	-	-	-	-	-	-	-
3.124	-	-	-	-	-	-	-	-	-	-
3.006	-	-	-	-	-	-	-	-	-	-
3.364	-	-	-	-	-	-	-	-	-	-
3.465	-	-	-	-	-	-	-	-	-	-
3.017	-	-	-	-	-	-	-	-	-	-
3.411	-	-	-	-	-	-	-	-	-	-
3.448	-	-	-	-	-	-	-	-	-	-
Frame number : 2000										
3.104	-	-	-	-	-	-	-	-	-	-
2.885	-	-	-	-	-	-	-	-	-	-
3.189	-	-	-	-	-	-	-	-	-	-
3.181	-	-	-	-	-	-	-	-	-	-
3.471	-	-	-	-	-	-	-	-	-	-
3.417	-	-	-	-	-	-	-	-	-	-
3.177	-	-	-	-	-	-	-	-	-	-
3.445	-	-	-	-	-	-	-	-	-	-

Continues on next page

Table S12 – *Continued from previous page*

C-H	H-O	C-O	O-O	C-C	H-N	C-N	N-O	H-Z	C-Zn	O-Zn
3.261	-	-	-	-	-	-	-	-	-	-
3.469	-	-	-	-	-	-	-	-	-	-
Frame number : 3000										
3.063	2.236	3.131	-	3.459	-	-	-	-	-	-
2.751	3.265	-	-	3.367	-	-	-	-	-	-
2.648	3.06	-	-	-	-	-	-	-	-	-
3.441	3.429	-	-	-	-	-	-	-	-	-
2.912	3.058	-	-	-	-	-	-	-	-	-
3.097	-	-	-	-	-	-	-	-	-	-
3.209	-	-	-	-	-	-	-	-	-	-
3.421	-	-	-	-	-	-	-	-	-	-
3.249	-	-	-	-	-	-	-	-	-	-
3.272	-	-	-	-	-	-	-	-	-	-
Frame number : 4000										
3.071	2.78	-	-	-	-	-	-	-	-	-
2.984	3.175	-	-	-	-	-	-	-	-	-
2.623	3.121	-	-	-	-	-	-	-	-	-
3.173	-	-	-	-	-	-	-	-	-	-
2.666	-	-	-	-	-	-	-	-	-	-
2.994	-	-	-	-	-	-	-	-	-	-
3.288	-	-	-	-	-	-	-	-	-	-
3.016	-	-	-	-	-	-	-	-	-	-
2.904	-	-	-	-	-	-	-	-	-	-
3.076	-	-	-	-	-	-	-	-	-	-

Continues on next page

Table S12 – *Continued from previous page*

C-H	H-O	C-O	O-O	C-C	H-N	C-N	N-O	H-Z	C-Zn	O-Zn
3.309	-	-	-	-	-	-	-	-	-	-
3.458	-	-	-	-	-	-	-	-	-	-
Frame number : 5000										
3.487	3.0	-	-	3.423	-	-	-	-	-	-
3.397	-	-	-	3.246	-	-	-	-	-	-
3.464	-	-	-	3.149	-	-	-	-	-	-
3.37	-	-	-	3.444	-	-	-	-	-	-
3.108	-	-	-	3.34	-	-	-	-	-	-
3.373	-	-	-	3.482	-	-	-	-	-	-
3.038	-	-	-	-	-	-	-	-	-	-
3.434	-	-	-	-	-	-	-	-	-	-
3.141	-	-	-	-	-	-	-	-	-	-
3.298	-	-	-	-	-	-	-	-	-	-
3.365	-	-	-	-	-	-	-	-	-	-
2.82	-	-	-	-	-	-	-	-	-	-
3.36	-	-	-	-	-	-	-	-	-	-
2.985	-	-	-	-	-	-	-	-	-	-
2.857	-	-	-	-	-	-	-	-	-	-
3.047	-	-	-	-	-	-	-	-	-	-
3.341	-	-	-	-	-	-	-	-	-	-
3.167	-	-	-	-	-	-	-	-	-	-
2.943	-	-	-	-	-	-	-	-	-	-
3.007	-	-	-	-	-	-	-	-	-	-
3.281	-	-	-	-	-	-	-	-	-	-

Continues on next page

Table S12 – *Continued from previous page*

C-H	H-O	C-O	O-O	C-C	H-N	C-N	N-O	H-Z	C-Zn	O-Zn
3.255	-	-	-	-	-	-	-	-	-	-
3.307	-	-	-	-	-	-	-	-	-	-
3.135	-	-	-	-	-	-	-	-	-	-
3.242	-	-	-	-	-	-	-	-	-	-
Frame number : 6000										
2.911	3.437	3.233	-	3.455	-	-	-	3.491	-	-
2.997	2.975	3.14	-	-	-	-	-	-	-	-
3.055	2.395	3.258	-	-	-	-	-	-	-	-
3.371	3.441	-	-	-	-	-	-	-	-	-
3.432	3.191	-	-	-	-	-	-	-	-	-
3.196	3.411	-	-	-	-	-	-	-	-	-
3.094	-	-	-	-	-	-	-	-	-	-
2.674	-	-	-	-	-	-	-	-	-	-
3.126	-	-	-	-	-	-	-	-	-	-
2.906	-	-	-	-	-	-	-	-	-	-
3.411	-	-	-	-	-	-	-	-	-	-
2.965	-	-	-	-	-	-	-	-	-	-
3.021	-	-	-	-	-	-	-	-	-	-
3.463	-	-	-	-	-	-	-	-	-	-
Frame number : 7000										
3.312	3.26	-	-	3.374	-	-	-	-	-	-
3.393	-	-	-	3.153	-	-	-	-	-	-
3.24	-	-	-	3.377	-	-	-	-	-	-
3.39	-	-	-	3.294	-	-	-	-	-	-

Continues on next page

Table S12 – *Continued from previous page*

C-H	H-O	C-O	O-O	C-C	H-N	C-N	N-O	H-Z	C-Zn	O-Zn
3.085	-	-	-	3.333	-	-	-	-	-	-
3.32	-	-	-	-	-	-	-	-	-	-
3.095	-	-	-	-	-	-	-	-	-	-
3.159	-	-	-	-	-	-	-	-	-	-
3.111	-	-	-	-	-	-	-	-	-	-
3.352	-	-	-	-	-	-	-	-	-	-
3.078	-	-	-	-	-	-	-	-	-	-
3.019	-	-	-	-	-	-	-	-	-	-
2.934	-	-	-	-	-	-	-	-	-	-
3.291	-	-	-	-	-	-	-	-	-	-
3.269	-	-	-	-	-	-	-	-	-	-
3.498	-	-	-	-	-	-	-	-	-	-
3.271	-	-	-	-	-	-	-	-	-	-
3.352	-	-	-	-	-	-	-	-	-	-
2.986	-	-	-	-	-	-	-	-	-	-
3.459	-	-	-	-	-	-	-	-	-	-
2.971	-	-	-	-	-	-	-	-	-	-
3.4	-	-	-	-	-	-	-	-	-	-
3.375	-	-	-	-	-	-	-	-	-	-
2.864	-	-	-	-	-	-	-	-	-	-
2.913	-	-	-	-	-	-	-	-	-	-
3.324	-	-	-	-	-	-	-	-	-	-
2.942	-	-	-	-	-	-	-	-	-	-
3.014	-	-	-	-	-	-	-	-	-	-

Continues on next page

Table S12 – *Continued from previous page*

C-H	H-O	C-O	O-O	C-C	H-N	C-N	N-O	H-Z	C-Zn	O-Zn
3.433	-	-	-	-	-	-	-	-	-	-
3.3	-	-	-	-	-	-	-	-	-	-
3.348	-	-	-	-	-	-	-	-	-	-
Frame number : 8000										
3.424	3.489	-	-	3.404	-	-	-	-	-	-
3.327	2.769	-	-	3.426	-	-	-	-	-	-
3.494	2.961	-	-	3.498	-	-	-	-	-	-
3.475	3.294	-	-	3.302	-	-	-	-	-	-
3.188	-	-	-	3.459	-	-	-	-	-	-
3.091	-	-	-	3.453	-	-	-	-	-	-
3.492	-	-	-	3.17	-	-	-	-	-	-
3.402	-	-	-	3.35	-	-	-	-	-	-
3.481	-	-	-	3.186	-	-	-	-	-	-
3.429	-	-	-	-	-	-	-	-	-	-
3.481	-	-	-	-	-	-	-	-	-	-
3.235	-	-	-	-	-	-	-	-	-	-
3.242	-	-	-	-	-	-	-	-	-	-
3.266	-	-	-	-	-	-	-	-	-	-
3.075	-	-	-	-	-	-	-	-	-	-
3.113	-	-	-	-	-	-	-	-	-	-
2.956	-	-	-	-	-	-	-	-	-	-
3.08	-	-	-	-	-	-	-	-	-	-
2.657	-	-	-	-	-	-	-	-	-	-
3.339	-	-	-	-	-	-	-	-	-	-

Continues on next page

Table S12 – *Continued from previous page*

C-H	H-O	C-O	O-O	C-C	H-N	C-N	N-O	H-Z	C-Zn	O-Zn
3.368	-	-	-	-	-	-	-	-	-	-
3.484	-	-	-	-	-	-	-	-	-	-
3.464	-	-	-	-	-	-	-	-	-	-
2.649	-	-	-	-	-	-	-	-	-	-
3.347	-	-	-	-	-	-	-	-	-	-
2.524	-	-	-	-	-	-	-	-	-	-
2.439	-	-	-	-	-	-	-	-	-	-
2.979	-	-	-	-	-	-	-	-	-	-
3.211	-	-	-	-	-	-	-	-	-	-
3.389	-	-	-	-	-	-	-	-	-	-
3.436	-	-	-	-	-	-	-	-	-	-
3.225	-	-	-	-	-	-	-	-	-	-
3.024	-	-	-	-	-	-	-	-	-	-
3.105	-	-	-	-	-	-	-	-	-	-
3.188	-	-	-	-	-	-	-	-	-	-
2.915	-	-	-	-	-	-	-	-	-	-
3.241	-	-	-	-	-	-	-	-	-	-
Frame number : 9000										
3.476	3.343	3.492	-	-	-	-	-	-	-	-
3.47	2.765	-	-	-	-	-	-	-	-	-
3.411	-	-	-	-	-	-	-	-	-	-
3.104	-	-	-	-	-	-	-	-	-	-
3.449	-	-	-	-	-	-	-	-	-	-
2.949	-	-	-	-	-	-	-	-	-	-

Continues on next page

Table S12 – *Continued from previous page*

C-H	H-O	C-O	O-O	C-C	H-N	C-N	N-O	H-Z	C-Zn	O-Zn
3.454	-	-	-	-	-	-	-	-	-	-
3.175	-	-	-	-	-	-	-	-	-	-
3.231	-	-	-	-	-	-	-	-	-	-
3.49	-	-	-	-	-	-	-	-	-	-
3.47	-	-	-	-	-	-	-	-	-	-
2.848	-	-	-	-	-	-	-	-	-	-
3.253	-	-	-	-	-	-	-	-	-	-
Frame number : 10000										
3.082	3.169	3.343	-	3.218	-	-	-	-	-	-
3.279	2.831	3.445	-	3.481	-	-	-	-	-	-
3.46	2.874	3.498	-	3.38	-	-	-	-	-	-
3.336	3.279	-	-	-	-	-	-	-	-	-
3.112	-	-	-	-	-	-	-	-	-	-
3.403	-	-	-	-	-	-	-	-	-	-
2.958	-	-	-	-	-	-	-	-	-	-
3.24	-	-	-	-	-	-	-	-	-	-
3.241	-	-	-	-	-	-	-	-	-	-
3.487	-	-	-	-	-	-	-	-	-	-
3.434	-	-	-	-	-	-	-	-	-	-
3.419	-	-	-	-	-	-	-	-	-	-
3.315	-	-	-	-	-	-	-	-	-	-
3.299	-	-	-	-	-	-	-	-	-	-
3.134	-	-	-	-	-	-	-	-	-	-

Table S13 Intermolecular distances (r < 3.5 Å) for surface ZnPW⊃-2-Styrene interactions computed for every 1000 molecular dynamic frames.

C-H	H-O	C-O	O-O	C-C	H-N	C-N	N-O	H-Z	C-Zn	O-Zn
Frame number : 0										
3.149	2.427	3.197	-	3.405	-	-	-	3.424	-	-
3.215	3.057	3.431	-	3.486	-	-	-	-	-	-
3.399	2.617	-	-	-	-	-	-	-	-	-
3.467	2.949	-	-	-	-	-	-	-	-	-
3.292	3.301	-	-	-	-	-	-	-	-	-
3.453	3.427	-	-	-	-	-	-	-	-	-
3.412	-	-	-	-	-	-	-	-	-	-
3.493	-	-	-	-	-	-	-	-	-	-
3.027	-	-	-	-	-	-	-	-	-	-
3.316	-	-	-	-	-	-	-	-	-	-
3.495	-	-	-	-	-	-	-	-	-	-
3.351	-	-	-	-	-	-	-	-	-	-
2.978	-	-	-	-	-	-	-	-	-	-
3.28	-	-	-	-	-	-	-	-	-	-
3.24	-	-	-	-	-	-	-	-	-	-
3.24	-	-	-	-	-	-	-	-	-	-
3.07	-	-	-	-	-	-	-	-	-	-
2.846	-	-	-	-	-	-	-	-	-	-
3.111	-	-	-	-	-	-	-	-	-	-
2.88	-	-	-	-	-	-	-	-	-	-
3.279	-	-	-	-	-	-	-	-	-	-

Continues on next page

Table S13 – *Continued from previous page*

C-H	H-O	C-O	O-O	C-C	H-N	C-N	N-O	H-Z	C-Zn	O-Zn
2.945	-	-	-	-	-	-	-	-	-	-
2.957	-	-	-	-	-	-	-	-	-	-
3.216	-	-	-	-	-	-	-	-	-	-
3.011	-	-	-	-	-	-	-	-	-	-
3.361	-	-	-	-	-	-	-	-	-	-
3.465	-	-	-	-	-	-	-	-	-	-
3.461	-	-	-	-	-	-	-	-	-	-
3.462	-	-	-	-	-	-	-	-	-	-
3.417	-	-	-	-	-	-	-	-	-	-
3.441	-	-	-	-	-	-	-	-	-	-
3.392	-	-	-	-	-	-	-	-	-	-
Frame number : 1000										
2.866	3.353	3.347	-	3.446	-	-	-	-	-	-
3.443	3.051	-	-	3.499	-	-	-	-	-	-
3.361	3.066	-	-	3.137	-	-	-	-	-	-
3.277	2.899	-	-	3.008	-	-	-	-	-	-
3.403	-	-	-	2.951	-	-	-	-	-	-
2.905	-	-	-	3.399	-	-	-	-	-	-
2.666	-	-	-	-	-	-	-	-	-	-
3.195	-	-	-	-	-	-	-	-	-	-
3.41	-	-	-	-	-	-	-	-	-	-
3.397	-	-	-	-	-	-	-	-	-	-
3.235	-	-	-	-	-	-	-	-	-	-
3.446	-	-	-	-	-	-	-	-	-	-

Continues on next page

Table S13 – *Continued from previous page*

C-H	H-O	C-O	O-O	C-C	H-N	C-N	N-O	H-Z	C-Zn	O-Zn
3.293	-	-	-	-	-	-	-	-	-	-
2.725	-	-	-	-	-	-	-	-	-	-
3.184	-	-	-	-	-	-	-	-	-	-
3.227	-	-	-	-	-	-	-	-	-	-
2.742	-	-	-	-	-	-	-	-	-	-
3.443	-	-	-	-	-	-	-	-	-	-
2.712	-	-	-	-	-	-	-	-	-	-
3.366	-	-	-	-	-	-	-	-	-	-
3.071	-	-	-	-	-	-	-	-	-	-
3.456	-	-	-	-	-	-	-	-	-	-
Frame number : 2000										
3.229	2.386	3.244	-	3.427	-	-	-	3.211	-	-
3.296	3.119	3.454	-	-	-	-	-	-	-	-
3.474	2.891	3.418	-	-	-	-	-	-	-	-
3.472	3.164	-	-	-	-	-	-	-	-	-
3.103	3.266	-	-	-	-	-	-	-	-	-
3.214	3.285	-	-	-	-	-	-	-	-	-
3.22	-	-	-	-	-	-	-	-	-	-
3.096	-	-	-	-	-	-	-	-	-	-
3.242	-	-	-	-	-	-	-	-	-	-
2.752	-	-	-	-	-	-	-	-	-	-
3.212	-	-	-	-	-	-	-	-	-	-
2.816	-	-	-	-	-	-	-	-	-	-
2.906	-	-	-	-	-	-	-	-	-	-

Continues on next page

Table S13 – *Continued from previous page*

C-H	H-O	C-O	O-O	C-C	H-N	C-N	N-O	H-Z	C-Zn	O-Zn
3.334	-	-	-	-	-	-	-	-	-	-
2.66	-	-	-	-	-	-	-	-	-	-
3.166	-	-	-	-	-	-	-	-	-	-
3.194	-	-	-	-	-	-	-	-	-	-
3.353	-	-	-	-	-	-	-	-	-	-
3.442	-	-	-	-	-	-	-	-	-	-
3.348	-	-	-	-	-	-	-	-	-	-
3.04	-	-	-	-	-	-	-	-	-	-
3.145	-	-	-	-	-	-	-	-	-	-
2.972	-	-	-	-	-	-	-	-	-	-
3.45	-	-	-	-	-	-	-	-	-	-
3.288	-	-	-	-	-	-	-	-	-	-
3.485	-	-	-	-	-	-	-	-	-	-
3.25	-	-	-	-	-	-	-	-	-	-
3.237	-	-	-	-	-	-	-	-	-	-
3.18	-	-	-	-	-	-	-	-	-	-
2.831	-	-	-	-	-	-	-	-	-	-
3.131	-	-	-	-	-	-	-	-	-	-
Frame number : 3000										
3.128	2.279	2.974	-	-	-	-	-	3.197	-	-
2.897	2.92	3.485	-	-	-	-	-	-	-	-
3.376	3.374	-	-	-	-	-	-	-	-	-
3.459	2.986	-	-	-	-	-	-	-	-	-
3.116	3.122	-	-	-	-	-	-	-	-	-

Continues on next page

Table S13 – *Continued from previous page*

C-H	H-O	C-O	O-O	C-C	H-N	C-N	N-O	H-Z	C-Zn	O-Zn
3.377	-	-	-	-	-	-	-	-	-	-
3.274	-	-	-	-	-	-	-	-	-	-
2.861	-	-	-	-	-	-	-	-	-	-
2.853	-	-	-	-	-	-	-	-	-	-
2.54	-	-	-	-	-	-	-	-	-	-
3.47	-	-	-	-	-	-	-	-	-	-
3.355	-	-	-	-	-	-	-	-	-	-
2.974	-	-	-	-	-	-	-	-	-	-
2.96	-	-	-	-	-	-	-	-	-	-
3.488	-	-	-	-	-	-	-	-	-	-
2.75	-	-	-	-	-	-	-	-	-	-
2.861	-	-	-	-	-	-	-	-	-	-
3.411	-	-	-	-	-	-	-	-	-	-
3.435	-	-	-	-	-	-	-	-	-	-
3.388	-	-	-	-	-	-	-	-	-	-
3.234	-	-	-	-	-	-	-	-	-	-
2.911	-	-	-	-	-	-	-	-	-	-
2.963	-	-	-	-	-	-	-	-	-	-
3.365	-	-	-	-	-	-	-	-	-	-
Frame number : 4000										
2.921	3.007	3.315	-	3.293	-	-	-	-	-	-
2.766	2.575	3.292	-	3.455	-	-	-	-	-	-
3.127	3.325	-	-	3.257	-	-	-	-	-	-
3.314	3.441	-	-	-	-	-	-	-	-	-

Continues on next page

Table S13 – *Continued from previous page*

C-H	H-O	C-O	O-O	C-C	H-N	C-N	N-O	H-Z	C-Zn	O-Zn
3.161	2.995	-	-	-	-	-	-	-	-	-
3.24	2.883	-	-	-	-	-	-	-	-	-
3.435	-	-	-	-	-	-	-	-	-	-
2.744	-	-	-	-	-	-	-	-	-	-
3.332	-	-	-	-	-	-	-	-	-	-
3.257	-	-	-	-	-	-	-	-	-	-
3.164	-	-	-	-	-	-	-	-	-	-
3.097	-	-	-	-	-	-	-	-	-	-
3.221	-	-	-	-	-	-	-	-	-	-
3.371	-	-	-	-	-	-	-	-	-	-
3.384	-	-	-	-	-	-	-	-	-	-
3.491	-	-	-	-	-	-	-	-	-	-
3.163	-	-	-	-	-	-	-	-	-	-
2.615	-	-	-	-	-	-	-	-	-	-
2.504	-	-	-	-	-	-	-	-	-	-
2.917	-	-	-	-	-	-	-	-	-	-
3.4	-	-	-	-	-	-	-	-	-	-
3.276	-	-	-	-	-	-	-	-	-	-
Frame number : 5000										
3.155	3.259	3.26	-	3.47	-	-	-	-	-	-
3.237	2.494	3.228	-	3.491	-	-	-	-	-	-
3.171	3.176	3.19	-	-	-	-	-	-	-	-
3.265	2.873	3.206	-	-	-	-	-	-	-	-
3.396	2.572	3.373	-	-	-	-	-	-	-	-

Continues on next page

Table S13 – *Continued from previous page*

C-H	H-O	C-O	O-O	C-C	H-N	C-N	N-O	H-Z	C-Zn	O-Zn
3.224	2.509	-	-	-	-	-	-	-	-	-
3.411	3.481	-	-	-	-	-	-	-	-	-
2.96	3.013	-	-	-	-	-	-	-	-	-
3.246	3.144	-	-	-	-	-	-	-	-	-
2.99	3.034	-	-	-	-	-	-	-	-	-
3.189	2.647	-	-	-	-	-	-	-	-	-
3.171	-	-	-	-	-	-	-	-	-	-
2.832	-	-	-	-	-	-	-	-	-	-
3.013	-	-	-	-	-	-	-	-	-	-
3.316	-	-	-	-	-	-	-	-	-	-
3.182	-	-	-	-	-	-	-	-	-	-
3.456	-	-	-	-	-	-	-	-	-	-
3.051	-	-	-	-	-	-	-	-	-	-
3.468	-	-	-	-	-	-	-	-	-	-
3.479	-	-	-	-	-	-	-	-	-	-
3.037	-	-	-	-	-	-	-	-	-	-
3.134	-	-	-	-	-	-	-	-	-	-
3.283	-	-	-	-	-	-	-	-	-	-
3.462	-	-	-	-	-	-	-	-	-	-
2.766	-	-	-	-	-	-	-	-	-	-
2.701	-	-	-	-	-	-	-	-	-	-
2.953	-	-	-	-	-	-	-	-	-	-
2.967	-	-	-	-	-	-	-	-	-	-
3.047	-	-	-	-	-	-	-	-	-	-

Continues on next page

Table S13 – *Continued from previous page*

C-H	H-O	C-O	O-O	C-C	H-N	C-N	N-O	H-Z	C-Zn	O-Zn
3.092	-	-	-	-	-	-	-	-	-	-
Frame number : 6000										
3.063	3.26	3.331	-	3.47	-	-	-	-	-	-
3.486	2.813	3.36	-	3.333	-	-	-	-	-	-
2.948	3.154	3.49	-	3.389	-	-	-	-	-	-
3.233	3.246	3.256	-	3.286	-	-	-	-	-	-
3.448	3.13	-	-	3.428	-	-	-	-	-	-
2.977	2.714	-	-	3.399	-	-	-	-	-	-
3.172	2.714	-	-	3.274	-	-	-	-	-	-
3.373	3.206	-	-	3.438	-	-	-	-	-	-
2.734	-	-	-	3.272	-	-	-	-	-	-
2.567	-	-	-	3.067	-	-	-	-	-	-
3.059	-	-	-	3.309	-	-	-	-	-	-
3.395	-	-	-	3.272	-	-	-	-	-	-
3.449	-	-	-	-	-	-	-	-	-	-
3.407	-	-	-	-	-	-	-	-	-	-
3.061	-	-	-	-	-	-	-	-	-	-
3.039	-	-	-	-	-	-	-	-	-	-
2.994	-	-	-	-	-	-	-	-	-	-
3.436	-	-	-	-	-	-	-	-	-	-
3.191	-	-	-	-	-	-	-	-	-	-
3.227	-	-	-	-	-	-	-	-	-	-
3.293	-	-	-	-	-	-	-	-	-	-
2.917	-	-	-	-	-	-	-	-	-	-

Continues on next page

Table S13 – *Continued from previous page*

C-H	H-O	C-O	O-O	C-C	H-N	C-N	N-O	H-Z	C-Zn	O-Zn
3.301	-	-	-	-	-	-	-	-	-	-
3.337	-	-	-	-	-	-	-	-	-	-
3.06	-	-	-	-	-	-	-	-	-	-
3.02	-	-	-	-	-	-	-	-	-	-
3.448	-	-	-	-	-	-	-	-	-	-
3.266	-	-	-	-	-	-	-	-	-	-
3.108	-	-	-	-	-	-	-	-	-	-
3.296	-	-	-	-	-	-	-	-	-	-
3.032	-	-	-	-	-	-	-	-	-	-
3.239	-	-	-	-	-	-	-	-	-	-
3.299	-	-	-	-	-	-	-	-	-	-
2.625	-	-	-	-	-	-	-	-	-	-
3.366	-	-	-	-	-	-	-	-	-	-
3.369	-	-	-	-	-	-	-	-	-	-
3.18	-	-	-	-	-	-	-	-	-	-
Frame number : 7000										
3.195	3.424	-	-	3.161	-	-	-	-	-	-
3.345	2.988	-	-	3.251	-	-	-	-	-	-
3.117	3.103	-	-	3.455	-	-	-	-	-	-
3.266	3.005	-	-	3.23	-	-	-	-	-	-
2.785	3.232	-	-	-	-	-	-	-	-	-
3.199	-	-	-	-	-	-	-	-	-	-
2.996	-	-	-	-	-	-	-	-	-	-
2.895	-	-	-	-	-	-	-	-	-	-

Continues on next page

Table S13 – *Continued from previous page*

C-H	H-O	C-O	O-O	C-C	H-N	C-N	N-O	H-Z	C-Zn	O-Zn
3.069	-	-	-	-	-	-	-	-	-	-
3.249	-	-	-	-	-	-	-	-	-	-
3.349	-	-	-	-	-	-	-	-	-	-
3.406	-	-	-	-	-	-	-	-	-	-
3.351	-	-	-	-	-	-	-	-	-	-
3.098	-	-	-	-	-	-	-	-	-	-
3.42	-	-	-	-	-	-	-	-	-	-
3.204	-	-	-	-	-	-	-	-	-	-
3.12	-	-	-	-	-	-	-	-	-	-
3.47	-	-	-	-	-	-	-	-	-	-
3.257	-	-	-	-	-	-	-	-	-	-
3.022	-	-	-	-	-	-	-	-	-	-
3.144	-	-	-	-	-	-	-	-	-	-
3.052	-	-	-	-	-	-	-	-	-	-
3.468	-	-	-	-	-	-	-	-	-	-
2.927	-	-	-	-	-	-	-	-	-	-
3.18	-	-	-	-	-	-	-	-	-	-
3.063	-	-	-	-	-	-	-	-	-	-
2.552	-	-	-	-	-	-	-	-	-	-
3.488	-	-	-	-	-	-	-	-	-	-
3.078	-	-	-	-	-	-	-	-	-	-
3.169	-	-	-	-	-	-	-	-	-	-
2.807	-	-	-	-	-	-	-	-	-	-
2.433	-	-	-	-	-	-	-	-	-	-

Continues on next page

Table S13 – *Continued from previous page*

C-H	H-O	C-O	O-O	C-C	H-N	C-N	N-O	H-Z	C-Zn	O-Zn
2.987	-	-	-	-	-	-	-	-	-	-
3.34	-	-	-	-	-	-	-	-	-	-
Frame number : 8000										
3.395	-	-	-	3.14	-	-	-	-	-	-
2.871	-	-	-	3.341	-	-	-	-	-	-
3.401	-	-	-	3.38	-	-	-	-	-	-
3.327	-	-	-	3.377	-	-	-	-	-	-
2.888	-	-	-	-	-	-	-	-	-	-
3.435	-	-	-	-	-	-	-	-	-	-
3.102	-	-	-	-	-	-	-	-	-	-
2.826	-	-	-	-	-	-	-	-	-	-
2.902	-	-	-	-	-	-	-	-	-	-
3.455	-	-	-	-	-	-	-	-	-	-
3.096	-	-	-	-	-	-	-	-	-	-
3.194	-	-	-	-	-	-	-	-	-	-
3.276	-	-	-	-	-	-	-	-	-	-
3.474	-	-	-	-	-	-	-	-	-	-
3.371	-	-	-	-	-	-	-	-	-	-
3.419	-	-	-	-	-	-	-	-	-	-
3.438	-	-	-	-	-	-	-	-	-	-
3.241	-	-	-	-	-	-	-	-	-	-
3.227	-	-	-	-	-	-	-	-	-	-
3.32	-	-	-	-	-	-	-	-	-	-
3.065	-	-	-	-	-	-	-	-	-	-

Continues on next page

Table S13 – *Continued from previous page*

C-H	H-O	C-O	O-O	C-C	H-N	C-N	N-O	H-Z	C-Zn	O-Zn
2.944	-	-	-	-	-	-	-	-	-	-
3.155	-	-	-	-	-	-	-	-	-	-
2.884	-	-	-	-	-	-	-	-	-	-
3.113	-	-	-	-	-	-	-	-	-	-
3.465	-	-	-	-	-	-	-	-	-	-
3.431	-	-	-	-	-	-	-	-	-	-
3.458	-	-	-	-	-	-	-	-	-	-
Frame number : 9000										
3.366	2.621	-	-	3.5	-	-	-	-	-	-
3.314	3.167	-	-	3.295	-	-	-	-	-	-
2.92	3.308	-	-	3.49	-	-	-	-	-	-
3.132	3.273	-	-	3.445	-	-	-	-	-	-
2.983	3.428	-	-	3.433	-	-	-	-	-	-
3.05	-	-	-	3.242	-	-	-	-	-	-
2.905	-	-	-	3.437	-	-	-	-	-	-
3.118	-	-	-	3.159	-	-	-	-	-	-
3.25	-	-	-	2.854	-	-	-	-	-	-
3.292	-	-	-	3.412	-	-	-	-	-	-
3.469	-	-	-	3.495	-	-	-	-	-	-
3.109	-	-	-	-	-	-	-	-	-	-
3.437	-	-	-	-	-	-	-	-	-	-
3.485	-	-	-	-	-	-	-	-	-	-
2.939	-	-	-	-	-	-	-	-	-	-
3.425	-	-	-	-	-	-	-	-	-	-

Continues on next page

Table S13 – *Continued from previous page*

C-H	H-O	C-O	O-O	C-C	H-N	C-N	N-O	H-Z	C-Zn	O-Zn
3.239	-	-	-	-	-	-	-	-	-	-
2.477	-	-	-	-	-	-	-	-	-	-
2.801	-	-	-	-	-	-	-	-	-	-
3.146	-	-	-	-	-	-	-	-	-	-
3.435	-	-	-	-	-	-	-	-	-	-
3.424	-	-	-	-	-	-	-	-	-	-
2.842	-	-	-	-	-	-	-	-	-	-
3.327	-	-	-	-	-	-	-	-	-	-
3.272	-	-	-	-	-	-	-	-	-	-
2.852	-	-	-	-	-	-	-	-	-	-
3.363	-	-	-	-	-	-	-	-	-	-
3.269	-	-	-	-	-	-	-	-	-	-
3.495	-	-	-	-	-	-	-	-	-	-
3.241	-	-	-	-	-	-	-	-	-	-
3.408	-	-	-	-	-	-	-	-	-	-
Frame number : 10000										
3.446	3.242	-	-	3.428	-	-	-	-	-	-
3.027	3.272	-	-	3.459	-	-	-	-	-	-
3.405	2.988	-	-	3.458	-	-	-	-	-	-
2.954	3.096	-	-	3.262	-	-	-	-	-	-
3.355	3.49	-	-	3.488	-	-	-	-	-	-
3.419	-	-	-	3.496	-	-	-	-	-	-
3.14	-	-	-	3.339	-	-	-	-	-	-
3.408	-	-	-	3.472	-	-	-	-	-	-

Continues on next page

Table S13 – *Continued from previous page*

C-H	H-O	C-O	O-O	C-C	H-N	C-N	N-O	H-Z	C-Zn	O-Zn
3.496	-	-	-	3.37	-	-	-	-	-	-
2.617	-	-	-	3.45	-	-	-	-	-	-
3.201	-	-	-	3.499	-	-	-	-	-	-
3.496	-	-	-	3.117	-	-	-	-	-	-
2.869	-	-	-	3.425	-	-	-	-	-	-
3.486	-	-	-	-	-	-	-	-	-	-
3.453	-	-	-	-	-	-	-	-	-	-
3.035	-	-	-	-	-	-	-	-	-	-
3.038	-	-	-	-	-	-	-	-	-	-
3.444	-	-	-	-	-	-	-	-	-	-
3.26	-	-	-	-	-	-	-	-	-	-
3.172	-	-	-	-	-	-	-	-	-	-
3.078	-	-	-	-	-	-	-	-	-	-
3.122	-	-	-	-	-	-	-	-	-	-
3.086	-	-	-	-	-	-	-	-	-	-
3.29	-	-	-	-	-	-	-	-	-	-
3.19	-	-	-	-	-	-	-	-	-	-
3.476	-	-	-	-	-	-	-	-	-	-
3.23	-	-	-	-	-	-	-	-	-	-
3.248	-	-	-	-	-	-	-	-	-	-
3.28	-	-	-	-	-	-	-	-	-	-
3.274	-	-	-	-	-	-	-	-	-	-
3.495	-	-	-	-	-	-	-	-	-	-
3.323	-	-	-	-	-	-	-	-	-	-

Continues on next page

Table S13 – *Continued from previous page*

C-H	H-O	C-O	O-O	C-C	H-N	C-N	N-O	H-Z	C-Zn	O-Zn
3.482	-	-	-	-	-	-	-	-	-	-

Table S14 Intermolecular distances (r < 3.5 Å) for surface ZnPW⊃-3-Styrene interactions computed for every 1000 molecular dynamic frames.

C-H	H-O	C-O	O-O	C-C	H-N	C-N	N-O	H-Z	C-Zn	O-Zn
Frame number : 0										
3.456	2.498	3.283	-	3.442	-	-	-	-	-	-
3.011	2.996	3.396	-	3.489	-	-	-	-	-	-
3.135	3.342	3.462	-	-	-	-	-	-	-	-
2.902	3.047	3.301	-	-	-	-	-	-	-	-
3.041	2.865	-	-	-	-	-	-	-	-	-
3.179	3.243	-	-	-	-	-	-	-	-	-
3.242	3.339	-	-	-	-	-	-	-	-	-
3.194	3.247	-	-	-	-	-	-	-	-	-
3.081	3.011	-	-	-	-	-	-	-	-	-
3.09	3.488	-	-	-	-	-	-	-	-	-
2.805	3.193	-	-	-	-	-	-	-	-	-
3.285	3.434	-	-	-	-	-	-	-	-	-
3.244	-	-	-	-	-	-	-	-	-	-
3.052	-	-	-	-	-	-	-	-	-	-
3.228	-	-	-	-	-	-	-	-	-	-
3.193	-	-	-	-	-	-	-	-	-	-

Continues on next page

Table S14 – *Continued from previous page*

C-H	H-O	C-O	O-O	C-C	H-N	C-N	N-O	H-Z	C-Zn	O-Zn
3.225	-	-	-	-	-	-	-	-	-	-
3.339	-	-	-	-	-	-	-	-	-	-
3.478	-	-	-	-	-	-	-	-	-	-
2.984	-	-	-	-	-	-	-	-	-	-
2.888	-	-	-	-	-	-	-	-	-	-
3.262	-	-	-	-	-	-	-	-	-	-
3.022	-	-	-	-	-	-	-	-	-	-
2.671	-	-	-	-	-	-	-	-	-	-
3.128	-	-	-	-	-	-	-	-	-	-
3.378	-	-	-	-	-	-	-	-	-	-
2.948	-	-	-	-	-	-	-	-	-	-
3.276	-	-	-	-	-	-	-	-	-	-
3.253	-	-	-	-	-	-	-	-	-	-
3.474	-	-	-	-	-	-	-	-	-	-
3.449	-	-	-	-	-	-	-	-	-	-
3.034	-	-	-	-	-	-	-	-	-	-
3.377	-	-	-	-	-	-	-	-	-	-
3.113	-	-	-	-	-	-	-	-	-	-
3.239	-	-	-	-	-	-	-	-	-	-
3.314	-	-	-	-	-	-	-	-	-	-
3.326	-	-	-	-	-	-	-	-	-	-
3.247	-	-	-	-	-	-	-	-	-	-
3.098	-	-	-	-	-	-	-	-	-	-
3.091	-	-	-	-	-	-	-	-	-	-

Continues on next page

Table S14 – *Continued from previous page*

C-H	H-O	C-O	O-O	C-C	H-N	C-N	N-O	H-Z	C-Zn	O-Zn
3.137	-	-	-	-	-	-	-	-	-	-
3.33	-	-	-	-	-	-	-	-	-	-
3.451	-	-	-	-	-	-	-	-	-	-
3.039	-	-	-	-	-	-	-	-	-	-
3.411	-	-	-	-	-	-	-	-	-	-
3.11	-	-	-	-	-	-	-	-	-	-
2.927	-	-	-	-	-	-	-	-	-	-
3.424	-	-	-	-	-	-	-	-	-	-
Frame number : 1000										
3.041	2.948	-	-	3.418	-	-	-	-	-	-
3.465	3.492	-	-	3.004	-	-	-	-	-	-
3.385	-	-	-	3.332	-	-	-	-	-	-
3.484	-	-	-	3.372	-	-	-	-	-	-
3.259	-	-	-	-	-	-	-	-	-	-
3.498	-	-	-	-	-	-	-	-	-	-
3.203	-	-	-	-	-	-	-	-	-	-
3.469	-	-	-	-	-	-	-	-	-	-
3.383	-	-	-	-	-	-	-	-	-	-
3.021	-	-	-	-	-	-	-	-	-	-
3.347	-	-	-	-	-	-	-	-	-	-
3.054	-	-	-	-	-	-	-	-	-	-
2.89	-	-	-	-	-	-	-	-	-	-
2.727	-	-	-	-	-	-	-	-	-	-
2.913	-	-	-	-	-	-	-	-	-	-

Continues on next page

Table S14 – *Continued from previous page*

C-H	H-O	C-O	O-O	C-C	H-N	C-N	N-O	H-Z	C-Zn	O-Zn
3.417	-	-	-	-	-	-	-	-	-	-
3.304	-	-	-	-	-	-	-	-	-	-
3.017	-	-	-	-	-	-	-	-	-	-
3.443	-	-	-	-	-	-	-	-	-	-
2.988	-	-	-	-	-	-	-	-	-	-
2.944	-	-	-	-	-	-	-	-	-	-
3.098	-	-	-	-	-	-	-	-	-	-
3.49	-	-	-	-	-	-	-	-	-	-
3.109	-	-	-	-	-	-	-	-	-	-
3.152	-	-	-	-	-	-	-	-	-	-
3.139	-	-	-	-	-	-	-	-	-	-
3.479	-	-	-	-	-	-	-	-	-	-
3.313	-	-	-	-	-	-	-	-	-	-
2.044	-	-	-	-	-	-	-	-	-	-
2.272	-	-	-	-	-	-	-	-	-	-
2.91	-	-	-	-	-	-	-	-	-	-
2.51	-	-	-	-	-	-	-	-	-	-
3.389	-	-	-	-	-	-	-	-	-	-
3.211	-	-	-	-	-	-	-	-	-	-
Frame number : 2000										
3.359	2.821	3.43	-	3.438	-	-	-	3.215	-	-
3.028	3.083	3.09	-	3.312	-	-	-	-	-	-
3.498	2.743	3.089	-	3.446	-	-	-	-	-	-
2.621	2.726	3.096	-	3.373	-	-	-	-	-	-

Continues on next page

Table S14 – *Continued from previous page*

C-H	H-O	C-O	O-O	C-C	H-N	C-N	N-O	H-Z	C-Zn	O-Zn
3.156	2.06	-	-	3.31	-	-	-	-	-	-
2.485	3.17	-	-	3.423	-	-	-	-	-	-
3.003	2.545	-	-	3.445	-	-	-	-	-	-
3.246	3.454	-	-	3.236	-	-	-	-	-	-
2.69	-	-	-	3.407	-	-	-	-	-	-
2.976	-	-	-	3.425	-	-	-	-	-	-
2.447	-	-	-	3.459	-	-	-	-	-	-
3.375	-	-	-	3.467	-	-	-	-	-	-
2.77	-	-	-	3.489	-	-	-	-	-	-
3.389	-	-	-	3.366	-	-	-	-	-	-
2.858	-	-	-	3.312	-	-	-	-	-	-
3.472	-	-	-	-	-	-	-	-	-	-
3.496	-	-	-	-	-	-	-	-	-	-
2.921	-	-	-	-	-	-	-	-	-	-
3.413	-	-	-	-	-	-	-	-	-	-
2.935	-	-	-	-	-	-	-	-	-	-
3.339	-	-	-	-	-	-	-	-	-	-
2.992	-	-	-	-	-	-	-	-	-	-
2.858	-	-	-	-	-	-	-	-	-	-
3.381	-	-	-	-	-	-	-	-	-	-
3.35	-	-	-	-	-	-	-	-	-	-
3.44	-	-	-	-	-	-	-	-	-	-
3.23	-	-	-	-	-	-	-	-	-	-
2.86	-	-	-	-	-	-	-	-	-	-

Continues on next page

Table S14 – *Continued from previous page*

C-H	H-O	C-O	O-O	C-C	H-N	C-N	N-O	H-Z	C-Zn	O-Zn
3.299	-	-	-	-	-	-	-	-	-	-
3.285	-	-	-	-	-	-	-	-	-	-
3.443	-	-	-	-	-	-	-	-	-	-
3.353	-	-	-	-	-	-	-	-	-	-
2.663	-	-	-	-	-	-	-	-	-	-
2.754	-	-	-	-	-	-	-	-	-	-
3.087	-	-	-	-	-	-	-	-	-	-
3.457	-	-	-	-	-	-	-	-	-	-
3.26	-	-	-	-	-	-	-	-	-	-
2.657	-	-	-	-	-	-	-	-	-	-
3.075	-	-	-	-	-	-	-	-	-	-
3.465	-	-	-	-	-	-	-	-	-	-
3.33	-	-	-	-	-	-	-	-	-	-
3.319	-	-	-	-	-	-	-	-	-	-
3.046	-	-	-	-	-	-	-	-	-	-
3.143	-	-	-	-	-	-	-	-	-	-
3.076	-	-	-	-	-	-	-	-	-	-
3.051	-	-	-	-	-	-	-	-	-	-
3.486	-	-	-	-	-	-	-	-	-	-
3.085	-	-	-	-	-	-	-	-	-	-
3.451	-	-	-	-	-	-	-	-	-	-
3.409	-	-	-	-	-	-	-	-	-	-
Frame number : 3000										
3.468	2.875	3.425	-	3.482	-	-	-	3.315	-	-

Continues on next page

Table S14 – *Continued from previous page*

C-H	H-O	C-O	O-O	C-C	H-N	C-N	N-O	H-Z	C-Zn	O-Zn
3.132	3.199	2.829	-	3.108	-	-	-	-	-	-
3.388	3.155	3.179	-	3.323	-	-	-	-	-	-
3.392	2.708	-	-	3.286	-	-	-	-	-	-
2.966	2.949	-	-	3.472	-	-	-	-	-	-
3.363	1.969	-	-	3.301	-	-	-	-	-	-
3.1	3.34	-	-	3.493	-	-	-	-	-	-
3.425	3.127	-	-	3.452	-	-	-	-	-	-
3.225	3.469	-	-	3.08	-	-	-	-	-	-
3.128	2.204	-	-	3.184	-	-	-	-	-	-
3.436	-	-	-	3.274	-	-	-	-	-	-
3.058	-	-	-	3.496	-	-	-	-	-	-
2.383	-	-	-	3.442	-	-	-	-	-	-
3.45	-	-	-	3.267	-	-	-	-	-	-
2.93	-	-	-	3.342	-	-	-	-	-	-
2.294	-	-	-	3.311	-	-	-	-	-	-
3.041	-	-	-	3.439	-	-	-	-	-	-
3.12	-	-	-	3.232	-	-	-	-	-	-
2.743	-	-	-	3.388	-	-	-	-	-	-
3.451	-	-	-	3.289	-	-	-	-	-	-
3.416	-	-	-	3.43	-	-	-	-	-	-
3.232	-	-	-	-	-	-	-	-	-	-
3.359	-	-	-	-	-	-	-	-	-	-
3.487	-	-	-	-	-	-	-	-	-	-
3.11	-	-	-	-	-	-	-	-	-	-

Continues on next page

Table S14 – *Continued from previous page*

C-H	H-O	C-O	O-O	C-C	H-N	C-N	N-O	H-Z	C-Zn	O-Zn
3.478	-	-	-	-	-	-	-	-	-	-
3.326	-	-	-	-	-	-	-	-	-	-
3.221	-	-	-	-	-	-	-	-	-	-
3.448	-	-	-	-	-	-	-	-	-	-
3.5	-	-	-	-	-	-	-	-	-	-
3.424	-	-	-	-	-	-	-	-	-	-
3.1	-	-	-	-	-	-	-	-	-	-
3.438	-	-	-	-	-	-	-	-	-	-
3.07	-	-	-	-	-	-	-	-	-	-
3.422	-	-	-	-	-	-	-	-	-	-
3.467	-	-	-	-	-	-	-	-	-	-
3.11	-	-	-	-	-	-	-	-	-	-
3.453	-	-	-	-	-	-	-	-	-	-
2.927	-	-	-	-	-	-	-	-	-	-
3.428	-	-	-	-	-	-	-	-	-	-
3.491	-	-	-	-	-	-	-	-	-	-
3.005	-	-	-	-	-	-	-	-	-	-
3.47	-	-	-	-	-	-	-	-	-	-
3.244	-	-	-	-	-	-	-	-	-	-
3.318	-	-	-	-	-	-	-	-	-	-
3.103	-	-	-	-	-	-	-	-	-	-
3.398	-	-	-	-	-	-	-	-	-	-
3.226	-	-	-	-	-	-	-	-	-	-
2.721	-	-	-	-	-	-	-	-	-	-

Continues on next page

Table S14 – *Continued from previous page*

C-H	H-O	C-O	O-O	C-C	H-N	C-N	N-O	H-Z	C-Zn	O-Zn
2.957	-	-	-	-	-	-	-	-	-	-
2.936	-	-	-	-	-	-	-	-	-	-
2.974	-	-	-	-	-	-	-	-	-	-
3.413	-	-	-	-	-	-	-	-	-	-
3.062	-	-	-	-	-	-	-	-	-	-
3.344	-	-	-	-	-	-	-	-	-	-
Frame number : 4000										
3.477	2.666	3.477	-	3.301	-	-	-	3.323	-	-
3.054	3.384	3.401	-	3.415	-	-	-	-	-	-
2.871	3.127	3.163	-	3.262	-	-	-	-	-	-
3.343	2.765	2.884	-	3.204	-	-	-	-	-	-
2.937	3.344	-	-	3.294	-	-	-	-	-	-
3.023	3.483	-	-	3.418	-	-	-	-	-	-
3.176	2.641	-	-	3.446	-	-	-	-	-	-
3.168	3.339	-	-	3.411	-	-	-	-	-	-
3.173	2.757	-	-	3.417	-	-	-	-	-	-
2.796	3.278	-	-	3.289	-	-	-	-	-	-
3.344	2.554	-	-	2.976	-	-	-	-	-	-
3.368	2.966	-	-	3.439	-	-	-	-	-	-
3.276	2.842	-	-	3.347	-	-	-	-	-	-
3.192	-	-	-	3.111	-	-	-	-	-	-
3.293	-	-	-	3.302	-	-	-	-	-	-
3.12	-	-	-	-	-	-	-	-	-	-
2.643	-	-	-	-	-	-	-	-	-	-

Continues on next page

Table S14 – *Continued from previous page*

C-H	H-O	C-O	O-O	C-C	H-N	C-N	N-O	H-Z	C-Zn	O-Zn
2.631	-	-	-	-	-	-	-	-	-	-
2.881	-	-	-	-	-	-	-	-	-	-
3.076	-	-	-	-	-	-	-	-	-	-
3.392	-	-	-	-	-	-	-	-	-	-
2.763	-	-	-	-	-	-	-	-	-	-
3.141	-	-	-	-	-	-	-	-	-	-
3.452	-	-	-	-	-	-	-	-	-	-
3.176	-	-	-	-	-	-	-	-	-	-
3.126	-	-	-	-	-	-	-	-	-	-
2.625	-	-	-	-	-	-	-	-	-	-
2.817	-	-	-	-	-	-	-	-	-	-
3.256	-	-	-	-	-	-	-	-	-	-
3.262	-	-	-	-	-	-	-	-	-	-
3.029	-	-	-	-	-	-	-	-	-	-
2.419	-	-	-	-	-	-	-	-	-	-
2.682	-	-	-	-	-	-	-	-	-	-
3.45	-	-	-	-	-	-	-	-	-	-
3.468	-	-	-	-	-	-	-	-	-	-
3.269	-	-	-	-	-	-	-	-	-	-
3.154	-	-	-	-	-	-	-	-	-	-
3.481	-	-	-	-	-	-	-	-	-	-
Frame number : 5000										
3.023	3.492	3.46	-	3.267	-	-	-	-	-	-
3.483	3.091	3.083	-	3.429	-	-	-	-	-	-

Continues on next page

Table S14 – *Continued from previous page*

C-H	H-O	C-O	O-O	C-C	H-N	C-N	N-O	H-Z	C-Zn	O-Zn
3.489	3.23	-	-	-	-	-	-	-	-	-
3.38	3.382	-	-	-	-	-	-	-	-	-
3.417	2.771	-	-	-	-	-	-	-	-	-
3.261	3.212	-	-	-	-	-	-	-	-	-
2.883	3.149	-	-	-	-	-	-	-	-	-
3.05	2.334	-	-	-	-	-	-	-	-	-
3.273	2.854	-	-	-	-	-	-	-	-	-
3.09	-	-	-	-	-	-	-	-	-	-
3.202	-	-	-	-	-	-	-	-	-	-
3.353	-	-	-	-	-	-	-	-	-	-
3.34	-	-	-	-	-	-	-	-	-	-
3.406	-	-	-	-	-	-	-	-	-	-
3.489	-	-	-	-	-	-	-	-	-	-
3.074	-	-	-	-	-	-	-	-	-	-
3.471	-	-	-	-	-	-	-	-	-	-
3.195	-	-	-	-	-	-	-	-	-	-
3.447	-	-	-	-	-	-	-	-	-	-
3.474	-	-	-	-	-	-	-	-	-	-
3.393	-	-	-	-	-	-	-	-	-	-
3.017	-	-	-	-	-	-	-	-	-	-
2.985	-	-	-	-	-	-	-	-	-	-
3.338	-	-	-	-	-	-	-	-	-	-
3.397	-	-	-	-	-	-	-	-	-	-
3.377	-	-	-	-	-	-	-	-	-	-

Continues on next page

Table S14 – *Continued from previous page*

C-H	H-O	C-O	O-O	C-C	H-N	C-N	N-O	H-Z	C-Zn	O-Zn
2.958	-	-	-	-	-	-	-	-	-	-
3.303	-	-	-	-	-	-	-	-	-	-
2.909	-	-	-	-	-	-	-	-	-	-
3.115	-	-	-	-	-	-	-	-	-	-
3.479	-	-	-	-	-	-	-	-	-	-
3.231	-	-	-	-	-	-	-	-	-	-
3.179	-	-	-	-	-	-	-	-	-	-
2.713	-	-	-	-	-	-	-	-	-	-
3.251	-	-	-	-	-	-	-	-	-	-
2.732	-	-	-	-	-	-	-	-	-	-
3.128	-	-	-	-	-	-	-	-	-	-
2.205	-	-	-	-	-	-	-	-	-	-
2.435	-	-	-	-	-	-	-	-	-	-
3.365	-	-	-	-	-	-	-	-	-	-
2.846	-	-	-	-	-	-	-	-	-	-
Frame number : 6000										
3.255	3.42	3.468	-	3.366	-	-	-	-	-	-
2.968	3.062	-	-	3.398	-	-	-	-	-	-
2.931	3.271	-	-	3.482	-	-	-	-	-	-
3.126	3.468	-	-	3.4	-	-	-	-	-	-
3.301	3.254	-	-	3.38	-	-	-	-	-	-
3.397	2.939	-	-	-	-	-	-	-	-	-
3.035	3.103	-	-	-	-	-	-	-	-	-
3.22	3.494	-	-	-	-	-	-	-	-	-

Continues on next page

Table S14 – *Continued from previous page*

C-H	H-O	C-O	O-O	C-C	H-N	C-N	N-O	H-Z	C-Zn	O-Zn
3.448	3.164	-	-	-	-	-	-	-	-	-
3.108	3.01	-	-	-	-	-	-	-	-	-
2.997	2.919	-	-	-	-	-	-	-	-	-
2.869	-	-	-	-	-	-	-	-	-	-
3.237	-	-	-	-	-	-	-	-	-	-
3.111	-	-	-	-	-	-	-	-	-	-
3.037	-	-	-	-	-	-	-	-	-	-
3.486	-	-	-	-	-	-	-	-	-	-
3.095	-	-	-	-	-	-	-	-	-	-
3.271	-	-	-	-	-	-	-	-	-	-
3.043	-	-	-	-	-	-	-	-	-	-
2.906	-	-	-	-	-	-	-	-	-	-
3.014	-	-	-	-	-	-	-	-	-	-
3.295	-	-	-	-	-	-	-	-	-	-
3.478	-	-	-	-	-	-	-	-	-	-
3.37	-	-	-	-	-	-	-	-	-	-
2.884	-	-	-	-	-	-	-	-	-	-
3.416	-	-	-	-	-	-	-	-	-	-
3.293	-	-	-	-	-	-	-	-	-	-
3.413	-	-	-	-	-	-	-	-	-	-
2.635	-	-	-	-	-	-	-	-	-	-
2.827	-	-	-	-	-	-	-	-	-	-
3.036	-	-	-	-	-	-	-	-	-	-
2.824	-	-	-	-	-	-	-	-	-	-

Continues on next page

Table S14 – *Continued from previous page*

C-H	H-O	C-O	O-O	C-C	H-N	C-N	N-O	H-Z	C-Zn	O-Zn
2.516	-	-	-	-	-	-	-	-	-	-
2.599	-	-	-	-	-	-	-	-	-	-
3.43	-	-	-	-	-	-	-	-	-	-
3.225	-	-	-	-	-	-	-	-	-	-
3.405	-	-	-	-	-	-	-	-	-	-
2.873	-	-	-	-	-	-	-	-	-	-
2.977	-	-	-	-	-	-	-	-	-	-
3.039	-	-	-	-	-	-	-	-	-	-
3.232	-	-	-	-	-	-	-	-	-	-
3.43	-	-	-	-	-	-	-	-	-	-
3.126	-	-	-	-	-	-	-	-	-	-
3.222	-	-	-	-	-	-	-	-	-	-
3.242	-	-	-	-	-	-	-	-	-	-
2.654	-	-	-	-	-	-	-	-	-	-
2.872	-	-	-	-	-	-	-	-	-	-
2.841	-	-	-	-	-	-	-	-	-	-
3.318	-	-	-	-	-	-	-	-	-	-
Frame number : 7000										
2.566	3.378	3.365	-	3.304	-	-	-	2.79	-	-
3.163	3.024	3.355	-	3.454	-	-	-	-	-	-
3.372	2.485	3.104	-	3.479	-	-	-	-	-	-
3.312	3.146	3.284	-	3.33	-	-	-	-	-	-
3.395	2.918	3.33	-	3.257	-	-	-	-	-	-
2.752	2.307	-	-	-	-	-	-	-	-	-

Continues on next page

Table S14 – *Continued from previous page*

C-H	H-O	C-O	O-O	C-C	H-N	C-N	N-O	H-Z	C-Zn	O-Zn
3.376	2.37	-	-	-	-	-	-	-	-	-
3.464	2.832	-	-	-	-	-	-	-	-	-
3.225	3.403	-	-	-	-	-	-	-	-	-
2.804	-	-	-	-	-	-	-	-	-	-
2.905	-	-	-	-	-	-	-	-	-	-
3.195	-	-	-	-	-	-	-	-	-	-
2.739	-	-	-	-	-	-	-	-	-	-
3.258	-	-	-	-	-	-	-	-	-	-
3.474	-	-	-	-	-	-	-	-	-	-
3.105	-	-	-	-	-	-	-	-	-	-
3.365	-	-	-	-	-	-	-	-	-	-
3.335	-	-	-	-	-	-	-	-	-	-
3.389	-	-	-	-	-	-	-	-	-	-
3.012	-	-	-	-	-	-	-	-	-	-
2.55	-	-	-	-	-	-	-	-	-	-
3.295	-	-	-	-	-	-	-	-	-	-
2.717	-	-	-	-	-	-	-	-	-	-
3.288	-	-	-	-	-	-	-	-	-	-
2.654	-	-	-	-	-	-	-	-	-	-
3.186	-	-	-	-	-	-	-	-	-	-
2.659	-	-	-	-	-	-	-	-	-	-
3.363	-	-	-	-	-	-	-	-	-	-
3.178	-	-	-	-	-	-	-	-	-	-
3.325	-	-	-	-	-	-	-	-	-	-

Continues on next page

Table S14 – *Continued from previous page*

C-H	H-O	C-O	O-O	C-C	H-N	C-N	N-O	H-Z	C-Zn	O-Zn
2.956	-	-	-	-	-	-	-	-	-	-
3.236	-	-	-	-	-	-	-	-	-	-
2.908	-	-	-	-	-	-	-	-	-	-
3.118	-	-	-	-	-	-	-	-	-	-
3.477	-	-	-	-	-	-	-	-	-	-
3.103	-	-	-	-	-	-	-	-	-	-
3.038	-	-	-	-	-	-	-	-	-	-
2.897	-	-	-	-	-	-	-	-	-	-
3.452	-	-	-	-	-	-	-	-	-	-
2.9	-	-	-	-	-	-	-	-	-	-
3.185	-	-	-	-	-	-	-	-	-	-
3.439	-	-	-	-	-	-	-	-	-	-
3.182	-	-	-	-	-	-	-	-	-	-
3.17	-	-	-	-	-	-	-	-	-	-
3.456	-	-	-	-	-	-	-	-	-	-
3.372	-	-	-	-	-	-	-	-	-	-
3.497	-	-	-	-	-	-	-	-	-	-
3.29	-	-	-	-	-	-	-	-	-	-
3.323	-	-	-	-	-	-	-	-	-	-
3.449	-	-	-	-	-	-	-	-	-	-
3.358	-	-	-	-	-	-	-	-	-	-
3.35	-	-	-	-	-	-	-	-	-	-
2.911	-	-	-	-	-	-	-	-	-	-
2.743	-	-	-	-	-	-	-	-	-	-

Continues on next page

Table S14 – *Continued from previous page*

C-H	H-O	C-O	O-O	C-C	H-N	C-N	N-O	H-Z	C-Zn	O-Zn
3.152	-	-	-	-	-	-	-	-	-	-
Frame number : 8000										
2.966	2.823	-	-	3.352	-	-	-	-	-	-
3.331	2.685	-	-	3.462	-	-	-	-	-	-
3.069	2.944	-	-	3.292	-	-	-	-	-	-
3.005	3.16	-	-	3.481	-	-	-	-	-	-
3.327	-	-	-	3.454	-	-	-	-	-	-
2.87	-	-	-	3.485	-	-	-	-	-	-
3.467	-	-	-	3.405	-	-	-	-	-	-
3.071	-	-	-	3.238	-	-	-	-	-	-
3.431	-	-	-	3.351	-	-	-	-	-	-
2.826	-	-	-	3.312	-	-	-	-	-	-
3.044	-	-	-	3.35	-	-	-	-	-	-
3.421	-	-	-	-	-	-	-	-	-	-
3.383	-	-	-	-	-	-	-	-	-	-
3.395	-	-	-	-	-	-	-	-	-	-
2.974	-	-	-	-	-	-	-	-	-	-
3.442	-	-	-	-	-	-	-	-	-	-
3.146	-	-	-	-	-	-	-	-	-	-
3.072	-	-	-	-	-	-	-	-	-	-
3.273	-	-	-	-	-	-	-	-	-	-
3.202	-	-	-	-	-	-	-	-	-	-
3.122	-	-	-	-	-	-	-	-	-	-
3.446	-	-	-	-	-	-	-	-	-	-

Continues on next page

Table S14 – *Continued from previous page*

C-H	H-O	C-O	O-O	C-C	H-N	C-N	N-O	H-Z	C-Zn	O-Zn
2.938	-	-	-	-	-	-	-	-	-	-
3.172	-	-	-	-	-	-	-	-	-	-
2.987	-	-	-	-	-	-	-	-	-	-
3.222	-	-	-	-	-	-	-	-	-	-
3.436	-	-	-	-	-	-	-	-	-	-
3.364	-	-	-	-	-	-	-	-	-	-
2.899	-	-	-	-	-	-	-	-	-	-
2.756	-	-	-	-	-	-	-	-	-	-
3.489	-	-	-	-	-	-	-	-	-	-
3.229	-	-	-	-	-	-	-	-	-	-
3.282	-	-	-	-	-	-	-	-	-	-
3.077	-	-	-	-	-	-	-	-	-	-
2.982	-	-	-	-	-	-	-	-	-	-
2.848	-	-	-	-	-	-	-	-	-	-
3.167	-	-	-	-	-	-	-	-	-	-
2.644	-	-	-	-	-	-	-	-	-	-
3.141	-	-	-	-	-	-	-	-	-	-
3.309	-	-	-	-	-	-	-	-	-	-
3.147	-	-	-	-	-	-	-	-	-	-
3.451	-	-	-	-	-	-	-	-	-	-
3.179	-	-	-	-	-	-	-	-	-	-
3.29	-	-	-	-	-	-	-	-	-	-
3.422	-	-	-	-	-	-	-	-	-	-
3.107	-	-	-	-	-	-	-	-	-	-

Continues on next page

Table S14 – *Continued from previous page*

C-H	H-O	C-O	O-O	C-C	H-N	C-N	N-O	H-Z	C-Zn	O-Zn
3.002	-	-	-	-	-	-	-	-	-	-
2.975	-	-	-	-	-	-	-	-	-	-
3.319	-	-	-	-	-	-	-	-	-	-
2.776	-	-	-	-	-	-	-	-	-	-
3.283	-	-	-	-	-	-	-	-	-	-
3.38	-	-	-	-	-	-	-	-	-	-
3.47	-	-	-	-	-	-	-	-	-	-
Frame number : 9000										
3.36	3.476	-	-	3.498	-	-	-	-	-	-
3.14	3.208	-	-	3.291	-	-	-	-	-	-
3.105	3.218	-	-	3.36	-	-	-	-	-	-
3.29	3.209	-	-	3.415	-	-	-	-	-	-
3.468	3.486	-	-	3.45	-	-	-	-	-	-
3.318	3.412	-	-	3.447	-	-	-	-	-	-
2.73	2.897	-	-	3.443	-	-	-	-	-	-
2.5	3.259	-	-	3.458	-	-	-	-	-	-
3.472	-	-	-	3.3	-	-	-	-	-	-
3.35	-	-	-	3.212	-	-	-	-	-	-
2.978	-	-	-	3.467	-	-	-	-	-	-
2.667	-	-	-	3.478	-	-	-	-	-	-
2.789	-	-	-	-	-	-	-	-	-	-
3.254	-	-	-	-	-	-	-	-	-	-
3.148	-	-	-	-	-	-	-	-	-	-
3.494	-	-	-	-	-	-	-	-	-	-

Continues on next page

Table S14 – *Continued from previous page*

C-H	H-O	C-O	O-O	C-C	H-N	C-N	N-O	H-Z	C-Zn	O-Zn
3.058	-	-	-	-	-	-	-	-	-	-
3.199	-	-	-	-	-	-	-	-	-	-
3.423	-	-	-	-	-	-	-	-	-	-
3.087	-	-	-	-	-	-	-	-	-	-
3.254	-	-	-	-	-	-	-	-	-	-
3.06	-	-	-	-	-	-	-	-	-	-
3.006	-	-	-	-	-	-	-	-	-	-
2.777	-	-	-	-	-	-	-	-	-	-
3.414	-	-	-	-	-	-	-	-	-	-
3.252	-	-	-	-	-	-	-	-	-	-
2.74	-	-	-	-	-	-	-	-	-	-
2.864	-	-	-	-	-	-	-	-	-	-
3.025	-	-	-	-	-	-	-	-	-	-
3.127	-	-	-	-	-	-	-	-	-	-
3.425	-	-	-	-	-	-	-	-	-	-
3.457	-	-	-	-	-	-	-	-	-	-
3.056	-	-	-	-	-	-	-	-	-	-
3.213	-	-	-	-	-	-	-	-	-	-
2.904	-	-	-	-	-	-	-	-	-	-
3.431	-	-	-	-	-	-	-	-	-	-
2.817	-	-	-	-	-	-	-	-	-	-
2.931	-	-	-	-	-	-	-	-	-	-
3.037	-	-	-	-	-	-	-	-	-	-
2.812	-	-	-	-	-	-	-	-	-	-

Continues on next page

Table S14 – *Continued from previous page*

C-H	H-O	C-O	O-O	C-C	H-N	C-N	N-O	H-Z	C-Zn	O-Zn
2.988	-	-	-	-	-	-	-	-	-	-
2.846	-	-	-	-	-	-	-	-	-	-
3.435	-	-	-	-	-	-	-	-	-	-
3.132	-	-	-	-	-	-	-	-	-	-
3.257	-	-	-	-	-	-	-	-	-	-
3.195	-	-	-	-	-	-	-	-	-	-
2.978	-	-	-	-	-	-	-	-	-	-
3.288	-	-	-	-	-	-	-	-	-	-
2.877	-	-	-	-	-	-	-	-	-	-
3.268	-	-	-	-	-	-	-	-	-	-
3.096	-	-	-	-	-	-	-	-	-	-
2.625	-	-	-	-	-	-	-	-	-	-
3.233	-	-	-	-	-	-	-	-	-	-
3.269	-	-	-	-	-	-	-	-	-	-
2.669	-	-	-	-	-	-	-	-	-	-
Frame number : 10000										
2.818	3.461	-	-	3.493	-	-	-	-	-	-
3.227	3.216	-	-	3.353	-	-	-	-	-	-
3.061	3.339	-	-	3.43	-	-	-	-	-	-
2.671	2.898	-	-	-	-	-	-	-	-	-
3.312	2.751	-	-	-	-	-	-	-	-	-
2.666	-	-	-	-	-	-	-	-	-	-
3.278	-	-	-	-	-	-	-	-	-	-
3.458	-	-	-	-	-	-	-	-	-	-

Continues on next page

Table S14 – *Continued from previous page*

C-H	H-O	C-O	O-O	C-C	H-N	C-N	N-O	H-Z	C-Zn	O-Zn
3.143	-	-	-	-	-	-	-	-	-	-
3.195	-	-	-	-	-	-	-	-	-	-
2.873	-	-	-	-	-	-	-	-	-	-
3.31	-	-	-	-	-	-	-	-	-	-
2.763	-	-	-	-	-	-	-	-	-	-
2.926	-	-	-	-	-	-	-	-	-	-
2.997	-	-	-	-	-	-	-	-	-	-
2.838	-	-	-	-	-	-	-	-	-	-
3.431	-	-	-	-	-	-	-	-	-	-
3.059	-	-	-	-	-	-	-	-	-	-
3.197	-	-	-	-	-	-	-	-	-	-
3.148	-	-	-	-	-	-	-	-	-	-
3.407	-	-	-	-	-	-	-	-	-	-
3.038	-	-	-	-	-	-	-	-	-	-
3.426	-	-	-	-	-	-	-	-	-	-
3.169	-	-	-	-	-	-	-	-	-	-
3.393	-	-	-	-	-	-	-	-	-	-
3.28	-	-	-	-	-	-	-	-	-	-
3.117	-	-	-	-	-	-	-	-	-	-
3.408	-	-	-	-	-	-	-	-	-	-
3.3	-	-	-	-	-	-	-	-	-	-
3.037	-	-	-	-	-	-	-	-	-	-
3.314	-	-	-	-	-	-	-	-	-	-
3.305	-	-	-	-	-	-	-	-	-	-

Continues on next page

Table S14 – *Continued from previous page*

C-H	H-O	C-O	O-O	C-C	H-N	C-N	N-O	H-Z	C-Zn	O-Zn
2.811	-	-	-	-	-	-	-	-	-	-
3.316	-	-	-	-	-	-	-	-	-	-
3.268	-	-	-	-	-	-	-	-	-	-
3.129	-	-	-	-	-	-	-	-	-	-
2.897	-	-	-	-	-	-	-	-	-	-
3.163	-	-	-	-	-	-	-	-	-	-
3.352	-	-	-	-	-	-	-	-	-	-
3.276	-	-	-	-	-	-	-	-	-	-
2.834	-	-	-	-	-	-	-	-	-	-
3.272	-	-	-	-	-	-	-	-	-	-
3.366	-	-	-	-	-	-	-	-	-	-

References

[1] M. A. Addicoat, S. Fukuoka, A. J. Page, S. Irle, *Journal of Computational Chemistry* **2013**, *34*, 2591–2600.
